# Estimation of groin recurrence risk in patients with squamous cell vulvar carcinoma by the assessment of marker gene expression in the lymph nodes

**DOI:** 10.1186/1471-2407-12-223

**Published:** 2012-06-06

**Authors:** Magdalena Kowalewska, Jakub Radziszewski, Krzysztof Goryca, Mateusz Bujko, Malgorzata Oczko-Wojciechowska, Michal Jarzab, Janusz Aleksander Siedlecki, Mariusz Bidzinski

**Affiliations:** 1 Department of Molecular Biology, Maria Sklodowska-Curie Memorial Cancer Centre and Institute of Oncology, Roentgena, 5, Warsaw 02-781, Poland; 2 Department of Surgery, Maria Sklodowska-Curie Memorial Cancer Centre and Institute of Oncology, Wawelska 15, Warsaw 00-973, Poland; 3 Department of Oncological Genetics, Maria Sklodowska-Curie Memorial Cancer Centre and Institute of Oncology, Roentgena 5, Warsaw 02-781, Poland; 4 Laboratory of Bioinformatics and Systems Biology, Maria Sklodowska-Curie Memorial Cancer Centre and Institute of Oncology, Roentgena 5, Warsaw 02-781, Poland; 5 Department of Nuclear Medicine and Endocrine Oncology, Maria Sklodowska-Curie Memorial Cancer Center and Institute of Oncology, Wybrzeze Armii Krajowej 15, Gliwice 44-100, Poland; 6 Department of Gynecological Oncology, Maria Sklodowska-Curie Memorial Cancer Centre and Institute of Oncology, Roentgena, 5, Warsaw 02-781, Poland

**Keywords:** Vulvar carcinoma, Lymph node, Microarray, Expression marker, Real-time RT-PCR

## Abstract

**Background:**

Regional lymph node (LN) status is a well-known prognostic factor for vulvar carcinoma (VC) patients. Although the reliable LN assessment in VC is crucial, it presents significant diagnostic problems. We aimed to identify specific mRNA markers of VC dissemination in the LN and to address the feasibility of predicting the risk of nodal recurrence by the patterns of gene expression.

**Methods:**

Sentinel and inguinal LN samples from 20 patients who had undergone surgery for stage T_1-3_, N_0-2,_ M_0_ primary vulvar squamous cell carcinoma were analyzed. Gene expression profiles were assessed in four metastatic [LN(+)] and four histologically negative [LN(−)] lymph node samples obtained from four VC patients, by the Affymetrix U133 Plus 2.0 gene expression microarrays. Of the set of genes of the highest expression in the metastatic LNs compared to LN(−), seven candidate marker genes were selected: *PERP*, *S100A8*, *FABP5*, *SFN*, *CA12, JUP* and *CSTA*, and the expression levels of these genes were further analyzed by the real-time reverse transcription polymerase chain reaction (qRT-PCR) in 71 LN samples.

**Results:**

All of the seven genes in question were significantly increased in LN(+) compared to LN(−) samples. In the initial validation of the seven putative markers of metastatic LN, the Cox proportional hazard model pointed to *SFN*, *CA12* and *JUP* expression to significantly relate to the time to groin recurrence in VC patients.

**Conclusions:**

Our findings first provided evidence that *SFN*, *CA12* and *JUP* have a potential of marker genes for the prediction of the groin recurrence LN in VC patients.

## Background

Vulvar carcinoma (VC) is a rare genital malignancy with age-standardized incidence rates (ASR) ranging worldwide between 0.5 and 1.5 per 100,000 [1]. The current ASR in Poland is 1.0, and 436 new cases of VC were diagnosed and 232 VC-related deaths were recorded in 2009 (age-standardized mortality rate 0.5) [2]. Squamous cell carcinoma is the most common histological type of VC, representing approximately 90% of lesions. VC spreads primarily by the local expansion and *via* the lymphatic system.

Lymph node (LN) status is the most important prognostic factor in VC patients [3]. Up to 24% of patients with LNs clinically considered to be normal have metastases, while more than 20% of patients with enlarged nodes in clinical examination are found metastases-free in the histopathologic assessment [4]. Therefore, in many centres the VC staging comprises an ultrasound-guided fine-needle biopsy of the suspicious groin LNs and cytologic evaluation of the regional LN status. In early stage VC patients, the probability of a positive inguinal LN finding is only 11-25% [5]. This means that if all women with early VC were assigned to lymphadenectomy, approximately 80% of them would undergo overtreatment. At present, the concept of sentinel LN biopsy in early stage VC is becoming increasingly accepted while the problem of optimal treatment of patients with metastatic regional LNs is still being discussed. Another issue is the assessment of the risk of metastases in non-sentinel LNs after removal of all positive sentinel LNs.

Finding a method of an accurate and sensitive pre- or perioperative determination of pathologic status of groin nodes would be of a great value to aid therapeutic decisions in early stages of VC. Time-consuming, routine pathological evaluation of hematoxylin- and eosin-stained (H&E) LN sections lacks sensitivity. Therefore, a real-time reverse-transcription PCR (qRT-PCR) is being used in various tumours to determine the presence of metastatic cells in LNs [6,7]. However, before the qRT-PCR-based molecular staging of VC can be applied, clinically relevant markers [8] need to be identified. In this study, to identify specific mRNA markers of VC dissemination into LNs, expression microarrays were employed.

## Materials

The material was obtained from 20 patients treated for vulvar carcinoma in the Maria Sklodowska-Curie Memorial Cancer Centre and Institute of Oncology in Warsaw between March 2003 and September 2006. Patients with microscopically confirmed vulvar squamous cell carcinoma in clinical stage T_1-3_, N_0-2,_ M_0_ and with no prior treatment for this or any other malignancy were enrolled. The selected characteristics, including the TNM categories according to the AJCC (American Joint Committee on Cancer TNM staging system), of VC patients are presented in Table 1. Surgery and LN identification were performed as described previously [9]. The median LN count was 5 nodes in the right groin, range 2–13, while in the left groin the median LN count was 5.5 nodes, range 0–15. The study was approved by the Independent Ethics Committee of the Maria Sklodowska-Curie Memorial Cancer Centre and Institute of Oncology in Warsaw and all patients gave their informed consent. LN specimens were divided into two parts: one part was examined histologically as described previously [10], the other was frozen in liquid nitrogen immediately after collection and stored at −70°C until RNA isolation. Median follow-up time, determined from the date of surgery to the date of death or the date of the last interview, was 1.2 years (range 0.43 – 6.29).

**Table 1:** Individual patient data

**Patient No.**	**Age (years)**	**TNM Stage**	**Histological Grade**	**Histological subtype**	**HPV Status/Type**	**Groin recurrence**
5	59.3	T2N0M0	n.d.	K	n.d.	occurred
7	81.3	T2N1M0	G2	K	n.d.	occurred
8	61.0	T2N1M0	G2	K	n.d.	occurred
15*	77.2	T2N2M0	G2	K	negative	occurred
18	78.3	T3N2M0	G1	nK	negative	not observed
19	51.1	T2N1M0	G3	nK	negative	not observed
21*	93.9	T2N1M0	G2	K	negative	not observed
23	71.5	T2N1M0	G2	K	negative	not observed
25	75.3	T2N0M0	G1	K	negative	not observed
26	56.8	T2N0M0	G1	K	negative	not observed
30	45.3	T2N0M0**	G3	nK	HPV16	not observed
34	71.2	T2N1M0	G2	nK	negative	not observed
41	80.6	T2N1M0	G1	K	negative	occurred
45	76.1	T2N0M0	G3	nK	negative	not observed
46*	64.5	T2N1M0	G3	K	negative	occurred
49	76.0	T2N0M0	G3	nK	negative	occurred
51	56.7	T2N2M0	G3	K	negative	occurred
53	70.3	T2N0M0**	G2	K	negative	occurred
58	52.4	T1bN2M0	G2	K	negative	not observed
61*	76.6	T2N1M0	G2	nK	negative	not observed

Abbreviations: *K* - keratinising squamous cell carcinoma; *nK* - non-keratinising squamous cell carcinoma; *n.d.* - not determined; *- *LNs* of these patients were incorporated into microarray analysis, **- patients subjected to the unilateral lymphadenectomy.

Microarray analysis of gene expression profiles was performed in four pairs of LN samples from four VC patients. One metastatic LN [LN(+)] and one histologically negative [LN(−)], according to a routine pathological examination with H&E, was examined per each of the four patients. These patients had human papillomavirus (HPV)-negative tumours, as previously determined using the Linear Array HPV Detection Kit and Linear Array HPV Genotyping Test (Roche Molecular Systems, Inc) [10].

A total of 71 right and left LNs (sentinel and inguinal specimens), from 20 VC patients were included in the real-time RT-PCR analysis of expression of selected genes. In a routine pathological examination, 22 LNs were evaluated as LN(+) and the remaining 49 were histologically negative [LN(−)].

## Methods

### RNA isolation

Total RNA was isolated from approximately 200 mg of pulverised (with the Microdismembrator II, B Braun Biotech International) LN samples using Nucleospin RNA L kit (Macherey-Nagel), according to the manufacturer’s protocol. RNA quality was assessed using the Agilent 2100 Bioanalyzer and RNA 6000 Nano Chip Kit (Agilent Technologies).

### Microarray procedure

cDNA synthesis was carried out from 5 μg of RNA with the One-Cycle cDNA Synthesis Kit (Affymetrix). After purification (GeneChip Ample Cleanup Module), 7 μl of double-stranded cDNA were used for biotinylated cRNA synthesis with IVT Labeling Kit (Affymetrix). The quantity and quality of the obtained cRNA were assessed using the Agilent 2100 Bioanalyser and RNA 6000 Nano Chip Kit (Agilent Technologies). Labeled cRNA was purified using a GeneChip Sample Cleanup Module, fragmented and hybridized with the Affymetrix GeneChip Human Genome U133 Plus 2.0 arrays. Washing, staining with streptavidin-phycoerythrin conjugate and scanning of the arrays in the Affymetrix GeneChip 3000 scanner were performed as recommended by the Affymetrix Gene Expression Analysis Technical Manual.

### Microarray data analysis

All the arrays were normalized by the GCRMA algorithm using a Bioconductor [11] (version 2.8.1) package gcrma version 2.14.1. The expression levels were log_2_ transformed. We filtered out all the probe sets with signal level below 7.5 in at least 8 samples. Genes differentially expressed in involved [LN(+)] and uninvolved [LN(−)] lymph nodes were searched by a random-variance t-test, with the statistical significance threshold set at *P*<0.01. To reduce false-differential gene expression, a Benjamini-Hochberg [12] multiple testing correction was applied. The full dataset has been deposited in the Gene Expression Omnibus repository (accession no GSE28442).

The probe sets with differential expression in two samples - one LN(+) and one LN(−) - obtained from patient No 15 were chosen to be analyzed first with pumaDE function from PUMA package version 1.8.1 [13]. Differentially expressed probe sets were annotated with Gene Ontology (GO) terms (GO.db version 2.2.5) using the Bioconductor [11] packages *GOstats* (version 2.8.0) and package *annotate* (version 1.20.1). The significance of differential representation of GO terms between the specified lists of probe sets was determined by the hypergeometric test implemented in GOstats (version 2.8.0). *P* values returned by GOstats were corrected for testing of multiple hypotheses with the Benjamini–Hochberg method implemented in an R environment (version 2.8.1, The R Foundation for Statistical Computing; http://www.r-project.org). Adjusted *P* values of less than 0.05 were considered significant. The same procedure was applied to the three additional pairs of LN(+) and LN(−) samples from three patients (No. 21, 46 and 61).

This analysis has provided a set of candidate marker genes, which were further investigated to define their tumour specificity using *Genevestigator V3*, a web-based microarray database and analysis system [14].

### Real time RT-PCR

RNA was extracted from 71 LN samples and 1 μg from each sample was reverse-transcribed by using the RT^2^ First Strand Kit (C-03) from SA Biosciences. Custom PCR arrays (SA Biosciences) were used to simultaneously examine the mRNA levels of seven genes of interest according to the manufacturer's protocol.

The arrays also included primers for two housekeeping genes and three internal controls. Quantitative real-time PCR analysis was performed with the RT2 Real-Time PCR Master Mix (SA Biosciences) in the 7500 Fast Real-Time PCR System (Applied Biosystems), according to the manufacturer's instructions (SA Biosciences). The collected data were analyzed using threshold-cycle (Ct) values for the genes with the SDS 2.1 software (Applied Biosystems). Normalization was performed based on the mean values of two housekeeping genes, *HMBS* and *HSP90AB1*, and the relative amounts of RNA for each gene were calculated by the 2^−ΔCT^ method using DataAssist™ Software (Applied Biosystems). Expression levels for each gene were visualized using GraphPadPrism (La Jolla, CA, USA).

### Statistical analysis

The significance of difference between the selected gene expression level in each of LN(+) and LN(−) samples was assessed by using Wilcoxon test. *P* < 0.01 was considered significant.

The Cox proportional-hazard model with Bonferroni correction for multiple hypotheses testing was applied to estimate the effect of gene expression levels in LN samples on the time to groin recurrence (TTR). The highest expression value of a given gene of all obtained in different LN samples of the same patient was chosen to be included in the statistical analysis. TTR was calculated from the date of primary surgery to the date of groin recurrence. MedCalc (Mariakerke, Belgium) software was used to generate Kaplan-Meier curves and to compare TTR using two sided log-rank analysis. The median of maximum gene expression levels (again, in one of the nodes examined per patient) in LNs from all the VC patients enrolled in the study was arbitrarily chosen as a cut-off value for patient stratification. *P* < 0.05 was considered significant.

## Results

### Identification of expressed genes associated with LN metastasis

The microarray data from two LN samples, one LN(+) and one LN(−), from one patient (case No. 15) with a rapid disease progression, were analyzed using pumaDE function from the PUMA package 1.8.1 [13]. The filtered dataset comprised 907 probe-sets with *P* value below 0.05 (five percent Benjamini-Hochberg false discovery rate) differentially expressed in these two LN samples. This list included only up-regulated genes in LN(+) samples as no genes were found to be reduced in LNs(+) compared to LNs(−).

To evaluate which biological processes are represented within the obtained probe set list, the differentially expressed genes were annotated with the Gene Ontology (GO) terms. The most significantly over-represented ontology classes for the genes differentiating between LN(+) and LN(−) tissue are given in Table 2. The most up-regulated genes include those coding for epidermal molecules as well as molecules associated with cell adhesion, locomotory behaviour and inflammatory response.

**Table 2:** Significant Gene Ontology categories overrepresented among differentially expressed genes

**GO category**	**GO term**	**Exp. No.**	***P***	***Pa***
GO:0007155	cell adhesion	28.3	1,52E-09	2,07E-06
GO:0008544	epidermis development	2.7	6,70E-09	4,54E-06
GO:0032502	developmental process	22.2	1,85E-06	0,000837
GO:0007275	multicellular organismal development	80.8	2,74E-06	0,000928
GO:0048730	epidermis morphogenesis	1.5	1,15E-05	0,00311
GO:0030216	keratinocyte differentiation	1.0	3,35E-05	0,006311
GO:0007626	locomotory behavior	7.2	3,45E-05	0,006311
GO:0006954	inflammatory response	11.6	3,72E-05	0,006311

Abbreviations: *GO* – Gene Ontology; *Exp. No*. - the expected number of differentially expressed genes (probesets) to be found among genes annotated to each GO term; *P* - *P* value; *Pa* - adjusted *P* value.

### Gene selection for validation

Based on the results of microarray analysis by the pumaDE function of PUMA package 1.8.1, we aimed to select genes with the highest fold-change of expression between LN(+) and LN(−) obtained from the patient No. 15, and lowest significant adjusted *P* values, to be assessed by the quantitative PCR. Genes with over two-fold differences in expression levels were selected from the list and further categorized into different functions and cellular processes. The genes significantly differentially expressed (at *P* value below 0.01) in the analyzed LN obtained from patient No. 15 were represented by 408 probe sets (Additional file: 1 Table S1). The genes selected were those with the highest fold-change values, represented by specific probe sets (named with the suffix ‘_at’), and those belonging to different GO categories. The group of genes of interest was further reduced to those which also proved to differentiate LNs(+) from LNs(−) in the PUMA analysis of microarray data of the three additional pairs of nodes from three consecutive patients (No. 21, 46 and 61). A list of the genes differentially expressed in LN(+) and LN(−) samples obtained from these four VC patients is presented in the Additional file: 2 Table S2.

Next, only the genes up-regulated in the four LNs(+) obtained from the four VC patients were analyzed in Genevestigator V3 system. Genevestigator allowed to validate their increased expression in vulva, as this repository contains high quality microarray expression data from experiments performed on 27 non-cancer vulvar tissue samples (including nine specimens of vulvar intraepithelial neoplasia, VIN). The genes that showed high signal intensities in these 27 microarray data sets were selected. The probe sets were chosen on the basis of the highest % Present” value generated in the Genevestigator. These values represent a fraction of arrays in which the signal for a given probe set is above the background. Finally, seven genes were selected from the microarray studies: TP53 apoptosis effector - *PERP*, S100 calcium binding protein A8 - *S100A8*, fatty acid binding protein 5 (psoriasis-associated) - *FABP5*, stratifin - *SFN*, carbonic anhydrase 12 - *CA12*, junction plakoglobin - *JUP* and cystatin A - *CSTA*. Five out of the seven selected genes, namely, *S100A8FABP5SFNJUP* and *CSTA*, have been described by Hsiao et al. [15] as “vulva-selective” .

### Validation of gene expression levels by real-time RT-PCR

To investigate the reliability of the cDNA microarray results and to check if our gene selection was correct, the expression levels of seven genes, *PERP*, *S100A8*, *FABP5*, *SFN*, *CA12*, *JUP* and *CSTA*, were measured by the qRT-PCR in 22 LN(+) and 49 LN(−) samples.

**Figure 1:**
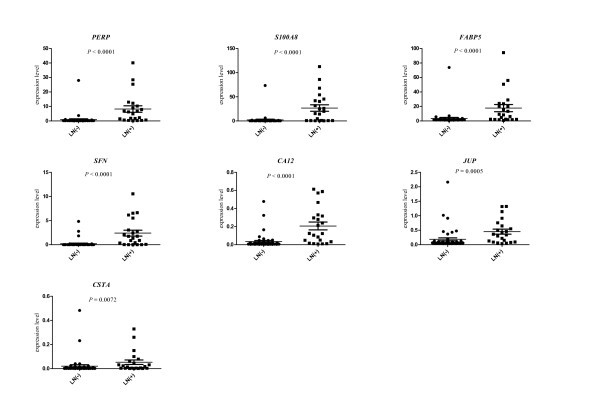
**The*PERP*,*S100A8*,*FABP5*,*SFN*,*CA12*,*JUP*and*CSTA*expression levels in lymph node samples, measured by qRT-PCR.** Abbreviations: LN(+) - involved lymph node, LN(−) - uninvolved lymph node sample, *P* – *P* value computed by the Wilcoxon test.

We confirmed the significant differences in the expression levels between uninvolved and involved (as assessed by routine histopathological analysis) of the all the seven genes in question (see Figure 1). As shown in Additional file: 3 Table S3, the expression levels of all of the seven genes tested by the qRT-PCR, in agreement with the microarray data, was lower in LN(−) than in LN(+).

### Correlations between gene expression levels and time to groin recurrence in VC patients

Subsequently, we examined a correlation between the expression levels of the seven selected genes and time to groin recurrence in VC patients using Cox's proportional hazard analysis. The highest expression value of a given gene of all obtained in different LN samples of the same patient was taken into account. Three genes, *SFN*, *CA12* and *JUP*, significantly correlated with TTR. High expression levels of the set of the four remaining genes in LNs were also associated with shorter TTR, but the trend did not reach statistical significance (Table 3).

**Table 3:** Impact of gene expression levels in LN samples on TTR and its significance

**Transcript**	**Coef.**	***P***
SFN	0.3119	0.01
CA12	3.8410	0.01
JUP	1.2405	0.01
CSTA	2.9463	0.17
S100A8	0.0075	0.39
FABP5	0.0061	0.59
PERP	0.0108	0.69

Abbreviations: *TTR* – time to groin recurrence, *Coef*. – Cox-model coefficient of modification of recurrence hazard by a given variable (i.e. gene expression); *P* – *P* value computed using Cox's proportional hazard analysis.

**Figure 2:**
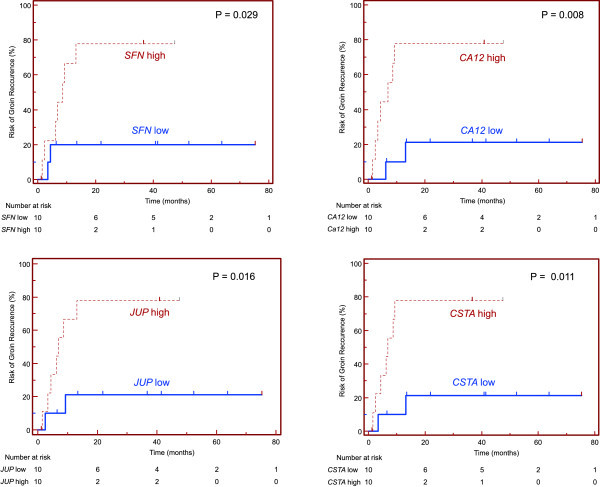
**Kaplan-Meier estimates of TTR according to*SFN*,*CA12 JUP*and*CSTA*expression status (high*vs*low) in regional LNs.** Abbreviations: *P* – *P* value calculated using the Mantel-Cox log-rank test; high - expression level, i.e. above the cut-off value; low - expression level, i.e. below the cut-off value (i.e. the median of maximum gene expression levels - as measured in one node per VC patient.

Finally, patients were stratified by the arbitrarily chosen cut-off values, i.e. the medians of maximum gene expression levels in LNs from all the VC patients enrolled in the study (again, one node with the highest gene expression was taken into account per patient). Kaplan-Meier analysis was used for comparison of TTR between the groups of patients stratified according to gene expression status, i.e. high (above the cut-off value) *vs* low gene expression status (below the cut-off value) and irrespective of the histopathological evaluation of their LNs status. Initially, Kaplan-Meier estimates of TTR were used for the evaluation of the prognostic value of the *SFN*, *CA12* and *JUP* gene expression, the genes whose expression was found to correlate significantly with TTR in above-mentioned COX-model analysis. Further, an analogous Kaplan-Meier analysis of the remaining four genes revealed the prognostic value of the expression level of *CSTA* gene. The Kaplan-Meier plots show the association of TTR with the LN expression levels of *SFN*, *CA12*, *JUP* and *CSTA* in VC patients, i.e. the expression levels of these genes predicted TTR (see Figure 2). The results of comparison of the Kaplan-Meier estimates for TTR depending on the expression level of *SFN*, *CA12*, *JUP* and *CSTA* genes, together with the cut-off values used for this analysis, are presented in Table 4. The differences in TTR between the groups of VC patients stratified by *PERP*, *S100A8* and *FABP* gene expression levels were not significant (Kaplan-Meier curves not shown).

**Table 4:** The results of the comparison of Kaplan-Meier estimates for TTR for VC patients according to their LN gene expression levels

**Transcript**		**TTR for patients with high expression level**		**TTR for patients with low expression level**	***P***
	Cut-off Value	Median TTR (months)	HR; CI	Median TTR (months)	
*SFN*	0.4815	8.7	0.21; 95% CI, 0.06-0.86	not reached	0.029
*CA12*	0.23	6.9	0.16; 95% CI, 0.04-0.61	not reached	0.008
*JUP*	0.4815	6.89	0.18; 95% CI, 0.05-0.74	not reached	0.016
*CSTA*	0.03	6.89	0.17; 95% CI, 0.04-0.66	not reached	0.011

Abbreviations: *TTR* – time to groin recurrence; *HR* – hazard ratio; *CI* – confidence interval; *Cut-off Value* - the arbitrarily chosen cut-off for gene expression value used for patient stratification; *P* – *P* value calculated with the use of the log-rank test.

## Discussion

Lymph node status is the most important prognostic factor in patients with VC [16]. Routine pathological staging is limited to the microscopic evaluation of H&E-stained LN sections. Unfortunately, small tumour foci may occasionally be missed by the pathologist in this examination [17]. As stated by Regauer [18], sentinel LNs of VC patients with even single tumour cells should be regarded as positive. Thus, to improve detection of LN metastases, pathological ultrastaging should ideally be performed [19,20]. This histological work-up of LNs involves serial sectioning and immunohistochemical analysis; the procedure is labour-intensive and time-consuming. Real-time RT-PCR technology is more sensitive, as it allows to detect a very small number of cells in larger volumes of previously pulverized (or homogenized) tissues. However, this method carries some disadvantages, for example it makes morphologic evaluation of the analyzed samples impossible. Therefore, a careful marker selection for RT-PCR analysis is a fundamental issue.

Since there are no molecular markers for diagnosing VC dissemination, we aimed to identify the potential mRNA markers of VC dissemination into LNs, and to verify their prognostic value by correlating their levels with time to groin recurrence. We used oligonucleotide microarrays which measure the expression level of over 47,000 transcripts and variants of human genes.

At first, the PUMA package, enabling a comparison between small-sample gene expression data [21] was employed, to analyze two samples obtained from one patient, who underwent a rapid progression of the disease during the follow-up time, with local recurrence, and groin recurrence in the same groin where the LN(+), and died due to cancer progression. The PUMA analysis produced a list of genes differentially expressed in LN(+) and LN(−). The most significantly over-represented gene ontology (GO) classes in the list included epidermal molecules. Thus, some of the differentially expressed genes are those specifically expressed in the primary tumour site. This finding testifies the methodology used for the comparison of the single microarray experiments. Similar results were obtained for the three additional pairs of LNs obtained from three patients.

Interestingly, gene expression signature distinguishing between the four pairs of histologically positive and negative LNs comprised no genes down-regulated in the metastatic LNs of the same patients. A possible explanation of this observation is that only transcripts that were highly up-regulated in the metastatic cells could be distinguished from the background of the numerous non-cancer cells as an “added value” while down-regulated transcripts, i.e. transcripts’ deprivation, would not be noticed in this background environment.

The consecutive steps of narrowing-down of the list of the differentially expressed in the four LNs obtained from the four VC patients genes led us to the choice of seven genes, namely *PERPS100A8FABP5SFNCA12JUP* and *CSTA,* to be validated by the real time RT-PCR. Gene expression data of 27 non-cancer vulvar tissue samples deposited in the Genevestigator V3 system [22,23] have shown that these seven genes are regularly up-regulated in vulvar tissue. In addition, Hsiao et al. [15], who created a compendium of tissue-selective genes based on microarray data, described five of these genes as “vulva-selective.” This strongly supports tissue specificity of the selected genes for the primary tumour site.

To our knowledge, this is the first time that these genes have been linked to VC dissemination. PERP, as a component of intercellular desmosome junctions, plays a role in epithelial integrity and cell-cell adhesion [24] and constitutes a proapoptotic transcriptional target of TP53 [25]. PERP-deficiency promotes cancer by enhancing cell survival, desmosome loss, and inflammation [26]. S100 calcium binding protein A8, S100A8, belongs to S100 proteins, a family of EF-hand signalling proteins [27]. The complex of S100A8 and S100A9 called calprotectin induces a proinflammatory and thrombogenic response, and is involved in danger signalling; what is more, its stimulation results in a loss of cell–cell contacts [28,29]. S100A8/S100A9 expression is increased in patients with various tumours, being involved in invasion and migration processes [30,31]. S100A8/9 expression is minimal in normal epidermis and is elevated in skin diseases [32]. However, Dell'oste et al. [33] showed that in HPV-immortalized keratinocytes S100A8/9 expression was downregulated. Fatty acid binding protein 5 (psoriasis-associated, FABP5), found in epidermal cells, was first identified to be up-regulated in psoriasis keratinocytes [34]. In cancer cells, FABP5 increases cell proliferation and invasiveness, as recently demonstrated in oral squamous cell carcinoma [35] where its expression may be HPV-related [36]. In non-cancer keratinocytes FABP5 elevation may be necessary for the activation of cell motility during epidermal wound healing [37]. Stratifin, SFN (14-3-3 sigma), plays a role in various cellular processes being the most cancer-associated 14-3-3 isoform [38]. 14-3-3 sigma expression is lost in numerous tumours [38], including VC [39], while its increased expression could be associated with a loss of TP53 function [40,41]. However, Wang et al. [42] found high levels of SFN detected immunohistochemically in over 70% of analyzed VC tumors, significantly correlating to large tumor diameter and deep invasion. Importantly, 14-3-3 sigma protein is present in various normal epithelia and absent in LNs [43,44], and lymphocytes may express its mRNA at relatively low levels [45]. Carbonic anhydrase XII is one of the tumour-associated carbonic anhydrases. The enzyme is overexpressed under hypoxic conditions and constitutes a possible target for anticancer therapy [46,47]. Junction plakoglobin, JUP, a member of the catenin family, is present both in desmosomes and in intermediate junctions [48]. The majority of studies suggest that plakoglobin has a tumour suppressor role [49]. Cystatin A (stefin A), CSTA*,* functions as a cysteine protease inhibitor and plays a role in epidermal development and maintenance. Serum level of CSTA has been proposed as a tool predicting nodal stage and poor prognosis in nasopharyngeal carcinoma [50].

The comparison of LNs(+) with LNs(−) confirmed that LNs(+) express significantly more transcripts of *PERP*, *S100A8*, *FABP5*, *SFN*, *CA12, JUP* and *CSTA*. The results of PCR analysis showed particularly increased expression of *S100A8* and *SFN* in the metastatic LNs, where there was over 10-fold change of the mean expression level. Higher expression of *PERP*, *FABP5*, *CA12, JUP* and *CSTA* were also found, but the differences were lower.

The significance of gene expression levels in LN samples for time to groin recurrence (TTR) was statistically analyzed using Cox's proportional hazard model. A coefficient computed by this method for each variable expression of “predictor” genes indicates the direction and degree of flexing that the predictor has on the TTR curve. A positive value of coefficients indicated that larger values of the expression were associated with greater groin recurrence rates. Two genes, namely *SFN* and *CA12*, were found significantly predictive of such recurrence. High expression levels of the other genes of the set of five in LNs were also associated with shorter TTRs, but the trend did not reach statistical significance.

The log-rank test used for testing significance of the TTR functions in Kaplan-Meier analysis yielded significant *P* values for separating the two groups of VC patients with different TTR according to their LN gene expression levels of *SFN*, *CA12*, *JUP* and *CSTA*. Patients with low expression of these genes had a superior TTR values of not reached *versus* 6.9 to 8.7 months.

It is worth noting that our study had some important limitations. Firstly, only seven of the most differentially expressed genes in the microarray experiment were analyzed by the qRT-PCR. Notwithstanding, the remaining genes (see Additional file: 2 Table S2) are candidates for validation in further studies. Secondly, LN assessment was based on routine pathological evaluation with H&E staining, while the state-of-the-art would be to perform ultrastaging on the excised LNs. Lastly, Kaplan-Meier analysis of TTR that was applied to *SFN*, *CA12*, *JUP* and *CSTA* expression, should be considered as just an illustration of potential clinical use of the identified marker genes. Future studies should include more patients to assure a better assessment of the novel markers’ performance. Such studies should also provide better cut-off values for patients’ stratification than the arbitrarily chosen median expression levels in LNs, and enable the ROC curve analysis. Importantly, our microarray data were obtained for HPV-negative patients in order to exclude the interference of the infection with the expression results and to focus on metastasis-associated genes’ selection. However, patients with HPV infection should also be enrolled in future studies to assess the influence of HPV on the markers’ performance.

Groin recurrences from VC are often fatal. Unfortunately, “the optimum mode of treatment and predictive factors for patients with groin recurrences are unknown” [51]. The size of LN metastases correlates with survival in patients with early [52] and advanced [53] stage VC. On the other hand, “inguinofemoral lymphadenectomy can be avoided when the sentinel node is negative for disease” [54]. However, although Oonk and colleagues [52] reported that the risk of non-sentinel LN involvement increases with size of sentinel LN metastases, they were unable to determine the cut-off size below which the risk of non-sentinel LN metastases would be inconsiderable. Therefore, the authors concluded that all patients with sentinel LN metastases - regardless of their size - should undergo additional groin treatment. The risk of additional metastases when only isolated tumour cells are present in the sentinel LN is 4% [55]. Still, approximately 12% of early-stage VC patients with a negative sentinel LN develop local recurrence” [54,56].

For the reasons discussed above, novel means to better stratify early-stage VC patients in order to optimize treatment modality, i.e. a tool to decide who should not undergo lymphadenectomy *vs* who should receive additional treatment to the groin would be highly beneficial. Further validation of markers and development of molecular tests is indispensable for the reliable up- or down-staging of LNs. Our results may ideally be used to develop a test that could be used during surgery to decide whether to remove inguinal LNs, similarly to the RT-PCR based, FDA-approved GeneSearch™ Breast Lymph Node (BLN) Assay (Veridex, LLC, Warren, NJ), used for the rapid intra-operative detection of sentinel LN metastases in breast cancer. Such tests may reduce the need for a second surgery for the axillary LN dissection [57-59]. As sentinel node procedure was confirmed to be safe in the early-stage VC patients [54], tests similar to GeneSearch™ should also be of clinical utility for VC patients.

## Conclusions

To conclude, the performance of qRT-PCR assays employing the LN(+) marker genes that we have identified, *PERP*, *S100A8*, *FABP5*, *SFN*, *CA12, JUP* and *CSTA*, may provide a promising tool for intra-operative sentinel LN evaluation in VC patients. Moreover, all the above marker genes have a potential of prognostic biomarkers, however, before their incorporation into clinical setting further studies are necessary to confirm their prognostic value in the qRT-PCR assays.

## Competing interests

The authors declare that they have no competing interests.

## Conflicts of interest

The authors report no potential conflicts of interest.

## Authors' contributions

MK and JR designed and coordinated the study. MBidzinski, JAS and MJ participated in its coordination. MK wrote the manuscript. KG and MBujko performed the statistical analysis. MO-W and MK carried out the microarray and qRT-PCR experiments, respectively. All authors read and approved the final manuscript.

## Pre-publication history

The pre-publication history for this paper can be accessed here:

http://www.biomedcentral.com/1471-2407/12/223/prepub

## Supplementary Material

Additional file 1 **Table S2.** Genes differentially expressed in LN(+) and LN(−) sample obtained from VC patient No 15. Abbreviations: *P* - p value; cP - Benjamini-Hochberg corrected p value; FC - fold change of gene expression ratio in LN(+) and LN(−) sample; exp. - expression level. Click here for file

Additional file 2 **Table S3.** Genes differentially expressed in the four LN(+) and LN(−) sample pairs obtained from VC patients No 15, 21, 46 and 61. Abbreviations: *P* - p value; NA - not applicable. Click here for file

Additional file 3 **Table S1.** Mean levels of expression of *PERP*, *S100A8*, *FABP5*, *SFN*, *CA12*, *JUP* and *CSTA* in LN(+) and LN(−) samples, measured by qRT-PCR. Abbreviations: ME – mean expression level in all involved [LN(+)] and uninvolved [LN(−)] lymph node samples included in the qRT-PCR analysis. Click here for file
