# Supplementary material for: Estimation of groin recurrence risk in patients with squamous cell vulvar carcinoma by the assessment of marker gene expression in the lymph nodes
Source: BMC Cancer. 2012 Jun 6;12:223. doi: 10.1186/1471-2407-12-223 (PMC3414830; doi:10.1186/1471-2407-12-223)
Supplement: Additional file 1 — Table S2. Genes differentially expressed in LN(+) and LN(−) sample obtained from VC patient No 15. Abbreviations: P - p value; cP - Benjamini-Hochberg corrected p value; FC - fold change of gene expression ratio in LN(+) and LN(−) sample; exp. - expression level. [file 1471-2407-12-223-S2.doc]

**Supplementary Table 2.** Genes differentially expressed in LN(+) and LN(-) sample obtained from VC patient No 15.

| **Probe Set** | **Gene Symbol** | ***P*** | **c*P*** | **FC** | **Exp. in LN(+)** | **Exp. in LN(-)** |
| --- | --- | --- | --- | --- | --- | --- |
| 202917_s_at | S100A8 | 6.92E-05 | 0.00943898 | 9.605789149 | 13.6246046 | 4.01881544 |
| 206884_s_at | SCEL | 3.87E-05 | 0.00643745 | 10.36216096 | 10.1877216 | -0.1744394 |
| 33322_i_at | SFN | 3.87E-07 | 0.00017048 | 8.753949209 | 13.2614158 | 4.50746656 |
| 204580_at | MMP12 | 2.19E-05 | 0.00422827 | 8.561486535 | 12.0104198 | 3.44893329 |
| 211756_at | PTHLH | 3.15E-05 | 0.00545428 | 9.048991938 | 9.94368739 | 0.89469546 |
| 234700_s_at | RNASE7 | 3.69E-05 | 0.00621204 | 8.867700107 | 9.94804222 | 1.08034211 |
| 219630_at | PDZK1IP1 | 2.89E-06 | 0.00084987 | 8.465847177 | 9.99680334 | 1.53095616 |
| 210074_at | CTSL2 | 1.21E-07 | 6.84E-05 | 8.06397085 | 11.7203803 | 3.65640941 |
| 1552487_a_at | BNC1 | 4.74E-05 | 0.00744041 | 8.197786201 | 9.34845445 | 1.15066825 |
| 202286_s_at | TACSTD2 | 8.38E-08 | 5.04E-05 | 7.675350778 | 11.9355254 | 4.26017463 |
| 238017_at | RDHE2 | 1.99E-05 | 0.00394684 | 7.858120995 | 10.1443705 | 2.2862495 |
| 219795_at | SLC6A14 | 2.61E-05 | 0.00474176 | 7.820290239 | 9.38788478 | 1.56759454 |
| 217744_s_at | PERP | 1.63E-07 | 8.33E-05 | 7.412432227 | 12.5110554 | 5.09862318 |
| 204750_s_at | DSC2 | 2.18E-05 | 0.00422827 | 7.544052232 | 9.9007451 | 2.35669286 |
| 203963_at | CA12 | 5.47E-05 | 0.00815614 | 7.323618533 | 10.2910462 | 2.96742766 |
| 209863_s_at | TP63 | 6.30E-06 | 0.00157363 | 6.842863985 | 10.5050763 | 3.66221232 |
| 1556793_a_at | FAM83C | 3.94E-05 | 0.00649208 | 6.988916223 | 8.40873903 | 1.41982281 |
| 231867_at | ODZ2 | 2.89E-07 | 0.00013515 | 6.587335681 | 9.9840424 | 3.39670672 |
| 204971_at | CSTA | 7.25E-05 | 0.00980927 | 6.405881049 | 12.6891046 | 6.28322354 |
| 218186_at | RAB25 | 6.32E-05 | 0.00903453 | 6.626687374 | 9.04333548 | 2.4166481 |
| 201839_s_at | TACSTD1 | 5.91E-05 | 0.00861945 | 6.839205919 | 8.21709762 | 1.3778917 |
| 232277_at | NA | 2.88E-05 | 0.00512988 | 6.903703098 | 7.93139144 | 1.02768834 |
| 228575_at | IL20RB | 4.86E-05 | 0.00753149 | 6.42504529 | 9.36262251 | 2.93757722 |
| 222392_x_at | PERP | 1.24E-06 | 0.00041212 | 6.221298662 | 12.7403581 | 6.51905939 |
| 1555778_a_at | POSTN | 1.41E-07 | 7.49E-05 | 5.970463514 | 11.4883171 | 5.51785354 |
| 223484_at | C15orf48 | 3.60E-08 | 2.59E-05 | 6.011010684 | 9.77422992 | 3.76321924 |
| 209395_at | CHI3L1 | 7.28E-05 | 0.00982161 | 5.910887271 | 10.0602874 | 4.14940014 |
| 226817_at | DSC2 | 4.85E-08 | 3.28E-05 | 5.844145222 | 12.0198757 | 6.17573052 |
| 222830_at | GRHL1 | 2.45E-05 | 0.00451135 | 5.918720157 | 9.69897629 | 3.78025613 |
| 219410_at | TMEM45A | 1.64E-15 | 1.79E-11 | 5.832527444 | 11.7236692 | 5.89114179 |
| 210735_s_at | CA12 | 1.01E-05 | 0.00230112 | 5.920183377 | 9.11400476 | 3.19382138 |
| 228010_at | PPP2R2C | 2.62E-06 | 0.00078566 | 5.841278601 | 9.59284298 | 3.75156438 |
| 232165_at | EPPK1 | 2.91E-05 | 0.00514054 | 5.787415941 | 9.51157292 | 3.72415698 |
| 209301_at | CA2 | 3.56E-06 | 0.00101436 | 5.707269146 | 10.4315056 | 4.72423648 |
| 209309_at | AZGP1 | 2.58E-08 | 1.96E-05 | 5.723674159 | 9.8405256 | 4.11685144 |
| 200606_at | DSP | 8.44E-12 | 2.43E-08 | 5.582960934 | 12.8624213 | 7.27946035 |
| 238513_at | PRRG4 | 1.33E-06 | 0.00043126 | 5.636454366 | 9.77637741 | 4.13992304 |
| 202935_s_at | SOX9 | 5.81E-05 | 0.00853678 | 5.652088212 | 9.33332151 | 3.6812333 |
| 212992_at | AHNAK2 | 3.37E-06 | 0.00096759 | 5.560616837 | 10.2969695 | 4.73635265 |
| 1555383_a_at | POF1B | 6.45E-05 | 0.00911891 | 5.682947546 | 8.38815556 | 2.70520801 |
| 219836_at | ZBED2 | 2.41E-05 | 0.00449011 | 5.563228284 | 8.85930722 | 3.29607893 |
| 223121_s_at | SFRP2 | 2.40E-05 | 0.00449011 | 5.465758613 | 10.5566694 | 5.09091082 |
| 205713_s_at | COMP | 4.32E-07 | 0.00018019 | 5.380723096 | 9.82882052 | 4.44809743 |
| 222853_at | FLRT3 | 3.40E-05 | 0.00578161 | 5.457522779 | 8.70165061 | 3.24412783 |
| 226185_at | CDS1 | 6.63E-05 | 0.00929015 | 5.412575901 | 8.80807307 | 3.39549717 |
| 206643_at | HAL | 6.85E-05 | 0.00943875 | 5.524710946 | 7.92572651 | 2.40101556 |
| 232164_s_at | EPPK1 | 5.16E-10 | 8.05E-07 | 5.26229109 | 10.1040483 | 4.84175722 |
| 203407_at | PPL | 5.51E-06 | 0.00144129 | 5.226684223 | 10.3860215 | 5.15933726 |
| 213909_at | LRRC15 | 5.71E-05 | 0.00843599 | 5.281300705 | 8.3232072 | 3.04190649 |
| 209212_s_at | KLF5 | 1.32E-05 | 0.0027693 | 5.162534612 | 9.67180177 | 4.50926715 |
| 202733_at | P4HA2 | 5.26E-05 | 0.00799012 | 5.070791945 | 9.7245425 | 4.65375055 |
| 202267_at | LAMC2 | 2.67E-05 | 0.00478553 | 4.954553451 | 10.1389997 | 5.18444626 |
| 211668_s_at | PLAU | 3.72E-05 | 0.00624682 | 4.971601319 | 9.47080453 | 4.49920321 |
| 215465_at | ABCA12 | 2.29E-05 | 0.00437853 | 5.112213911 | 7.51217372 | 2.39995981 |
| 210809_s_at | POSTN | 4.27E-12 | 1.46E-08 | 4.834692064 | 12.750661 | 7.9159689 |
| 201286_at | SDC1 | 4.31E-08 | 2.99E-05 | 4.814782609 | 10.7151217 | 5.90033911 |
| 227140_at | NA | 1.26E-06 | 0.00041288 | 4.828130597 | 9.90736584 | 5.07923524 |
| 213797_at | RSAD2 | 5.28E-05 | 0.00799512 | 4.81145528 | 9.75200494 | 4.94054966 |
| 210511_s_at | INHBA | 2.06E-06 | 0.0006352 | 4.78362149 | 9.21400205 | 4.43038056 |
| 209875_s_at | SPP1 | 7.76E-13 | 3.86E-09 | 4.710540402 | 12.6780953 | 7.9675549 |
| 223541_at | HAS3 | 4.73E-05 | 0.00744041 | 4.791562237 | 8.67386828 | 3.88230604 |
| 225612_s_at | B3GNT5 | 1.28E-07 | 7.07E-05 | 4.720247274 | 9.8729953 | 5.15274803 |
| 203789_s_at | SEMA3C | 5.49E-09 | 5.77E-06 | 4.69335305 | 10.579573 | 5.88621996 |
| 202411_at | IFI27 | 9.96E-10 | 1.32E-06 | 4.592062355 | 12.3211182 | 7.72905581 |
| 236313_at | CDKN2B | 7.43E-09 | 7.26E-06 | 4.624866635 | 9.35520504 | 4.73033841 |
| 202311_s_at | COL1A1 | 6.34E-05 | 0.00903453 | 4.592408879 | 10.1217731 | 5.52936418 |
| 203780_at | MPZL2 | 1.57E-10 | 3.04E-07 | 4.578050364 | 9.84716931 | 5.26911895 |
| 201015_s_at | JUP | 8.88E-07 | 0.00032794 | 4.523194026 | 11.1109641 | 6.58777011 |
| 223122_s_at | SFRP2 | 5.04E-08 | 3.32E-05 | 4.458844899 | 11.8566162 | 7.39777133 |
| 201250_s_at | SLC2A1 | 7.78E-10 | 1.06E-06 | 4.441008838 | 10.6463759 | 6.2053671 |
| 203153_at | IFIT1 | 1.77E-09 | 2.15E-06 | 4.418499488 | 11.4221276 | 7.00362809 |
| 215646_s_at | VCAN | 1.26E-06 | 0.00041288 | 4.431893342 | 9.68669659 | 5.25480325 |
| 205807_s_at | TUFT1 | 2.48E-06 | 0.00075878 | 4.430432702 | 9.23431152 | 4.80387882 |
| 228293_at | DEPDC7 | 3.76E-05 | 0.00629353 | 4.532311819 | 7.57383316 | 3.04152134 |
| 206199_at | CEACAM7 | 2.63E-05 | 0.00476382 | 4.5251661 | 7.58621326 | 3.06104716 |
| 202345_s_at | FABP5 | 3.50E-09 | 3.98E-06 | 4.369962129 | 13.2496641 | 8.87970201 |
| 235733_at | NA | 5.76E-06 | 0.00148297 | 4.42356387 | 8.87428618 | 4.45072231 |
| 209211_at | KLF5 | 1.24E-06 | 0.00041212 | 4.34829079 | 9.75119255 | 5.40290176 |
| 217901_at | DSG2 | 1.25E-05 | 0.00265527 | 4.308863116 | 9.70284651 | 5.39398339 |
| 203184_at | FBN2 | 2.42E-05 | 0.00449011 | 4.304231127 | 8.3769155 | 4.07268437 |
| 203819_s_at | IGF2BP3 | 6.27E-06 | 0.00157362 | 4.286014519 | 8.57714812 | 4.2911336 |
| 225016_at | APCDD1 | 9.60E-07 | 0.00034939 | 4.123789889 | 10.4584183 | 6.33462844 |
| 219250_s_at | FLRT3 | 4.09E-06 | 0.00112833 | 4.164091503 | 8.50980573 | 4.34571423 |
| 212488_at | COL5A1 | 3.85E-07 | 0.00017048 | 4.114294199 | 10.4128702 | 6.29857601 |
| 205483_s_at | ISG15 | 3.31E-10 | 5.54E-07 | 4.084218584 | 11.6122636 | 7.528045 |
| 215243_s_at | GJB3 | 2.40E-05 | 0.00449011 | 4.141796098 | 8.45209126 | 4.31029516 |
| 211571_s_at | VCAN | 2.44E-10 | 4.30E-07 | 4.10227176 | 9.70164013 | 5.59936837 |
| 209365_s_at | ECM1 | 1.23E-05 | 0.00264796 | 4.094158468 | 9.52424662 | 5.43008815 |
| 219850_s_at | EHF | 5.15E-05 | 0.00788014 | 4.264869454 | 6.75118103 | 2.48631157 |
| 238439_at | ANKRD22 | 6.16E-05 | 0.00890813 | 4.023578335 | 8.99524153 | 4.97166319 |
| 204620_s_at | VCAN | 1.39E-07 | 7.47E-05 | 3.959766574 | 10.8734638 | 6.91369718 |
| 1553764_a_at | JUB | 1.36E-05 | 0.00281956 | 3.96518013 | 9.12298944 | 5.15780931 |
| 227461_at | STON2 | 1.01E-09 | 1.32E-06 | 3.941933604 | 9.89032472 | 5.94839112 |
| 225806_at | JUB | 9.54E-06 | 0.00220903 | 3.944628994 | 9.44300227 | 5.49837328 |
| 239272_at | MMP28 | 4.13E-05 | 0.00670089 | 3.984194551 | 8.16112446 | 4.17692991 |
| 229400_at | HOXD10 | 1.17E-05 | 0.00255627 | 3.960533607 | 8.23803359 | 4.27749998 |
| 242625_at | RSAD2 | 2.75E-06 | 0.00081315 | 3.910723448 | 10.0347957 | 6.1240723 |
| 203820_s_at | IGF2BP3 | 3.24E-06 | 0.00094251 | 3.93985696 | 8.47617454 | 4.53631758 |
| 219083_at | SHQ1 | 1.18E-05 | 0.00255627 | 3.881858949 | 10.9187448 | 7.03688581 |
| 226237_at | NA | 6.69E-11 | 1.52E-07 | 3.874414366 | 9.5621784 | 5.68776403 |
| 1554252_a_at | LASS3 | 3.35E-05 | 0.00574672 | 3.929543014 | 7.43543715 | 3.50589413 |
| 204747_at | IFIT3 | 7.48E-06 | 0.0018032 | 3.81435749 | 9.89862868 | 6.08427119 |
| 204619_s_at | VCAN | 9.88E-07 | 0.00034939 | 3.823638781 | 9.29098312 | 5.46734434 |
| 202404_s_at | COL1A2 | 3.54E-08 | 2.58E-05 | 3.770248659 | 12.8696769 | 9.09942825 |
| 226311_at | NA | 9.92E-07 | 0.00034939 | 3.785973706 | 9.08916397 | 5.30319026 |
| 201195_s_at | SLC7A5 | 6.72E-05 | 0.00939402 | 3.769297816 | 9.65291742 | 5.88361961 |
| 204052_s_at | SFRP4 | 6.82E-06 | 0.00168741 | 3.763387592 | 8.63540172 | 4.87201413 |
| 203560_at | GGH | 2.34E-09 | 2.77E-06 | 3.717662372 | 10.3117008 | 6.59403843 |
| 203961_at | NEBL | 6.39E-05 | 0.00907291 | 3.78446472 | 7.7552582 | 3.97079348 |
| 224799_at | NDFIP2 | 1.46E-07 | 7.69E-05 | 3.727390038 | 9.32458857 | 5.59719853 |
| 209758_s_at | MFAP5 | 1.74E-10 | 3.17E-07 | 3.688656266 | 10.4858875 | 6.79723124 |
| 228158_at | LOC645166 | 6.61E-05 | 0.00928604 | 3.728466745 | 7.96728009 | 4.23881334 |
| 201309_x_at | C5orf13 | 4.91E-05 | 0.00756795 | 3.696018886 | 8.84101646 | 5.14499758 |
| 228708_at | RAB27B | 7.37E-09 | 7.26E-06 | 3.67796694 | 9.36082729 | 5.68286035 |
| 228260_at | ELAVL2 | 7.44E-07 | 0.00028462 | 3.771845061 | 7.17586706 | 3.404022 |
| 212489_at | COL5A1 | 2.02E-07 | 9.86E-05 | 3.667926739 | 9.7307732 | 6.06284646 |
| 204990_s_at | ITGB4 | 3.83E-05 | 0.0063888 | 3.666318699 | 9.36584667 | 5.69952797 |
| 218717_s_at | LEPREL1 | 2.20E-05 | 0.00423691 | 3.67413239 | 8.88173344 | 5.20760105 |
| 201287_s_at | SDC1 | 4.05E-05 | 0.0066144 | 3.637021531 | 11.0745636 | 7.43754209 |
| 221731_x_at | VCAN | 1.34E-09 | 1.67E-06 | 3.630754115 | 10.6842554 | 7.05350124 |
| 229635_at | NA | 2.96E-05 | 0.00518069 | 3.717810866 | 7.39912726 | 3.68131639 |
| 213287_s_at | KRT10 | 3.05E-07 | 0.00014139 | 3.610436288 | 12.4706801 | 8.86024386 |
| 203726_s_at | LAMA3 | 2.39E-08 | 1.84E-05 | 3.617917926 | 10.033277 | 6.41535907 |
| 230746_s_at | STC1 | 9.92E-07 | 0.00034939 | 3.608000613 | 9.09854079 | 5.49054018 |
| 203962_s_at | NEBL | 2.56E-05 | 0.0046647 | 3.606213593 | 7.89037499 | 4.2841614 |
| 209803_s_at | PHLDA2 | 8.66E-07 | 0.00032197 | 3.551440535 | 9.49453078 | 5.94309025 |
| 203367_at | DUSP14 | 1.13E-05 | 0.00250122 | 3.532939883 | 9.11449438 | 5.5815545 |
| 220253_s_at | LRP12 | 6.85E-05 | 0.00943875 | 3.599922613 | 6.81323964 | 3.21331702 |
| 235371_at | GLT8D4 | 2.17E-08 | 1.69E-05 | 3.459218653 | 10.5430212 | 7.08380255 |
| 209596_at | MXRA5 | 4.06E-06 | 0.00112833 | 3.451505647 | 10.0670331 | 6.61552745 |
| 206025_s_at | TNFAIP6 | 4.62E-05 | 0.00735911 | 3.49128443 | 7.84750142 | 4.35621699 |
| 212464_s_at | FN1 | 5.05E-07 | 0.00020614 | 3.426013785 | 12.5291425 | 9.10312875 |
| 216442_x_at | FN1 | 1.72E-06 | 0.00055153 | 3.426039012 | 12.3247319 | 8.89869286 |
| 240991_at | NA | 4.89E-05 | 0.00755845 | 3.430925412 | 8.93896629 | 5.50804088 |
| 202310_s_at | COL1A1 | 7.77E-08 | 4.72E-05 | 3.403076175 | 12.8282818 | 9.42520561 |
| 229450_at | IFIT3 | 4.40E-15 | 3.44E-11 | 3.38384366 | 11.5605872 | 8.17674355 |
| 203423_at | RBP1 | 8.85E-06 | 0.00206713 | 3.401056115 | 8.95474382 | 5.55368771 |
| 226545_at | CD109 | 1.09E-05 | 0.00241834 | 3.375109572 | 9.46303715 | 6.08792758 |
| 211719_x_at | FN1 | 9.80E-07 | 0.00034939 | 3.350400991 | 12.6252386 | 9.27483762 |
| 204141_at | TUBB2A | 1.06E-06 | 0.00036757 | 3.352270969 | 11.028169 | 7.67589806 |
| 203917_at | CXADR | 3.77E-07 | 0.00017013 | 3.370672195 | 8.97546232 | 5.60479012 |
| 203126_at | IMPA2 | 6.75E-06 | 0.00167756 | 3.34760725 | 9.33277637 | 5.98516912 |
| 217996_at | PHLDA1 | 3.89E-08 | 2.76E-05 | 3.338032844 | 9.58318732 | 6.24515448 |
| 210495_x_at | FN1 | 9.97E-07 | 0.00034939 | 3.317511948 | 12.5123454 | 9.1948335 |
| 1552619_a_at | ANLN | 7.45E-05 | 0.00998451 | 3.395296984 | 7.31449026 | 3.91919327 |
| 213288_at | MBOAT2 | 2.65E-05 | 0.00477349 | 3.326618947 | 9.13954682 | 5.81292788 |
| 213139_at | SNAI2 | 4.77E-06 | 0.00126582 | 3.308983481 | 10.4216122 | 7.11262868 |
| 221729_at | COL5A2 | 4.32E-09 | 4.82E-06 | 3.304222065 | 10.872337 | 7.56811493 |
| 222288_at | NA | 4.82E-20 | 2.64E-15 | 3.296825314 | 9.83259786 | 6.53577255 |
| 204006_s_at | FCGR3B | 1.01E-05 | 0.00229144 | 3.320202672 | 8.17074798 | 4.85054531 |
| 212070_at | GPR56 | 1.65E-05 | 0.00333074 | 3.29148506 | 9.52005704 | 6.22857198 |
| 211538_s_at | HSPA2 | 3.93E-06 | 0.00110405 | 3.267480837 | 9.85565396 | 6.58817312 |
| 230518_at | MPZL2 | 4.84E-05 | 0.00753149 | 3.298326315 | 8.11853858 | 4.82021226 |
| 215177_s_at | ITGA6 | 1.54E-16 | 2.11E-12 | 3.254686487 | 10.5879563 | 7.33326982 |
| 213506_at | F2RL1 | 5.46E-08 | 3.51E-05 | 3.256634367 | 9.52261298 | 6.26597861 |
| 202869_at | OAS1 | 5.45E-06 | 0.0014314 | 3.22402698 | 10.5752987 | 7.35127176 |
| 238755_at | NA | 6.89E-05 | 0.00943898 | 3.284382656 | 6.99809095 | 3.7137083 |
| 212353_at | SULF1 | 1.70E-19 | 4.66E-15 | 3.195932784 | 10.1746779 | 6.97874513 |
| 205552_s_at | OAS1 | 4.10E-08 | 2.87E-05 | 3.183501093 | 10.0572414 | 6.87374029 |
| 225519_at | PPP4R2 | 1.75E-07 | 8.87E-05 | 3.165123388 | 11.1263116 | 7.96118826 |
| 221730_at | COL5A2 | 1.50E-07 | 7.79E-05 | 3.158449302 | 10.3064628 | 7.14801349 |
| 212344_at | SULF1 | 7.10E-06 | 0.00173415 | 3.168841443 | 9.01859395 | 5.84975251 |
| 203083_at | THBS2 | 6.13E-08 | 3.85E-05 | 3.122756312 | 10.4224036 | 7.29964724 |
| 203434_s_at | MME | 7.10E-07 | 0.0002752 | 3.167214511 | 7.48570633 | 4.31849182 |
| 215446_s_at | LOX | 3.97E-07 | 0.00017072 | 3.113483593 | 9.11561324 | 6.00212965 |
| 222646_s_at | ERO1L | 1.20E-08 | 1.04E-05 | 3.09851332 | 10.2714595 | 7.17294616 |
| 212354_at | SULF1 | 2.77E-08 | 2.07E-05 | 3.097918681 | 10.1818738 | 7.08395511 |
| 63009_at | SHQ1 | 1.23E-17 | 2.25E-13 | 3.099491904 | 9.94813025 | 6.84863834 |
| 227998_at | S100A16 | 7.38E-08 | 4.53E-05 | 3.078377014 | 11.2155886 | 8.13721157 |
| 211564_s_at | PDLIM4 | 4.24E-06 | 0.00115835 | 3.099422774 | 8.35402938 | 5.2546066 |
| 218507_at | HIG2 | 4.85E-05 | 0.00753149 | 3.089659881 | 8.5903403 | 5.50068042 |
| 219352_at | HERC6 | 3.36E-08 | 2.48E-05 | 3.051306007 | 9.99800604 | 6.94670003 |
| 201846_s_at | RYBP | 4.11E-15 | 3.44E-11 | 3.03710296 | 11.5744122 | 8.53730923 |
| 228033_at | E2F7 | 1.21E-06 | 0.00040972 | 3.09206575 | 7.25705442 | 4.16498867 |
| 1568611_at | NA | 1.03E-05 | 0.00231062 | 3.032065385 | 7.91256915 | 4.88050376 |
| 227566_at | HNT | 1.04E-07 | 6.10E-05 | 3.002280948 | 8.81086329 | 5.80858234 |
| 219959_at | MOCOS | 1.35E-05 | 0.00281956 | 3.015487912 | 8.11603761 | 5.10054969 |
| 226702_at | CMPK2 | 1.13E-05 | 0.00249536 | 2.987843751 | 10.0231262 | 7.03528242 |
| 225150_s_at | RTKN | 5.93E-06 | 0.0015162 | 3.010504149 | 7.89334233 | 4.88283819 |
| 216615_s_at | HTR3A | 4.48E-05 | 0.00722224 | 3.012595447 | 7.66655264 | 4.65395719 |
| 201844_s_at | RYBP | 9.96E-13 | 4.54E-09 | 2.960201967 | 11.6421303 | 8.68192833 |
| 204051_s_at | SFRP4 | 3.65E-07 | 0.00016639 | 2.963818191 | 10.3165226 | 7.35270444 |
| 202729_s_at | LTBP1 | 6.09E-12 | 1.87E-08 | 2.962970799 | 10.4232089 | 7.46023806 |
| 201667_at | GJA1 | 3.24E-07 | 0.00014887 | 2.953126826 | 12.4022098 | 9.449083 |
| 200862_at | DHCR24 | 9.31E-07 | 0.00034168 | 2.97025369 | 8.96863252 | 5.99837883 |
| 225681_at | CTHRC1 | 2.70E-14 | 1.84E-10 | 2.947696192 | 11.319977 | 8.37228082 |
| 202236_s_at | SLC16A1 | 1.92E-07 | 9.55E-05 | 2.924665466 | 9.21830885 | 6.29364338 |
| 201012_at | ANXA1 | 2.07E-13 | 1.13E-09 | 2.905465639 | 13.0343299 | 10.1288642 |
| 205220_at | GPR109B | 4.31E-07 | 0.00018019 | 2.917348354 | 8.49045366 | 5.57310531 |
| 226317_at | PPP4R2 | 1.00E-13 | 6.10E-10 | 2.875120919 | 11.2550944 | 8.37997349 |
| 229947_at | PI15 | 7.78E-06 | 0.00185736 | 2.881794208 | 7.90111268 | 5.01931848 |
| 212724_at | RND3 | 5.49E-07 | 0.00022065 | 2.845134579 | 10.9556842 | 8.11054965 |
| 211122_s_at | CXCL11 | 3.45E-12 | 1.26E-08 | 2.841436082 | 8.90761919 | 6.0661831 |
| 217771_at | GOLM1 | 4.83E-05 | 0.00753149 | 2.841051272 | 8.08402746 | 5.24297619 |
| 203649_s_at | PLA2G2A | 3.52E-05 | 0.00596032 | 2.801121367 | 9.16666033 | 6.36553897 |
| 200632_s_at | NDRG1 | 2.65E-05 | 0.00477349 | 2.786830774 | 11.4497792 | 8.66294839 |
| 209772_s_at | CD24 | 5.38E-05 | 0.00812985 | 2.786628936 | 9.77268811 | 6.98605918 |
| 39248_at | AQP3 | 8.69E-11 | 1.90E-07 | 2.775724526 | 11.9591656 | 9.18344106 |
| 202990_at | PYGL | 6.06E-07 | 0.00023994 | 2.781469667 | 9.88246439 | 7.10099472 |
| 210715_s_at | SPINT2 | 1.48E-06 | 0.00047898 | 2.778974777 | 9.97523045 | 7.19625567 |
| 203476_at | TPBG | 8.23E-07 | 0.00031042 | 2.771353358 | 10.4546484 | 7.683295 |
| 213793_s_at | HOMER1 | 5.43E-05 | 0.00815361 | 2.784532225 | 8.0039222 | 5.21938997 |
| 202234_s_at | SLC16A1 | 5.12E-07 | 0.00020722 | 2.791818149 | 7.61509471 | 4.82327656 |
| 211597_s_at | HOPX | 1.02E-06 | 0.0003556 | 2.74088021 | 10.6177085 | 7.8768283 |
| 201310_s_at | C5orf13 | 2.52E-05 | 0.00461515 | 2.730867521 | 10.4898273 | 7.7589598 |
| 205016_at | TGFA | 3.38E-06 | 0.00096759 | 2.729815652 | 9.25535352 | 6.52553786 |
| 235651_at | NA | 3.67E-06 | 0.00103834 | 2.726479249 | 8.76650418 | 6.04002493 |
| 201852_x_at | COL3A1 | 3.41E-11 | 8.89E-08 | 2.708006606 | 12.8516292 | 10.1436226 |
| 228846_at | MXD1 | 3.94E-06 | 0.00110405 | 2.717126312 | 8.31223808 | 5.59511176 |
| 201845_s_at | RYBP | 2.40E-12 | 9.36E-09 | 2.69180893 | 11.771585 | 9.07977611 |
| 229802_at | NA | 1.65E-12 | 6.95E-09 | 2.704434844 | 8.56365008 | 5.85921524 |
| 222668_at | KCTD15 | 6.25E-07 | 0.0002459 | 2.713563497 | 8.00172454 | 5.28816104 |
| 209218_at | SQLE | 1.00E-05 | 0.00229144 | 2.690688619 | 9.85456988 | 7.16388126 |
| 227628_at | GPX8 | 2.52E-05 | 0.00461515 | 2.671751078 | 8.31103501 | 5.63928394 |
| 226188_at | HSPC159 | 4.11E-06 | 0.00112833 | 2.650717961 | 9.67865786 | 7.0279399 |
| 203695_s_at | DFNA5 | 4.61E-06 | 0.00123523 | 2.649278833 | 9.21129309 | 6.56201426 |
| 201506_at | TGFBI | 3.66E-11 | 9.10E-08 | 2.61095629 | 12.7430396 | 10.1320833 |
| 226930_at | FNDC1 | 1.54E-05 | 0.00312606 | 2.622349887 | 9.20609069 | 6.5837408 |
| 224802_at | NDFIP2 | 1.99E-06 | 0.0006173 | 2.611709783 | 9.58518659 | 6.97347681 |
| 1555167_s_at | NAMPT | 8.61E-07 | 0.00032197 | 2.591014427 | 8.67698606 | 6.08597163 |
| 210026_s_at | CARD10 | 1.07E-05 | 0.0023896 | 2.575495952 | 8.88187964 | 6.30638369 |
| 206284_x_at | CLTB | 1.97E-05 | 0.00391019 | 2.569698516 | 9.56495384 | 6.99525533 |
| 226777_at | NA | 6.17E-12 | 1.87E-08 | 2.561330603 | 8.93675332 | 6.37542272 |
| 217739_s_at | NAMPT | 6.29E-10 | 8.84E-07 | 2.523317249 | 10.6758566 | 8.15253939 |
| 225060_at | LRP11 | 1.36E-05 | 0.00281956 | 2.51714063 | 9.95714402 | 7.44000339 |
| 224911_s_at | DCBLD2 | 1.43E-05 | 0.00295215 | 2.521987882 | 8.71325654 | 6.19126866 |
| 202068_s_at | LDLR | 1.57E-10 | 3.04E-07 | 2.511458791 | 9.74914602 | 7.23768723 |
| 211043_s_at | CLTB | 4.95E-05 | 0.00760525 | 2.502616901 | 9.97097813 | 7.46836123 |
| 203431_s_at | RICS | 4.52E-05 | 0.00727029 | 2.540359861 | 7.22975849 | 4.68939863 |
| 210519_s_at | NQO1 | 6.26E-06 | 0.00157362 | 2.504064188 | 8.50454336 | 6.00047918 |
| 201242_s_at | ATP1B1 | 5.74E-09 | 5.92E-06 | 2.484507445 | 11.3254564 | 8.84094893 |
| 219691_at | SAMD9 | 4.26E-10 | 6.85E-07 | 2.487993479 | 9.51961902 | 7.03162554 |
| 220658_s_at | ARNTL2 | 4.59E-05 | 0.00735911 | 2.499676244 | 7.90827332 | 5.40859708 |
| 217997_at | PHLDA1 | 4.10E-05 | 0.00666413 | 2.501966674 | 7.77391091 | 5.27194424 |
| 220161_s_at | EPB41L4B | 2.25E-05 | 0.00432169 | 2.464811467 | 8.75714036 | 6.29232889 |
| 227556_at | NME7 | 1.61E-10 | 3.04E-07 | 2.449010431 | 10.2457704 | 7.79675998 |
| 215707_s_at | PRNP | 6.31E-10 | 8.84E-07 | 2.427407 | 9.92183919 | 7.49443219 |
| 208650_s_at | CD24 | 7.05E-06 | 0.00172768 | 2.422274529 | 10.6576544 | 8.23537991 |
| 227034_at | ANKRD57 | 1.02E-05 | 0.00230593 | 2.415422443 | 9.69079956 | 7.27537712 |
| 210749_x_at | DDR1 | 3.98E-05 | 0.00654197 | 2.414648605 | 9.61073374 | 7.19608514 |
| 211161_s_at | COL3A1 | 1.32E-08 | 1.11E-05 | 2.401977591 | 12.3466077 | 9.94463013 |
| 204341_at | TRIM16 | 6.05E-06 | 0.00153124 | 2.414486908 | 8.3964743 | 5.98198739 |
| 204675_at | SRD5A1 | 2.80E-05 | 0.00500403 | 2.412102497 | 8.30276618 | 5.89066368 |
| 204201_s_at | PTPN13 | 2.43E-05 | 0.00449011 | 2.39599125 | 9.07952278 | 6.68353153 |
| 223194_s_at | SLC22A23 | 5.03E-06 | 0.0013284 | 2.395955151 | 8.93017965 | 6.5342245 |
| 201201_at | CSTB | 7.20E-05 | 0.00976827 | 2.370809211 | 12.2687519 | 9.89794268 |
| 202620_s_at | PLOD2 | 9.79E-09 | 9.07E-06 | 2.374965179 | 9.19695076 | 6.82198558 |
| 231766_s_at | COL12A1 | 1.05E-09 | 1.34E-06 | 2.375333754 | 8.83706103 | 6.46172727 |
| 208836_at | ATP1B3 | 9.01E-09 | 8.49E-06 | 2.356297662 | 11.3108495 | 8.95455186 |
| 201005_at | CD9 | 2.05E-05 | 0.00403758 | 2.355351681 | 10.656314 | 8.30096233 |
| 213765_at | MFAP5 | 1.07E-08 | 9.70E-06 | 2.352971127 | 9.39603174 | 7.04306061 |
| 222608_s_at | ANLN | 1.30E-05 | 0.00272774 | 2.360235377 | 8.02941919 | 5.66918382 |
| 201243_s_at | ATP1B1 | 1.06E-05 | 0.00237544 | 2.338689615 | 9.80264943 | 7.46395982 |
| 214734_at | EXPH5 | 4.12E-11 | 9.79E-08 | 2.353668747 | 7.76546297 | 5.41179422 |
| 55081_at | MICALL1 | 1.70E-05 | 0.00341405 | 2.334882248 | 9.44128277 | 7.10640052 |
| 218400_at | OAS3 | 1.44E-05 | 0.00295215 | 2.328806673 | 9.55239637 | 7.2235897 |
| 222847_s_at | EGLN3 | 6.58E-05 | 0.00926847 | 2.340072184 | 8.10917998 | 5.76910779 |
| 1554452_a_at | HIG2 | 6.33E-05 | 0.00903453 | 2.334957039 | 8.39095601 | 6.05599897 |
| 202086_at | MX1 | 1.20E-05 | 0.00259083 | 2.313394283 | 11.0699013 | 8.75650697 |
| 218986_s_at | DDX60 | 4.54E-09 | 4.96E-06 | 2.303349608 | 10.5889532 | 8.28560363 |
| 201983_s_at | EGFR | 2.08E-08 | 1.65E-05 | 2.302282185 | 10.4486156 | 8.1463334 |
| 204992_s_at | PFN2 | 1.23E-08 | 1.05E-05 | 2.299163063 | 10.0020931 | 7.70293005 |
| 209146_at | SC4MOL | 1.08E-08 | 9.70E-06 | 2.297702233 | 10.1539601 | 7.85625785 |
| 213905_x_at | BGN | 6.35E-05 | 0.00903453 | 2.295527984 | 10.2268472 | 7.93131921 |
| 206685_at | HCG4 | 5.16E-05 | 0.00788014 | 2.360875815 | 6.22619861 | 3.86532279 |
| 202998_s_at | LOXL2 | 1.45E-05 | 0.00296107 | 2.289558844 | 9.13040832 | 6.84084947 |
| 217738_at | NAMPT | 5.23E-08 | 3.41E-05 | 2.281578323 | 10.0395255 | 7.75794717 |
| 226885_at | NA | 8.79E-06 | 0.00206713 | 2.286599793 | 8.65758482 | 6.37098503 |
| 201563_at | SORD | 5.89E-10 | 8.70E-07 | 2.283762417 | 8.98034828 | 6.69658586 |
| 204517_at | PPIC | 4.94E-08 | 3.29E-05 | 2.274736486 | 10.0498407 | 7.77510419 |
| 201841_s_at | HSPB1 | 5.61E-08 | 3.57E-05 | 2.267840424 | 12.3922123 | 10.1243719 |
| 215813_s_at | PTGS1 | 4.63E-05 | 0.00735911 | 2.276146825 | 8.63257479 | 6.35642797 |
| 242881_x_at | NA | 1.52E-11 | 4.16E-08 | 2.285496385 | 7.885583 | 5.60008662 |
| 222433_at | ENAH | 2.40E-05 | 0.00449011 | 2.255689896 | 9.9364723 | 7.6807824 |
| 218888_s_at | NETO2 | 4.66E-05 | 0.00738565 | 2.264147759 | 7.94559951 | 5.68145175 |
| 212190_at | SERPINE2 | 1.78E-08 | 1.43E-05 | 2.243072133 | 10.6984664 | 8.45539429 |
| 201185_at | HTRA1 | 2.38E-09 | 2.77E-06 | 2.219492637 | 11.0036267 | 8.7841341 |
| 215076_s_at | COL3A1 | 1.06E-07 | 6.17E-05 | 2.210579672 | 13.2662034 | 11.0556237 |
| 230175_s_at | DCBLD2 | 1.11E-10 | 2.33E-07 | 2.222710181 | 7.99886879 | 5.77615861 |
| 202403_s_at | COL1A2 | 1.39E-07 | 7.47E-05 | 2.197559387 | 12.9442368 | 10.7466774 |
| 201849_at | BNIP3 | 1.45E-08 | 1.19E-05 | 2.185306219 | 10.1095345 | 7.9242283 |
| 204533_at | CXCL10 | 4.85E-08 | 3.28E-05 | 2.182538205 | 10.4927973 | 8.31025914 |
| 224999_at | NA | 1.27E-07 | 7.06E-05 | 2.168214116 | 10.1398929 | 7.97167881 |
| 202071_at | SDC4 | 1.46E-08 | 1.19E-05 | 2.165111901 | 9.58398409 | 7.41887219 |
| 202193_at | LIMK2 | 6.28E-05 | 0.00902987 | 2.146503211 | 9.2095843 | 7.06308109 |
| 218002_s_at | CXCL14 | 6.19E-09 | 6.27E-06 | 2.136843544 | 12.0993721 | 9.9625286 |
| 204059_s_at | ME1 | 1.09E-07 | 6.19E-05 | 2.135999366 | 9.71715882 | 7.58115946 |
| 202887_s_at | DDIT4 | 2.55E-07 | 0.00012131 | 2.129811438 | 11.7428599 | 9.61304846 |
| 208898_at | ATP6V1D | 4.67E-09 | 5.01E-06 | 2.119453435 | 9.87204912 | 7.75259569 |
| 201141_at | GPNMB | 4.30E-07 | 0.00018019 | 2.110615764 | 11.9828661 | 9.87225038 |
| 225018_at | SPIRE1 | 5.78E-10 | 8.70E-07 | 2.133318184 | 7.59845662 | 5.46513843 |
| 208937_s_at | ID1 | 1.82E-07 | 9.14E-05 | 2.113223062 | 9.8281611 | 7.71493804 |
| 201798_s_at | FER1L3 | 7.68E-09 | 7.37E-06 | 2.096088756 | 10.4438322 | 8.34774341 |
| 226177_at | GLTP | 1.60E-07 | 8.25E-05 | 2.094218346 | 10.7213287 | 8.62711035 |
| 236172_at | LTB4R | 3.35E-10 | 5.54E-07 | 2.085676279 | 7.69660469 | 5.61092841 |
| 227309_at | YOD1 | 6.76E-05 | 0.00940579 | 2.067867736 | 9.14609469 | 7.07822696 |
| 225664_at | COL12A1 | 4.06E-07 | 0.00017354 | 2.061445301 | 10.7804013 | 8.718956 |
| 202263_at | CYB5R1 | 4.95E-07 | 0.00020348 | 2.047325988 | 10.2064453 | 8.15911932 |
| 205943_at | TDO2 | 1.38E-07 | 7.47E-05 | 2.052406149 | 8.20967379 | 6.15726764 |
| 226517_at | BCAT1 | 1.08E-07 | 6.19E-05 | 2.027881951 | 8.59129408 | 6.56341213 |
| 209911_x_at | HIST1H2BD | 1.19E-08 | 1.04E-05 | 2.013513563 | 8.74178837 | 6.7282748 |
| 224435_at | C10orf58 | 9.72E-07 | 0.00034939 | 1.990655469 | 10.6082177 | 8.61756225 |
| 201590_x_at | ANXA2 | 2.23E-07 | 0.00010771 | 1.981305247 | 12.6213908 | 10.6400856 |
| 212295_s_at | SLC7A1 | 1.53E-06 | 0.00049207 | 1.983478178 | 10.4637328 | 8.48025459 |
| 200872_at | S100A10 | 3.90E-07 | 0.00017048 | 1.977009121 | 12.4855405 | 10.5085314 |
| 227388_at | TUSC1 | 6.94E-08 | 4.31E-05 | 1.977666493 | 9.6091383 | 7.63147181 |
| 225283_at | ARRDC4 | 1.23E-06 | 0.00041116 | 1.967015114 | 9.73442 | 7.76740489 |
| 214453_s_at | IFI44 | 1.14E-06 | 0.00038868 | 1.959696764 | 9.78185756 | 7.8221608 |
| 201069_at | MMP2 | 2.53E-06 | 0.00076524 | 1.927838787 | 10.3606717 | 8.43283289 |
| 203987_at | FZD6 | 1.98E-07 | 9.74E-05 | 1.927847471 | 9.01271846 | 7.08487099 |
| 1556499_s_at | COL1A1 | 3.36E-06 | 0.00096759 | 1.913782415 | 12.9454199 | 11.0316375 |
| 210427_x_at | ANXA2 | 3.82E-07 | 0.00017048 | 1.908601613 | 12.5425204 | 10.6339187 |
| 200999_s_at | CKAP4 | 2.66E-06 | 0.00079437 | 1.907493649 | 9.8590158 | 7.95152215 |
| 224097_s_at | F11R | 2.25E-07 | 0.00010797 | 1.911284996 | 7.98132808 | 6.07004309 |
| 201468_s_at | NQO1 | 1.22E-06 | 0.00041116 | 1.90534792 | 8.32661131 | 6.42126339 |
| 213476_x_at | TUBB3 | 1.74E-06 | 0.00055386 | 1.895690906 | 10.3156348 | 8.41994384 |
| 208864_s_at | TXN | 4.60E-06 | 0.00123523 | 1.891424832 | 11.2940159 | 9.40259105 |
| 205241_at | SCO2 | 4.27E-06 | 0.00116082 | 1.891631081 | 9.71479984 | 7.82316876 |
| 213503_x_at | ANXA2 | 3.93E-07 | 0.00017048 | 1.881216571 | 12.5489332 | 10.6677166 |
| 1555756_a_at | CLEC7A | 2.13E-06 | 0.00065378 | 1.897381924 | 7.56493945 | 5.66755753 |
| 203413_at | NELL2 | 6.52E-07 | 0.00025479 | 1.877421656 | 10.8665073 | 8.98908561 |
| 203939_at | NT5E | 1.94E-06 | 0.00060533 | 1.87602682 | 9.01244111 | 7.13641429 |
| 266_s_at | CD24 | 7.31E-06 | 0.00177731 | 1.8480632 | 11.1277005 | 9.27963725 |
| 31874_at | GAS2L1 | 5.92E-07 | 0.00023621 | 1.842948477 | 8.41096912 | 6.56802064 |
| 209154_at | TAX1BP3 | 5.78E-06 | 0.00148297 | 1.829085825 | 11.396332 | 9.56724621 |
| 207121_s_at | MAPK6 | 5.60E-06 | 0.00145074 | 1.826872236 | 10.0389855 | 8.21211331 |
| 204439_at | IFI44L | 6.89E-06 | 0.00169692 | 1.826760838 | 9.94106966 | 8.11430882 |
| 201666_at | TIMP1 | 8.91E-06 | 0.00207235 | 1.816395233 | 11.9466658 | 10.1302706 |
| 218718_at | PDGFC | 9.74E-06 | 0.0022471 | 1.816017679 | 10.4342308 | 8.61821312 |
| 230422_at | FPR3 | 4.68E-06 | 0.00124785 | 1.82271466 | 7.98940088 | 6.16668622 |
| 225314_at | OCIAD2 | 7.43E-07 | 0.00028462 | 1.811045037 | 9.8602817 | 8.04923666 |
| 220187_at | STEAP4 | 1.02E-07 | 6.09E-05 | 1.82664435 | 7.37341392 | 5.54676957 |
| 201505_at | LAMB1 | 8.76E-06 | 0.00206713 | 1.803373576 | 10.0450545 | 8.24168091 |
| 231879_at | COL12A1 | 7.52E-06 | 0.0018032 | 1.807666033 | 8.41594115 | 6.60827512 |
| 200832_s_at | SCD | 1.00E-05 | 0.00229144 | 1.796785747 | 10.7253069 | 8.92852115 |
| 216379_x_at | NA | 1.18E-05 | 0.00255627 | 1.792777741 | 12.3128558 | 10.5200781 |
| 201695_s_at | NP | 5.99E-06 | 0.00152407 | 1.787222112 | 9.88093607 | 8.09371396 |
| 211240_x_at | CTNND1 | 2.85E-07 | 0.00013415 | 1.78766275 | 9.4990965 | 7.71143375 |
| 202949_s_at | FHL2 | 1.12E-06 | 0.00038345 | 1.780315568 | 9.82097401 | 8.04065844 |
| 219215_s_at | SLC39A4 | 4.64E-07 | 0.00019211 | 1.783131088 | 7.46084389 | 5.67771281 |
| 200650_s_at | LDHA | 1.27E-05 | 0.0026745 | 1.760029597 | 12.5793228 | 10.8192932 |
| 201594_s_at | PPP4R1 | 1.49E-05 | 0.00303274 | 1.751054902 | 10.0845339 | 8.33347899 |
| 229152_at | C4orf7 | 1.81E-05 | 0.00361503 | 1.745918619 | 11.8421465 | 10.0962279 |
| 212983_at | HRAS | 4.40E-06 | 0.0011897 | 1.731259269 | 8.54649578 | 6.81523651 |
| 219956_at | GALNT6 | 2.53E-06 | 0.00076524 | 1.730449567 | 8.37993946 | 6.6494899 |
| 226560_at | NA | 8.84E-06 | 0.00206713 | 1.729980888 | 8.22508916 | 6.49510827 |
| 203344_s_at | RBBP8 | 1.59E-05 | 0.00321323 | 1.723207815 | 9.51971735 | 7.79650953 |
| 212013_at | PXDN | 8.15E-07 | 0.00030937 | 1.726508648 | 7.97685556 | 6.25034691 |
| 223220_s_at | PARP9 | 2.30E-05 | 0.00437853 | 1.703222575 | 9.99415079 | 8.29092822 |
| 200737_at | PGK1 | 1.79E-06 | 0.00056239 | 1.701514138 | 9.99084537 | 8.28933123 |
| 209946_at | VEGFC | 1.24E-05 | 0.00265527 | 1.690621957 | 9.1156459 | 7.42502394 |
| 200629_at | WARS | 3.40E-05 | 0.00578161 | 1.685591419 | 10.7623031 | 9.07671172 |
| 207076_s_at | ASS1 | 2.95E-05 | 0.00517843 | 1.686526341 | 9.42022312 | 7.73369678 |
| 203323_at | CAV2 | 3.36E-05 | 0.00574835 | 1.682269993 | 10.3029342 | 8.62066418 |
| 209771_x_at | NA | 3.15E-05 | 0.00545428 | 1.67827388 | 12.3020117 | 10.6237378 |
| 225799_at | LOC541471 | 5.59E-06 | 0.00145074 | 1.680604959 | 9.2983948 | 7.61778985 |
| 217294_s_at | ENO1 | 3.93E-05 | 0.00649208 | 1.675812251 | 11.696458 | 10.0206457 |
| 226752_at | FAM174A | 3.14E-06 | 0.00091692 | 1.672055705 | 8.80032964 | 7.12827394 |
| 212012_at | PXDN | 1.78E-06 | 0.00056239 | 1.659250252 | 9.2178234 | 7.55857315 |
| 218009_s_at | PRC1 | 2.01E-05 | 0.00396286 | 1.653992137 | 8.29923554 | 6.6452434 |
| 217975_at | WBP5 | 3.59E-05 | 0.00605288 | 1.63536273 | 9.88307096 | 8.24770823 |
| 201325_s_at | EMP1 | 1.23E-05 | 0.00264796 | 1.63673185 | 9.10777896 | 7.47104711 |
| 202540_s_at | HMGCR | 4.20E-05 | 0.00679688 | 1.638224661 | 8.61133095 | 6.97310629 |
| 226421_at | AMMECR1 | 3.31E-05 | 0.00570401 | 1.639470017 | 8.2956894 | 6.65621938 |
| 227204_at | PARD6G | 1.25E-05 | 0.00265527 | 1.633074041 | 8.84617465 | 7.21310061 |
| 208977_x_at | TUBB2C | 5.20E-05 | 0.00791661 | 1.620550642 | 10.4841592 | 8.86360855 |
| 212460_at | C14orf147 | 5.40E-05 | 0.00812991 | 1.621939733 | 9.14075908 | 7.51881935 |
| 200641_s_at | YWHAZ | 5.97E-05 | 0.00866189 | 1.617296396 | 11.1181385 | 9.50084215 |
| 212977_at | CXCR7 | 4.72E-05 | 0.00744041 | 1.61911146 | 9.78764985 | 8.16853839 |
| 224937_at | PTGFRN | 5.92E-05 | 0.00861945 | 1.618635973 | 8.99816607 | 7.3795301 |
| 201739_at | SGK1 | 5.65E-05 | 0.00838818 | 1.614022646 | 10.9778366 | 9.36381399 |
| 223193_x_at | FAM162A | 5.68E-05 | 0.00840971 | 1.613198987 | 10.6353143 | 9.02211536 |
| 200665_s_at | SPARC | 5.90E-05 | 0.00861945 | 1.611174708 | 11.9204672 | 10.3092925 |
| 222484_s_at | CXCL14 | 6.18E-05 | 0.00890813 | 1.609254141 | 12.3883943 | 10.7791401 |
| 201577_at | NME1 | 5.46E-05 | 0.00815554 | 1.600708861 | 9.48961812 | 7.88890926 |
| 213726_x_at | TUBB2C | 2.18E-05 | 0.00422827 | 1.598819927 | 10.2887083 | 8.68988837 |
| 224847_at | CDK6 | 6.90E-05 | 0.00943898 | 1.591682424 | 9.56417809 | 7.97249567 |
| 214168_s_at | TJP1 | 2.68E-06 | 0.00079756 | 1.598318525 | 7.66895098 | 6.07063246 |
| 201952_at | ALCAM | 5.93E-05 | 0.00861945 | 1.586122584 | 10.1713948 | 8.58527226 |
| 214247_s_at | DKK3 | 6.80E-05 | 0.00943131 | 1.583838873 | 10.0305042 | 8.44666536 |
| 230424_at | C5orf13 | 4.07E-06 | 0.00112833 | 1.588631211 | 7.54695584 | 5.95832463 |
| 222750_s_at | SRD5A3 | 2.10E-05 | 0.0041206 | 1.586248948 | 7.62849509 | 6.04224615 |
| 210087_s_at | MPZL1 | 2.12E-05 | 0.00413566 | 1.581150722 | 7.82559052 | 6.2444398 |
| 215071_s_at | HIST1H2AC | 6.44E-05 | 0.00911891 | 1.573837668 | 8.92749906 | 7.35366139 |
| 219764_at | FZD10 | 1.81E-05 | 0.0036195 | 1.579204641 | 7.82682687 | 6.24762223 |
| 223062_s_at | PSAT1 | 5.78E-05 | 0.008514 | 1.572274397 | 8.34831598 | 6.77604158 |
| 236297_at | NA | 7.33E-05 | 0.00985342 | 1.580003997 | 7.22751916 | 5.64751517 |
| 226075_at | SPSB1 | 7.52E-06 | 0.0018032 | 1.574068689 | 7.40681152 | 5.83274283 |
| 1554600_s_at | LMNA | 2.42E-05 | 0.00449011 | 1.547365987 | 9.16195442 | 7.61458843 |
| 210559_s_at | CDC2 | 8.07E-06 | 0.00191805 | 1.536301977 | 8.11625297 | 6.57995099 |
| 239443_at | PCDHB6 | 3.32E-05 | 0.00570401 | 1.558367216 | 6.30770019 | 4.74933297 |
| 226003_at | KIF21A | 6.76E-05 | 0.00940579 | 1.529579579 | 7.93562718 | 6.4060476 |
| 204068_at | STK3 | 2.37E-05 | 0.00448522 | 1.525078571 | 8.58806539 | 7.06298682 |
| 222981_s_at | RAB10 | 2.90E-05 | 0.00514054 | 1.515793637 | 10.0211621 | 8.50536847 |
| 211681_s_at | PDLIM5 | 6.92E-05 | 0.00943898 | 1.522447302 | 7.3949356 | 5.8724883 |
| 201105_at | LGALS1 | 4.86E-05 | 0.00753149 | 1.501084942 | 10.6436523 | 9.14256732 |
| 61734_at | RCN3 | 1.15E-05 | 0.00251864 | 1.494012385 | 7.77859344 | 6.28458105 |
| 228152_s_at | DDX60L | 7.03E-05 | 0.00956078 | 1.485803402 | 9.33183488 | 7.84603148 |
| 212325_at | LIMCH1 | 2.97E-05 | 0.00518093 | 1.469902072 | 7.82868602 | 6.35878394 |
| 227072_at | RTTN | 2.92E-05 | 0.00514411 | 1.452678041 | 8.53582809 | 7.08315005 |
| 228378_at | C12orf29 | 2.32E-05 | 0.00440984 | 1.458567605 | 7.59585293 | 6.13728532 |
| 224995_at | SPIRE1 | 4.02E-05 | 0.00657913 | 1.439529916 | 8.20289247 | 6.76336255 |
| 226600_at | TMTC3 | 6.85E-05 | 0.00943875 | 1.425510878 | 7.83435475 | 6.40884387 |
| 206683_at | ZNF165 | 4.63E-05 | 0.00735911 | 1.44536674 | 6.19787224 | 4.7525055 |
| 223078_s_at | TMOD3 | 3.89E-05 | 0.00644416 | 1.399170788 | 7.13410677 | 5.73493598 |
| 205990_s_at | WNT5A | 7.33E-05 | 0.00985342 | 1.378178241 | 8.27244078 | 6.89426254 |
| 243367_at | NA | 2.99E-05 | 0.00521365 | 1.397564323 | 6.18835396 | 4.79078964 |
| 223611_s_at | LNX1 | 5.44E-05 | 0.00815361 | 1.327451438 | 6.82688322 | 5.49943178 |

Abbreviations: *P* - p value; c*P* - Benjamini-Hochberg corrected p value; FC - fold change of gene expression ratio in LN(+) and LN(-) sample; Exp. - expression level.
