# Supplementary material for: Estimation of groin recurrence risk in patients with squamous cell vulvar carcinoma by the assessment of marker gene expression in the lymph nodes
Source: BMC Cancer. 2012 Jun 6;12:223. doi: 10.1186/1471-2407-12-223 (PMC3414830; doi:10.1186/1471-2407-12-223)
Supplement: Additional file 2 — Table S3. Genes differentially expressed in the four LN(+) and LN(−) sample pairs obtained from VC patients No 15, 21, 46 and 61. Abbreviations: P - p value; NA - not applicable. [file 1471-2407-12-223-S3.doc]

**Supplementary Table 3.** Genes differentially expressed in the four LN(+) and LN(-) sample pairs obtained from VC patients No. 15, 21, 46 and 61.

| **Probe Set** | **Gene Symbol** | **Gene Description** | **15LN(+) *vs*** | **21LN(+) *vs*** | **46LN(+) *vs*** | **61LN(+)** |
| --- | --- | --- | --- | --- | --- | --- |
|  |  |  | **15LN(-)** | **21LN(-)** | **46LN(-)** | **vs 61LN(-)** |
| 222288_at | NA | NA | 4.98E-60 | 0.835232341 | 7.30E-10 | 1 |
| 209875_s_at | SPP1 | secreted phosphoprotein 1 (osteopontin, bone sialoprotein I, early T-lymphocyte activation 1) | 4.51E-52 | 1 | 6.40E-15 | 1 |
| 63009_at | SHQ1 | SHQ1 homolog (S. cerevisiae) | 4.63E-51 | 1 | 0.811845368 | 0.757924823 |
| 235371_at | PPP4R2 | protein phosphatase 4, regulatory subunit 2 | 4.04E-44 | 0.835232341 | 0.000241068 | 1 |
| 226317_at | PPP4R2 | protein phosphatase 4, regulatory subunit 2 | 2.47E-41 | 0.835232341 | 0.821376255 | 0.757924823 |
| 201012_at | ANXA1 | annexin A1 | 2.55E-41 | 1 | 0.087354305 | 1 |
| 225681_at | CTHRC1 | collagen triple helix repeat containing 1 | 6.68E-41 | 1 | 6.46E-20 | 1 |
| 203560_at | GGH | gamma-glutamyl hydrolase (conjugase, folylpolygammaglutamyl hydrolase) | 3.29E-39 | 1 | 0.010501099 | 1 |
| 225519_at | PPP4R2 | protein phosphatase 4, regulatory subunit 2 | 3.63E-39 | 1 | 0.76149608 | 0.976502423 |
| 226237_at | COL8A1 | collagen, type VIII, alpha 1 | 5.09E-39 | 1 | 7.03E-16 | 0.985885685 |
| 213765_at | MFAP5 | microfibrillar associated protein 5 | 1.03E-38 | 0.575914419 | 0.120237233 | 1 |
| 210809_s_at | POSTN | periostin, osteoblast specific factor | 1.12E-38 | 1 | 6.18E-18 | 1 |
| 209758_s_at | MFAP5 | microfibrillar associated protein 5 | 5.50E-37 | 1 | 0.00955441 | 1 |
| 219691_at | SAMD9 | sterile alpha motif domain containing 9 | 1.78E-36 | 1 | 0.76149608 | 1 |
| 221729_at | COL5A2 | collagen, type V, alpha 2 | 2.09E-32 | 1 | 5.94E-20 | 1 |
| 205552_s_at | OAS1 | 2',5'-oligoadenylate synthetase 1, 40/46kDa | 2.31E-32 | 1 | 0.317672151 | 1 |
| 39248_at | AQP3 | aquaporin 3 (Gill blood group) | 5.97E-31 | 1 | 0.003748975 | 1 |
| 221731_x_at | VCAN | versican | 1.25E-29 | 1 | 1.29E-18 | 1 |
| 212190_at | SERPINE2 | serpin peptidase inhibitor, clade E (nexin, plasminogen activator inhibitor type 1), member 2 | 1.57E-29 | 1 | 3.24E-10 | 1 |
| 202620_s_at | PLOD2 | procollagen-lysine, 2-oxoglutarate 5-dioxygenase 2 | 1.57E-29 | 1 | 8.98E-23 | 1 |
| 229802_at | NA | NA | 1.70E-29 | 1 | 6.04E-08 | 1 |
| 203153_at | IFIT1 | interferon-induced protein with tetratricopeptide repeats 1 | 3.37E-29 | 1 | 0.052824274 | 1 |
| 201506_at | TGFBI | transforming growth factor, beta-induced, 68kDa | 5.52E-29 | 1 | 7.78E-17 | 1 |
| 201667_at | GJA1 | gap junction protein, alpha 1, 43kDa | 7.90E-29 | 1 | 0.000301734 | 1 |
| 217739_s_at | PBEF1 | pre-B-cell colony enhancing factor 1 | 1.48E-28 | 1 | 0.007884266 | 1 |
| 219352_at | HERC6 | hect domain and RLD 6 | 2.22E-28 | 1 | 0.250458367 | 1 |
| 205483_s_at | ISG15 | ISG15 ubiquitin-like modifier | 4.47E-28 | 1 | 6.47E-11 | 1 |
| 212353_at | SULF1 | sulfatase 1 | 5.99E-28 | 1 | 1.74E-21 | 1 |
| 215707_s_at | PRNP | prion protein (p27-30) (Creutzfeldt-Jakob disease, Gerstmann-Strausler-Scheinker syndrome, fatal familial insomnia) | 8.29E-28 | 1 | 0.418778332 | 1 |
| 227566_at | HNT | neurotrimin | 1.52E-27 | 1 | 2.61E-09 | 1 |
| 1555167_s_at | PBEF1 | pre-B-cell colony enhancing factor 1 | 1.66E-27 | 1 | 0.76149608 | 1 |
| 230746_s_at | STC1 | stanniocalcin 1 | 1.69E-27 | 1 | 0.002576007 | 1 |
| 218986_s_at | FLJ20035 | hypothetical protein FLJ20035 | 2.42E-27 | 1 | 0.032738804 | 1 |
| 202068_s_at | LDLR | low density lipoprotein receptor (familial hypercholesterolemia) | 7.72E-27 | 1 | 0.017064491 | 1 |
| 212354_at | SULF1 | sulfatase 1 | 3.00E-26 | 1 | 1.29E-18 | 1 |
| 202990_at | PYGL | phosphorylase, glycogen; liver (Hers disease, glycogen storage disease type VI) | 2.24E-25 | 1 | 0.003888935 | 1 |
| 215446_s_at | LOX | lysyl oxidase | 3.64E-25 | 1 | 1.54E-09 | 1 |
| 236297_at | NA | NA | 1.04E-24 | 1 | 0.046290975 | 1 |
| 202086_at | MX1 | myxovirus (influenza virus) resistance 1, interferon-inducible protein p78 (mouse) | 1.39E-24 | 1 | 0.335984306 | 1 |
| 211122_s_at | CXCL11 | chemokine (C-X-C motif) ligand 11 | 4.78E-24 | 1 | 0.003959734 | 1 |
| 202437_s_at | CYP1B1 | cytochrome P450, family 1, subfamily B, polypeptide 1 | 7.09E-24 | 0.001611763 | 2.53E-11 | 1 |
| 224999_at | EGFR | epidermal growth factor receptor (erythroblastic leukemia viral (v-erb-b) oncogene homolog, avian) | 7.49E-24 | 1 | 0.000199456 | 1 |
| 223122_s_at | SFRP2 | secreted frizzled-related protein 2 | 8.08E-24 | 1 | 1.17E-06 | 1 |
| 217294_s_at | ENO1 | enolase 1, (alpha) | 9.14E-24 | 1 | 0.000335182 | 1 |
| 219410_at | TMEM45A | transmembrane protein 45A | 3.70E-23 | 1 | 6.36E-06 | 1 |
| 202345_s_at | FABP5 | fatty acid binding protein 5 (psoriasis-associated) | 3.99E-23 | 1 | 0.000621656 | 1 |
| 208650_s_at | CD24 | CD24 molecule | 4.47E-23 | 1 | 0.295307923 | 1 |
| 228708_at | GPER | G protein-coupled estrogen receptor 1 | 6.35E-23 | 1 | 0.116128623 | 1 |
| 201185_at | HTRA1 | HtrA serine peptidase 1 | 1.35E-22 | 1 | 2.18E-06 | 1 |
| 226777_at | NA | NA | 1.47E-22 | 1 | 1.03E-19 | 1 |
| 214453_s_at | IFI44 | interferon-induced protein 44 | 1.58E-22 | 1 | 0.067351726 | 1 |
| 227998_at | S100A16 | S100 calcium binding protein A16 | 3.64E-22 | 1 | 0.000278448 | 1 |
| 225060_at | LRP11 | low density lipoprotein receptor-related protein 11 | 4.83E-22 | 1 | 0.137956998 | 1 |
| 226885_at | NA | NA | 6.57E-22 | 1 | 0.011364688 | 1 |
| 203789_s_at | SEMA3C | sema domain, immunoglobulin domain (Ig), short basic domain, secreted, (semaphorin) 3C | 7.40E-22 | 1 | 0.000331509 | 1 |
| 231879_at | COL12A1 | collagen, type XII, alpha 1 | 7.64E-22 | 1 | 1.49E-07 | 1 |
| 224802_at | NDFIP2 | Nedd4 family interacting protein 2 | 9.86E-22 | 1 | 0.133693382 | 1 |
| 225342_at | AK3L1 | adenylate kinase 3-like 1 | 1.27E-21 | 1 | 0.76149608 | 1 |
| 242625_at | RSAD2 | radical S-adenosyl methionine domain containing 2 | 1.45E-21 | 1 | 0.006476586 | 1 |
| 208836_at | ATP1B3 | ATPase, Na+/K+ transporting, beta 3 polypeptide | 2.37E-21 | 1 | 8.22E-07 | 1 |
| 227309_at | YOD1 | YOD1 OTU deubiquinating enzyme 1 homolog (S. cerevisiae) | 5.40E-21 | 1 | 0.76149608 | 1 |
| 202404_s_at | COL1A2 | collagen, type I, alpha 2 | 1.05E-20 | 1 | 9.09E-11 | 1 |
| 203476_at | TPBG | trophoblast glycoprotein | 1.29E-20 | 1 | 0.002389664 | 1 |
| 210512_s_at | VEGFA | vascular endothelial growth factor A | 1.32E-20 | 1 | 0.76149608 | 0.954043109 |
| 201563_at | SORD | sorbitol dehydrogenase | 1.85E-20 | 1 | 0.76149608 | 1 |
| 210495_x_at | FN1 | fibronectin 1 | 3.47E-20 | 1 | 2.40E-10 | 1 |
| 227556_at | NME7 | non-metastatic cells 7, protein expressed in (nucleoside-diphosphate kinase) | 4.82E-20 | 0.94943249 | 5.94E-05 | 0.816275799 |
| 227461_at | STON2 | stonin 2 | 8.60E-20 | 1 | 4.04E-05 | 1 |
| 202540_s_at | HMGCR | 3-hydroxy-3-methylglutaryl-Coenzyme A reductase | 1.20E-19 | 1 | 0.554519999 | 1 |
| 211719_x_at | FN1 | fibronectin 1 | 1.33E-19 | 1 | 1.05E-10 | 1 |
| 202310_s_at | COL1A1 | collagen, type I, alpha 1 | 1.47E-19 | 1 | 2.37E-12 | 1 |
| 202071_at | SDC4 | syndecan 4 | 2.18E-19 | 1 | 2.22E-11 | 1 |
| 212724_at | RND3 | Rho family GTPase 3 | 2.33E-19 | 1 | 0.012182795 | 1 |
| 55081_at | MICALL1 | MICAL-like 1 | 3.22E-19 | 1 | 0.209544587 | 1 |
| 242881_x_at | NA | NA | 3.74E-19 | 1 | 0.018605656 | 0.757924823 |
| 204051_s_at | SFRP4 | secreted frizzled-related protein 4 | 4.07E-19 | 0.835232341 | 1.14E-09 | 1 |
| 221730_at | COL5A2 | collagen, type V, alpha 2 | 4.15E-19 | 1 | 1.54E-09 | 1 |
| 216442_x_at | FN1 | fibronectin 1 | 4.28E-19 | 1 | 7.04E-11 | 1 |
| 235651_at | NA | NA | 7.81E-19 | 1 | 0.76149608 | 1 |
| 217996_at | PHLDA1 | pleckstrin homology-like domain, family A, member 1 | 9.66E-19 | 1 | 0.001158569 | 1 |
| 210026_s_at | CARD10 | caspase recruitment domain family, member 10 | 1.07E-18 | 1 | 0.76149608 | 1 |
| 202193_at | LIMK2 | LIM domain kinase 2 | 1.56E-18 | 1 | 0.055600384 | 1 |
| 204992_s_at | PFN2 | profilin 2 | 2.04E-18 | 1 | 5.74E-14 | 0.929311513 |
| 201310_s_at | C5orf13 | chromosome 5 open reading frame 13 | 2.21E-18 | 1 | 0.31872895 | 1 |
| 202411_at | IFI27 | interferon, alpha-inducible protein 27 | 4.39E-18 | 1 | 3.52E-08 | 1 |
| 224801_at | NDFIP2 | Nedd4 family interacting protein 2 | 5.26E-18 | 1 | 0.76149608 | 1 |
| 201798_s_at | FER1L3 | fer-1-like 3, myoferlin (C. elegans) | 1.13E-17 | 1 | 0.000245491 | 1 |
| 226702_at | LOC129607 | hypothetical protein LOC129607 | 1.58E-17 | 1 | 0.29483592 | 1 |
| 203434_s_at | MME | membrane metallo-endopeptidase | 1.64E-17 | 1 | 0.196999615 | 1 |
| 213506_at | F2RL1 | coagulation factor II (thrombin) receptor-like 1 | 2.04E-17 | 1 | 4.17E-05 | 1 |
| 236313_at | CDKN2B | cyclin-dependent kinase inhibitor 2B (p15, inhibits CDK4) | 2.50E-17 | 1 | 0.054193035 | 1 |
| 225803_at | FBXO32 | F-box protein 32 | 2.80E-17 | 1 | 0.02404267 | 0.757924823 |
| 222433_at | ENAH | enabled homolog (Drosophila) | 2.97E-17 | 1 | 4.54E-05 | 1 |
| 230175_s_at | NA | NA | 3.14E-17 | 1 | 0.76149608 | 0.869104195 |
| 213287_s_at | KRT10 | keratin 10 (epidermolytic hyperkeratosis; keratosis palmaris et plantaris) | 3.36E-17 | 1 | 0.76149608 | 1 |
| 200872_at | S100A10 | S100 calcium binding protein A10 | 3.52E-17 | 1 | 0.002571409 | 1 |
| 225283_at | ARRDC4 | arrestin domain containing 4 | 5.32E-17 | 1 | 0.099059447 | 0.945164946 |
| 201695_s_at | NP | nucleoside phosphorylase | 6.32E-17 | 1 | 0.24029319 | 1 |
| 210519_s_at | NQO1 | NAD(P)H dehydrogenase, quinone 1 | 8.86E-17 | 1 | 0.76149608 | 1 |
| 230183_at | NA | NA | 1.23E-16 | 1 | 0.76149608 | 1 |
| 224911_s_at | DCBLD2 | discoidin, CUB and LCCL domain containing 2 | 1.48E-16 | 1 | 2.94E-08 | 1 |
| 204400_at | EFS | embryonal Fyn-associated substrate | 1.83E-16 | 1 | 0.64071832 | 1 |
| 202234_s_at | SLC16A1 | solute carrier family 16, member 1 (monocarboxylic acid transporter 1) | 1.83E-16 | 1 | 0.469406615 | 1 |
| 201141_at | GPNMB | glycoprotein (transmembrane) nmb | 2.07E-16 | 1 | 0.001466787 | 1 |
| 202998_s_at | LOXL2 | lysyl oxidase-like 2 | 2.14E-16 | 1 | 1.92E-07 | 1 |
| 225328_at | NA | NA | 2.42E-16 | 0.835232341 | 0.76149608 | 0.757924823 |
| 202869_at | OAS1 | 2',5'-oligoadenylate synthetase 1, 40/46kDa | 3.71E-16 | 1 | 0.76149608 | 0.988209081 |
| 218400_at | OAS3 | 2'-5'-oligoadenylate synthetase 3, 100kDa | 3.86E-16 | 1 | 0.547698392 | 1 |
| 203780_at | MPZL2 | myelin protein zero-like 2 | 6.28E-16 | 1 | 0.042886126 | 1 |
| 201286_at | SDC1 | syndecan 1 | 6.54E-16 | 1 | 0.029652562 | 1 |
| 204141_at | TUBB2A | tubulin, beta 2A | 6.65E-16 | 1 | 0.558036417 | 1 |
| 223194_s_at | C6orf85 | chromosome 6 open reading frame 85 | 6.79E-16 | 1 | 0.76149608 | 0.920901663 |
| 226930_at | FNDC1 | fibronectin type III domain containing 1 | 6.79E-16 | 1 | 0.00010516 | 1 |
| 1555778_a_at | POSTN | periostin, osteoblast specific factor | 6.79E-16 | 1 | 3.41E-05 | 1 |
| 202581_at | HSPA1B | heat shock 70kDa protein 1B | 6.90E-16 | 1 | 0.76149608 | 1 |
| 210715_s_at | SPINT2 | serine peptidase inhibitor, Kunitz type, 2 | 8.18E-16 | 1 | 0.020839745 | 1 |
| 211538_s_at | HSPA2 | heat shock 70kDa protein 2 | 8.24E-16 | 1 | 0.241440682 | 1 |
| 209576_at | GNAI1 | guanine nucleotide binding protein (G protein), alpha inhibiting activity polypeptide 1 | 8.68E-16 | 1 | 0.68549602 | 1 |
| 31874_at | GAS2L1 | growth arrest-specific 2 like 1 | 8.68E-16 | 1 | 0.092371458 | 1 |
| 1555480_a_at | FBLIM1 | filamin binding LIM protein 1 | 8.72E-16 | 1 | 4.00E-06 | 1 |
| 226188_at | HSPC159 | galectin-related protein | 8.86E-16 | 1 | 0.76149608 | 1 |
| 238455_at | NA | NA | 1.03E-15 | 1 | 0.097699792 | 1 |
| 213139_at | SNAI2 | snail homolog 2 (Drosophila) | 1.08E-15 | 1 | 0.017051141 | 1 |
| 225540_at | MAP2 | microtubule-associated protein 2 | 1.50E-15 | 1 | 0.806671022 | 1 |
| 243296_at | PBEF1 | pre-B-cell colony enhancing factor 1 | 1.69E-15 | 1 | 0.028862217 | 1 |
| 218943_s_at | DDX58 | DEAD (Asp-Glu-Ala-Asp) box polypeptide 58 | 1.69E-15 | 1 | 0.113999991 | 1 |
| 226560_at | NA | NA | 1.74E-15 | 1 | 0.921602941 | 1 |
| 1568611_at | P4HA2 | procollagen-proline, 2-oxoglutarate 4-dioxygenase (proline 4-hydroxylase), alpha polypeptide II | 1.93E-15 | 1 | 0.0024257 | 1 |
| 201549_x_at | JARID1B | jumonji, AT rich interactive domain 1B | 2.52E-15 | 1 | 0.341534375 | 1 |
| 205016_at | TGFA | transforming growth factor, alpha | 2.84E-15 | 1 | 0.76149608 | 1 |
| 224799_at | NDFIP2 | Nedd4 family interacting protein 2 | 2.91E-15 | 1 | 0.087417485 | 1 |
| 227388_at | TUSC1 | tumor suppressor candidate 1 | 2.97E-15 | 1 | 0.362753182 | 1 |
| 201468_s_at | NQO1 | NAD(P)H dehydrogenase, quinone 1 | 2.97E-15 | 1 | 0.859922759 | 0.757924823 |
| 201243_s_at | ATP1B1 | ATPase, Na+/K+ transporting, beta 1 polypeptide | 3.15E-15 | 1 | 1.05E-05 | 1 |
| 203083_at | THBS2 | thrombospondin 2 | 3.56E-15 | 1 | 6.23E-06 | 1 |
| 202729_s_at | LTBP1 | latent transforming growth factor beta binding protein 1 | 4.00E-15 | 1 | 0.324551008 | 1 |
| 204201_s_at | PTPN13 | protein tyrosine phosphatase, non-receptor type 13 (APO-1/CD95 (Fas)-associated phosphatase) | 4.04E-15 | 1 | 0.76149608 | 1 |
| 218002_s_at | CXCL14 | chemokine (C-X-C motif) ligand 14 | 5.94E-15 | 1 | 2.92E-13 | 1 |
| 224937_at | PTGFRN | prostaglandin F2 receptor negative regulator | 7.15E-15 | 1 | 2.05E-05 | 1 |
| 227692_at | GNAI1 | guanine nucleotide binding protein (G protein), alpha inhibiting activity polypeptide 1 | 7.15E-15 | 1 | 0.769711909 | 1 |
| 202672_s_at | ATF3 | activating transcription factor 3 | 7.15E-15 | 1 | 0.76149608 | 1 |
| 210749_x_at | DDR1 | discoidin domain receptor family, member 1 | 8.52E-15 | 1 | 0.005542578 | 1 |
| 226817_at | DSC2 | desmocollin 2 | 1.10E-14 | 1 | 0.315751047 | 1 |
| 213905_x_at | BGN | biglycan | 1.31E-14 | 1 | 1.02E-06 | 1 |
| 228531_at | SAMD9 | sterile alpha motif domain containing 9 | 1.50E-14 | 1 | 0.76149608 | 0.991738636 |
| 212977_at | CXCR7 | chemokine (C-X-C motif) receptor 7 | 1.95E-14 | 1 | 2.21E-06 | 1 |
| 208898_at | ATP6V1D | ATPase, H+ transporting, lysosomal 34kDa, V1 subunit D | 2.40E-14 | 1 | 0.013072735 | 1 |
| 226545_at | CD109 | CD109 molecule | 2.42E-14 | 1 | 0.533729056 | 1 |
| 225018_at | SPIRE1 | spire homolog 1 (Drosophila) | 2.47E-14 | 1 | 0.638533355 | 1 |
| 201069_at | MMP2 | matrix metallopeptidase 2 (gelatinase A, 72kDa gelatinase, 72kDa type IV collagenase) | 2.56E-14 | 1 | 5.03E-12 | 1 |
| 211681_s_at | PDLIM5 | PDZ and LIM domain 5 | 2.89E-14 | 1 | 0.76149608 | 1 |
| 225016_at | APCDD1 | adenomatosis polyposis coli down-regulated 1 | 3.21E-14 | 1 | 0.087354305 | 1 |
| 205713_s_at | COMP | cartilage oligomeric matrix protein | 3.22E-14 | 1 | 0.000171342 | 0.999109147 |
| 200632_s_at | NDRG1 | N-myc downstream regulated gene 1 | 6.48E-14 | 1 | 0.08458589 | 1 |
| 226769_at | FIBIN | fin bud initiation factor | 6.50E-14 | 0.218465576 | 0.970413281 | 0.996729349 |
| 204341_at | TRIM16 | tripartite motif-containing 16 | 7.04E-14 | 1 | 0.76149608 | 1 |
| 206385_s_at | ANK3 | ankyrin 3, node of Ranvier (ankyrin G) | 7.34E-14 | 1 | 0.89232255 | 1 |
| 222757_s_at | ZAK | sterile alpha motif and leucine zipper containing kinase AZK | 7.45E-14 | 1 | 0.7026286 | 1 |
| 235733_at | NA | NA | 7.52E-14 | 0.835232341 | 0.314991708 | 1 |
| 207426_s_at | TNFSF4 | tumor necrosis factor (ligand) superfamily, member 4 (tax-transcriptionally activated glycoprotein 1, 34kDa) | 7.52E-14 | 0.835232341 | 0.266301628 | 0.941822835 |
| 203987_at | FZD6 | frizzled homolog 6 (Drosophila) | 8.03E-14 | 1 | 0.002681689 | 1 |
| 215071_s_at | HIST1H2AC | histone cluster 1, H2ac | 9.02E-14 | 1 | 0.03516974 | 0.992642041 |
| 226421_at | AMMECR1 | Alport syndrome, mental retardation, midface hypoplasia and elliptocytosis chromosomal region, gene 1 | 1.06E-13 | 1 | 0.76149608 | 1 |
| 209596_at | MXRA5 | matrix-remodelling associated 5 | 1.09E-13 | 1 | 5.65E-05 | 1 |
| 200606_at | DSP | desmoplakin | 1.18E-13 | 1 | 0.006248843 | 1 |
| 203695_s_at | DFNA5 | deafness, autosomal dominant 5 | 1.18E-13 | 1 | 4.14E-07 | 1 |
| 212765_at | CAMSAP1L1 | calmodulin regulated spectrin-associated protein 1-like 1 | 1.28E-13 | 1 | 0.059812998 | 1 |
| 212489_at | COL5A1 | collagen, type V, alpha 1 | 1.35E-13 | 1 | 5.08E-07 | 1 |
| 206284_x_at | CLTB | clathrin, light chain (Lcb) | 1.38E-13 | 1 | 0.170664325 | 1 |
| 225911_at | NPNT | nephronectin | 1.68E-13 | 1 | 0.879403075 | 0.757924823 |
| 221841_s_at | KLF4 | Kruppel-like factor 4 (gut) | 1.68E-13 | 1 | 0.76149608 | 1 |
| 220161_s_at | EPB41L4B | erythrocyte membrane protein band 4.1 like 4B | 1.74E-13 | 1 | 0.76149608 | 1 |
| 215646_s_at | VCAN | versican | 1.92E-13 | 1 | 1.75E-05 | 1 |
| 226612_at | FLJ25076 | similar to CG4502-PA | 2.19E-13 | 1 | 0.708255882 | 0.972861351 |
| 229450_at | IFIT3 | interferon-induced protein with tetratricopeptide repeats 3 | 2.29E-13 | 1 | 0.007461286 | 1 |
| 217820_s_at | ENAH | enabled homolog (Drosophila) | 2.40E-13 | 1 | 0.015081018 | 1 |
| 204675_at | SRD5A1 | steroid-5-alpha-reductase, alpha polypeptide 1 (3-oxo-5 alpha-steroid delta 4-dehydrogenase alpha 1) | 2.75E-13 | 1 | 0.76149608 | 1 |
| 213764_s_at | MFAP5 | microfibrillar associated protein 5 | 2.80E-13 | 0.835232341 | 0.563184621 | 1 |
| 209191_at | TUBB6 | tubulin, beta 6 | 3.37E-13 | 1 | 0.000400438 | 1 |
| 227492_at | LOC647859 | occludin pseudogene | 3.60E-13 | 1 | 0.838446519 | 1 |
| 201744_s_at | LUM | lumican | 3.67E-13 | 1 | 0.000110294 | 1 |
| 209218_at | SQLE | squalene epoxidase | 4.12E-13 | 1 | 0.615598167 | 1 |
| 212488_at | COL5A1 | collagen, type V, alpha 1 | 4.39E-13 | 1 | 6.78E-06 | 1 |
| 204485_s_at | TOM1L1 | target of myb1 (chicken)-like 1 | 5.39E-13 | 1 | 0.76149608 | 1 |
| 213476_x_at | TUBB3 | tubulin, beta 3 | 5.64E-13 | 1 | 0.001687361 | 1 |
| 225366_at | PGM2 | phosphoglucomutase 2 | 6.24E-13 | 1 | 0.76149608 | 1 |
| 201005_at | CD9 | CD9 molecule | 7.53E-13 | 1 | 0.36231129 | 1 |
| 201656_at | ITGA6 | integrin, alpha 6 | 1.18E-12 | 1 | 0.004453494 | 1 |
| 203820_s_at | IGF2BP3 | insulin-like growth factor 2 mRNA binding protein 3 | 1.22E-12 | 1 | 0.119037277 | 1 |
| 202949_s_at | FHL2 | four and a half LIM domains 2 | 1.22E-12 | 1 | 0.011054555 | 1 |
| 215813_s_at | PTGS1 | prostaglandin-endoperoxide synthase 1 (prostaglandin G/H synthase and cyclooxygenase) | 1.39E-12 | 1 | 0.311840926 | 1 |
| 214710_s_at | CCNB1 | cyclin B1 | 1.50E-12 | 1 | 0.065967196 | 1 |
| 229554_at | NA | NA | 1.55E-12 | 1 | 0.000147876 | 1 |
| 210427_x_at | ANXA2 | annexin A2 | 1.56E-12 | 1 | 0.0233217 | 1 |
| 200999_s_at | CKAP4 | cytoskeleton-associated protein 4 | 1.65E-12 | 1 | 8.25E-06 | 1 |
| 225288_at | NA | NA | 1.83E-12 | 1 | 0.057758615 | 1 |
| 203726_s_at | LAMA3 | laminin, alpha 3 | 1.83E-12 | 1 | 1.00E-08 | 1 |
| 32137_at | JAG2 | jagged 2 | 1.95E-12 | 1 | 0.006002302 | 1 |
| 205128_x_at | PTGS1 | prostaglandin-endoperoxide synthase 1 (prostaglandin G/H synthase and cyclooxygenase) | 1.96E-12 | 1 | 0.241779614 | 1 |
| 218309_at | CAMK2N1 | calcium/calmodulin-dependent protein kinase II inhibitor 1 | 1.96E-12 | 1 | 0.016561005 | 1 |
| 212983_at | HRAS | v-Ha-ras Harvey rat sarcoma viral oncogene homolog | 1.97E-12 | 1 | 0.141484043 | 1 |
| 201681_s_at | DLG5 | discs, large homolog 5 (Drosophila) | 1.98E-12 | 1 | 0.101165656 | 1 |
| 32128_at | CCL18 | chemokine (C-C motif) ligand 18 (pulmonary and activation-regulated) | 2.15E-12 | 1 | 1.41E-05 | 1 |
| 203510_at | MET | met proto-oncogene (hepatocyte growth factor receptor) | 2.21E-12 | 1 | 0.429225403 | 1 |
| 204779_s_at | HOXB7 | homeobox B7 | 2.66E-12 | 1 | 0.051747094 | 0.854886879 |
| 217911_s_at | BAG3 | BCL2-associated athanogene 3 | 2.79E-12 | 1 | 0.100799433 | 1 |
| 211571_s_at | VCAN | versican | 3.09E-12 | 1 | 3.13E-07 | 1 |
| 228570_at | BTBD11 | BTB (POZ) domain containing 11 | 3.09E-12 | 1 | 0.76149608 | 1 |
| 241762_at | FBXO32 | F-box protein 32 | 3.96E-12 | 0.835232341 | 0.107231996 | 0.757924823 |
| 213503_x_at | ANXA2 | annexin A2 | 4.10E-12 | 1 | 0.022246587 | 1 |
| 209946_at | VEGFC | vascular endothelial growth factor C | 5.08E-12 | 1 | 1.05E-05 | 0.957875601 |
| 227204_at | PARD6G | par-6 partitioning defective 6 homolog gamma (C. elegans) | 5.32E-12 | 1 | 0.76149608 | 1 |
| 222484_s_at | CXCL14 | chemokine (C-X-C motif) ligand 14 | 5.36E-12 | 1 | 5.00E-12 | 1 |
| 222847_s_at | EGLN3 | egl nine homolog 3 (C. elegans) | 5.52E-12 | 1 | 0.048335098 | 1 |
| 211612_s_at | IL13RA1 | interleukin 13 receptor, alpha 1 | 5.60E-12 | 1 | 0.062699237 | 1 |
| 228152_s_at | FLJ31033 | hypothetical protein FLJ31033 | 5.73E-12 | 1 | 0.76149608 | 0.876704339 |
| 209822_s_at | VLDLR | very low density lipoprotein receptor | 6.38E-12 | 1 | 0.811074901 | 0.996778025 |
| 225612_s_at | B3GNT5 | UDP-GlcNAc:betaGal beta-1,3-N-acetylglucosaminyltransferase 5 | 6.41E-12 | 1 | 0.003283091 | 1 |
| 212325_at | LIMCH1 | LIM and calponin homology domains 1 | 6.49E-12 | 1 | 0.88072492 | 0.861615689 |
| 215177_s_at | ITGA6 | integrin, alpha 6 | 7.07E-12 | 1 | 0.231443573 | 1 |
| 219463_at | C20orf103 | chromosome 20 open reading frame 103 | 7.09E-12 | 1 | 0.236333725 | 0.757924823 |
| 214734_at | EXPH5 | exophilin 5 | 7.10E-12 | 1 | 0.76149608 | 1 |
| 208637_x_at | ACTN1 | actinin, alpha 1 | 7.56E-12 | 1 | 2.55E-12 | 1 |
| 213652_at | PCSK5 | proprotein convertase subtilisin/kexin type 5 | 8.30E-12 | 0.980995047 | 0.76149608 | 1 |
| 201015_s_at | JUP | junction plakoglobin | 8.53E-12 | 1 | 0.266468415 | 1 |
| 211651_s_at | LAMB1 | laminin, beta 1 | 9.75E-12 | 1 | 0.000107906 | 1 |
| 204972_at | OAS2 | 2'-5'-oligoadenylate synthetase 2, 69/71kDa | 9.93E-12 | 1 | 0.673521734 | 1 |
| 212344_at | SULF1 | sulfatase 1 | 1.11E-11 | 1 | 1.73E-06 | 1 |
| 204714_s_at | F5 | coagulation factor V (proaccelerin, labile factor) | 1.12E-11 | 0.835232341 | 0.76149608 | 0.757924823 |
| 225726_s_at | PLEKHH1 | pleckstrin homology domain containing, family H (with MyTH4 domain) member 1 | 1.28E-11 | 1 | 0.517971546 | 1 |
| 211597_s_at | HOP | homeodomain-only protein | 1.48E-11 | 1 | 0.76149608 | 1 |
| 226498_at | FLT1 | fms-related tyrosine kinase 1 (vascular endothelial growth factor/vascular permeability factor receptor) | 1.58E-11 | 1 | 0.76149608 | 0.946501103 |
| 201564_s_at | FSCN1 | fascin homolog 1, actin-bundling protein (Strongylocentrotus purpuratus) | 1.66E-11 | 1 | 0.160905078 | 1 |
| 209911_x_at | HIST1H2BD | histone cluster 1, H2bd | 1.67E-11 | 1 | 0.016985377 | 0.831433622 |
| 233814_at | NA | NA | 1.68E-11 | 1 | 0.856109771 | 1 |
| 201201_at | CSTB | cystatin B (stefin B) | 1.69E-11 | 1 | 0.204435372 | 1 |
| 205220_at | GPR109B | G protein-coupled receptor 109B | 1.74E-11 | 1 | 0.062477029 | 1 |
| 214651_s_at | HOXA9 | homeobox A9 | 1.85E-11 | 1 | 0.76149608 | 1 |
| 240991_at | NDRG1 | N-myc downstream regulated gene 1 | 2.05E-11 | 1 | 0.32537301 | 0.904531166 |
| 201846_s_at | RYBP | RING1 and YY1 binding protein | 2.31E-11 | 0.87107257 | 0.76149608 | 0.757924823 |
| 231183_s_at | JAG1 | jagged 1 (Alagille syndrome) | 2.51E-11 | 1 | 2.31E-06 | 1 |
| 208581_x_at | MT1X | metallothionein 1X | 2.53E-11 | 1 | 0.000164294 | 1 |
| 229546_at | LOC653602 | hypothetical LOC653602 | 2.71E-11 | 1 | 1 | 1 |
| 205495_s_at | GNLY | granulysin | 2.89E-11 | 0.835232341 | 0.76149608 | 1 |
| 204619_s_at | VCAN | versican | 3.47E-11 | 1 | 0.000107678 | 1 |
| 228033_at | E2F7 | E2F transcription factor 7 | 3.56E-11 | 1 | 0.059812998 | 1 |
| 205559_s_at | PCSK5 | proprotein convertase subtilisin/kexin type 5 | 4.76E-11 | 1 | 0.271420665 | 1 |
| 202800_at | SLC1A3 | solute carrier family 1 (glial high affinity glutamate transporter), member 3 | 4.92E-11 | 1 | 0.76149608 | 1 |
| 202912_at | ADM | adrenomedullin | 5.84E-11 | 1 | 0.76149608 | 1 |
| 1560926_at | PPP4R2 | protein phosphatase 4, regulatory subunit 2 | 6.47E-11 | 0.835232341 | 1 | 0.757924823 |
| 203764_at | DLG7 | discs, large homolog 7 (Drosophila) | 6.47E-11 | 1 | 0.011285705 | 1 |
| 227960_s_at | FAHD1 | fumarylacetoacetate hydrolase domain containing 1 | 6.88E-11 | 1 | 0.749281805 | 1 |
| 213288_at | MBOAT2 | membrane bound O-acyltransferase domain containing 2 | 7.19E-11 | 1 | 0.76149608 | 0.89381914 |
| 209803_s_at | PHLDA2 | pleckstrin homology-like domain, family A, member 2 | 7.30E-11 | 1 | 0.235170379 | 1 |
| 208999_at | SEPT8 | septin 8 | 7.86E-11 | 1 | 0.360302879 | 1 |
| 201844_s_at | RYBP | RING1 and YY1 binding protein | 7.92E-11 | 0.835232341 | 0.856554481 | 0.757924823 |
| 232164_s_at | EPPK1 | epiplakin 1 | 7.93E-11 | 1 | 0.76149608 | 1 |
| 201125_s_at | ITGB5 | integrin, beta 5 | 8.50E-11 | 1 | 0.009618142 | 1 |
| 227628_at | LOC493869 | similar to RIKEN cDNA 2310016C16 | 8.79E-11 | 1 | 5.72E-06 | 1 |
| 228141_at | LOC493869 | similar to RIKEN cDNA 2310016C16 | 9.07E-11 | 1 | 0.117864256 | 1 |
| 201169_s_at | BHLHB2 | basic helix-loop-helix domain containing, class B, 2 | 9.73E-11 | 1 | 0.76149608 | 1 |
| 204747_at | IFIT3 | interferon-induced protein with tetratricopeptide repeats 3 | 1.20E-10 | 1 | 0.008915449 | 1 |
| 225056_at | SIPA1L2 | signal-induced proliferation-associated 1 like 2 | 1.21E-10 | 1 | 0.76149608 | 1 |
| 219232_s_at | EGLN3 | egl nine homolog 3 (C. elegans) | 1.23E-10 | 1 | 0.76149608 | 0.993346543 |
| 213154_s_at | BICD2 | bicaudal D homolog 2 (Drosophila) | 1.32E-10 | 1 | 0.76149608 | 1 |
| 218888_s_at | NETO2 | neuropilin (NRP) and tolloid (TLL)-like 2 | 1.34E-10 | 1 | 0.123599671 | 1 |
| 201984_s_at | EGFR | epidermal growth factor receptor (erythroblastic leukemia viral (v-erb-b) oncogene homolog, avian) | 1.45E-10 | 1 | 0.327652185 | 1 |
| 219956_at | GALNT6 | UDP-N-acetyl-alpha-D-galactosamine:polypeptide N-acetylgalactosaminyltransferase 6 (GalNAc-T6) | 1.69E-10 | 1 | 0.241779614 | 1 |
| 212327_at | LIMCH1 | LIM and calponin homology domains 1 | 1.70E-10 | 0.835232341 | 0.987730377 | 0.757924823 |
| 218507_at | HIG2 | hypoxia-inducible protein 2 | 1.70E-10 | 1 | 0.76149608 | 1 |
| 211564_s_at | PDLIM4 | PDZ and LIM domain 4 | 1.72E-10 | 1 | 0.000133435 | 1 |
| 227072_at | RTTN | rotatin | 1.87E-10 | 1 | 0.76149608 | 1 |
| 203394_s_at | HES1 | hairy and enhancer of split 1, (Drosophila) | 2.08E-10 | 1 | 0.76149608 | 1 |
| 219959_at | MOCOS | molybdenum cofactor sulfurase | 2.12E-10 | 1 | 0.34209183 | 1 |
| 212070_at | GPR56 | G protein-coupled receptor 56 | 2.12E-10 | 1 | 0.04355669 | 1 |
| 212185_x_at | MT2A | metallothionein 2A | 2.14E-10 | 1 | 9.71E-05 | 1 |
| 211043_s_at | CLTB | clathrin, light chain (Lcb) | 2.20E-10 | 1 | 0.346820034 | 1 |
| 207714_s_at | SERPINH1 | serpin peptidase inhibitor, clade H (heat shock protein 47), member 1, (collagen binding protein 1) | 2.28E-10 | 1 | 0.000120556 | 1 |
| 214043_at | PTPRD | protein tyrosine phosphatase, receptor type, D | 2.31E-10 | 1 | 0.6861386 | 0.98400861 |
| 201251_at | PKM2 | pyruvate kinase, muscle | 2.31E-10 | 1 | 0.018753407 | 1 |
| 231882_at | LOC440157 | hypothetical gene supported by BC066547 | 2.33E-10 | 0.835232341 | 0.76149608 | 0.757924823 |
| 219787_s_at | ECT2 | epithelial cell transforming sequence 2 oncogene | 2.52E-10 | 1 | 0.00066111 | 1 |
| 204844_at | ENPEP | glutamyl aminopeptidase (aminopeptidase A) | 2.75E-10 | 1 | 0.76149608 | 1 |
| 209694_at | PTS | 6-pyruvoyltetrahydropterin synthase | 3.05E-10 | 1 | 0.06989674 | 1 |
| 210074_at | CTSL2 | cathepsin L2 | 3.39E-10 | 1 | 0.118116141 | 1 |
| 1554452_a_at | HIG2 | hypoxia-inducible protein 2 | 3.52E-10 | 1 | 0.06938809 | 1 |
| 214168_s_at | TJP1 | tight junction protein 1 (zona occludens 1) | 3.56E-10 | 1 | 0.76149608 | 1 |
| 218280_x_at | HIST2H2AA3 | histone cluster 2, H2aa3 | 3.60E-10 | 1 | 1.02E-06 | 1 |
| 201841_s_at | HSPB1 | heat shock 27kDa protein 1 | 3.66E-10 | 1 | 0.000359295 | 1 |
| 218984_at | PUS7 | pseudouridylate synthase 7 homolog (S. cerevisiae) | 3.72E-10 | 1 | 0.76149608 | 1 |
| 203367_at | DUSP14 | dual specificity phosphatase 14 | 3.81E-10 | 1 | 0.565395268 | 1 |
| 212812_at | SERINC5 | serine incorporator 5 | 4.10E-10 | 1 | 0.76149608 | 1 |
| 201287_s_at | SDC1 | syndecan 1 | 4.11E-10 | 1 | 0.180804398 | 1 |
| 1555564_a_at | CFI | complement factor I | 4.56E-10 | 1 | 0.76149608 | 0.757924823 |
| 33646_g_at | GM2A | GM2 ganglioside activator | 5.69E-10 | 1 | 0.76149608 | 1 |
| 226752_at | TMEM157 | transmembrane protein 157 | 5.80E-10 | 1 | 0.76149608 | 1 |
| 201845_s_at | RYBP | RING1 and YY1 binding protein | 5.95E-10 | 0.854558522 | 0.785825431 | 0.757924823 |
| 223062_s_at | PSAT1 | phosphoserine aminotransferase 1 | 6.37E-10 | 1 | 0.76149608 | 0.757924823 |
| 200737_at | PGK1 | phosphoglycerate kinase 1 | 6.89E-10 | 1 | 0.565395268 | 1 |
| 201641_at | BST2 | bone marrow stromal cell antigen 2 | 6.98E-10 | 1 | 0.477789132 | 1 |
| 209211_at | KLF5 | Kruppel-like factor 5 (intestinal) | 7.13E-10 | 1 | 0.586408931 | 1 |
| 1554079_at | GALNTL4 | UDP-N-acetyl-alpha-D-galactosamine:polypeptide N-acetylgalactosaminyltransferase-like 4 | 8.14E-10 | 1 | 0.76149608 | 0.970101956 |
| 215891_s_at | GM2A | GM2 ganglioside activator | 9.23E-10 | 1 | 0.76149608 | 1 |
| 203397_s_at | GALNT3 | UDP-N-acetyl-alpha-D-galactosamine:polypeptide N-acetylgalactosaminyltransferase 3 (GalNAc-T3) | 9.31E-10 | 1 | 0.173329633 | 1 |
| 1556499_s_at | COL1A1 | collagen, type I, alpha 1 | 9.31E-10 | 1 | 2.50E-05 | 1 |
| 202284_s_at | CDKN1A | cyclin-dependent kinase inhibitor 1A (p21, Cip1) | 9.40E-10 | 1 | 0.020287131 | 1 |
| 226804_at | FAM20A | family with sequence similarity 20, member A | 9.45E-10 | 1 | 0.099059447 | 1 |
| 226600_at | TMTC3 | transmembrane and tetratricopeptide repeat containing 3 | 9.87E-10 | 1 | 0.03934677 | 1 |
| 204948_s_at | FST | follistatin | 1.06E-09 | 1 | 0.76149608 | 1 |
| 235944_at | HMCN1 | hemicentin 1 | 1.39E-09 | 1 | 0.362753182 | 1 |
| 217997_at | PHLDA1 | pleckstrin homology-like domain, family A, member 1 | 1.50E-09 | 1 | 0.104053614 | 1 |
| 230422_at | FPRL2 | formyl peptide receptor-like 2 | 1.59E-09 | 1 | 8.32E-06 | 1 |
| 210087_s_at | MPZL1 | myelin protein zero-like 1 | 1.59E-09 | 1 | 0.088908552 | 1 |
| 208370_s_at | RCAN1 | regulator of calcineurin 1 | 1.62E-09 | 1 | 0.371648455 | 1 |
| 219918_s_at | ASPM | asp (abnormal spindle) homolog, microcephaly associated (Drosophila) | 1.79E-09 | 1 | 0.113270514 | 1 |
| 212171_x_at | VEGFA | vascular endothelial growth factor A | 1.93E-09 | 1 | 0.76149608 | 0.892416223 |
| 222036_s_at | MCM4 | minichromosome maintenance complex component 4 | 2.16E-09 | 1 | 0.049595797 | 1 |
| 208744_x_at | HSPH1 | heat shock 105kDa/110kDa protein 1 | 2.26E-09 | 1 | 0.76149608 | 1 |
| 209309_at | AZGP1 | alpha-2-glycoprotein 1, zinc-binding | 2.35E-09 | 1 | 0.76149608 | 1 |
| 202539_s_at | HMGCR | 3-hydroxy-3-methylglutaryl-Coenzyme A reductase | 2.58E-09 | 1 | 0.76149608 | 1 |
| 201261_x_at | BGN | biglycan | 2.60E-09 | 1 | 0.006258245 | 1 |
| 201110_s_at | THBS1 | thrombospondin 1 | 2.63E-09 | 1 | 2.85E-07 | 1 |
| 201579_at | FAT | FAT tumor suppressor homolog 1 (Drosophila) | 2.69E-09 | 1 | 0.542196764 | 1 |
| 201250_s_at | SLC2A1 | solute carrier family 2 (facilitated glucose transporter), member 1 | 2.77E-09 | 1 | 0.029133622 | 1 |
| 209398_at | HIST1H1C | histone cluster 1, H1c | 3.45E-09 | 1 | 0.76149608 | 0.757924823 |
| 203126_at | IMPA2 | inositol(myo)-1(or 4)-monophosphatase 2 | 3.53E-09 | 1 | 0.426941484 | 1 |
| 219250_s_at | FLRT3 | fibronectin leucine rich transmembrane protein 3 | 3.91E-09 | 1 | 0.76149608 | 1 |
| 224773_at | NAV1 | neuron navigator 1 | 3.92E-09 | 1 | 0.118823265 | 1 |
| 210933_s_at | FSCN1 | fascin homolog 1, actin-bundling protein (Strongylocentrotus purpuratus) | 4.02E-09 | 1 | 0.29094326 | 1 |
| 235144_at | NA | NA | 4.13E-09 | 1 | 0.918570202 | 0.757924823 |
| 219906_at | FLJ10213 | hypothetical protein FLJ10213 | 4.24E-09 | 0.835232341 | 0.888229531 | 0.757924823 |
| 203819_s_at | C7orf30 | chromosome 7 open reading frame 30 | 4.34E-09 | 1 | 0.141681673 | 1 |
| 205100_at | GFPT2 | glutamine-fructose-6-phosphate transaminase 2 | 4.53E-09 | 1 | 5.95E-05 | 1 |
| 213508_at | C14orf147 | chromosome 14 open reading frame 147 | 4.91E-09 | 1 | 0.76149608 | 1 |
| 212680_x_at | LOC644879 | hypothetical LOC644879 | 5.28E-09 | 1 | 0.000758037 | 1 |
| 201325_s_at | EMP1 | epithelial membrane protein 1 | 5.84E-09 | 1 | 0.76149608 | 1 |
| 213793_s_at | HOMER1 | homer homolog 1 (Drosophila) | 6.07E-09 | 1 | 0.06364937 | 1 |
| 229810_at | NA | NA | 6.60E-09 | 1 | 0.655286025 | 0.968344116 |
| 206245_s_at | IVNS1ABP | influenza virus NS1A binding protein | 6.60E-09 | 1 | 0.008378801 | 1 |
| 204620_s_at | VCAN | versican | 6.92E-09 | 1 | 0.000158399 | 1 |
| 228846_at | MXD1 | MAX dimerization protein 1 | 7.21E-09 | 1 | 0.727505852 | 1 |
| 236172_at | LTB4R | leukotriene B4 receptor | 7.21E-09 | 1 | 0.898285395 | 1 |
| 1007_s_at | DDR1 | discoidin domain receptor family, member 1 | 7.42E-09 | 1 | 0.090042628 | 1 |
| 225150_s_at | RTKN | rhotekin | 7.78E-09 | 1 | 0.005647179 | 1 |
| 212013_at | PXDN | peroxidasin homolog (Drosophila) | 7.83E-09 | 1 | 1.58E-11 | 1 |
| 204595_s_at | STC1 | stanniocalcin 1 | 8.08E-09 | 1 | 0.077668972 | 0.995261784 |
| 212012_at | PXDN | peroxidasin homolog (Drosophila) | 8.82E-09 | 1 | 7.93E-07 | 1 |
| 205732_s_at | NCOA2 | nuclear receptor coactivator 2 | 9.13E-09 | 1 | 0.881325715 | 0.8966871 |
| 208677_s_at | BSG | basigin (Ok blood group) | 9.62E-09 | 1 | 0.76149608 | 0.994466858 |
| 205499_at | SRPX2 | sushi-repeat-containing protein, X-linked 2 | 1.00E-08 | 1 | 0.004431265 | 1 |
| 201131_s_at | CDH1 | cadherin 1, type 1, E-cadherin (epithelial) | 1.02E-08 | 1 | 0.39054736 | 1 |
| 225314_at | OCIAD2 | OCIA domain containing 2 | 1.10E-08 | 1 | 0.224458812 | 0.7665302 |
| 211240_x_at | CTNND1 | catenin (cadherin-associated protein), delta 1 | 1.10E-08 | 1 | 0.488013356 | 1 |
| 207828_s_at | CENPF | centromere protein F, 350/400ka (mitosin) | 1.11E-08 | 1 | 0.384230819 | 1 |
| 213726_x_at | TUBB2C | tubulin, beta 2C | 1.15E-08 | 1 | 0.170485168 | 1 |
| 203411_s_at | LMNA | lamin A/C | 1.19E-08 | 1 | 0.012182795 | 1 |
| 201161_s_at | CSDA | cold shock domain protein A | 1.19E-08 | 1 | 0.572756439 | 1 |
| 238513_at | PRRG4 | proline rich Gla (G-carboxyglutamic acid) 4 (transmembrane) | 1.22E-08 | 1 | 0.373194686 | 1 |
| 225301_s_at | ACAA2 | acetyl-Coenzyme A acyltransferase 2 (mitochondrial 3-oxoacyl-Coenzyme A thiolase) | 1.32E-08 | 1 | 0.76149608 | 1 |
| 33322_i_at | SFN | stratifin | 1.36E-08 | 1 | 2.73E-05 | 1 |
| 219479_at | KDELC1 | KDEL (Lys-Asp-Glu-Leu) containing 1 | 1.46E-08 | 1 | 0.213805381 | 1 |
| 213552_at | GLCE | glucuronic acid epimerase | 1.53E-08 | 1 | 0.76149608 | 0.93009914 |
| 226425_at | CLIP4 | CAP-GLY domain containing linker protein family, member 4 | 1.59E-08 | 1 | 0.76149608 | 1 |
| 201939_at | PLK2 | polo-like kinase 2 (Drosophila) | 1.73E-08 | 1 | 0.618395117 | 1 |
| 239272_at | MMP28 | matrix metallopeptidase 28 | 1.82E-08 | 1 | 0.76149608 | 1 |
| 227609_at | EPSTI1 | epithelial stromal interaction 1 (breast) | 1.91E-08 | 1 | 0.398402774 | 1 |
| 225140_at | KLF3 | Kruppel-like factor 3 (basic) | 1.96E-08 | 1 | 0.76149608 | 1 |
| 222646_s_at | ERO1L | ERO1-like (S. cerevisiae) | 1.96E-08 | 1 | 0.011095673 | 1 |
| 228260_at | ELAVL2 | ELAV (embryonic lethal, abnormal vision, Drosophila)-like 2 (Hu antigen B) | 2.03E-08 | 1 | 0.76149608 | 1 |
| 204114_at | NID2 | nidogen 2 (osteonidogen) | 2.16E-08 | 1 | 0.000122087 | 1 |
| 226497_s_at | FLT1 | fms-related tyrosine kinase 1 (vascular endothelial growth factor/vascular permeability factor receptor) | 2.16E-08 | 1 | 0.76149608 | 0.90232871 |
| 200757_s_at | CALU | calumenin | 2.21E-08 | 1 | 0.043285306 | 1 |
| 227276_at | PLXDC2 | plexin domain containing 2 | 2.65E-08 | 1 | 0.10214739 | 1 |
| 217496_s_at | IDE | insulin-degrading enzyme | 2.65E-08 | 1 | 0.76149608 | 1 |
| 227140_at | NA | NA | 2.69E-08 | 1 | 0.001170659 | 1 |
| 227107_at | NA | NA | 2.69E-08 | 1 | 0.264295799 | 1 |
| 201559_s_at | CLIC4 | chloride intracellular channel 4 | 2.83E-08 | 1 | 0.76149608 | 1 |
| 225947_at | MYOHD1 | myosin head domain containing 1 | 2.84E-08 | 1 | 0.76149608 | 1 |
| 241985_at | JMY | junction-mediating and regulatory protein | 2.88E-08 | 1 | 0.76149608 | 1 |
| 223427_s_at | EPB41L4B | erythrocyte membrane protein band 4.1 like 4B | 2.97E-08 | 1 | 0.76149608 | 1 |
| 200636_s_at | PTPRF | protein tyrosine phosphatase, receptor type, F | 2.98E-08 | 1 | 0.375831321 | 1 |
| 225792_at | HOOK1 | hook homolog 1 (Drosophila) | 3.06E-08 | 1 | 0.76149608 | 0.757924823 |
| 226767_s_at | FAHD1 | fumarylacetoacetate hydrolase domain containing 1 | 3.10E-08 | 1 | 0.76149608 | 1 |
| 226279_at | PRSS23 | protease, serine, 23 | 3.19E-08 | 1 | 0.000492605 | 1 |
| 206133_at | XAF1 | XIAP associated factor-1 | 3.28E-08 | 1 | 0.76149608 | 1 |
| 204858_s_at | ECGF1 | endothelial cell growth factor 1 (platelet-derived) | 3.32E-08 | 1 | 0.004431265 | 1 |
| 220266_s_at | KLF4 | Kruppel-like factor 4 (gut) | 3.39E-08 | 1 | 0.76149608 | 1 |
| 228010_at | PPP2R2C | protein phosphatase 2 (formerly 2A), regulatory subunit B, gamma isoform | 3.39E-08 | 1 | 0.76149608 | 1 |
| 212328_at | LIMCH1 | LIM and calponin homology domains 1 | 3.44E-08 | 0.97349211 | 0.980902924 | 0.757924823 |
| 224097_s_at | F11R | F11 receptor | 3.44E-08 | 1 | 0.235170379 | 1 |
| 204647_at | HOMER3 | homer homolog 3 (Drosophila) | 3.48E-08 | 1 | 0.004780681 | 1 |
| 225806_at | JUB | jub, ajuba homolog (Xenopus laevis) | 3.51E-08 | 1 | 0.007613737 | 1 |
| 219347_at | NUDT15 | nudix (nucleoside diphosphate linked moiety X)-type motif 15 | 3.59E-08 | 1 | 0.76149608 | 1 |
| 201590_x_at | ANXA2 | annexin A2 | 3.60E-08 | 1 | 0.229144298 | 1 |
| 213419_at | APBB2 | amyloid beta (A4) precursor protein-binding, family B, member 2 (Fe65-like) | 3.66E-08 | 1 | 0.100799433 | 0.831038248 |
| 222664_at | KCTD15 | potassium channel tetramerisation domain containing 15 | 3.72E-08 | 1 | 0.76149608 | 1 |
| 200923_at | LGALS3BP | lectin, galactoside-binding, soluble, 3 binding protein | 3.86E-08 | 1 | 0.000793097 | 1 |
| 229218_at | COL1A2 | collagen, type I, alpha 2 | 4.07E-08 | 1 | 3.97E-16 | 1 |
| 213428_s_at | COL6A1 | collagen, type VI, alpha 1 | 4.19E-08 | 1 | 2.45E-06 | 1 |
| 219083_at | SHQ1 | SHQ1 homolog (S. cerevisiae) | 4.25E-08 | 0.835232341 | 0.76149608 | 0.764860713 |
| 225842_at | PHLDA1 | pleckstrin homology-like domain, family A, member 1 | 4.27E-08 | 1 | 0.246250467 | 1 |
| 219215_s_at | SLC39A4 | solute carrier family 39 (zinc transporter), member 4 | 4.30E-08 | 1 | 0.76149608 | 1 |
| 211161_s_at | COL3A1 | collagen, type III, alpha 1 (Ehlers-Danlos syndrome type IV, autosomal dominant) | 4.30E-08 | 1 | 1.14E-09 | 1 |
| 222981_s_at | RAB10 | RAB10, member RAS oncogene family | 4.33E-08 | 1 | 0.76149608 | 1 |
| 202619_s_at | PLOD2 | procollagen-lysine, 2-oxoglutarate 5-dioxygenase 2 | 4.42E-08 | 1 | 6.28E-08 | 1 |
| 213797_at | RSAD2 | radical S-adenosyl methionine domain containing 2 | 4.43E-08 | 1 | 0.615857642 | 1 |
| 216973_s_at | HOXB7 | homeobox B7 | 4.49E-08 | 1 | 0.078442783 | 0.952164328 |
| 217744_s_at | PERP | PERP, TP53 apoptosis effector | 4.53E-08 | 1 | 0.116128623 | 1 |
| 229947_at | PI15 | peptidase inhibitor 15 | 4.53E-08 | 1 | 1.36E-15 | 1 |
| 223121_s_at | SFRP2 | secreted frizzled-related protein 2 | 4.53E-08 | 1 | 0.011245127 | 1 |
| 218355_at | KIF4A | kinesin family member 4A | 4.53E-08 | 1 | 0.10214739 | 1 |
| 229635_at | NA | NA | 4.66E-08 | 1 | 0.016045804 | 1 |
| 1561180_at | LRP11 | low density lipoprotein receptor-related protein 11 | 4.66E-08 | 1 | 0.018356159 | 0.778337768 |
| 205381_at | LRRC17 | leucine rich repeat containing 17 | 4.73E-08 | 0.835232341 | 0.76149608 | 1 |
| 205660_at | OASL | 2'-5'-oligoadenylate synthetase-like | 4.79E-08 | 1 | 0.345983402 | 1 |
| 225262_at | FOSL2 | FOS-like antigen 2 | 4.85E-08 | 1 | 0.146840734 | 1 |
| 204030_s_at | SCHIP1 | schwannomin interacting protein 1 | 5.35E-08 | 1 | 0.001464489 | 1 |
| 221541_at | CRISPLD2 | cysteine-rich secretory protein LCCL domain containing 2 | 5.53E-08 | 1 | 1.25E-05 | 1 |
| 201490_s_at | PPIF | peptidylprolyl isomerase F (cyclophilin F) | 5.62E-08 | 1 | 0.76149608 | 1 |
| 204005_s_at | PAWR | PRKC, apoptosis, WT1, regulator | 5.62E-08 | 1 | 0.76149608 | 1 |
| 213010_at | PRKCDBP | protein kinase C, delta binding protein | 5.75E-08 | 1 | 0.003290551 | 1 |
| 219764_at | FZD10 | frizzled homolog 10 (Drosophila) | 5.81E-08 | 1 | 0.002658071 | 1 |
| 202145_at | LY6E | lymphocyte antigen 6 complex, locus E | 6.01E-08 | 1 | 0.007077712 | 1 |
| 202708_s_at | HIST2H2BE | histone cluster 2, H2be | 6.01E-08 | 1 | 0.100799433 | 1 |
| 201849_at | BNIP3 | BCL2/adenovirus E1B 19kDa interacting protein 3 | 6.24E-08 | 1 | 0.76149608 | 1 |
| 207169_x_at | DDR1 | discoidin domain receptor family, member 1 | 6.29E-08 | 1 | 0.107758282 | 1 |
| 226653_at | MARK1 | MAP/microtubule affinity-regulating kinase 1 | 6.32E-08 | 1 | 0.76149608 | 1 |
| 201309_x_at | C5orf13 | chromosome 5 open reading frame 13 | 6.72E-08 | 1 | 0.76149608 | 1 |
| 232541_at | EGFR | epidermal growth factor receptor (erythroblastic leukemia viral (v-erb-b) oncogene homolog, avian) | 6.86E-08 | 1 | 0.012280336 | 1 |
| 209924_at | CCL18 | chemokine (C-C motif) ligand 18 (pulmonary and activation-regulated) | 6.94E-08 | 0.988061971 | 0.002453104 | 1 |
| 200862_at | DHCR24 | 24-dehydrocholesterol reductase | 7.77E-08 | 1 | 0.596473358 | 1 |
| 210716_s_at | CLIP1 | CAP-GLY domain containing linker protein 1 | 7.92E-08 | 1 | 0.76149608 | 1 |
| 238909_at | S100A10 | S100 calcium binding protein A10 | 8.32E-08 | 1 | 0.76149608 | 1 |
| 231726_at | PCDHB14 | protocadherin beta 14 | 8.37E-08 | 1 | 0.76149608 | 1 |
| 205046_at | CENPE | centromere protein E, 312kDa | 8.38E-08 | 1 | 0.263740048 | 1 |
| 210609_s_at | TP53I3 | tumor protein p53 inducible protein 3 | 8.86E-08 | 1 | 0.00242885 | 1 |
| 203407_at | PPL | periplakin | 8.98E-08 | 1 | 0.76149608 | 1 |
| 204326_x_at | MT1X | metallothionein 1X | 9.03E-08 | 1 | 0.03898317 | 1 |
| 209099_x_at | JAG1 | jagged 1 (Alagille syndrome) | 9.04E-08 | 1 | 0.003302401 | 1 |
| 224511_s_at | TXNDC17 | thioredoxin domain containing 17 | 9.53E-08 | 1 | 0.006631361 | 1 |
| 203207_s_at | MTFR1 | mitochondrial fission regulator 1 | 1.01E-07 | 1 | 0.76149608 | 1 |
| 202887_s_at | DDIT4 | DNA-damage-inducible transcript 4 | 1.02E-07 | 0.835232341 | 0.76149608 | 0.902491484 |
| 212294_at | GNG12 | guanine nucleotide binding protein (G protein), gamma 12 | 1.03E-07 | 1 | 0.76149608 | 1 |
| 209772_s_at | CD24 | CD24 molecule | 1.03E-07 | 1 | 0.76149608 | 1 |
| 233587_s_at | SIPA1L2 | signal-induced proliferation-associated 1 like 2 | 1.04E-07 | 1 | 0.76149608 | 1 |
| 204298_s_at | LOX | lysyl oxidase | 1.08E-07 | 1 | 0.148327796 | 1 |
| 214581_x_at | TNFRSF21 | tumor necrosis factor receptor superfamily, member 21 | 1.13E-07 | 1 | 0.360462773 | 0.937222481 |
| 1555326_a_at | ADAM9 | ADAM metallopeptidase domain 9 (meltrin gamma) | 1.17E-07 | 1 | 0.76149608 | 1 |
| 201242_s_at | ATP1B1 | ATPase, Na+/K+ transporting, beta 1 polypeptide | 1.24E-07 | 1 | 0.006955275 | 1 |
| 204822_at | TTK | TTK protein kinase | 1.25E-07 | 1 | 0.391656899 | 1 |
| 222719_s_at | PDGFC | platelet derived growth factor C | 1.27E-07 | 1 | 0.76149608 | 1 |
| 205807_s_at | TUFT1 | tuftelin 1 | 1.28E-07 | 1 | 0.090772619 | 1 |
| 228729_at | CCNB1 | cyclin B1 | 1.29E-07 | 1 | 0.76149608 | 1 |
| 202236_s_at | SLC16A1 | solute carrier family 16, member 1 (monocarboxylic acid transporter 1) | 1.29E-07 | 1 | 0.76149608 | 1 |
| 229689_s_at | NA | NA | 1.31E-07 | 1 | 0.76149608 | 1 |
| 219496_at | ANKRD57 | ankyrin repeat domain 57 | 1.33E-07 | 1 | 0.384230819 | 1 |
| 212290_at | SLC7A1 | solute carrier family 7 (cationic amino acid transporter, y+ system), member 1 | 1.37E-07 | 1 | 0.76149608 | 1 |
| 218498_s_at | ERO1L | ERO1-like (S. cerevisiae) | 1.37E-07 | 1 | 0.76149608 | 0.915023204 |
| 214720_x_at | SEPT10 | septin 10 | 1.40E-07 | 1 | 0.76149608 | 1 |
| 200637_s_at | PTPRF | protein tyrosine phosphatase, receptor type, F | 1.40E-07 | 1 | 0.400684144 | 1 |
| 238755_at | LOC644943 | similar to peptidylglycine alpha-amidating monooxygenase COOH-terminal interactor | 1.44E-07 | 1 | 0.76149608 | 1 |
| 210052_s_at | TPX2 | TPX2, microtubule-associated, homolog (Xenopus laevis) | 1.47E-07 | 1 | 0.194233667 | 1 |
| 202488_s_at | FXYD3 | FXYD domain containing ion transport regulator 3 | 1.47E-07 | 1 | 0.76149608 | 1 |
| 204058_at | ME1 | malic enzyme 1, NADP(+)-dependent, cytosolic | 1.48E-07 | 1 | 0.76149608 | 1 |
| 214687_x_at | ALDOA | aldolase A, fructose-bisphosphate | 1.49E-07 | 1 | 0.040180894 | 1 |
| 214845_s_at | CALU | calumenin | 1.54E-07 | 1 | 0.085234636 | 1 |
| 223078_s_at | TMOD3 | tropomodulin 3 (ubiquitous) | 1.57E-07 | 1 | 0.76149608 | 1 |
| 209098_s_at | JAG1 | jagged 1 (Alagille syndrome) | 1.67E-07 | 1 | 0.029652562 | 1 |
| 234331_s_at | FAM84A | family with sequence similarity 84, member A | 1.68E-07 | 1 | 0.947821529 | 1 |
| 219901_at | FGD6 | FYVE, RhoGEF and PH domain containing 6 | 1.68E-07 | 1 | 0.76149608 | 1 |
| 242234_at | XAF1 | XIAP associated factor-1 | 1.73E-07 | 1 | 0.012995826 | 1 |
| 218252_at | CKAP2 | cytoskeleton associated protein 2 | 1.74E-07 | 1 | 0.76149608 | 1 |
| 225263_at | HS6ST1 | heparan sulfate 6-O-sulfotransferase 1 | 1.81E-07 | 1 | 0.477483834 | 1 |
| 205943_at | TDO2 | tryptophan 2,3-dioxygenase | 1.81E-07 | 1 | 1.04E-09 | 1 |
| 213358_at | KIAA0802 | KIAA0802 | 1.83E-07 | 1 | 0.255875252 | 1 |
| 225088_at | C16orf63 | chromosome 16 open reading frame 63 | 1.90E-07 | 1 | 0.76149608 | 0.996228181 |
| 209546_s_at | APOL1 | apolipoprotein L, 1 | 2.04E-07 | 1 | 0.754792854 | 1 |
| 212464_s_at | FN1 | fibronectin 1 | 2.10E-07 | 1 | 4.12E-06 | 1 |
| 203431_s_at | RICS | Rho GTPase-activating protein | 2.11E-07 | 1 | 0.522662242 | 1 |
| 224817_at | SH3PXD2A | SH3 and PX domains 2A | 2.22E-07 | 1 | 0.104053614 | 1 |
| 214007_s_at | TWF1 | twinfilin, actin-binding protein, homolog 1 (Drosophila) | 2.27E-07 | 1 | 0.76149608 | 1 |
| 219489_s_at | RHBDL2 | rhomboid, veinlet-like 2 (Drosophila) | 2.27E-07 | 1 | 0.066660595 | 1 |
| 237444_at | NA | NA | 2.32E-07 | 1 | 0.76149608 | 1 |
| 201995_at | EXT1 | exostoses (multiple) 1 | 2.36E-07 | 1 | 0.015699677 | 1 |
| 217901_at | DSG2 | desmoglein 2 | 2.37E-07 | 1 | 0.197738854 | 1 |
| 224995_at | SPIRE1 | spire homolog 1 (Drosophila) | 2.39E-07 | 1 | 0.76149608 | 1 |
| 211395_x_at | FCGR2C | Fc fragment of IgG, low affinity IIc, receptor for (CD32) | 2.48E-07 | 1 | 0.089061372 | 1 |
| 230372_at | NA | NA | 2.51E-07 | 1 | 0.76149608 | 1 |
| 203917_at | CXADR | coxsackie virus and adenovirus receptor | 2.75E-07 | 1 | 0.062047596 | 1 |
| 201852_x_at | COL3A1 | collagen, type III, alpha 1 (Ehlers-Danlos syndrome type IV, autosomal dominant) | 2.87E-07 | 1 | 2.94E-08 | 1 |
| 224973_at | FAM46A | family with sequence similarity 46, member A | 2.90E-07 | 1 | 0.76149608 | 0.988687126 |
| 220952_s_at | PLEKHA5 | pleckstrin homology domain containing, family A member 5 | 2.95E-07 | 1 | 0.333227412 | 1 |
| 201170_s_at | BHLHB2 | basic helix-loop-helix domain containing, class B, 2 | 2.97E-07 | 1 | 0.76149608 | 1 |
| 202286_s_at | TACSTD2 | tumor-associated calcium signal transducer 2 | 3.10E-07 | 1 | 0.004488528 | 1 |
| 209146_at | SC4MOL | sterol-C4-methyl oxidase-like | 3.10E-07 | 1 | 0.565395268 | 1 |
| 231867_at | ODZ2 | odz, odd Oz/ten-m homolog 2 (Drosophila) | 3.16E-07 | 1 | 1.65E-05 | 1 |
| 206685_at | HCG4 | HLA complex group 4 | 3.24E-07 | 1 | 0.76149608 | 0.81036415 |
| 217771_at | GOLM1 | golgi membrane protein 1 | 3.24E-07 | 1 | 0.002481078 | 1 |
| 1555460_a_at | SLC39A6 | solute carrier family 39 (zinc transporter), member 6 | 3.30E-07 | 1 | 0.76149608 | 1 |
| 205120_s_at | SGCB | sarcoglycan, beta (43kDa dystrophin-associated glycoprotein) | 3.42E-07 | 1 | 0.023889686 | 1 |
| 1555758_a_at | CDKN3 | cyclin-dependent kinase inhibitor 3 (CDK2-associated dual specificity phosphatase) | 3.55E-07 | 1 | 0.000365636 | 1 |
| 204006_s_at | FCGR3B | Fc fragment of IgG, low affinity IIIb, receptor (CD16b) | 3.56E-07 | 1 | 0.001109326 | 1 |
| 207023_x_at | KRT10 | keratin 10 (epidermolytic hyperkeratosis; keratosis palmaris et plantaris) | 3.80E-07 | 1 | 0.76149608 | 1 |
| 209301_at | CA2 | carbonic anhydrase II | 3.91E-07 | 1 | 0.76149608 | 1 |
| 228325_at | KIAA0146 | KIAA0146 | 4.03E-07 | 1 | 0.76149608 | 1 |
| 202952_s_at | ADAM12 | ADAM metallopeptidase domain 12 (meltrin alpha) | 4.15E-07 | 1 | 0.000338598 | 1 |
| 203304_at | BAMBI | BMP and activin membrane-bound inhibitor homolog (Xenopus laevis) | 4.16E-07 | 0.661447829 | 0.898158985 | 0.352165907 |
| 1554600_s_at | LMNA | lamin A/C | 4.23E-07 | 1 | 0.234444002 | 1 |
| 217196_s_at | CAMSAP1L1 | calmodulin regulated spectrin-associated protein 1-like 1 | 4.29E-07 | 1 | 0.632921563 | 1 |
| 235678_at | GM2A | GM2 ganglioside activator | 4.29E-07 | 1 | 0.235170379 | 1 |
| 203561_at | FCGR2A | Fc fragment of IgG, low affinity IIa, receptor (CD32) | 4.33E-07 | 1 | 0.039832022 | 1 |
| 204052_s_at | SFRP4 | secreted frizzled-related protein 4 | 4.33E-07 | 0.835232341 | 0.000158929 | 1 |
| 61734_at | RCN3 | reticulocalbin 3, EF-hand calcium binding domain | 4.52E-07 | 1 | 0.000734452 | 1 |
| 209442_x_at | ANK3 | ankyrin 3, node of Ranvier (ankyrin G) | 4.54E-07 | 1 | 0.800880333 | 0.900576773 |
| 223484_at | C15orf48 | chromosome 15 open reading frame 48 | 4.65E-07 | 1 | 0.00511483 | 1 |
| 208079_s_at | AURKA | aurora kinase A | 4.68E-07 | 1 | 0.147693304 | 1 |
| 210559_s_at | CDC2 | cell division cycle 2, G1 to S and G2 to M | 4.90E-07 | 1 | 0.76149608 | 1 |
| 226448_at | FAM89A | family with sequence similarity 89, member A | 5.00E-07 | 1 | 0.76149608 | 0.9252267 |
| 218980_at | FHOD3 | formin homology 2 domain containing 3 | 5.04E-07 | 1 | 0.76149608 | 1 |
| 231034_s_at | NHSL1 | NHS-like 1 | 5.07E-07 | 1 | 0.76149608 | 1 |
| 228378_at | C12orf29 | chromosome 12 open reading frame 29 | 5.11E-07 | 1 | 0.76149608 | 1 |
| 239586_at | FAM83A | family with sequence similarity 83, member A | 5.32E-07 | 1 | 0.76149608 | 1 |
| 202733_at | P4HA2 | procollagen-proline, 2-oxoglutarate 4-dioxygenase (proline 4-hydroxylase), alpha polypeptide II | 5.48E-07 | 1 | 0.021528821 | 1 |
| 1553764_a_at | JUB | jub, ajuba homolog (Xenopus laevis) | 5.79E-07 | 1 | 0.016985377 | 1 |
| 201645_at | TNC | tenascin C (hexabrachion) | 5.79E-07 | 1 | 0.018402664 | 1 |
| 203869_at | USP46 | ubiquitin specific peptidase 46 | 5.86E-07 | 1 | 0.781529535 | 1 |
| 204033_at | TRIP13 | thyroid hormone receptor interactor 13 | 5.86E-07 | 1 | 0.143837418 | 1 |
| 218162_at | OLFML3 | olfactomedin-like 3 | 5.86E-07 | 1 | 0.76149608 | 1 |
| 209569_x_at | D4S234E | DNA segment on chromosome 4 (unique) 234 expressed sequence | 5.91E-07 | 1 | 0.76149608 | 1 |
| 201189_s_at | ITPR3 | inositol 1,4,5-triphosphate receptor, type 3 | 6.07E-07 | 1 | 0.76149608 | 0.775525207 |
| 217475_s_at | LIMK2 | LIM domain kinase 2 | 6.09E-07 | 1 | 0.76149608 | 1 |
| 203725_at | GADD45A | growth arrest and DNA-damage-inducible, alpha | 6.17E-07 | 1 | 0.76149608 | 1 |
| 226075_at | SPSB1 | splA/ryanodine receptor domain and SOCS box containing 1 | 6.17E-07 | 1 | 0.001559489 | 1 |
| 213285_at | TMEM30B | transmembrane protein 30B | 6.28E-07 | 1 | 0.006793514 | 1 |
| 223125_s_at | C1orf21 | chromosome 1 open reading frame 21 | 6.38E-07 | 1 | 0.76149608 | 1 |
| 228523_at | NANOS1 | nanos homolog 1 (Drosophila) | 6.44E-07 | 1 | 0.76149608 | 1 |
| 200755_s_at | CALU | calumenin | 6.48E-07 | 1 | 0.000867127 | 1 |
| 210145_at | PLA2G4A | phospholipase A2, group IVA (cytosolic, calcium-dependent) | 6.48E-07 | 1 | 0.892003436 | 0.562291219 |
| 235456_at | NA | NA | 6.94E-07 | 1 | 0.000707047 | 0.757924823 |
| 226003_at | KIF21A | kinesin family member 21A | 6.95E-07 | 1 | 0.645985184 | 0.894950009 |
| 225853_at | GNPNAT1 | glucosamine-phosphate N-acetyltransferase 1 | 7.08E-07 | 1 | 0.321119124 | 0.989382694 |
| 226245_at | KCTD1 | potassium channel tetramerisation domain containing 1 | 7.46E-07 | 1 | 0.516279523 | 1 |
| 227376_at | GLI3 | GLI-Kruppel family member GLI3 (Greig cephalopolysyndactyly syndrome) | 7.52E-07 | 1 | 0.003059025 | 1 |
| 212992_at | AHNAK2 | AHNAK nucleoprotein 2 | 7.52E-07 | 1 | 0.080176981 | 1 |
| 220658_s_at | ARNTL2 | aryl hydrocarbon receptor nuclear translocator-like 2 | 7.63E-07 | 1 | 0.740258878 | 1 |
| 230518_at | MPZL2 | myelin protein zero-like 2 | 7.82E-07 | 1 | 0.76149608 | 1 |
| 227473_at | NA | NA | 7.88E-07 | 1 | 0.671367293 | 1 |
| 205401_at | AGPS | alkylglycerone phosphate synthase | 7.90E-07 | 1 | 0.76149608 | 1 |
| 223611_s_at | LNX1 | ligand of numb-protein X 1 | 8.50E-07 | 1 | 0.967480274 | 1 |
| 32094_at | CHST3 | carbohydrate (chondroitin 6) sulfotransferase 3 | 8.52E-07 | 1 | 0.129887825 | 1 |
| 211202_s_at | JARID1B | jumonji, AT rich interactive domain 1B | 8.69E-07 | 1 | 0.711912572 | 1 |
| 202403_s_at | COL1A2 | collagen, type I, alpha 2 | 8.77E-07 | 1 | 1.38E-06 | 1 |
| 40148_at | APBB2 | amyloid beta (A4) precursor protein-binding, family B, member 2 (Fe65-like) | 8.81E-07 | 1 | 0.048385009 | 0.856501108 |
| 202263_at | CYB5R1 | cytochrome b5 reductase 1 | 9.19E-07 | 1 | 0.139759633 | 1 |
| 204990_s_at | ITGB4 | integrin, beta 4 | 9.24E-07 | 1 | 0.005647179 | 1 |
| 219288_at | C3orf14 | chromosome 3 open reading frame 14 | 9.46E-07 | 1 | 0.76149608 | 1 |
| 227717_at | FLJ41603 | FLJ41603 protein | 9.62E-07 | 1 | 0.76149608 | 1 |
| 235295_at | NA | NA | 9.72E-07 | 1 | 0.517467477 | 1 |
| 210058_at | MAPK13 | mitogen-activated protein kinase 13 | 9.76E-07 | 1 | 0.76149608 | 1 |
| 205199_at | CA9 | carbonic anhydrase IX | 9.87E-07 | 0.895588923 | 0.76149608 | 0.925255384 |
| 238021_s_at | hCG_1815491 | hCG1815491 | 1.00E-06 | 1 | 0.76149608 | 1 |
| 204254_s_at | VDR | vitamin D (1,25- dihydroxyvitamin D3) receptor | 1.01E-06 | 1 | 0.339038167 | 1 |
| 215464_s_at | TAX1BP3 | Tax1 (human T-cell leukemia virus type I) binding protein 3 | 1.03E-06 | 1 | 0.032738804 | 1 |
| 201983_s_at | EGFR | epidermal growth factor receptor (erythroblastic leukemia viral (v-erb-b) oncogene homolog, avian) | 1.04E-06 | 1 | 0.034835551 | 1 |
| 236179_at | NA | NA | 1.09E-06 | 0.835232341 | 0.76149608 | 0.757924823 |
| 207574_s_at | GADD45B | growth arrest and DNA-damage-inducible, beta | 1.09E-06 | 0.944783333 | 0.76149608 | 0.919939295 |
| 229648_at | NA | NA | 1.11E-06 | 1 | 0.76149608 | 1 |
| 202357_s_at | CFB | complement factor B | 1.12E-06 | 1 | 0.048173733 | 1 |
| 224837_at | FOXP1 | forkhead box P1 | 1.12E-06 | 0.252878918 | 0.8854949 | 0.757924823 |
| 223461_at | TBC1D7 | TBC1 domain family, member 7 | 1.16E-06 | 1 | 0.76149608 | 1 |
| 201548_s_at | JARID1B | jumonji, AT rich interactive domain 1B | 1.18E-06 | 1 | 0.520646665 | 1 |
| 204616_at | UCHL3 | ubiquitin carboxyl-terminal esterase L3 (ubiquitin thiolesterase) | 1.19E-06 | 1 | 0.76149608 | 1 |
| 208867_s_at | CSNK1A1 | casein kinase 1, alpha 1 | 1.21E-06 | 1 | 0.76149608 | 1 |
| 201976_s_at | MYO10 | myosin X | 1.25E-06 | 1 | 0.275176827 | 1 |
| 203775_at | SLC25A13 | solute carrier family 25, member 13 (citrin) | 1.25E-06 | 1 | 0.220339426 | 1 |
| 217738_at | PBEF1 | pre-B-cell colony enhancing factor 1 | 1.34E-06 | 1 | 0.76149608 | 1 |
| 229068_at | CCT5 | chaperonin containing TCP1, subunit 5 (epsilon) | 1.34E-06 | 1 | 0.76149608 | 0.888305247 |
| 201489_at | PPIF | peptidylprolyl isomerase F (cyclophilin F) | 1.36E-06 | 1 | 0.377056066 | 1 |
| 200664_s_at | DNAJB1 | DnaJ (Hsp40) homolog, subfamily B, member 1 | 1.37E-06 | 1 | 0.76149608 | 1 |
| 230360_at | GLDN | gliomedin | 1.37E-06 | 0.835232341 | 0.76149608 | 1 |
| 203962_s_at | NEBL | nebulette | 1.40E-06 | 1 | 0.76149608 | 1 |
| 221958_s_at | GPR177 | G protein-coupled receptor 177 | 1.43E-06 | 1 | 0.76149608 | 1 |
| 209080_x_at | GLRX3 | glutaredoxin 3 | 1.43E-06 | 1 | 0.76149608 | 1 |
| 227314_at | ITGA2 | integrin, alpha 2 (CD49B, alpha 2 subunit of VLA-2 receptor) | 1.43E-06 | 1 | 0.048335098 | 1 |
| 224218_s_at | TRPS1 | trichorhinophalangeal syndrome I | 1.48E-06 | 1 | 0.76149608 | 1 |
| 201389_at | ITGA5 | integrin, alpha 5 (fibronectin receptor, alpha polypeptide) | 1.50E-06 | 1 | 5.99E-06 | 1 |
| 217841_s_at | PPME1 | protein phosphatase methylesterase 1 | 1.52E-06 | 1 | 0.76149608 | 1 |
| 205020_s_at | ARL4A | ADP-ribosylation factor-like 4A | 1.62E-06 | 1 | 0.81430664 | 0.972130292 |
| 205548_s_at | BTG3 | BTG family, member 3 | 1.62E-06 | 1 | 0.76149608 | 1 |
| 225878_at | KIF1B | kinesin family member 1B | 1.70E-06 | 1 | 0.76149608 | 1 |
| 206683_at | ZNF165 | zinc finger protein 165 | 1.74E-06 | 1 | 0.76149608 | 0.757924823 |
| 218180_s_at | EPS8L2 | EPS8-like 2 | 1.76E-06 | 1 | 0.76149608 | 1 |
| 221922_at | GPSM2 | G-protein signaling modulator 2 (AGS3-like, C. elegans) | 1.76E-06 | 1 | 0.76149608 | 1 |
| 202012_s_at | EXT2 | exostoses (multiple) 2 | 1.76E-06 | 1 | 0.270384589 | 1 |
| 221009_s_at | ANGPTL4 | angiopoietin-like 4 | 1.76E-06 | 1 | 0.76149608 | 1 |
| 225387_at | TSPAN5 | tetraspanin 5 | 1.79E-06 | 1 | 0.002156645 | 1 |
| 213478_at | KIAA1026 | kazrin | 1.84E-06 | 1 | 0.76149608 | 1 |
| 205579_at | HRH1 | histamine receptor H1 | 1.86E-06 | 1 | 0.65577958 | 1 |
| 237169_at | NA | NA | 1.95E-06 | 0.835232341 | 0.770248327 | 1 |
| 210633_x_at | KRT10 | keratin 10 (epidermolytic hyperkeratosis; keratosis palmaris et plantaris) | 2.06E-06 | 1 | 0.76149608 | 1 |
| 208779_x_at | DDR1 | discoidin domain receptor family, member 1 | 2.09E-06 | 1 | 0.412852447 | 1 |
| 237456_at | RYBP | RING1 and YY1 binding protein | 2.11E-06 | 0.835232341 | 1 | 0.757924823 |
| 224009_x_at | DHRS9 | dehydrogenase/reductase (SDR family) member 9 | 2.13E-06 | 1 | 0.76149608 | 0.772252929 |
| 210117_at | SPAG1 | sperm associated antigen 1 | 2.13E-06 | 1 | 0.76149608 | 1 |
| 210845_s_at | PLAUR | plasminogen activator, urokinase receptor | 2.14E-06 | 1 | 0.093651752 | 1 |
| 226021_at | RDH10 | retinol dehydrogenase 10 (all-trans) | 2.22E-06 | 1 | 0.1943454 | 1 |
| 215076_s_at | COL3A1 | collagen, type III, alpha 1 (Ehlers-Danlos syndrome type IV, autosomal dominant) | 2.24E-06 | 1 | 3.32E-07 | 1 |
| 223276_at | MST150 | MSTP150 | 2.44E-06 | 1 | 0.044501715 | 1 |
| 209203_s_at | BICD2 | bicaudal D homolog 2 (Drosophila) | 2.45E-06 | 1 | 0.837711613 | 1 |
| 200827_at | PLOD1 | procollagen-lysine 1, 2-oxoglutarate 5-dioxygenase 1 | 2.45E-06 | 1 | 0.108334445 | 1 |
| 201848_s_at | BNIP3 | BCL2/adenovirus E1B 19kDa interacting protein 3 | 2.48E-06 | 1 | 0.76149608 | 1 |
| 225687_at | FAM83D | family with sequence similarity 83, member D | 2.48E-06 | 1 | 0.406110356 | 1 |
| 1555736_a_at | AGTRAP | angiotensin II receptor-associated protein | 2.49E-06 | 1 | 0.037994885 | 1 |
| 204944_at | PTPRG | protein tyrosine phosphatase, receptor type, G | 2.52E-06 | 1 | 0.044501715 | 1 |
| 220559_at | EN1 | engrailed homeobox 1 | 2.52E-06 | 1 | 0.76149608 | 0.757924823 |
| 226029_at | VANGL2 | vang-like 2 (van gogh, Drosophila) | 2.54E-06 | 1 | 0.882982138 | 0.817026817 |
| 213125_at | OLFML2B | olfactomedin-like 2B | 2.54E-06 | 1 | 3.74E-07 | 1 |
| 224435_at | C10orf58 | chromosome 10 open reading frame 58 | 2.59E-06 | 1 | 0.76149608 | 1 |
| 242055_at | C6orf86 | chromosome 6 open reading frame 86 | 2.66E-06 | 1 | 0.975175106 | 0.757924823 |
| 228284_at | TLE1 | transducin-like enhancer of split 1 (E(sp1) homolog, Drosophila) | 2.67E-06 | 1 | 0.76430132 | 0.901839732 |
| 218804_at | TMEM16A | transmembrane protein 16A | 2.73E-06 | 1 | 0.004059754 | 1 |
| 59625_at | NOL3 | nucleolar protein 3 (apoptosis repressor with CARD domain) | 2.79E-06 | 1 | 0.76149608 | 0.984687978 |
| 209213_at | CBR1 | carbonyl reductase 1 | 2.82E-06 | 1 | 0.76149608 | 1 |
| 202967_at | GSTA4 | glutathione S-transferase A4 | 2.82E-06 | 1 | 0.789963208 | 1 |
| 209167_at | GPM6B | glycoprotein M6B | 2.93E-06 | 1 | 0.025946422 | 1 |
| 211071_s_at | MLLT11 | myeloid/lymphoid or mixed-lineage leukemia (trithorax homolog, Drosophila); translocated to, 11 | 3.01E-06 | 1 | 0.341573215 | 0.995242592 |
| 202289_s_at | TACC2 | transforming, acidic coiled-coil containing protein 2 | 3.03E-06 | 1 | 0.76149608 | 1 |
| 210513_s_at | VEGFA | vascular endothelial growth factor A | 3.09E-06 | 1 | 0.76149608 | 0.968788633 |
| 201195_s_at | SLC7A5 | solute carrier family 7 (cationic amino acid transporter, y+ system), member 5 | 3.09E-06 | 1 | 0.230231092 | 1 |
| 203638_s_at | FGFR2 | fibroblast growth factor receptor 2 (bacteria-expressed kinase, keratinocyte growth factor receptor, craniofacial dysostosis 1, Crouzon syndrome, Pfeiffer syndrome, Jackson-Weiss syndrome) | 3.13E-06 | 1 | 0.76149608 | 1 |
| 36936_at | TSTA3 | tissue specific transplantation antigen P35B | 3.13E-06 | 1 | 0.045000168 | 1 |
| 204255_s_at | VDR | vitamin D (1,25- dihydroxyvitamin D3) receptor | 3.15E-06 | 1 | 0.231443573 | 1 |
| 212719_at | PHLPP | PH domain and leucine rich repeat protein phosphatase | 3.30E-06 | 1 | 0.985266312 | 1 |
| 226609_at | DCBLD1 | discoidin, CUB and LCCL domain containing 1 | 3.30E-06 | 1 | 0.000679824 | 1 |
| 238692_at | BTBD11 | BTB (POZ) domain containing 11 | 3.47E-06 | 1 | 0.76149608 | 1 |
| 200924_s_at | SLC3A2 | solute carrier family 3 (activators of dibasic and neutral amino acid transport), member 2 | 3.52E-06 | 1 | 0.137113736 | 1 |
| 213279_at | DHRS1 | dehydrogenase/reductase (SDR family) member 1 | 3.53E-06 | 1 | 0.76149608 | 1 |
| 222668_at | KCTD15 | potassium channel tetramerisation domain containing 15 | 3.59E-06 | 1 | 0.76149608 | 1 |
| 202570_s_at | DLGAP4 | discs, large (Drosophila) homolog-associated protein 4 | 3.62E-06 | 1 | 0.46350749 | 0.997677394 |
| 202095_s_at | BIRC5 | baculoviral IAP repeat-containing 5 (survivin) | 3.85E-06 | 1 | 0.01042533 | 1 |
| 226311_at | NA | NA | 3.94E-06 | 1 | 0.000697106 | 1 |
| 209900_s_at | SLC16A1 | solute carrier family 16, member 1 (monocarboxylic acid transporter 1) | 3.95E-06 | 1 | 0.76149608 | 1 |
| 238439_at | ANKRD22 | ankyrin repeat domain 22 | 3.95E-06 | 1 | 0.300235316 | 1 |
| 206414_s_at | DDEF2 | development and differentiation enhancing factor 2 | 3.98E-06 | 1 | 0.37294367 | 1 |
| 217165_x_at | MT1F | metallothionein 1F | 3.98E-06 | 1 | 0.010075128 | 1 |
| 223854_at | PCDHB10 | protocadherin beta 10 | 4.02E-06 | 1 | 0.76149608 | 1 |
| 223839_s_at | SCD | stearoyl-CoA desaturase (delta-9-desaturase) | 4.07E-06 | 1 | 0.76149608 | 0.757924823 |
| 215411_s_at | TRAF3IP2 | TRAF3 interacting protein 2 | 4.08E-06 | 1 | 0.76149608 | 1 |
| 1557458_s_at | SHB | Src homology 2 domain containing adaptor protein B | 4.13E-06 | 1 | 0.76149608 | 1 |
| 213110_s_at | COL4A5 | collagen, type IV, alpha 5 (Alport syndrome) | 4.30E-06 | 1 | 0.008471567 | 0.943177284 |
| 203851_at | IGFBP6 | insulin-like growth factor binding protein 6 | 4.41E-06 | 1 | 0.76149608 | 0.956093291 |
| 224848_at | CDK6 | cyclin-dependent kinase 6 | 4.49E-06 | 1 | 0.76149608 | 1 |
| 204219_s_at | PSMC1 | proteasome (prosome, macropain) 26S subunit, ATPase, 1 | 4.57E-06 | 1 | 0.286767819 | 1 |
| 206857_s_at | FKBP1B | FK506 binding protein 1B, 12.6 kDa | 4.58E-06 | 1 | 0.76149608 | 1 |
| 219555_s_at | CENPN | centromere protein N | 4.62E-06 | 1 | 0.76149608 | 1 |
| 226847_at | FST | follistatin | 4.65E-06 | 1 | 0.76149608 | 1 |
| 225061_at | DNAJA4 | DnaJ (Hsp40) homolog, subfamily A, member 4 | 4.67E-06 | 1 | 0.76149608 | 0.9775758 |
| 204713_s_at | F5 | coagulation factor V (proaccelerin, labile factor) | 4.67E-06 | 0.835232341 | 0.76149608 | 0.757924823 |
| 226185_at | CDS1 | CDP-diacylglycerol synthase (phosphatidate cytidylyltransferase) 1 | 4.99E-06 | 1 | 0.564616942 | 1 |
| 219799_s_at | DHRS9 | dehydrogenase/reductase (SDR family) member 9 | 5.13E-06 | 1 | 0.76149608 | 0.757924823 |
| 1558214_s_at | CTNNA1 | catenin (cadherin-associated protein), alpha 1, 102kDa | 5.15E-06 | 1 | 0.76149608 | 1 |
| 202381_at | ADAM9 | ADAM metallopeptidase domain 9 (meltrin gamma) | 5.15E-06 | 1 | 0.305056885 | 1 |
| 209365_s_at | ECM1 | extracellular matrix protein 1 | 5.30E-06 | 1 | 0.408266689 | 1 |
| 65438_at | KIAA1609 | KIAA1609 | 5.54E-06 | 1 | 0.580964201 | 1 |
| 219631_at | LRP12 | low density lipoprotein-related protein 12 | 5.75E-06 | 1 | 0.185092012 | 1 |
| 212481_s_at | TPM4 | tropomyosin 4 | 5.99E-06 | 1 | 0.00565737 | 1 |
| 209679_s_at | LOC57228 | small trans-membrane and glycosylated protein | 6.06E-06 | 1 | 0.76149608 | 1 |
| 1552312_a_at | MFAP3 | microfibrillar-associated protein 3 | 6.10E-06 | 1 | 0.76149608 | 1 |
| 212763_at | CAMSAP1L1 | calmodulin regulated spectrin-associated protein 1-like 1 | 6.16E-06 | 1 | 0.76149608 | 1 |
| 219836_at | ZBED2 | zinc finger, BED-type containing 2 | 6.31E-06 | 1 | 0.76149608 | 1 |
| 225735_at | ANKRD50 | ankyrin repeat domain 50 | 6.46E-06 | 1 | 0.76149608 | 1 |
| 208651_x_at | CD24 | CD24 molecule | 6.49E-06 | 1 | 0.76149608 | 1 |
| 219087_at | ASPN | asporin | 6.51E-06 | 1 | 0.003550993 | 1 |
| 243367_at | NA | NA | 6.52E-06 | 1 | 0.001225146 | 1 |
| 209170_s_at | GPM6B | glycoprotein M6B | 6.53E-06 | 1 | 0.001125792 | 1 |
| 228293_at | DEPDC7 | DEP domain containing 7 | 6.65E-06 | 1 | 0.646960757 | 1 |
| 209863_s_at | TP63 | tumor protein p63 | 6.69E-06 | 1 | 0.148904435 | 1 |
| 222750_s_at | SRD5A2L | steroid 5 alpha-reductase 2-like | 7.02E-06 | 1 | 0.174556601 | 1 |
| 227812_at | TNFRSF19 | tumor necrosis factor receptor superfamily, member 19 | 7.07E-06 | 1 | 0.385023064 | 1 |
| 217430_x_at | COL1A1 | collagen, type I, alpha 1 | 7.10E-06 | 1 | 0.001588298 | 1 |
| 1557944_s_at | CTNND1 | catenin (cadherin-associated protein), delta 1 | 7.15E-06 | 1 | 0.76149608 | 1 |
| 209882_at | RIT1 | Ras-like without CAAX 1 | 7.15E-06 | 1 | 0.302838171 | 1 |
| 1555842_at | LOC284356 | hypothetical protein LOC284356 | 7.24E-06 | 1 | 0.76149608 | 0.8743931 |
| 225900_at | EXOC6B | exocyst complex component 6B | 7.30E-06 | 1 | 0.76149608 | 1 |
| 232165_at | EPPK1 | epiplakin 1 | 7.31E-06 | 1 | 0.76149608 | 1 |
| 235022_at | C18orf19 | chromosome 18 open reading frame 19 | 7.47E-06 | 1 | 0.76149608 | 1 |
| 205626_s_at | CALB1 | calbindin 1, 28kDa | 7.66E-06 | 0.835232341 | 0.76149608 | 0.757924823 |
| 213496_at | LPPR4 | plasticity related gene 1 | 7.66E-06 | 1 | 0.76149608 | 1 |
| 213943_at | TWIST1 | twist homolog 1 (acrocephalosyndactyly 3; Saethre-Chotzen syndrome) (Drosophila) | 7.75E-06 | 1 | 0.187783247 | 1 |
| 203790_s_at | HRSP12 | heat-responsive protein 12 | 8.14E-06 | 1 | 0.528820893 | 0.982902236 |
| 204059_s_at | ME1 | malic enzyme 1, NADP(+)-dependent, cytosolic | 8.18E-06 | 1 | 0.76149608 | 1 |
| 202376_at | SERPINA3 | serpin peptidase inhibitor, clade A (alpha-1 antiproteinase, antitrypsin), member 3 | 8.22E-06 | 1 | 0.76149608 | 0.795477772 |
| 203214_x_at | CDC2 | cell division cycle 2, G1 to S and G2 to M | 8.26E-06 | 1 | 0.76149608 | 1 |
| 227846_at | GPR176 | G protein-coupled receptor 176 | 8.37E-06 | 0.835232341 | 0.76149608 | 1 |
| 213134_x_at | BTG3 | BTG family, member 3 | 8.51E-06 | 1 | 0.76149608 | 1 |
| 202435_s_at | CYP1B1 | cytochrome P450, family 1, subfamily B, polypeptide 1 | 8.71E-06 | 0.122318244 | 0.001874799 | 1 |
| 232958_at | PYGL | phosphorylase, glycogen; liver (Hers disease, glycogen storage disease type VI) | 8.71E-06 | 1 | 0.268840717 | 1 |
| 201124_at | ITGB5 | integrin, beta 5 | 9.13E-06 | 1 | 0.76149608 | 1 |
| 227195_at | ZNF503 | zinc finger protein 503 | 9.53E-06 | 1 | 0.76149608 | 1 |
| 216092_s_at | SLC7A8 | solute carrier family 7 (cationic amino acid transporter, y+ system), member 8 | 9.54E-06 | 1 | 0.022844933 | 0.84803597 |
| 223279_s_at | UACA | uveal autoantigen with coiled-coil domains and ankyrin repeats | 9.61E-06 | 1 | 0.110741054 | 1 |
| 229055_at | GPR68 | G protein-coupled receptor 68 | 9.64E-06 | 1 | 0.068270391 | 1 |
| 203562_at | FEZ1 | fasciculation and elongation protein zeta 1 (zygin I) | 9.68E-06 | 1 | 7.79E-05 | 0.997253899 |
| 204306_s_at | CD151 | CD151 molecule (Raph blood group) | 9.94E-06 | 1 | 0.113999991 | 0.940839118 |
| 227034_at | ANKRD57 | ankyrin repeat domain 57 | 9.96E-06 | 1 | 0.76149608 | 1 |
| 205479_s_at | PLAU | plasminogen activator, urokinase | 9.97E-06 | 1 | 0.019112758 | 1 |
| 209969_s_at | STAT1 | signal transducer and activator of transcription 1, 91kDa | 1.01E-05 | 1 | 6.47E-05 | 1 |
| 211750_x_at | TUBA1C | tubulin, alpha 1c | 1.02E-05 | 1 | 0.168189306 | 1 |
| 227919_at | UCA1 | urothelial cancer associated 1 | 1.02E-05 | 1 | 0.76149608 | 0.949503011 |
| 235852_at | STON2 | stonin 2 | 1.03E-05 | 1 | 0.692012804 | 1 |
| 204580_at | MMP12 | matrix metallopeptidase 12 (macrophage elastase) | 1.11E-05 | 1 | 0.002906504 | 1 |
| 219773_at | NOX4 | NADPH oxidase 4 | 1.11E-05 | 1 | 0.003609514 | 1 |
| 213929_at | NA | NA | 1.12E-05 | 1 | 0.76149608 | 1 |
| 228667_at | AGPAT4 | 1-acylglycerol-3-phosphate O-acyltransferase 4 (lysophosphatidic acid acyltransferase, delta) | 1.13E-05 | 1 | 0.574986324 | 0.972249038 |
| 35666_at | SEMA3F | sema domain, immunoglobulin domain (Ig), short basic domain, secreted, (semaphorin) 3F | 1.14E-05 | 1 | 0.76149608 | 1 |
| 228617_at | XAF1 | XIAP associated factor-1 | 1.16E-05 | 1 | 0.34735822 | 1 |
| 215501_s_at | DUSP10 | dual specificity phosphatase 10 | 1.16E-05 | 1 | 0.76149608 | 0.851824295 |
| 203119_at | CCDC86 | coiled-coil domain containing 86 | 1.17E-05 | 1 | 0.76149608 | 1 |
| 238075_at | CHEK1 | CHK1 checkpoint homolog (S. pombe) | 1.17E-05 | 1 | 0.197591448 | 1 |
| 228654_at | SPIN4 | spindlin family, member 4 | 1.18E-05 | 1 | 0.76149608 | 1 |
| 214866_at | PLAUR | plasminogen activator, urokinase receptor | 1.21E-05 | 1 | 0.048173733 | 1 |
| 202311_s_at | COL1A1 | collagen, type I, alpha 1 | 1.23E-05 | 1 | 0.01590687 | 1 |
| 204641_at | NEK2 | NIMA (never in mitosis gene a)-related kinase 2 | 1.24E-05 | 1 | 0.152820286 | 1 |
| 209251_x_at | TUBA1C | tubulin, alpha 1c | 1.25E-05 | 1 | 0.097354187 | 1 |
| 203208_s_at | MTFR1 | mitochondrial fission regulator 1 | 1.26E-05 | 1 | 0.590790382 | 1 |
| 228575_at | IL20RB | interleukin 20 receptor beta | 1.26E-05 | 1 | 0.48469403 | 1 |
| 204517_at | PPIC | peptidylprolyl isomerase C (cyclophilin C) | 1.28E-05 | 1 | 0.082635738 | 1 |
| 209426_s_at | AMACR | alpha-methylacyl-CoA racemase | 1.29E-05 | 1 | 0.76149608 | 0.757924823 |
| 213006_at | CEBPD | CCAAT/enhancer binding protein (C/EBP), delta | 1.30E-05 | 1 | 0.76149608 | 1 |
| 222434_at | ENAH | enabled homolog (Drosophila) | 1.35E-05 | 1 | 0.76149608 | 0.970468279 |
| 210372_s_at | TPD52L1 | tumor protein D52-like 1 | 1.35E-05 | 1 | 0.76149608 | 1 |
| 222853_at | FLRT3 | fibronectin leucine rich transmembrane protein 3 | 1.38E-05 | 1 | 0.76149608 | 1 |
| 202613_at | CTPS | CTP synthase | 1.40E-05 | 1 | 0.76149608 | 1 |
| 221911_at | ETV1 | ets variant gene 1 | 1.42E-05 | 0.835232341 | 0.024412226 | 0.934189759 |
| 208456_s_at | RRAS2 | related RAS viral (r-ras) oncogene homolog 2 | 1.42E-05 | 1 | 0.76149608 | 1 |
| 231932_at | TRAF3IP3 | TRAF3 interacting protein 3 | 1.42E-05 | 1 | 0.76149608 | 1 |
| 218542_at | CEP55 | centrosomal protein 55kDa | 1.43E-05 | 1 | 0.412790354 | 1 |
| 232381_s_at | DNAH5 | dynein, axonemal, heavy chain 5 | 1.44E-05 | 0.964222271 | 0.76149608 | 0.808220888 |
| 209305_s_at | GADD45B | growth arrest and DNA-damage-inducible, beta | 1.45E-05 | 0.989594873 | 0.76149608 | 0.849638589 |
| 209212_s_at | KLF5 | Kruppel-like factor 5 (intestinal) | 1.47E-05 | 1 | 0.632075961 | 1 |
| 215243_s_at | GJB3 | gap junction protein, beta 3, 31kDa | 1.55E-05 | 1 | 0.586408931 | 1 |
| 227038_at | SGMS2 | sphingomyelin synthase 2 | 1.57E-05 | 1 | 0.168326572 | 1 |
| 205226_at | PDGFRL | platelet-derived growth factor receptor-like | 1.57E-05 | 0.571748151 | 0.175912647 | 1 |
| 214247_s_at | DKK3 | dickkopf homolog 3 (Xenopus laevis) | 1.58E-05 | 1 | 0.032660148 | 1 |
| 266_s_at | CD24 | CD24 molecule | 1.58E-05 | 1 | 0.383982959 | 1 |
| 202196_s_at | DKK3 | dickkopf homolog 3 (Xenopus laevis) | 1.63E-05 | 1 | 0.363571991 | 1 |
| 223544_at | TMEM79 | transmembrane protein 79 | 1.63E-05 | 1 | 0.76149608 | 1 |
| 209436_at | SPON1 | spondin 1, extracellular matrix protein | 1.65E-05 | 1 | 0.039209032 | 1 |
| 204348_s_at | AK3L1 | adenylate kinase 3-like 1 | 1.65E-05 | 1 | 0.565049929 | 1 |
| 209395_at | CHI3L1 | chitinase 3-like 1 (cartilage glycoprotein-39) | 1.65E-05 | 1 | 0.058730366 | 1 |
| 205403_at | IL1R2 | interleukin 1 receptor, type II | 1.66E-05 | 1 | 0.76149608 | 1 |
| 209448_at | HTATIP2 | HIV-1 Tat interactive protein 2, 30kDa | 1.66E-05 | 1 | 0.76149608 | 0.986252155 |
| 200628_s_at | WARS | tryptophanyl-tRNA synthetase | 1.66E-05 | 1 | 0.133547327 | 1 |
| 209276_s_at | GLRX | glutaredoxin (thioltransferase) | 1.66E-05 | 0.848071235 | 0.76149608 | 0.757924823 |
| 211964_at | COL4A2 | collagen, type IV, alpha 2 | 1.66E-05 | 1 | 0.000175821 | 1 |
| 203325_s_at | COL5A1 | collagen, type V, alpha 1 | 1.66E-05 | 1 | 0.017064491 | 1 |
| 204284_at | PPP1R3C | protein phosphatase 1, regulatory (inhibitor) subunit 3C | 1.67E-05 | 1 | 1 | 0.759079083 |
| 203788_s_at | SEMA3C | sema domain, immunoglobulin domain (Ig), short basic domain, secreted, (semaphorin) 3C | 1.67E-05 | 1 | 0.76149608 | 1 |
| 1557080_s_at | ITGBL1 | integrin, beta-like 1 (with EGF-like repeat domains) | 1.68E-05 | 0.91182849 | 0.006742485 | 1 |
| 37145_at | GNLY | granulysin | 1.75E-05 | 0.835232341 | 0.76149608 | 1 |
| 232458_at | COL3A1 | collagen, type III, alpha 1 (Ehlers-Danlos syndrome type IV, autosomal dominant) | 1.75E-05 | 1 | 2.44E-35 | 1 |
| 201108_s_at | THBS1 | thrombospondin 1 | 1.76E-05 | 1 | 0.036800146 | 1 |
| 57588_at | SLC24A3 | solute carrier family 24 (sodium/potassium/calcium exchanger), member 3 | 1.77E-05 | 1 | 0.76149608 | 1 |
| 202458_at | PRSS23 | protease, serine, 23 | 1.84E-05 | 1 | 0.024540993 | 1 |
| 210367_s_at | PTGES | prostaglandin E synthase | 1.90E-05 | 1 | 0.872414258 | 1 |
| 227955_s_at | NA | NA | 1.93E-05 | 1 | 0.76149608 | 0.936308143 |
| 219962_at | ACE2 | angiotensin I converting enzyme (peptidyl-dipeptidase A) 2 | 1.97E-05 | 1 | 0.162217756 | 0.859603807 |
| 203184_at | FBN2 | fibrillin 2 (congenital contractural arachnodactyly) | 2.01E-05 | 1 | 0.76149608 | 0.970469778 |
| 227046_at | SLC39A11 | solute carrier family 39 (metal ion transporter), member 11 | 2.03E-05 | 1 | 0.011028651 | 1 |
| 222608_s_at | ANLN | anillin, actin binding protein | 2.03E-05 | 1 | 0.000842757 | 1 |
| 202888_s_at | ANPEP | alanyl (membrane) aminopeptidase (aminopeptidase N, aminopeptidase M, microsomal aminopeptidase, CD13, p150) | 2.04E-05 | 1 | 9.17E-14 | 1 |
| 210797_s_at | OASL | 2'-5'-oligoadenylate synthetase-like | 2.06E-05 | 1 | 0.76149608 | 1 |
| 223541_at | HAS3 | hyaluronan synthase 3 | 2.09E-05 | 1 | 0.76149608 | 1 |
| 226651_at | HOMER1 | homer homolog 1 (Drosophila) | 2.10E-05 | 1 | 0.76149608 | 1 |
| 224813_at | WASL | Wiskott-Aldrich syndrome-like | 2.14E-05 | 1 | 0.76149608 | 1 |
| 216607_s_at | CYP51A1 | cytochrome P450, family 51, subfamily A, polypeptide 1 | 2.16E-05 | 1 | 0.76149608 | 1 |
| 208228_s_at | FGFR2 | fibroblast growth factor receptor 2 (bacteria-expressed kinase, keratinocyte growth factor receptor, craniofacial dysostosis 1, Crouzon syndrome, Pfeiffer syndrome, Jackson-Weiss syndrome) | 2.22E-05 | 1 | 0.76149608 | 1 |
| 225750_at | NA | NA | 2.24E-05 | 1 | 0.447975661 | 0.757924823 |
| 201469_s_at | SHC1 | SHC (Src homology 2 domain containing) transforming protein 1 | 2.26E-05 | 1 | 0.76149608 | 0.968414646 |
| 203971_at | SLC31A1 | solute carrier family 31 (copper transporters), member 1 | 2.26E-05 | 1 | 0.113999991 | 1 |
| 239196_at | ANKRD22 | ankyrin repeat domain 22 | 2.27E-05 | 1 | 0.39233294 | 1 |
| 208636_at | ACTN1 | actinin, alpha 1 | 2.27E-05 | 1 | 8.90E-05 | 1 |
| 213349_at | TMCC1 | transmembrane and coiled-coil domain family 1 | 2.30E-05 | 1 | 0.030247283 | 1 |
| 1557948_at | PHLDB3 | pleckstrin homology-like domain, family B, member 3 | 2.30E-05 | 1 | 0.786682387 | 0.973174593 |
| 218585_s_at | DTL | denticleless homolog (Drosophila) | 2.30E-05 | 1 | 0.025827267 | 1 |
| 236034_at | NA | NA | 2.33E-05 | 1 | 0.76149608 | 0.757924823 |
| 214587_at | COL8A1 | collagen, type VIII, alpha 1 | 2.33E-05 | 1 | 0.76149608 | 0.868056699 |
| 202468_s_at | CTNNAL1 | catenin (cadherin-associated protein), alpha-like 1 | 2.33E-05 | 0.835232341 | 0.76149608 | 1 |
| 218454_at | FLJ22662 | hypothetical protein FLJ22662 | 2.34E-05 | 1 | 0.219446431 | 1 |
| 221803_s_at | NRBF2 | nuclear receptor binding factor 2 | 2.34E-05 | 1 | 0.76149608 | 1 |
| 1553535_a_at | RANGAP1 | Ran GTPase activating protein 1 | 2.36E-05 | 1 | 0.76149608 | 1 |
| 205241_at | SCO2 | SCO cytochrome oxidase deficient homolog 2 (yeast) | 2.36E-05 | 1 | 0.028859874 | 1 |
| 202833_s_at | SERPINA1 | serpin peptidase inhibitor, clade A (alpha-1 antiproteinase, antitrypsin), member 1 | 2.40E-05 | 1 | 2.20E-09 | 1 |
| 227522_at | CMBL | carboxymethylenebutenolidase homolog (Pseudomonas) | 2.48E-05 | 1 | 0.762682287 | 0.900770745 |
| 221698_s_at | CLEC7A | C-type lectin domain family 7, member A | 2.50E-05 | 1 | 0.145134467 | 1 |
| 228950_s_at | GPR177 | G protein-coupled receptor 177 | 2.52E-05 | 1 | 0.76149608 | 1 |
| 218705_s_at | SNX24 | sorting nexin 24 | 2.57E-05 | 1 | 0.76149608 | 0.972387066 |
| 210511_s_at | INHBA | inhibin, beta A | 2.61E-05 | 1 | 0.032738804 | 1 |
| 204971_at | CSTA | cystatin A (stefin A) | 2.65E-05 | 1 | 0.399852027 | 1 |
| 201951_at | ALCAM | activated leukocyte cell adhesion molecule | 2.65E-05 | 0.835232341 | 0.428474443 | 0.757924823 |
| 219911_s_at | SLCO4A1 | solute carrier organic anion transporter family, member 4A1 | 2.67E-05 | 1 | 0.76149608 | 0.992031629 |
| 213342_at | YAP1 | Yes-associated protein 1, 65kDa | 2.69E-05 | 1 | 0.76149608 | 1 |
| 1569003_at | TMEM49 | transmembrane protein 49 | 2.73E-05 | 1 | 0.018605656 | 1 |
| 203827_at | WIPI1 | WD repeat domain, phosphoinositide interacting 1 | 2.74E-05 | 1 | 0.003839639 | 0.960638052 |
| 211148_s_at | ANGPT2 | angiopoietin 2 | 2.78E-05 | 1 | 0.76149608 | 0.831966145 |
| 225330_at | IGF1R | insulin-like growth factor 1 receptor | 2.79E-05 | 1 | 0.76149608 | 1 |
| 208393_s_at | RAD50 | RAD50 homolog (S. cerevisiae) | 2.86E-05 | 1 | 0.76149608 | 0.858587744 |
| 227069_at | CUTL1 | cut-like 1, CCAAT displacement protein (Drosophila) | 2.89E-05 | 1 | 0.76149608 | 0.812772882 |
| 41037_at | TEAD4 | TEA domain family member 4 | 2.91E-05 | 1 | 0.074411873 | 1 |
| 215253_s_at | RCAN1 | regulator of calcineurin 1 | 2.95E-05 | 1 | 0.76149608 | 1 |
| 205034_at | CCNE2 | cyclin E2 | 2.97E-05 | 1 | 0.148769225 | 1 |
| 203423_at | RBP1 | retinol binding protein 1, cellular | 2.99E-05 | 1 | 0.015663845 | 1 |
| 206932_at | CH25H | cholesterol 25-hydroxylase | 3.03E-05 | 1 | 0.76149608 | 1 |
| 219727_at | DUOX2 | dual oxidase 2 | 3.04E-05 | 1 | 0.76149608 | 0.876702975 |
| 217497_at | ECGF1 | endothelial cell growth factor 1 (platelet-derived) | 3.06E-05 | 1 | 0.141681673 | 1 |
| 201647_s_at | SCARB2 | scavenger receptor class B, member 2 | 3.06E-05 | 1 | 0.76149608 | 1 |
| 1554018_at | GPNMB | glycoprotein (transmembrane) nmb | 3.06E-05 | 1 | 0.048173733 | 1 |
| 228158_at | LOC645166 | similar to lymphocyte-specific protein 1 isoform 1 | 3.10E-05 | 1 | 0.031158289 | 1 |
| 201328_at | ETS2 | v-ets erythroblastosis virus E26 oncogene homolog 2 (avian) | 3.27E-05 | 1 | 0.963594019 | 1 |
| 202934_at | HK2 | hexokinase 2 | 3.28E-05 | 1 | 0.76149608 | 1 |
| 222258_s_at | SH3BP4 | SH3-domain binding protein 4 | 3.30E-05 | 1 | 0.005724783 | 1 |
| 202935_s_at | SOX9 | SRY (sex determining region Y)-box 9 (campomelic dysplasia, autosomal sex-reversal) | 3.37E-05 | 1 | 0.445819065 | 1 |
| 201714_at | TUBG1 | tubulin, gamma 1 | 3.37E-05 | 1 | 0.565395268 | 1 |
| 208937_s_at | ID1 | inhibitor of DNA binding 1, dominant negative helix-loop-helix protein | 3.37E-05 | 1 | 0.76149608 | 0.856447919 |
| 218326_s_at | LGR4 | leucine-rich repeat-containing G protein-coupled receptor 4 | 3.46E-05 | 1 | 0.76149608 | 1 |
| 230369_at | GPR161 | G protein-coupled receptor 161 | 3.52E-05 | 1 | 0.76149608 | 1 |
| 241864_x_at | NA | NA | 3.59E-05 | 0.835232341 | 0.76149608 | 0.946080948 |
| 219321_at | MPP5 | membrane protein, palmitoylated 5 (MAGUK p55 subfamily member 5) | 3.64E-05 | 1 | 0.76149608 | 0.999016625 |
| 1553105_s_at | DSG2 | desmoglein 2 | 3.64E-05 | 1 | 0.527979496 | 1 |
| 209921_at | SLC7A11 | solute carrier family 7, (cationic amino acid transporter, y+ system) member 11 | 3.67E-05 | 1 | 0.76149608 | 1 |
| 201505_at | LAMB1 | laminin, beta 1 | 3.75E-05 | 1 | 0.023997584 | 1 |
| 225955_at | LOC653506 | similar to meteorin, glial cell differentiation regulator-like | 3.80E-05 | 1 | 0.339667587 | 1 |
| 216836_s_at | ERBB2 | v-erb-b2 erythroblastic leukemia viral oncogene homolog 2, neuro/glioblastoma derived oncogene homolog (avian) | 3.82E-05 | 1 | 0.76149608 | 1 |
| 213252_at | SH3PXD2A | SH3 and PX domains 2A | 3.85E-05 | 1 | 0.092834235 | 1 |
| 226799_at | NA | NA | 3.88E-05 | 0.977650186 | 0.76149608 | 0.812782942 |
| 221610_s_at | STAP2 | signal transducing adaptor family member 2 | 3.91E-05 | 1 | 0.477789132 | 1 |
| 204924_at | TLR2 | toll-like receptor 2 | 4.10E-05 | 1 | 0.000248257 | 1 |
| 204068_at | STK3 | serine/threonine kinase 3 (STE20 homolog, yeast) | 4.11E-05 | 1 | 0.76149608 | 1 |
| 202718_at | IGFBP2 | insulin-like growth factor binding protein 2, 36kDa | 4.11E-05 | 1 | 0.76149608 | 0.812525914 |
| 206354_at | SLCO1B3 | solute carrier organic anion transporter family, member 1B3 | 4.11E-05 | 1 | 0.76149608 | 0.757924823 |
| 218729_at | LXN | latexin | 4.13E-05 | 0.835232341 | 0.00017855 | 1 |
| 220428_at | CD207 | CD207 molecule, langerin | 4.34E-05 | 1 | 0.76149608 | 1 |
| 202036_s_at | SFRP1 | secreted frizzled-related protein 1 | 4.37E-05 | 1 | 1 | 1 |
| 229097_at | DIAPH3 | diaphanous homolog 3 (Drosophila) | 4.45E-05 | 1 | 0.450944622 | 1 |
| 203963_at | CA12 | carbonic anhydrase XII | 4.47E-05 | 1 | 0.090042628 | 1 |
| 226026_at | DIRC2 | disrupted in renal carcinoma 2 | 4.54E-05 | 1 | 0.005282701 | 1 |
| 230381_at | C1orf186 | chromosome 1 open reading frame 186 | 4.54E-05 | 1 | 0.494117176 | 0.868387839 |
| 1556361_s_at | ANKRD13C | ankyrin repeat domain 13C | 4.64E-05 | 1 | 0.76149608 | 1 |
| 230263_s_at | DOCK5 | dedicator of cytokinesis 5 | 4.66E-05 | 1 | 0.142269812 | 1 |
| 205404_at | HSD11B1 | hydroxysteroid (11-beta) dehydrogenase 1 | 4.68E-05 | 0.936069922 | 0.004148068 | 1 |
| 1556194_a_at | NA | NA | 4.69E-05 | 1 | 0.76149608 | 1 |
| 203585_at | ZNF185 | zinc finger protein 185 (LIM domain) | 4.75E-05 | 1 | 0.586408931 | 1 |
| 214104_at | GPR161 | G protein-coupled receptor 161 | 4.80E-05 | 1 | 0.148769225 | 1 |
| 206300_s_at | PTHLH | parathyroid hormone-like hormone | 4.81E-05 | 1 | 0.76149608 | 1 |
| 207172_s_at | CDH11 | cadherin 11, type 2, OB-cadherin (osteoblast) | 4.85E-05 | 0.882933446 | 0.76149608 | 1 |
| 225078_at | EMP2 | epithelial membrane protein 2 | 5.03E-05 | 1 | 0.794239036 | 1 |
| 242722_at | LMO7 | LIM domain 7 | 5.09E-05 | 1 | 0.76149608 | 0.993559565 |
| 209360_s_at | RUNX1 | runt-related transcription factor 1 (acute myeloid leukemia 1; aml1 oncogene) | 5.12E-05 | 1 | 0.001484659 | 1 |
| 226120_at | TTC8 | tetratricopeptide repeat domain 8 | 5.14E-05 | 1 | 0.007698621 | 1 |
| 225686_at | FAM33A | family with sequence similarity 33, member A | 5.16E-05 | 1 | 0.353090725 | 1 |
| 219411_at | ELMO3 | engulfment and cell motility 3 | 5.24E-05 | 1 | 0.76149608 | 0.944582815 |
| 211343_s_at | COL13A1 | collagen, type XIII, alpha 1 | 5.25E-05 | 1 | 0.76149608 | 1 |
| 218188_s_at | TIMM13 | translocase of inner mitochondrial membrane 13 homolog (yeast) | 5.27E-05 | 1 | 0.76149608 | 1 |
| 209570_s_at | D4S234E | DNA segment on chromosome 4 (unique) 234 expressed sequence | 5.38E-05 | 1 | 0.76149608 | 1 |
| 218847_at | IGF2BP2 | insulin-like growth factor 2 mRNA binding protein 2 | 5.38E-05 | 1 | 0.008507053 | 1 |
| 200756_x_at | CALU | calumenin | 5.39E-05 | 1 | 0.386841025 | 1 |
| 220800_s_at | TMOD3 | tropomodulin 3 (ubiquitous) | 5.51E-05 | 1 | 0.76149608 | 1 |
| 216251_s_at | TTLL12 | tubulin tyrosine ligase-like family, member 12 | 5.53E-05 | 1 | 0.76149608 | 1 |
| 1554333_at | DNAJA4 | DnaJ (Hsp40) homolog, subfamily A, member 4 | 5.59E-05 | 1 | 0.76149608 | 1 |
| 204017_at | KDELR3 | KDEL (Lys-Asp-Glu-Leu) endoplasmic reticulum protein retention receptor 3 | 5.59E-05 | 1 | 0.019282144 | 1 |
| 202235_at | SLC16A1 | solute carrier family 16, member 1 (monocarboxylic acid transporter 1) | 5.65E-05 | 1 | 0.76149608 | 1 |
| 204962_s_at | CENPA | centromere protein A | 5.65E-05 | 1 | 0.097354187 | 1 |
| 228347_at | SIX1 | SIX homeobox 1 | 5.69E-05 | 0.953932238 | 0.76149608 | 0.912512954 |
| 226390_at | STARD4 | StAR-related lipid transfer (START) domain containing 4 | 5.69E-05 | 1 | 0.76149608 | 0.757924823 |
| 217014_s_at | AZGP1 | alpha-2-glycoprotein 1, zinc-binding | 5.69E-05 | 1 | 0.76149608 | 1 |
| 1555756_a_at | CLEC7A | C-type lectin domain family 7, member A | 5.69E-05 | 1 | 0.000368506 | 1 |
| 222774_s_at | NETO2 | neuropilin (NRP) and tolloid (TLL)-like 2 | 5.81E-05 | 1 | 0.76149608 | 1 |
| 202870_s_at | CDC20 | cell division cycle 20 homolog (S. cerevisiae) | 5.82E-05 | 1 | 0.317164219 | 1 |
| 228933_at | NHS | Nance-Horan syndrome (congenital cataracts and dental anomalies) | 5.87E-05 | 1 | 0.247461033 | 1 |
| 204165_at | WASF1 | WAS protein family, member 1 | 5.95E-05 | 1 | 0.76149608 | 1 |
| 213365_at | EXOD1 | exonuclease domain containing 1 | 5.96E-05 | 1 | 0.76149608 | 1 |
| 203081_at | CTNNBIP1 | catenin, beta interacting protein 1 | 5.96E-05 | 1 | 0.76149608 | 1 |
| AFFX-HUMISGF3A/M97935_5_at | STAT1 | signal transducer and activator of transcription 1, 91kDa | 5.97E-05 | 1 | 0.613175927 | 1 |
| 203641_s_at | COBLL1 | COBL-like 1 | 6.00E-05 | 1 | 0.794152061 | 0.084958107 |
| 1552277_a_at | C9orf30 | chromosome 9 open reading frame 30 | 6.01E-05 | 1 | 0.012204662 | 1 |
| 221047_s_at | MARK1 | MAP/microtubule affinity-regulating kinase 1 | 6.06E-05 | 1 | 0.76149608 | 1 |
| 229144_at | KIAA1026 | kazrin | 6.16E-05 | 1 | 0.76149608 | 1 |
| 200635_s_at | PTPRF | protein tyrosine phosphatase, receptor type, F | 6.21E-05 | 1 | 0.76149608 | 1 |
| 224774_s_at | NAV1 | neuron navigator 1 | 6.27E-05 | 1 | 0.76149608 | 0.848600492 |
| 209154_at | TAX1BP3 | Tax1 (human T-cell leukemia virus type I) binding protein 3 | 6.27E-05 | 1 | 0.002906504 | 1 |
| 221563_at | DUSP10 | dual specificity phosphatase 10 | 6.27E-05 | 1 | 0.76149608 | 0.829671851 |
| 203160_s_at | RNF8 | ring finger protein 8 | 6.30E-05 | 1 | 0.76149608 | 1 |
| 202367_at | CUTL1 | cut-like 1, CCAAT displacement protein (Drosophila) | 6.49E-05 | 1 | 0.76149608 | 0.795203844 |
| 218434_s_at | AACS | acetoacetyl-CoA synthetase | 6.53E-05 | 1 | 0.76149608 | 0.957918697 |
| 225941_at | EIF4E3 | eukaryotic translation initiation factor 4E family member 3 | 6.54E-05 | 0.247080807 | 0.989687339 | 0.757924823 |
| 1567107_s_at | TPM4 | tropomyosin 4 | 6.56E-05 | 1 | 0.153573041 | 1 |
| 209372_x_at | TUBB2A | tubulin, beta 2A | 6.58E-05 | 1 | 0.76149608 | 1 |
| 202096_s_at | TSPO | translocator protein (18kDa) | 6.62E-05 | 1 | 0.477789132 | 1 |
| 202267_at | LAMC2 | laminin, gamma 2 | 6.65E-05 | 1 | 0.03357627 | 1 |
| 203798_s_at | VSNL1 | visinin-like 1 | 6.79E-05 | 1 | 0.76149608 | 1 |
| 222689_at | PHCA | phytoceramidase, alkaline | 6.79E-05 | 1 | 0.384230819 | 0.918593466 |
| 210813_s_at | XRCC4 | X-ray repair complementing defective repair in Chinese hamster cells 4 | 6.82E-05 | 1 | 0.76149608 | 0.925292309 |
| 203395_s_at | HES1 | hairy and enhancer of split 1, (Drosophila) | 7.04E-05 | 1 | 0.76149608 | 1 |
| 37005_at | NBL1 | neuroblastoma, suppression of tumorigenicity 1 | 7.04E-05 | 1 | 0.089506761 | 1 |
| 208837_at | TMED3 | transmembrane emp24 protein transport domain containing 3 | 7.05E-05 | 1 | 0.423476255 | 1 |
| 225285_at | BCAT1 | branched chain aminotransferase 1, cytosolic | 7.35E-05 | 1 | 0.001840796 | 1 |
| 222654_at | IMPAD1 | inositol monophosphatase domain containing 1 | 7.44E-05 | 1 | 0.002468293 | 1 |
| 220066_at | NOD2 | nucleotide-binding oligomerization domain containing 2 | 7.44E-05 | 1 | 0.76149608 | 1 |
| 212295_s_at | SLC7A1 | solute carrier family 7 (cationic amino acid transporter, y+ system), member 1 | 7.48E-05 | 1 | 0.76149608 | 1 |
| 212472_at | MICAL2 | microtubule associated monoxygenase, calponin and LIM domain containing 2 | 7.48E-05 | 1 | 0.000315308 | 1 |
| 229975_at | NA | NA | 7.61E-05 | 1 | 0.76149608 | 0.982363874 |
| 212702_s_at | BICD2 | bicaudal D homolog 2 (Drosophila) | 7.61E-05 | 1 | 0.76149608 | 1 |
| 235182_at | C20orf82 | chromosome 20 open reading frame 82 | 7.61E-05 | 1 | 0.76149608 | 1 |
| 213273_at | ODZ4 | odz, odd Oz/ten-m homolog 4 (Drosophila) | 7.86E-05 | 1 | 0.148413581 | 1 |
| 238996_x_at | ALDOA | aldolase A, fructose-bisphosphate | 7.89E-05 | 1 | 0.496206411 | 0.93658341 |
| 206392_s_at | RARRES1 | retinoic acid receptor responder (tazarotene induced) 1 | 8.21E-05 | 0.890869413 | 0.00020597 | 1 |
| 226517_at | BCAT1 | branched chain aminotransferase 1, cytosolic | 8.28E-05 | 1 | 0.000350988 | 1 |
| 219872_at | C4orf18 | chromosome 4 open reading frame 18 | 8.33E-05 | 0.939822938 | 0.76149608 | 1 |
| 204715_at | PANX1 | pannexin 1 | 8.41E-05 | 1 | 0.233475959 | 1 |
| 37408_at | MRC2 | mannose receptor, C type 2 | 8.43E-05 | 1 | 0.034184842 | 1 |
| 238017_at | RDHE2 | epidermal retinal dehydrogenase 2 | 8.63E-05 | 1 | 0.76149608 | 1 |
| 217999_s_at | PHLDA1 | pleckstrin homology-like domain, family A, member 1 | 8.78E-05 | 1 | 0.76149608 | 1 |
| 200731_s_at | PTP4A1 | protein tyrosine phosphatase type IVA, member 1 | 8.78E-05 | 1 | 0.76149608 | 1 |
| 202728_s_at | LTBP1 | latent transforming growth factor beta binding protein 1 | 8.79E-05 | 1 | 0.76149608 | 1 |
| 202454_s_at | ERBB3 | v-erb-b2 erythroblastic leukemia viral oncogene homolog 3 (avian) | 8.83E-05 | 1 | 0.76149608 | 1 |
| 226184_at | FMNL2 | formin-like 2 | 8.90E-05 | 1 | 0.76149608 | 1 |
| 226285_at | CAPRIN1 | cell cycle associated protein 1 | 9.11E-05 | 1 | 0.76149608 | 1 |
| 218198_at | DHX32 | DEAH (Asp-Glu-Ala-His) box polypeptide 32 | 9.21E-05 | 1 | 0.76149608 | 1 |
| 211756_at | PTHLH | parathyroid hormone-like hormone | 9.22E-05 | 1 | 0.384230819 | 1 |
| 218384_at | CARHSP1 | calcium regulated heat stable protein 1, 24kDa | 9.25E-05 | 1 | 0.240592565 | 0.945206566 |
| 210128_s_at | LTB4R | leukotriene B4 receptor | 9.34E-05 | 1 | 0.76149608 | 1 |
| 218099_at | TEX2 | testis expressed 2 | 9.34E-05 | 1 | 0.76149608 | 1 |
| 1555137_a_at | FGD6 | FYVE, RhoGEF and PH domain containing 6 | 9.36E-05 | 1 | 0.76149608 | 1 |
| 201888_s_at | IL13RA1 | interleukin 13 receptor, alpha 1 | 9.38E-05 | 1 | 0.574986324 | 1 |
| 231918_s_at | GFM2 | G elongation factor, mitochondrial 2 | 9.42E-05 | 1 | 0.76149608 | 0.887660788 |
| 212338_at | MYO1D | myosin ID | 9.44E-05 | 1 | 0.170862903 | 1 |
| 230424_at | C5orf13 | chromosome 5 open reading frame 13 | 9.52E-05 | 1 | 0.76149608 | 0.902411021 |
| 212104_s_at | RBM9 | RNA binding motif protein 9 | 9.52E-05 | 1 | 0.76149608 | 1 |
| 202706_s_at | UMPS | uridine monophosphate synthetase (orotate phosphoribosyl transferase and orotidine-5'-decarboxylase) | 9.54E-05 | 1 | 0.76149608 | 1 |
| 202779_s_at | UBE2S | ubiquitin-conjugating enzyme E2S | 9.54E-05 | 1 | 0.385169465 | 1 |
| 212009_s_at | STIP1 | stress-induced-phosphoprotein 1 (Hsp70/Hsp90-organizing protein) | 9.66E-05 | 1 | 0.76149608 | 1 |
| 234351_x_at | TRPS1 | trichorhinophalangeal syndrome I | 9.67E-05 | 1 | 0.784395205 | 1 |
| 200766_at | CTSD | cathepsin D | 9.71E-05 | 1 | 0.230231092 | 0.99321009 |
| 222883_at | C1orf163 | chromosome 1 open reading frame 163 | 9.76E-05 | 1 | 0.76149608 | 1 |
| 202219_at | SLC6A8 | solute carrier family 6 (neurotransmitter transporter, creatine), member 8 | 9.82E-05 | 1 | 0.112627909 | 0.969637474 |
| 221872_at | RARRES1 | retinoic acid receptor responder (tazarotene induced) 1 | 9.82E-05 | 0.999709631 | 1.20E-05 | 1 |
| 226733_at | PFKFB2 | 6-phosphofructo-2-kinase/fructose-2,6-biphosphatase 2 | 9.99E-05 | 1 | 0.76149608 | 0.757924823 |
| 213562_s_at | SQLE | squalene epoxidase | 9.99E-05 | 1 | 0.76149608 | 1 |
| 219209_at | IFIH1 | interferon induced with helicase C domain 1 | 0.000100556 | 1 | 0.76149608 | 1 |
| 223363_at | PSMG3 | proteasome (prosome, macropain) assembly chaperone 3 | 0.000100563 | 1 | 0.502888911 | 0.820722195 |
| 219976_at | HOOK1 | hook homolog 1 (Drosophila) | 0.000100563 | 1 | 0.76149608 | 0.757924823 |
| 225303_at | KIRREL | kin of IRRE like (Drosophila) | 0.000101074 | 1 | 0.032738804 | 1 |
| 214059_at | IFI44 | interferon-induced protein 44 | 0.000102176 | 1 | 0.155046283 | 1 |
| 220253_s_at | LRP12 | low density lipoprotein-related protein 12 | 0.000102207 | 1 | 0.76149608 | 1 |
| 217975_at | WBP5 | WW domain binding protein 5 | 0.00010251 | 1 | 0.076468662 | 1 |
| 212365_at | MYO1B | myosin IB | 0.000104226 | 1 | 0.046786461 | 1 |
| 117_at | HSPA6 | heat shock 70kDa protein 6 (HSP70B') | 0.000105432 | 1 | 0.113972283 | 1 |
| 223393_s_at | TSHZ3 | teashirt zinc finger homeobox 3 | 0.000105584 | 1 | 0.289611659 | 1 |
| 200665_s_at | SPARC | secreted protein, acidic, cysteine-rich (osteonectin) | 0.000106205 | 1 | 7.32E-05 | 1 |
| 214672_at | TTLL5 | tubulin tyrosine ligase-like family, member 5 | 0.000106657 | 1 | 0.477277045 | 0.99641378 |
| 219795_at | SLC6A14 | solute carrier family 6 (amino acid transporter), member 14 | 0.000106657 | 1 | 0.76149608 | 1 |
| 207076_s_at | ASS1 | argininosuccinate synthetase 1 | 0.000108139 | 1 | 0.76149608 | 1 |
| 226863_at | FAM110C | family with sequence similarity 110, member C | 0.000108236 | 1 | 0.718269333 | 1 |
| 1558924_s_at | CLIP1 | CAP-GLY domain containing linker protein 1 | 0.000109303 | 1 | 0.76149608 | 1 |
| 219532_at | ELOVL4 | elongation of very long chain fatty acids (FEN1/Elo2, SUR4/Elo3, yeast)-like 4 | 0.000110539 | 1 | 0.76149608 | 1 |
| 203736_s_at | PPFIBP1 | PTPRF interacting protein, binding protein 1 (liprin beta 1) | 0.000113584 | 1 | 0.76149608 | 1 |
| 200824_at | GSTP1 | glutathione S-transferase pi | 0.000113584 | 1 | 0.116128623 | 1 |
| 244780_at | SGPP2 | sphingosine-1-phosphate phosphotase 2 | 0.000113584 | 1 | 0.76149608 | 0.986362843 |
| 231807_at | KIAA1217 | KIAA1217 | 0.000114238 | 1 | 0.003747848 | 1 |
| 225481_at | FRMD6 | FERM domain containing 6 | 0.00011743 | 1 | 0.005195688 | 1 |
| 1555274_a_at | SELI | selenoprotein I | 0.000117646 | 1 | 0.185092012 | 1 |
| 1564520_s_at | PRMT5 | protein arginine methyltransferase 5 | 0.0001179 | 1 | 0.176386573 | 1 |
| 234973_at | SLC38A5 | solute carrier family 38, member 5 | 0.000118492 | 1 | 0.273355849 | 0.963228249 |
| 203573_s_at | RABGGTA | Rab geranylgeranyltransferase, alpha subunit | 0.000118492 | 1 | 0.76149608 | 0.850910514 |
| 1556097_at | NA | NA | 0.000119548 | 1 | 0.76149608 | 0.757924823 |
| 205394_at | CHEK1 | CHK1 checkpoint homolog (S. pombe) | 0.000119976 | 1 | 0.724792549 | 1 |
| 209784_s_at | JAG2 | jagged 2 | 0.000122847 | 1 | 0.266301628 | 1 |
| 240673_at | NA | NA | 0.000125103 | 1 | 0.857540288 | 0.757924823 |
| 241342_at | TMEM65 | transmembrane protein 65 | 0.000126041 | 1 | 0.76149608 | 1 |
| 203650_at | PROCR | protein C receptor, endothelial (EPCR) | 0.000126041 | 1 | 0.76149608 | 1 |
| 216615_s_at | HTR3A | 5-hydroxytryptamine (serotonin) receptor 3A | 0.000126418 | 1 | 0.781162263 | 0.757924823 |
| 213423_x_at | TUSC3 | tumor suppressor candidate 3 | 0.000127348 | 1 | 0.76149608 | 0.956193273 |
| 201594_s_at | PPP4R1 | protein phosphatase 4, regulatory subunit 1 | 0.000127666 | 1 | 0.03990468 | 1 |
| 205822_s_at | HMGCS1 | 3-hydroxy-3-methylglutaryl-Coenzyme A synthase 1 (soluble) | 0.000128054 | 1 | 0.76149608 | 0.762842309 |
| 213076_at | ITPKC | inositol 1,4,5-trisphosphate 3-kinase C | 0.000131081 | 1 | 0.76149608 | 1 |
| 225059_at | AGTRAP | angiotensin II receptor-associated protein | 0.000132856 | 1 | 0.210294203 | 1 |
| 205567_at | CHST1 | carbohydrate (keratan sulfate Gal-6) sulfotransferase 1 | 0.000134435 | 0.915806115 | 0.76149608 | 0.857718386 |
| 224770_s_at | NAV1 | neuron navigator 1 | 0.000135965 | 0.96553354 | 0.76149608 | 0.827614261 |
| 221779_at | MICALL1 | MICAL-like 1 | 0.000136016 | 1 | 0.76149608 | 1 |
| 209633_at | PPP2R3A | protein phosphatase 2 (formerly 2A), regulatory subunit B'', alpha | 0.000137022 | 1 | 0.694884139 | 1 |
| 222849_s_at | SCRN3 | secernin 3 | 0.000137674 | 1 | 0.76149608 | 1 |
| 241813_at | MBD1 | methyl-CpG binding domain protein 1 | 0.000139293 | 1 | 0.762018632 | 0.978780674 |
| 225866_at | BXDC1 | brix domain containing 1 | 0.000139649 | 1 | 0.76149608 | 1 |
| 228698_at | PINX1 | PIN2-interacting protein 1 | 0.000142587 | 1 | 0.781184764 | 1 |
| 209632_at | PPP2R3A | protein phosphatase 2 (formerly 2A), regulatory subunit B'', alpha | 0.000142679 | 1 | 0.346820034 | 1 |
| 1554334_a_at | DNAJA4 | DnaJ (Hsp40) homolog, subfamily A, member 4 | 0.000142679 | 1 | 0.76149608 | 1 |
| 218145_at | TRIB3 | tribbles homolog 3 (Drosophila) | 0.000144394 | 1 | 0.76149608 | 0.757924823 |
| 210959_s_at | SRD5A1 | steroid-5-alpha-reductase, alpha polypeptide 1 (3-oxo-5 alpha-steroid delta 4-dehydrogenase alpha 1) | 0.000145731 | 1 | 0.76149608 | 1 |
| 214696_at | MGC14376 | hypothetical protein MGC14376 | 0.000145874 | 1 | 0.175912647 | 1 |
| 204533_at | CXCL10 | chemokine (C-X-C motif) ligand 10 | 0.000148337 | 1 | 0.081465958 | 1 |
| 201415_at | GSS | glutathione synthetase | 0.000148883 | 1 | 0.172762397 | 1 |
| 217127_at | CTH | cystathionase (cystathionine gamma-lyase) | 0.000149866 | 0.717304309 | 0.76149608 | 0.21820839 |
| 210880_s_at | EFS | embryonal Fyn-associated substrate | 0.000149866 | 1 | 0.76149608 | 1 |
| 210735_s_at | CA12 | carbonic anhydrase XII | 0.000150339 | 1 | 0.332615075 | 1 |
| 201666_at | TIMP1 | TIMP metallopeptidase inhibitor 1 | 0.000151331 | 1 | 8.81E-07 | 1 |
| 219630_at | PDZK1IP1 | PDZK1 interacting protein 1 | 0.000151501 | 1 | 0.147863132 | 1 |
| 227100_at | B3GALTL | beta 1,3-galactosyltransferase-like | 0.000151706 | 1 | 0.813139982 | 0.956727356 |
| 223392_s_at | TSHZ3 | teashirt zinc finger homeobox 3 | 0.000153393 | 1 | 0.045000168 | 1 |
| 218330_s_at | NAV2 | neuron navigator 2 | 0.000154001 | 1 | 0.016768313 | 0.874856852 |
| 214431_at | GMPS | guanine monphosphate synthetase | 0.00015509 | 1 | 0.232796818 | 1 |
| 224952_at | TANC2 | tetratricopeptide repeat, ankyrin repeat and coiled-coil containing 2 | 0.000157899 | 1 | 0.175935143 | 1 |
| 231688_at | NA | NA | 0.000158256 | 0.852005589 | 0.76149608 | 0.984366054 |
| 218849_s_at | PPP1R13L | protein phosphatase 1, regulatory (inhibitor) subunit 13 like | 0.00015866 | 1 | 0.76149608 | 1 |
| 209369_at | ANXA3 | annexin A3 | 0.000159051 | 1 | 0.314991708 | 1 |
| 205442_at | MFAP3L | microfibrillar-associated protein 3-like | 0.000163802 | 0.97119291 | 0.876156535 | 1 |
| 211966_at | COL4A2 | collagen, type IV, alpha 2 | 0.00016562 | 1 | 0.000491521 | 1 |
| 211905_s_at | ITGB4 | integrin, beta 4 | 0.000167628 | 1 | 0.176386573 | 0.983236285 |
| 215489_x_at | HOMER3 | homer homolog 3 (Drosophila) | 0.000170943 | 1 | 0.070758245 | 1 |
| 213927_at | MAP3K9 | mitogen-activated protein kinase kinase kinase 9 | 0.00017109 | 1 | 0.76149608 | 0.973765542 |
| 233404_at | SH3PXD2A | SH3 and PX domains 2A | 0.000171127 | 1 | 0.084779216 | 0.757924823 |
| 1552257_a_at | TTLL12 | tubulin tyrosine ligase-like family, member 12 | 0.000171257 | 1 | 0.76149608 | 1 |
| 202122_s_at | M6PRBP1 | mannose-6-phosphate receptor binding protein 1 | 0.00017229 | 1 | 0.76149608 | 1 |
| 227443_at | C9orf150 | chromosome 9 open reading frame 150 | 0.000172343 | 1 | 0.76149608 | 1 |
| 222449_at | TMEPAI | transmembrane, prostate androgen induced RNA | 0.000173146 | 1 | 0.000153555 | 1 |
| 235296_at | EIF5A2 | eukaryotic translation initiation factor 5A2 | 0.000173869 | 0.835232341 | 0.76149608 | 1 |
| 222958_s_at | DEPDC1 | DEP domain containing 1 | 0.000173869 | 1 | 0.650811153 | 1 |
| 219100_at | OBFC1 | oligonucleotide/oligosaccharide-binding fold containing 1 | 0.000174798 | 1 | 0.774847373 | 0.85849001 |
| 226726_at | MBOAT2 | membrane bound O-acyltransferase domain containing 2 | 0.000177244 | 1 | 0.76149608 | 0.991971932 |
| 212432_at | GRPEL1 | GrpE-like 1, mitochondrial (E. coli) | 0.000177244 | 1 | 0.408823752 | 0.994604312 |
| 208950_s_at | ALDH7A1 | aldehyde dehydrogenase 7 family, member A1 | 0.000177543 | 1 | 0.76149608 | 1 |
| 204537_s_at | GABRE | gamma-aminobutyric acid (GABA) A receptor, epsilon | 0.00017797 | 1 | 0.007130093 | 0.943727312 |
| 228367_at | ALPK2 | alpha-kinase 2 | 0.000180881 | 0.794964101 | 0.76149608 | 0.757924823 |
| 208951_at | ALDH7A1 | aldehyde dehydrogenase 7 family, member A1 | 0.000183164 | 1 | 0.76149608 | 1 |
| 243754_at | NA | NA | 0.00018361 | 1 | 0.76149608 | 0.913176656 |
| 214657_s_at | TncRNA | trophoblast-derived noncoding RNA | 0.000184136 | 1 | 0.76149608 | 1 |
| 204750_s_at | DSC2 | desmocollin 2 | 0.000187634 | 1 | 0.76149608 | 1 |
| 207245_at | UGT2B17 | UDP glucuronosyltransferase 2 family, polypeptide B17 | 0.00019101 | 0.835232341 | 0.76149608 | 0.757924823 |
| 1554252_a_at | LASS3 | LAG1 homolog, ceramide synthase 3 | 0.000191493 | 1 | 0.76149608 | 1 |
| 205625_s_at | CALB1 | calbindin 1, 28kDa | 0.000192308 | 0.935091353 | 0.76149608 | 0.757924823 |
| 160020_at | MMP14 | matrix metallopeptidase 14 (membrane-inserted) | 0.000193176 | 1 | 0.088051307 | 1 |
| 204345_at | COL16A1 | collagen, type XVI, alpha 1 | 0.000194729 | 1 | 0.129986448 | 1 |
| 225201_s_at | MRPL14 | mitochondrial ribosomal protein L14 | 0.00019503 | 1 | 0.395135474 | 1 |
| 206884_s_at | SCEL | sciellin | 0.000195524 | 1 | 0.76149608 | 1 |
| 206785_s_at | KLRC2 | killer cell lectin-like receptor subfamily C, member 2 | 0.000197209 | 0.724071678 | 0.76149608 | 1 |
| 218717_s_at | LEPREL1 | leprecan-like 1 | 0.000197865 | 1 | 0.76149608 | 1 |
| 204709_s_at | KIF23 | kinesin family member 23 | 0.000197865 | 1 | 0.76149608 | 1 |
| 203968_s_at | CDC6 | cell division cycle 6 homolog (S. cerevisiae) | 0.000197865 | 1 | 0.76149608 | 1 |
| 236826_at | C9orf52 | chromosome 9 open reading frame 52 | 0.000198037 | 1 | 0.76149608 | 0.964965863 |
| 235635_at | ARHGAP5 | Rho GTPase activating protein 5 | 0.000202523 | 0.835232341 | 0.891516714 | 0.757924823 |
| 217050_at | EPAG | early lymphoid activation protein | 0.000204927 | 1 | 0.76149608 | 0.785293372 |
| 1553973_a_at | SPINK6 | serine peptidase inhibitor, Kazal type 6 | 0.000205546 | 1 | 0.76149608 | 0.84677964 |
| 206499_s_at | RCC1 | regulator of chromosome condensation 1 | 0.000208914 | 1 | 0.76149608 | 1 |
| 207357_s_at | GALNT10 | UDP-N-acetyl-alpha-D-galactosamine:polypeptide N-acetylgalactosaminyltransferase 10 (GalNAc-T10) | 0.000209594 | 0.835232341 | 0.76149608 | 0.836165299 |
| 217755_at | HN1 | hematological and neurological expressed 1 | 0.000210407 | 1 | 0.000238283 | 1 |
| 1569157_s_at | LOC162993 | hypothetical protein LOC162993 | 0.000211667 | 1 | 0.836957384 | 0.870022633 |
| 225252_at | SRXN1 | sulfiredoxin 1 homolog (S. cerevisiae) | 0.000214558 | 1 | 0.542196764 | 1 |
| 205204_at | NMB | neuromedin B | 0.000227562 | 1 | 0.76149608 | 0.757924823 |
| 205206_at | KAL1 | Kallmann syndrome 1 sequence | 0.000230101 | 1 | 0.039543779 | 1 |
| 222830_at | GRHL1 | grainyhead-like 1 (Drosophila) | 0.000231636 | 1 | 0.76149608 | 1 |
| 203344_s_at | RBBP8 | retinoblastoma binding protein 8 | 0.000231636 | 1 | 0.426642155 | 1 |
| 225851_at | FNTB | farnesyltransferase, CAAX box, beta | 0.000233684 | 1 | 0.76149608 | 0.960378447 |
| 219933_at | GLRX2 | glutaredoxin 2 | 0.000234517 | 1 | 0.748560472 | 1 |
| 1555728_a_at | MS4A4A | membrane-spanning 4-domains, subfamily A, member 4 | 0.000236149 | 0.835232341 | 0.048385009 | 1 |
| 211150_s_at | DLAT | dihydrolipoamide S-acetyltransferase (E2 component of pyruvate dehydrogenase complex) | 0.000236149 | 1 | 0.76149608 | 1 |
| 228865_at | C1orf116 | chromosome 1 open reading frame 116 | 0.000240985 | 1 | 0.76149608 | 1 |
| 231897_at | LTB4DH | leukotriene B4 12-hydroxydehydrogenase | 0.000240985 | 1 | 0.76149608 | 1 |
| 242133_s_at | LOC654342 | similar to lymphocyte-specific protein 1 | 0.000242185 | 0.909738655 | 0.033166685 | 1 |
| 204035_at | SCG2 | secretogranin II (chromogranin C) | 0.000247331 | 0.849562728 | 0.76149608 | 0.940513281 |
| 225589_at | SH3RF1 | SH3 domain containing ring finger 1 | 0.000247441 | 1 | 0.76149608 | 0.891522087 |
| 234700_s_at | RNASE7 | ribonuclease, RNase A family, 7 | 0.000249658 | 1 | 0.340107785 | 1 |
| 216379_x_at | CD24 | CD24 molecule | 0.000252702 | 1 | 0.540969241 | 1 |
| 49077_at | PPME1 | protein phosphatase methylesterase 1 | 0.000255485 | 1 | 0.76149608 | 1 |
| 1554220_a_at | SMOX | spermine oxidase | 0.000256477 | 1 | 0.399852027 | 0.757924823 |
| 217904_s_at | BACE1 | beta-site APP-cleaving enzyme 1 | 0.000257611 | 1 | 0.76149608 | 1 |
| 52255_s_at | COL5A3 | collagen, type V, alpha 3 | 0.000260077 | 1 | 0.04043938 | 1 |
| 224771_at | NAV1 | neuron navigator 1 | 0.000260244 | 1 | 0.76149608 | 0.825132086 |
| 235089_at | FBXL20 | F-box and leucine-rich repeat protein 20 | 0.000263796 | 1 | 0.76149608 | 0.906260527 |
| 1559606_at | GBP6 | guanylate binding protein family, member 6 | 0.000263796 | 1 | 0.76149608 | 1 |
| 208977_x_at | TUBB2C | tubulin, beta 2C | 0.000273686 | 1 | 0.243474743 | 1 |
| 225946_at | RASSF8 | Ras association (RalGDS/AF-6) domain family 8 | 0.000276699 | 0.974639167 | 0.800505965 | 1 |
| 208864_s_at | TXN | thioredoxin | 0.000278161 | 1 | 0.552557583 | 1 |
| 1564064_a_at | ATP11B | ATPase, Class VI, type 11B | 0.000281764 | 1 | 0.76149608 | 0.863254404 |
| 213994_s_at | SPON1 | spondin 1, extracellular matrix protein | 0.00028247 | 1 | 0.032738804 | 1 |
| 203649_s_at | PLA2G2A | phospholipase A2, group IIA (platelets, synovial fluid) | 0.000283675 | 0.887918081 | 0.066878896 | 1 |
| 225436_at | FAM108C1 | family with sequence similarity 108, member C1 | 0.000284234 | 1 | 0.780187235 | 1 |
| 218295_s_at | NUP50 | nucleoporin 50kDa | 0.00028616 | 1 | 0.76149608 | 0.975839012 |
| 200832_s_at | SCD | stearoyl-CoA desaturase (delta-9-desaturase) | 0.000288852 | 1 | 0.76149608 | 0.757924823 |
| 210163_at | CXCL11 | chemokine (C-X-C motif) ligand 11 | 0.000288852 | 1 | 0.76149608 | 1 |
| 218782_s_at | ATAD2 | ATPase family, AAA domain containing 2 | 0.000288852 | 1 | 0.76149608 | 1 |
| 224396_s_at | ASPN | asporin | 0.000288852 | 1 | 0.76149608 | 1 |
| 202917_s_at | S100A8 | S100 calcium binding protein A8 | 0.000288852 | 1 | 0.130665765 | 1 |
| 209709_s_at | HMMR | hyaluronan-mediated motility receptor (RHAMM) | 0.000289897 | 1 | 0.213454447 | 1 |
| 238932_at | TSC22D2 | TSC22 domain family, member 2 | 0.000290309 | 0.835232341 | 0.76149608 | 0.757924823 |
| 200820_at | PSMD8 | proteasome (prosome, macropain) 26S subunit, non-ATPase, 8 | 0.00029671 | 1 | 0.403190595 | 1 |
| 229400_at | HOXD10 | homeobox D10 | 0.00029878 | 1 | 0.097354187 | 1 |
| 212021_s_at | MKI67 | antigen identified by monoclonal antibody Ki-67 | 0.00030028 | 1 | 0.145134467 | 1 |
| 205729_at | OSMR | oncostatin M receptor | 0.000302396 | 1 | 0.76149608 | 1 |
| 238712_at | FOXP1 | forkhead box P1 | 0.000303271 | 0.835232341 | 0.909189133 | 0.757924823 |
| 203440_at | CDH2 | cadherin 2, type 1, N-cadherin (neuronal) | 0.000304129 | 0.835232341 | 0.76149608 | 0.757924823 |
| 228565_at | KIAA1804 | mixed lineage kinase 4 | 0.000304392 | 1 | 0.76149608 | 0.757924823 |
| 206335_at | GALNS | galactosamine (N-acetyl)-6-sulfate sulfatase (Morquio syndrome, mucopolysaccharidosis type IVA) | 0.000306665 | 0.890846099 | 0.409700523 | 0.816414038 |
| 223599_at | TRIM6 | tripartite motif-containing 6 | 0.000306665 | 1 | 0.76149608 | 1 |
| 209408_at | KIF2C | kinesin family member 2C | 0.000306665 | 1 | 0.477483834 | 1 |
| 205227_at | IL1RAP | interleukin 1 receptor accessory protein | 0.00030747 | 1 | 0.76149608 | 1 |
| 210059_s_at | MAPK13 | mitogen-activated protein kinase 13 | 0.000313829 | 1 | 0.76149608 | 1 |
| 201059_at | CTTN | cortactin | 0.000314562 | 1 | 0.108795341 | 1 |
| 203328_x_at | IDE | insulin-degrading enzyme | 0.000314643 | 1 | 0.76149608 | 1 |
| 201876_at | PON2 | paraoxonase 2 | 0.000315597 | 1 | 0.378225088 | 1 |
| 209605_at | TST | thiosulfate sulfurtransferase (rhodanese) | 0.000315597 | 1 | 0.76149608 | 1 |
| 226213_at | ERBB3 | v-erb-b2 erythroblastic leukemia viral oncogene homolog 3 (avian) | 0.00032133 | 1 | 0.479541646 | 1 |
| 243386_at | NA | NA | 0.000325284 | 1 | 0.765300849 | 1 |
| 226858_at | CSNK1E | casein kinase 1, epsilon | 0.000325329 | 1 | 0.76149608 | 1 |
| 219201_s_at | TWSG1 | twisted gastrulation homolog 1 (Drosophila) | 0.00032706 | 1 | 0.76149608 | 1 |
| 203554_x_at | PTTG1 | pituitary tumor-transforming 1 | 0.000329942 | 1 | 0.284257791 | 1 |
| 204415_at | IFI6 | interferon, alpha-inducible protein 6 | 0.00033059 | 1 | 0.76149608 | 1 |
| 1552627_a_at | ARHGAP5 | Rho GTPase activating protein 5 | 0.000334165 | 1 | 0.76149608 | 0.909029268 |
| 224484_s_at | BRMS1L | breast cancer metastasis-suppressor 1-like | 0.000334321 | 1 | 0.76149608 | 1 |
| 240467_at | NA | NA | 0.000336339 | 1 | 0.646245723 | 1 |
| 217839_at | TFG | TRK-fused gene | 0.000341105 | 1 | 0.76149608 | 1 |
| 202444_s_at | ERLIN1 | ER lipid raft associated 1 | 0.000341239 | 1 | 0.76149608 | 1 |
| 202005_at | ST14 | suppression of tumorigenicity 14 (colon carcinoma) | 0.00034356 | 1 | 0.209415851 | 1 |
| 239719_at | CD109 | CD109 molecule | 0.000343826 | 1 | 0.76149608 | 1 |
| 203323_at | CAV2 | caveolin 2 | 0.000350672 | 1 | 0.76149608 | 1 |
| 213418_at | HSPA6 | heat shock 70kDa protein 6 (HSP70B') | 0.0003543 | 1 | 0.001687361 | 1 |
| 208850_s_at | THY1 | Thy-1 cell surface antigen | 0.000356453 | 1 | 0.000231207 | 1 |
| 211709_s_at | CLEC11A | C-type lectin domain family 11, member A | 0.000358815 | 1 | 0.075093919 | 1 |
| 244258_at | NA | NA | 0.00036312 | 1 | 0.217822663 | 0.885849607 |
| 205941_s_at | COL10A1 | collagen, type X, alpha 1(Schmid metaphyseal chondrodysplasia) | 0.000363684 | 1 | 0.007850265 | 1 |
| 241763_s_at | FBXO32 | F-box protein 32 | 0.000369117 | 1 | 0.761434168 | 0.871920225 |
| 200629_at | WARS | tryptophanyl-tRNA synthetase | 0.000369452 | 1 | 0.000893834 | 1 |
| 209424_s_at | AMACR | alpha-methylacyl-CoA racemase | 0.000371735 | 1 | 0.76149608 | 0.757924823 |
| 201329_s_at | ETS2 | v-ets erythroblastosis virus E26 oncogene homolog 2 (avian) | 0.000375208 | 1 | 0.76149608 | 1 |
| 205260_s_at | ACYP1 | acylphosphatase 1, erythrocyte (common) type | 0.000375297 | 1 | 0.784555009 | 0.757924823 |
| 216985_s_at | STX3 | syntaxin 3 | 0.000375925 | 1 | 0.76149608 | 1 |
| 1555759_a_at | CCL5 | chemokine (C-C motif) ligand 5 | 0.000376075 | 0.519529761 | 0.76149608 | 1 |
| 230076_at | NA | NA | 0.000381277 | 1 | 0.76149608 | 0.983335048 |
| 242719_at | NA | NA | 0.000388037 | 0.929555628 | 0.776728541 | 0.769614007 |
| 221489_s_at | SPRY4 | sprouty homolog 4 (Drosophila) | 0.000395999 | 1 | 0.76149608 | 0.838898772 |
| 51192_at | SSH3 | slingshot homolog 3 (Drosophila) | 0.000397536 | 1 | 0.76149608 | 1 |
| 232591_s_at | TMEM30A | transmembrane protein 30A | 0.00040923 | 1 | 0.76149608 | 1 |
| 218898_at | FAM57A | family with sequence similarity 57, member A | 0.000415837 | 1 | 0.250648663 | 1 |
| 204518_s_at | PPIC | peptidylprolyl isomerase C (cyclophilin C) | 0.00042681 | 1 | 0.76149608 | 1 |
| 209000_s_at | SEPT8 | septin 8 | 0.000432549 | 1 | 0.76149608 | 1 |
| 244563_at | QSER1 | glutamine and serine rich 1 | 0.000437776 | 1 | 0.76149608 | 1 |
| 1558687_a_at | FOXN1 | forkhead box N1 | 0.000438571 | 1 | 0.76149608 | 1 |
| 219597_s_at | DUOX1 | dual oxidase 1 | 0.000440437 | 1 | 0.76149608 | 1 |
| 200660_at | S100A11 | S100 calcium binding protein A11 | 0.000442219 | 1 | 0.196647353 | 1 |
| 213011_s_at | TPI1 | triosephosphate isomerase 1 | 0.000447568 | 1 | 0.215152065 | 1 |
| 212285_s_at | AGRN | agrin | 0.000449131 | 1 | 0.305056885 | 1 |
| 217777_s_at | PTPLAD1 | protein tyrosine phosphatase-like A domain containing 1 | 0.000453822 | 1 | 0.76149608 | 1 |
| 204787_at | VSIG4 | V-set and immunoglobulin domain containing 4 | 0.00045514 | 0.858562945 | 7.00E-08 | 1 |
| 202712_s_at | CKMT1B | creatine kinase, mitochondrial 1B | 0.000455714 | 1 | 0.76149608 | 1 |
| 214452_at | BCAT1 | branched chain aminotransferase 1, cytosolic | 0.000458311 | 1 | 0.76149608 | 1 |
| 203961_at | NEBL | nebulette | 0.000459131 | 1 | 0.80604048 | 1 |
| 215867_x_at | CA12 | carbonic anhydrase XII | 0.000466977 | 1 | 0.246250467 | 1 |
| 201417_at | SOX4 | SRY (sex determining region Y)-box 4 | 0.000468934 | 1 | 0.001399342 | 1 |
| 201109_s_at | THBS1 | thrombospondin 1 | 0.000470114 | 1 | 0.254965584 | 1 |
| 231925_at | P2RY1 | purinergic receptor P2Y, G-protein coupled, 1 | 0.000472896 | 1 | 0.76149608 | 1 |
| 218663_at | NCAPG | non-SMC condensin I complex, subunit G | 0.000473998 | 1 | 0.404775076 | 1 |
| 224328_s_at | LCE3D | late cornified envelope 3D | 0.000477234 | 1 | 0.76149608 | 1 |
| 213909_at | LRRC15 | leucine rich repeat containing 15 | 0.000479409 | 1 | 0.004288003 | 1 |
| 218662_s_at | NCAPG | non-SMC condensin I complex, subunit G | 0.000489937 | 1 | 0.76149608 | 1 |
| 232278_s_at | DEPDC1 | DEP domain containing 1 | 0.000492676 | 1 | 0.76149608 | 1 |
| 212141_at | MCM4 | minichromosome maintenance complex component 4 | 0.000492897 | 1 | 0.37134827 | 1 |
| 229103_at | WNT3 | wingless-type MMTV integration site family, member 3 | 0.0004987 | 1 | 0.76149608 | 1 |
| 225664_at | COL12A1 | collagen, type XII, alpha 1 | 0.000500342 | 1 | 0.000867127 | 1 |
| 228824_s_at | LTB4DH | leukotriene B4 12-hydroxydehydrogenase | 0.000500523 | 1 | 0.76149608 | 1 |
| 231964_at | NA | NA | 0.000500939 | 0.38272118 | 0.764147552 | 0.757924823 |
| 210301_at | XDH | xanthine dehydrogenase | 0.000501527 | 1 | 0.76149608 | 0.889494966 |
| 214953_s_at | APP | amyloid beta (A4) precursor protein (peptidase nexin-II, Alzheimer disease) | 0.00050767 | 1 | 0.032738804 | 1 |
| 228115_at | NA | NA | 0.000509206 | 1 | 0.76149608 | 1 |
| 202856_s_at | SLC16A3 | solute carrier family 16, member 3 (monocarboxylic acid transporter 4) | 0.000510059 | 1 | 0.01763806 | 1 |
| 212657_s_at | IL1RN | interleukin 1 receptor antagonist | 0.000510173 | 1 | 0.232124085 | 1 |
| 223710_at | CCL26 | chemokine (C-C motif) ligand 26 | 0.00051165 | 0.835232341 | 0.230231092 | 0.757924823 |
| 1552487_a_at | BNC1 | basonuclin 1 | 0.00051165 | 1 | 0.352045203 | 1 |
| 203213_at | CDC2 | cell division cycle 2, G1 to S and G2 to M | 0.000512865 | 1 | 0.53680827 | 1 |
| 202342_s_at | TRIM2 | tripartite motif-containing 2 | 0.000512865 | 1 | 1 | 0.757924823 |
| 225702_at | C8orf76 | chromosome 8 open reading frame 76 | 0.000512865 | 1 | 0.365795995 | 0.925968647 |
| 226380_at | PTPN21 | protein tyrosine phosphatase, non-receptor type 21 | 0.000519059 | 1 | 0.76149608 | 1 |
| 213577_at | SQLE | squalene epoxidase | 0.000519059 | 1 | 0.119784075 | 1 |
| 201946_s_at | CCT2 | chaperonin containing TCP1, subunit 2 (beta) | 0.000520389 | 1 | 0.76149608 | 1 |
| 227372_s_at | BAIAP2L1 | BAI1-associated protein 2-like 1 | 0.000522888 | 1 | 0.20852244 | 1 |
| 200906_s_at | PALLD | palladin, cytoskeletal associated protein | 0.000528546 | 1 | 0.76149608 | 1 |
| 206025_s_at | TNFAIP6 | tumor necrosis factor, alpha-induced protein 6 | 0.000535865 | 1 | 0.000348011 | 1 |
| 218718_at | PDGFC | platelet derived growth factor C | 0.000536227 | 1 | 0.087354305 | 0.956791102 |
| 38037_at | HBEGF | heparin-binding EGF-like growth factor | 0.000536227 | 1 | 0.555668634 | 1 |
| 213422_s_at | MXRA8 | matrix-remodelling associated 8 | 0.000536227 | 1 | 0.029133622 | 1 |
| 214146_s_at | PPBP | pro-platelet basic protein (chemokine (C-X-C motif) ligand 7) | 0.000548323 | 1 | 0.76149608 | 0.986799178 |
| 1553081_at | WFDC12 | WAP four-disulfide core domain 12 | 0.000549394 | 1 | 0.76149608 | 1 |
| 224690_at | C20orf108 | chromosome 20 open reading frame 108 | 0.000551574 | 1 | 0.76149608 | 1 |
| 206125_s_at | KLK8 | kallikrein-related peptidase 8 | 0.000553577 | 1 | 0.76149608 | 1 |
| 213427_at | RPP40 | ribonuclease P/MRP 40kDa subunit | 0.000555614 | 1 | 0.76149608 | 1 |
| 204444_at | KIF11 | kinesin family member 11 | 0.000559705 | 1 | 0.234641531 | 1 |
| 236035_at | NA | NA | 0.000562962 | 0.985157606 | 0.703335156 | 0.938195781 |
| 223497_at | FAM135A | family with sequence similarity 135, member A | 0.000562962 | 1 | 0.76149608 | 1 |
| 242064_at | SDK2 | sidekick homolog 2 (chicken) | 0.000566342 | 1 | 0.76149608 | 0.757924823 |
| 223463_at | RAB23 | RAB23, member RAS oncogene family | 0.000567281 | 1 | 0.509637019 | 1 |
| 207717_s_at | PKP2 | plakophilin 2 | 0.000568696 | 1 | 0.76149608 | 1 |
| 214807_at | NA | NA | 0.000582837 | 1 | 0.048467407 | 1 |
| 200894_s_at | FKBP4 | FK506 binding protein 4, 59kDa | 0.000583865 | 1 | 0.76149608 | 1 |
| 212142_at | MCM4 | minichromosome maintenance complex component 4 | 0.000583865 | 1 | 0.76149608 | 1 |
| 222716_s_at | SNX24 | sorting nexin 24 | 0.000590075 | 1 | 0.757033931 | 0.96978281 |
| 228188_at | FOSL2 | FOS-like antigen 2 | 0.000593533 | 1 | 0.209568282 | 1 |
| 202330_s_at | UNG | uracil-DNA glycosylase | 0.000596845 | 1 | 0.76149608 | 1 |
| 1554807_a_at | SPIRE1 | spire homolog 1 (Drosophila) | 0.000602211 | 1 | 0.76149608 | 0.977881842 |
| 223037_at | PDZD11 | PDZ domain containing 11 | 0.000609114 | 1 | 0.664775704 | 1 |
| 221843_s_at | KIAA1609 | KIAA1609 | 0.000609114 | 1 | 0.28849273 | 1 |
| 205990_s_at | WNT5A | wingless-type MMTV integration site family, member 5A | 0.000611918 | 1 | 0.76149608 | 1 |
| 225832_s_at | DAGLB | diacylglycerol lipase, beta | 0.000614665 | 1 | 0.76149608 | 0.94871386 |
| 226226_at | TMEM45B | transmembrane protein 45B | 0.000614665 | 1 | 0.76149608 | 1 |
| 204796_at | EML1 | echinoderm microtubule associated protein like 1 | 0.000616012 | 1 | 0.76149608 | 1 |
| 214770_at | MSR1 | macrophage scavenger receptor 1 | 0.000623597 | 1 | 0.005542578 | 1 |
| 204887_s_at | PLK4 | polo-like kinase 4 (Drosophila) | 0.000632582 | 1 | 0.76149608 | 1 |
| 200650_s_at | LDHA | lactate dehydrogenase A | 0.000635077 | 1 | 0.188069788 | 1 |
| 222392_x_at | PERP | PERP, TP53 apoptosis effector | 0.000641787 | 1 | 0.113999991 | 1 |
| 227180_at | ELOVL7 | ELOVL family member 7, elongation of long chain fatty acids (yeast) | 0.000642675 | 1 | 0.76149608 | 1 |
| 212340_at | YIPF6 | Yip1 domain family, member 6 | 0.000647996 | 1 | 0.76149608 | 0.927759528 |
| 219434_at | TREM1 | triggering receptor expressed on myeloid cells 1 | 0.000650824 | 1 | 0.119100498 | 1 |
| 203305_at | F13A1 | coagulation factor XIII, A1 polypeptide | 0.00065648 | 0.892735038 | 0.075044753 | 1 |
| 205363_at | BBOX1 | butyrobetaine (gamma), 2-oxoglutarate dioxygenase (gamma-butyrobetaine hydroxylase) 1 | 0.000657603 | 1 | 0.76149608 | 1 |
| 229900_at | CD109 | CD109 molecule | 0.000661847 | 1 | 0.76149608 | 1 |
| 214011_s_at | HSPC111 | hypothetical protein HSPC111 | 0.000663372 | 1 | 0.76149608 | 1 |
| 218502_s_at | TRPS1 | trichorhinophalangeal syndrome I | 0.000672776 | 1 | 0.76149608 | 1 |
| 225450_at | AMOTL1 | angiomotin like 1 | 0.000675583 | 1 | 0.76149608 | 1 |
| 212907_at | SLC30A1 | solute carrier family 30 (zinc transporter), member 1 | 0.000678863 | 1 | 0.76149608 | 1 |
| 223220_s_at | PARP9 | poly (ADP-ribose) polymerase family, member 9 | 0.000678863 | 1 | 0.398037822 | 1 |
| 1555310_a_at | PAK6 | p21(CDKN1A)-activated kinase 6 | 0.000679207 | 1 | 0.76149608 | 1 |
| 219684_at | RTP4 | receptor (chemosensory) transporter protein 4 | 0.000679207 | 1 | 0.053623717 | 1 |
| 212460_at | C14orf147 | chromosome 14 open reading frame 147 | 0.000681179 | 1 | 0.197738854 | 1 |
| 209493_at | PDZD2 | PDZ domain containing 2 | 0.00068357 | 1 | 0.992732822 | 1 |
| 207908_at | KRT2 | keratin 2 (epidermal ichthyosis bullosa of Siemens) | 0.00068726 | 1 | 0.769964366 | 1 |
| 235093_at | NA | NA | 0.000699566 | 1 | 0.76149608 | 1 |
| 219793_at | SNX16 | sorting nexin 16 | 0.000701262 | 1 | 0.76149608 | 1 |
| 213696_s_at | MED8 | mediator complex subunit 8 | 0.000703496 | 1 | 0.608894252 | 1 |
| 208653_s_at | CD164 | CD164 molecule, sialomucin | 0.00071196 | 1 | 0.76149608 | 1 |
| 36499_at | CELSR2 | cadherin, EGF LAG seven-pass G-type receptor 2 (flamingo homolog, Drosophila) | 0.000712145 | 1 | 0.76149608 | 1 |
| 209949_at | NCF2 | neutrophil cytosolic factor 2 (65kDa, chronic granulomatous disease, autosomal 2) | 0.000714786 | 1 | 0.000251536 | 1 |
| 210992_x_at | FCGR2C | Fc fragment of IgG, low affinity IIc, receptor for (CD32) | 0.000732954 | 1 | 0.76149608 | 1 |
| 212175_s_at | AK2 | adenylate kinase 2 | 0.000734363 | 1 | 0.76149608 | 1 |
| 213030_s_at | PLXNA2 | plexin A2 | 0.000739061 | 1 | 0.342973574 | 0.797788378 |
| 225100_at | FBXO45 | F-box protein 45 | 0.000746528 | 1 | 0.76149608 | 1 |
| 228310_at | ENAH | enabled homolog (Drosophila) | 0.000752075 | 0.835232341 | 0.9323922 | 0.757924823 |
| 1563900_at | FAM83B | family with sequence similarity 83, member B | 0.000756907 | 1 | 0.76149608 | 1 |
| 205968_at | KCNS3 | potassium voltage-gated channel, delayed-rectifier, subfamily S, member 3 | 0.000760291 | 1 | 0.76149608 | 0.946258816 |
| 208097_s_at | TXNDC1 | thioredoxin domain containing 1 | 0.000768276 | 1 | 0.76149608 | 1 |
| 218880_at | FOSL2 | FOS-like antigen 2 | 0.00076991 | 1 | 0.119784075 | 1 |
| 227475_at | FOXQ1 | forkhead box Q1 | 0.000773159 | 1 | 0.093058266 | 1 |
| 209373_at | MALL | mal, T-cell differentiation protein-like | 0.000773159 | 1 | 0.76149608 | 1 |
| 209363_s_at | MED21 | mediator complex subunit 21 | 0.000773981 | 1 | 0.76149608 | 1 |
| 225300_at | C15orf23 | chromosome 15 open reading frame 23 | 0.000778795 | 1 | 0.76149608 | 1 |
| 229549_at | OPN1SW | opsin 1 (cone pigments), short-wave-sensitive (color blindness, tritan) | 0.00078273 | 1 | 0.76149608 | 1 |
| 229699_at | NA | NA | 0.000783853 | 0.936749556 | 0.76149608 | 0.757924823 |
| 204204_at | SLC31A2 | solute carrier family 31 (copper transporters), member 2 | 0.000784325 | 1 | 0.252708785 | 1 |
| 207275_s_at | ACSL1 | acyl-CoA synthetase long-chain family member 1 | 0.000793725 | 1 | 0.76149608 | 1 |
| 212768_s_at | OLFM4 | olfactomedin 4 | 0.000799531 | 0.835232341 | 0.76149608 | 0.758617479 |
| 222989_s_at | UBQLN1 | ubiquilin 1 | 0.000812777 | 1 | 0.76149608 | 1 |
| 216268_s_at | JAG1 | jagged 1 (Alagille syndrome) | 0.000815452 | 1 | 0.027768911 | 1 |
| 219296_at | ZDHHC13 | zinc finger, DHHC-type containing 13 | 0.000817707 | 1 | 0.76149608 | 1 |
| 236616_at | NA | NA | 0.000817707 | 1 | 0.810944439 | 0.790774191 |
| 214110_s_at | LOC654342 | similar to lymphocyte-specific protein 1 | 0.000817831 | 0.835232341 | 0.060397871 | 1 |
| 238320_at | TncRNA | trophoblast-derived noncoding RNA | 0.000821715 | 1 | 0.194233667 | 1 |
| 208862_s_at | CTNND1 | catenin (cadherin-associated protein), delta 1 | 0.000821876 | 1 | 0.76149608 | 1 |
| 212014_x_at | CD44 | CD44 molecule (Indian blood group) | 0.000821876 | 1 | 8.32E-06 | 1 |
| 227970_at | GPR157 | G protein-coupled receptor 157 | 0.000826164 | 1 | 0.76149608 | 1 |
| 201300_s_at | PRNP | prion protein (p27-30) (Creutzfeldt-Jakob disease, Gerstmann-Strausler-Scheinker syndrome, fatal familial insomnia) | 0.000826615 | 1 | 0.76149608 | 1 |
| 212186_at | ACACA | acetyl-Coenzyme A carboxylase alpha | 0.000827607 | 1 | 0.76149608 | 0.982151144 |
| 203072_at | MYO1E | myosin IE | 0.000829635 | 1 | 0.76149608 | 0.982363874 |
| 224358_s_at | MS4A7 | membrane-spanning 4-domains, subfamily A, member 7 | 0.00083631 | 1 | 0.318717423 | 1 |
| 211668_s_at | PLAU | plasminogen activator, urokinase | 0.000837171 | 1 | 0.060253615 | 1 |
| 207180_s_at | HTATIP2 | HIV-1 Tat interactive protein 2, 30kDa | 0.000855624 | 1 | 0.76149608 | 0.961279485 |
| 235573_at | NA | NA | 0.000866567 | 1 | 0.76149608 | 0.890907292 |
| 209771_x_at | CD24 | CD24 molecule | 0.000872149 | 1 | 0.572756439 | 1 |
| 227112_at | TMCC1 | transmembrane and coiled-coil domain family 1 | 0.000872149 | 1 | 0.421658404 | 0.76987956 |
| 206777_s_at | CRYBB2 | crystallin, beta B2 | 0.000878413 | 0.836727745 | 0.767208877 | 0.757924823 |
| 222793_at | DDX58 | DEAD (Asp-Glu-Ala-Asp) box polypeptide 58 | 0.000878413 | 1 | 0.76149608 | 0.995291276 |
| 1564358_at | NA | NA | 0.000882301 | 1 | 0.76149608 | 0.90248759 |
| 242546_at | LOC440157 | hypothetical gene supported by BC066547 | 0.000883741 | 0.854158783 | 0.76149608 | 0.757924823 |
| 222631_at | PI4K2B | phosphatidylinositol 4-kinase type 2 beta | 0.000888607 | 1 | 0.76149608 | 0.795461012 |
| 232530_at | PLD1 | phospholipase D1, phosphatidylcholine-specific | 0.000894164 | 1 | 2.50E-05 | 1 |
| 202023_at | EFNA1 | ephrin-A1 | 0.000894749 | 1 | 0.76149608 | 1 |
| 209935_at | ATP2C1 | ATPase, Ca++ transporting, type 2C, member 1 | 0.00090465 | 1 | 0.032266862 | 1 |
| 219909_at | MMP28 | matrix metallopeptidase 28 | 0.00090465 | 1 | 0.76149608 | 0.808420001 |
| 218631_at | AVPI1 | arginine vasopressin-induced 1 | 0.000908286 | 1 | 0.76149608 | 1 |
| 222344_at | C5orf13 | chromosome 5 open reading frame 13 | 0.000913592 | 1 | 0.632709156 | 1 |
| 231930_at | ELMOD1 | ELMO/CED-12 domain containing 1 | 0.000914964 | 1 | 0.76149608 | 1 |
| 226615_at | NA | NA | 0.000917967 | 0.843401695 | 0.423476255 | 0.788301712 |
| 213558_at | PCLO | piccolo (presynaptic cytomatrix protein) | 0.000918567 | 0.835232341 | 0.76149608 | 0.850206887 |
| 235287_at | CDK6 | cyclin-dependent kinase 6 | 0.000919102 | 0.835232341 | 0.776086628 | 1 |
| 200641_s_at | YWHAZ | tyrosine 3-monooxygenase/tryptophan 5-monooxygenase activation protein, zeta polypeptide | 0.000926249 | 1 | 0.76149608 | 1 |
| 227735_s_at | C10orf99 | chromosome 10 open reading frame 99 | 0.00093453 | 1 | 0.76149608 | 1 |
| 224650_at | MAL2 | mal, T-cell differentiation protein 2 | 0.00093939 | 1 | 0.76149608 | 1 |
| 226926_at | DMKN | dermokine | 0.000941938 | 1 | 0.76149608 | 1 |
| 200697_at | HK1 | hexokinase 1 | 0.000947891 | 1 | 0.76149608 | 1 |
| 220955_x_at | RAB23 | RAB23, member RAS oncogene family | 0.000951599 | 1 | 0.76149608 | 1 |
| 1555501_s_at | RSRC1 | arginine/serine-rich coiled-coil 1 | 0.000952111 | 1 | 0.76149608 | 0.91457788 |
| 223952_x_at | DHRS9 | dehydrogenase/reductase (SDR family) member 9 | 0.000952111 | 1 | 0.76149608 | 0.757924823 |
| 230151_at | C13orf1 | chromosome 13 open reading frame 1 | 0.000954263 | 1 | 0.76149608 | 1 |
| 217173_s_at | LDLR | low density lipoprotein receptor (familial hypercholesterolemia) | 0.000955136 | 1 | 0.76149608 | 1 |
| 236641_at | KIF14 | kinesin family member 14 | 0.000960099 | 1 | 0.76149608 | 1 |
| 205072_s_at | XRCC4 | X-ray repair complementing defective repair in Chinese hamster cells 4 | 0.000960099 | 1 | 0.76149608 | 0.85501266 |
| 218856_at | TNFRSF21 | tumor necrosis factor receptor superfamily, member 21 | 0.000960412 | 1 | 0.749298616 | 0.778337768 |
| 215465_at | ABCA12 | ATP-binding cassette, sub-family A (ABC1), member 12 | 0.000960412 | 1 | 0.76149608 | 1 |
| 201476_s_at | RRM1 | ribonucleotide reductase M1 polypeptide | 0.0009674 | 1 | 0.76149608 | 1 |
| 235821_at | NA | NA | 0.000971849 | 1 | 0.76149608 | 1 |
| 226893_at | NA | NA | 0.000977152 | 1 | 0.76149608 | 1 |
| 234725_s_at | SEMA4B | sema domain, immunoglobulin domain (Ig), transmembrane domain (TM) and short cytoplasmic domain, (semaphorin) 4B | 0.00097859 | 1 | 0.76149608 | 0.99290851 |
| 1554062_at | XG | Xg blood group | 0.000982585 | 1 | 0.193881293 | 1 |
| 201324_at | EMP1 | epithelial membrane protein 1 | 0.000988681 | 1 | 0.76149608 | 1 |
| 221447_s_at | GLT8D2 | glycosyltransferase 8 domain containing 2 | 0.000989425 | 1 | 0.76149608 | 1 |
| 230002_at | CLCC1 | chloride channel CLIC-like 1 | 0.000990795 | 1 | 0.76149608 | 1 |
| 214070_s_at | ATP10B | ATPase, Class V, type 10B | 0.001017351 | 1 | 0.76149608 | 1 |
| 230266_at | RAB7B | RAB7B, member RAS oncogene family | 0.001025601 | 1 | 0.76149608 | 1 |
| 213004_at | ANGPTL2 | angiopoietin-like 2 | 0.001030108 | 1 | 0.065838758 | 1 |
| 217080_s_at | HOMER2 | homer homolog 2 (Drosophila) | 0.001033968 | 1 | 0.76149608 | 1 |
| 238654_at | LOC147645 | hypothetical protein LOC147645 | 0.001037037 | 1 | 0.76149608 | 1 |
| 205289_at | BMP2 | bone morphogenetic protein 2 | 0.001042119 | 1 | 0.76149608 | 0.757924823 |
| 211981_at | COL4A1 | collagen, type IV, alpha 1 | 0.001047012 | 1 | 0.311840926 | 1 |
| 230951_at | EPB41L5 | erythrocyte membrane protein band 4.1 like 5 | 0.001051209 | 1 | 0.861026643 | 0.926714483 |
| 214430_at | GLA | galactosidase, alpha | 0.001059948 | 1 | 0.646960757 | 1 |
| 202826_at | SPINT1 | serine peptidase inhibitor, Kunitz type 1 | 0.001066881 | 1 | 0.669554127 | 1 |
| 233888_s_at | SRGAP1 | SLIT-ROBO Rho GTPase activating protein 1 | 0.001075803 | 1 | 0.76149608 | 0.999016625 |
| 210355_at | PTHLH | parathyroid hormone-like hormone | 0.00108274 | 1 | 0.76149608 | 1 |
| 210473_s_at | GPR125 | G protein-coupled receptor 125 | 0.001097911 | 1 | 0.76149608 | 1 |
| 224868_at | ZDHHC5 | zinc finger, DHHC-type containing 5 | 0.001099096 | 1 | 0.76149608 | 1 |
| 207018_s_at | RAB27B | RAB27B, member RAS oncogene family | 0.001104403 | 1 | 0.76149608 | 1 |
| 227889_at | AYTL1 | acyltransferase like 1 | 0.001105728 | 1 | 0.026660991 | 1 |
| 204337_at | RGS4 | regulator of G-protein signaling 4 | 0.00111313 | 1 | 0.158683092 | 1 |
| 229764_at | FAM79B | family with sequence similarity 79, member B | 0.001113148 | 1 | 0.76149608 | 1 |
| 209008_x_at | KRT8 | keratin 8 | 0.001116286 | 1 | 0.618395117 | 1 |
| 240382_at | DSP | desmoplakin | 0.001117761 | 1 | 0.76149608 | 0.920055971 |
| 202504_at | TRIM29 | tripartite motif-containing 29 | 0.001117761 | 1 | 0.204183675 | 1 |
| 206432_at | HAS2 | hyaluronan synthase 2 | 0.001117761 | 1 | 0.76149608 | 1 |
| 222451_s_at | ZDHHC9 | zinc finger, DHHC-type containing 9 | 0.00111838 | 1 | 0.462028586 | 1 |
| 221664_s_at | F11R | F11 receptor | 0.00111838 | 1 | 0.284601398 | 1 |
| 208606_s_at | WNT4 | wingless-type MMTV integration site family, member 4 | 0.00111838 | 1 | 0.30650347 | 1 |
| 228067_at | C2orf55 | chromosome 2 open reading frame 55 | 0.001122397 | 1 | 0.76149608 | 1 |
| 225582_at | KIAA1754 | KIAA1754 | 0.001130881 | 1 | 0.76149608 | 0.917105859 |
| 205084_at | BCAP29 | B-cell receptor-associated protein 29 | 0.001131335 | 1 | 0.76149608 | 1 |
| 205127_at | PTGS1 | prostaglandin-endoperoxide synthase 1 (prostaglandin G/H synthase and cyclooxygenase) | 0.001138284 | 1 | 0.76149608 | 1 |
| 231381_at | HESRG | embryonic stem cell related protein | 0.001140923 | 0.835392277 | 0.76149608 | 0.757924823 |
| 213008_at | FANCI | Fanconi anemia, complementation group I | 0.001144149 | 1 | 0.76149608 | 1 |
| 213993_at | SPON1 | spondin 1, extracellular matrix protein | 0.001170289 | 1 | 0.01042533 | 1 |
| 210650_s_at | PCLO | piccolo (presynaptic cytomatrix protein) | 0.001195147 | 0.835232341 | 0.76149608 | 0.854814446 |
| 204136_at | COL7A1 | collagen, type VII, alpha 1 (epidermolysis bullosa, dystrophic, dominant and recessive) | 0.001195847 | 1 | 0.099059447 | 1 |
| 204162_at | NDC80 | NDC80 homolog, kinetochore complex component (S. cerevisiae) | 0.001204766 | 1 | 0.460622493 | 1 |
| 203786_s_at | TPD52L1 | tumor protein D52-like 1 | 0.001210036 | 1 | 0.76149608 | 1 |
| 1560712_at | TMPRSS11B | transmembrane protease, serine 11B | 0.001212372 | 1 | 0.76149608 | 0.757924823 |
| 203023_at | HSPC111 | hypothetical protein HSPC111 | 0.001216724 | 1 | 0.76149608 | 1 |
| 226177_at | GLTP | glycolipid transfer protein | 0.001221961 | 1 | 0.76149608 | 1 |
| 212091_s_at | COL6A1 | collagen, type VI, alpha 1 | 0.001222303 | 1 | 0.019282144 | 1 |
| 228273_at | NA | NA | 0.001235278 | 1 | 0.000301734 | 1 |
| 212859_x_at | MT1E | metallothionein 1E | 0.00124757 | 1 | 0.10563304 | 1 |
| 225799_at | LOC541471 | hypothetical LOC541471 | 0.00124757 | 1 | 0.135799489 | 1 |
| 207847_s_at | MUC1 | mucin 1, cell surface associated | 0.00124757 | 1 | 0.76149608 | 0.994001887 |
| 218424_s_at | STEAP3 | STEAP family member 3 | 0.00124757 | 1 | 0.059812998 | 1 |
| 225603_s_at | NA | NA | 0.001257933 | 0.835232341 | 0.76149608 | 1 |
| 205194_at | PSPH | phosphoserine phosphatase | 0.001261799 | 1 | 0.402731094 | 0.848556458 |
| 218186_at | RAB25 | RAB25, member RAS oncogene family | 0.001266218 | 1 | 0.586408931 | 1 |
| 222379_at | NA | NA | 0.001274881 | 0.535701189 | 0.231443573 | 0.757924823 |
| 235592_at | ELL2 | elongation factor, RNA polymerase II, 2 | 0.001277588 | 1 | 0.222144237 | 0.757924823 |
| 225367_at | PGM2 | phosphoglucomutase 2 | 0.001289908 | 1 | 0.76149608 | 1 |
| 202138_x_at | JTV1 | JTV1 gene | 0.001293543 | 1 | 0.180804398 | 1 |
| 209169_at | GPM6B | glycoprotein M6B | 0.001301809 | 1 | 0.76149608 | 1 |
| 244353_s_at | SLC2A12 | solute carrier family 2 (facilitated glucose transporter), member 12 | 0.001315497 | 0.954674177 | 0.76149608 | 0.975539151 |
| 211527_x_at | VEGFA | vascular endothelial growth factor A | 0.001337743 | 1 | 0.76149608 | 0.81767016 |
| 208423_s_at | MSR1 | macrophage scavenger receptor 1 | 0.001342678 | 1 | 0.76149608 | 1 |
| 221794_at | DOCK6 | dedicator of cytokinesis 6 | 0.001343783 | 1 | 0.76149608 | 0.916590359 |
| 244107_at | NA | NA | 0.001379834 | 1 | 0.76149608 | 1 |
| 201340_s_at | ENC1 | ectodermal-neural cortex (with BTB-like domain) | 0.001396127 | 1 | 0.128991538 | 1 |
| 1553077_at | SDR-O | orphan short-chain dehydrogenase / reductase | 0.001413608 | 1 | 0.76149608 | 1 |
| 215380_s_at | C7orf24 | chromosome 7 open reading frame 24 | 0.001419349 | 1 | 0.76149608 | 1 |
| 1552797_s_at | PROM2 | prominin 2 | 0.001419349 | 1 | 0.384127883 | 1 |
| 205778_at | KLK7 | kallikrein-related peptidase 7 | 0.001419349 | 1 | 0.459485614 | 1 |
| 223126_s_at | C1orf21 | chromosome 1 open reading frame 21 | 0.001424917 | 1 | 0.855319625 | 1 |
| 222033_s_at | FLT1 | fms-related tyrosine kinase 1 (vascular endothelial growth factor/vascular permeability factor receptor) | 0.001431326 | 1 | 0.820854911 | 0.757924823 |
| 205109_s_at | ARHGEF4 | Rho guanine nucleotide exchange factor (GEF) 4 | 0.001455243 | 1 | 0.76149608 | 1 |
| 221485_at | B4GALT5 | UDP-Gal:betaGlcNAc beta 1,4- galactosyltransferase, polypeptide 5 | 0.001473439 | 1 | 0.141681673 | 1 |
| 228124_at | ABHD12 | abhydrolase domain containing 12 | 0.001476964 | 1 | 0.76149608 | 1 |
| 228765_at | GTF2IRD2 | GTF2I repeat domain containing 2 | 0.001479832 | 1 | 0.76149608 | 0.757924823 |
| 1438_at | EPHB3 | EPH receptor B3 | 0.00148546 | 1 | 0.76149608 | 1 |
| 234915_s_at | DENR | density-regulated protein | 0.001489717 | 1 | 0.76149608 | 1 |
| 236407_at | KCNE1 | potassium voltage-gated channel, Isk-related family, member 1 | 0.00149132 | 0.835232341 | 0.831209585 | 0.757924823 |
| 235643_at | SAMD9L | sterile alpha motif domain containing 9-like | 0.00149132 | 1 | 0.76149608 | 0.87118226 |
| 226870_at | COMTD1 | catechol-O-methyltransferase domain containing 1 | 0.001502959 | 1 | 0.76149608 | 0.970584768 |
| 219756_s_at | POF1B | premature ovarian failure, 1B | 0.001502959 | 1 | 0.76149608 | 1 |
| 221881_s_at | CLIC4 | chloride intracellular channel 4 | 0.001514625 | 1 | 0.76149608 | 1 |
| 200800_s_at | HSPA1A | heat shock 70kDa protein 1A | 0.001517451 | 1 | 0.76149608 | 1 |
| 228261_at | MIB2 | mindbomb homolog 2 (Drosophila) | 0.001518874 | 1 | 0.76149608 | 0.930015162 |
| 1556793_a_at | FAM83C | family with sequence similarity 83, member C | 0.001535968 | 1 | 0.560427662 | 1 |
| 206102_at | GINS1 | GINS complex subunit 1 (Psf1 homolog) | 0.001535968 | 1 | 0.255560084 | 1 |
| 229352_at | NOX5 | NADPH oxidase, EF-hand calcium binding domain 5 | 0.001548769 | 0.971254882 | 0.76149608 | 0.990671716 |
| 202671_s_at | PDXK | pyridoxal (pyridoxine, vitamin B6) kinase | 0.001548769 | 1 | 0.019433393 | 1 |
| 216258_s_at | SERPINB13 | serpin peptidase inhibitor, clade B (ovalbumin), member 13 | 0.001565347 | 1 | 0.76149608 | 1 |
| 232277_at | NA | NA | 0.001565621 | 1 | 0.073953909 | 1 |
| 225599_s_at | LOC286144 | hypothetical protein LOC286144 | 0.001569709 | 1 | 0.135655695 | 1 |
| 200935_at | CALR | calreticulin | 0.001572273 | 1 | 0.011029308 | 0.974563843 |
| 209684_at | RIN2 | Ras and Rab interactor 2 | 0.001575265 | 1 | 0.303172116 | 1 |
| 204994_at | MX2 | myxovirus (influenza virus) resistance 2 (mouse) | 0.001576827 | 1 | 0.76149608 | 1 |
| 231175_at | C6orf65 | chromosome 6 open reading frame 65 | 0.001577496 | 1 | 0.105381937 | 1 |
| 203586_s_at | ARL4D | ADP-ribosylation factor-like 4D | 0.001578484 | 1 | 0.76149608 | 1 |
| 225987_at | STEAP4 | STEAP family member 4 | 0.001581013 | 1 | 1 | 1 |
| 201952_at | ALCAM | activated leukocyte cell adhesion molecule | 0.001581795 | 0.835232341 | 0.76149608 | 0.757924823 |
| 227021_at | AOF1 | amine oxidase (flavin containing) domain 1 | 0.001601871 | 1 | 0.76149608 | 0.908347012 |
| 219874_at | SLC12A8 | solute carrier family 12 (potassium/chloride transporters), member 8 | 0.001608778 | 1 | 0.260540583 | 0.989329893 |
| 219412_at | RAB38 | RAB38, member RAS oncogene family | 0.001620344 | 1 | 0.615857642 | 1 |
| 208579_x_at | H2BFS | H2B histone family, member S | 0.001635894 | 1 | 0.672208476 | 0.757924823 |
| 216915_s_at | PTPN12 | protein tyrosine phosphatase, non-receptor type 12 | 0.001644548 | 1 | 0.76149608 | 1 |
| 203413_at | NELL2 | NEL-like 2 (chicken) | 0.001647108 | 1 | 1 | 0.918275845 |
| 221962_s_at | UBE2H | ubiquitin-conjugating enzyme E2H (UBC8 homolog, yeast) | 0.001647108 | 1 | 0.76149608 | 1 |
| 203343_at | UGDH | UDP-glucose dehydrogenase | 0.001652708 | 1 | 0.01017324 | 1 |
| 208216_at | DLX4 | distal-less homeobox 4 | 0.001672179 | 0.890699421 | 0.76149608 | 0.770827734 |
| 1553994_at | NT5E | 5'-nucleotidase, ecto (CD73) | 0.001683908 | 1 | 0.782516684 | 1 |
| 204331_s_at | MRPS12 | mitochondrial ribosomal protein S12 | 0.001684803 | 1 | 0.76149608 | 1 |
| 225345_s_at | NA | NA | 0.001689743 | 0.835232341 | 0.76149608 | 0.757924823 |
| 229057_at | SCN2A | sodium channel, voltage-gated, type II, alpha subunit | 0.001695716 | 1 | 0.76149608 | 0.979626778 |
| 218722_s_at | CCDC51 | coiled-coil domain containing 51 | 0.001696301 | 1 | 0.76149608 | 0.950104233 |
| 204989_s_at | ITGB4 | integrin, beta 4 | 0.001701185 | 1 | 0.268024077 | 1 |
| 206429_at | F2RL1 | coagulation factor II (thrombin) receptor-like 1 | 0.001708254 | 1 | 0.76149608 | 1 |
| 227569_at | LNX2 | ligand of numb-protein X 2 | 0.001727318 | 1 | 0.76149608 | 0.798096072 |
| 1554679_a_at | LAPTM4B | lysosomal associated protein transmembrane 4 beta | 0.00172806 | 1 | 0.352045203 | 1 |
| 225001_at | RAB3D | RAB3D, member RAS oncogene family | 0.001743185 | 1 | 0.76149608 | 1 |
| 202845_s_at | RALBP1 | ralA binding protein 1 | 0.001746109 | 1 | 0.76149608 | 0.873955624 |
| 1558846_at | PNLIPRP3 | pancreatic lipase-related protein 3 | 0.00175062 | 1 | 0.55081196 | 1 |
| 1566764_at | NA | NA | 0.001753378 | 1 | 0.76149608 | 0.757924823 |
| 216950_s_at | FCGR1A | Fc fragment of IgG, high affinity Ia, receptor (CD64) | 0.001753378 | 1 | 0.000307326 | 1 |
| 201438_at | COL6A3 | collagen, type VI, alpha 3 | 0.001753378 | 1 | 0.005195688 | 1 |
| 227467_at | RDH10 | retinol dehydrogenase 10 (all-trans) | 0.001755678 | 1 | 0.369948223 | 1 |
| 220267_at | KRT24 | keratin 24 | 0.001768918 | 1 | 0.76149608 | 0.761667753 |
| 201249_at | SLC2A1 | solute carrier family 2 (facilitated glucose transporter), member 1 | 0.001781011 | 1 | 0.76149608 | 0.943798055 |
| 205319_at | PSCA | prostate stem cell antigen | 0.001781011 | 1 | 0.76149608 | 0.757924823 |
| 218755_at | KIF20A | kinesin family member 20A | 0.001794514 | 1 | 0.76149608 | 1 |
| 214483_s_at | ARFIP1 | ADP-ribosylation factor interacting protein 1 (arfaptin 1) | 0.00180271 | 1 | 0.76149608 | 1 |
| 233487_s_at | LRRC8A | leucine rich repeat containing 8 family, member A | 0.001809407 | 1 | 0.76149608 | 1 |
| 221750_at | HMGCS1 | 3-hydroxy-3-methylglutaryl-Coenzyme A synthase 1 (soluble) | 0.00181132 | 1 | 0.76149608 | 0.989975429 |
| 236009_at | NA | NA | 0.001819615 | 1 | 0.76149608 | 1 |
| 231823_s_at | SH3PXD2B | SH3 and PX domains 2B | 0.001819615 | 1 | 0.018605656 | 1 |
| 241412_at | BTC | betacellulin | 0.001839288 | 0.872365741 | 0.76565684 | 0.968217067 |
| 240557_at | TSC22D2 | TSC22 domain family, member 2 | 0.001848554 | 0.835232341 | 0.76149608 | 0.757924823 |
| 217762_s_at | RAB31 | RAB31, member RAS oncogene family | 0.001848554 | 1 | 0.000640185 | 1 |
| 202238_s_at | NNMT | nicotinamide N-methyltransferase | 0.001864051 | 1 | 1.41E-05 | 1 |
| 1552658_a_at | NAV3 | neuron navigator 3 | 0.001880912 | 1 | 0.76149608 | 1 |
| 227250_at | KREMEN1 | kringle containing transmembrane protein 1 | 0.001883087 | 1 | 0.76149608 | 1 |
| 200734_s_at | ARF3 | ADP-ribosylation factor 3 | 0.001883087 | 1 | 0.377056066 | 1 |
| 238022_at | hCG_1815491 | hCG1815491 | 0.001905081 | 1 | 0.76149608 | 1 |
| 226325_at | ADSSL1 | adenylosuccinate synthase like 1 | 0.001917466 | 1 | 0.76149608 | 0.812573369 |
| 226129_at | FAM83H | family with sequence similarity 83, member H | 0.001926 | 1 | 0.30650347 | 1 |
| 201906_s_at | CTDSPL | CTD (carboxy-terminal domain, RNA polymerase II, polypeptide A) small phosphatase-like | 0.001933622 | 1 | 0.76149608 | 1 |
| 201739_at | SGK | serum/glucocorticoid regulated kinase | 0.001948616 | 1 | 0.76149608 | 1 |
| 204379_s_at | FGFR3 | fibroblast growth factor receptor 3 (achondroplasia, thanatophoric dwarfism) | 0.001969729 | 1 | 0.542196764 | 1 |
| 212086_x_at | LMNA | lamin A/C | 0.001970675 | 1 | 0.76149608 | 1 |
| 210023_s_at | PCGF1 | polycomb group ring finger 1 | 0.00198711 | 1 | 0.76149608 | 0.758045734 |
| 243252_at | NA | NA | 0.002007171 | 1 | 0.76149608 | 1 |
| 226936_at | C6orf173 | chromosome 6 open reading frame 173 | 0.002007171 | 1 | 0.615567958 | 1 |
| 209747_at | TGFB3 | transforming growth factor, beta 3 | 0.00201451 | 0.923213025 | 0.362030555 | 1 |
| 1552544_at | SERPINA12 | serpin peptidase inhibitor, clade A (alpha-1 antiproteinase, antitrypsin), member 12 | 0.00201451 | 1 | 0.76149608 | 1 |
| 215228_at | NHLH2 | nescient helix loop helix 2 | 0.002018143 | 1 | 0.76149608 | 0.785567193 |
| 203041_s_at | LAMP2 | lysosomal-associated membrane protein 2 | 0.002018758 | 1 | 0.141681673 | 1 |
| 226757_at | IFIT2 | interferon-induced protein with tetratricopeptide repeats 2 | 0.002018758 | 1 | 0.76149608 | 1 |
| 219278_at | MAP3K6 | mitogen-activated protein kinase kinase kinase 6 | 0.002022328 | 1 | 0.76149608 | 1 |
| 206199_at | CEACAM7 | carcinoembryonic antigen-related cell adhesion molecule 7 | 0.002022864 | 1 | 0.76149608 | 0.757924823 |
| 228497_at | SLC22A15 | solute carrier family 22 (organic cation transporter), member 15 | 0.002035793 | 1 | 0.76149608 | 0.957740497 |
| 204885_s_at | MSLN | mesothelin | 0.002043783 | 0.835232341 | 0.76149608 | 0.757924823 |
| 202089_s_at | SLC39A6 | solute carrier family 39 (zinc transporter), member 6 | 0.002044612 | 1 | 0.76149608 | 1 |
| 230254_at | NA | NA | 0.00204559 | 0.908466319 | 0.76149608 | 0.989308732 |
| 1554648_a_at | DUOXA1 | dual oxidase maturation factor 1 | 0.002055274 | 1 | 0.76149608 | 1 |
| 206371_at | FOLR3 | folate receptor 3 (gamma) | 0.002067943 | 1 | 0.76149608 | 0.757924823 |
| 222835_at | THSD4 | thrombospondin, type I, domain containing 4 | 0.002067943 | 1 | 0.817503632 | 1 |
| 200638_s_at | YWHAZ | tyrosine 3-monooxygenase/tryptophan 5-monooxygenase activation protein, zeta polypeptide | 0.002098008 | 1 | 0.76149608 | 1 |
| 202752_x_at | SLC7A8 | solute carrier family 7 (cationic amino acid transporter, y+ system), member 8 | 0.002107491 | 1 | 0.250962731 | 0.784960935 |
| 227747_at | MPZL3 | myelin protein zero-like 3 | 0.002107642 | 1 | 0.76149608 | 1 |
| 1553928_at | ELMOD2 | ELMO/CED-12 domain containing 2 | 0.00213067 | 1 | 0.586408931 | 0.978830448 |
| 218817_at | SPCS3 | signal peptidase complex subunit 3 homolog (S. cerevisiae) | 0.002136043 | 1 | 0.175912647 | 0.445357072 |
| 201577_at | NME1 | non-metastatic cells 1, protein (NM23A) expressed in | 0.00214446 | 1 | 0.026056034 | 1 |
| 226829_at | AFAP1L2 | actin filament associated protein 1-like 2 | 0.002146713 | 1 | 0.76149608 | 1 |
| 225807_at | JUB | jub, ajuba homolog (Xenopus laevis) | 0.002170966 | 1 | 0.053762959 | 1 |
| 220295_x_at | DEPDC1 | DEP domain containing 1 | 0.002205299 | 0.999414563 | 0.76149608 | 1 |
| 205239_at | AREG | amphiregulin (schwannoma-derived growth factor) | 0.002205299 | 1 | 0.263622359 | 1 |
| 206976_s_at | HSPH1 | heat shock 105kDa/110kDa protein 1 | 0.002209735 | 1 | 0.76149608 | 0.982901544 |
| 216905_s_at | ST14 | suppression of tumorigenicity 14 (colon carcinoma) | 0.002228352 | 1 | 0.452012122 | 1 |
| 202827_s_at | MMP14 | matrix metallopeptidase 14 (membrane-inserted) | 0.002246376 | 1 | 0.384230819 | 0.993638462 |
| 223229_at | UBE2T | ubiquitin-conjugating enzyme E2T (putative) | 0.002265048 | 1 | 0.76149608 | 1 |
| 223471_at | RAB3IP | RAB3A interacting protein (rabin3) | 0.00227443 | 1 | 0.847603882 | 0.757924823 |
| 212194_s_at | TM9SF4 | transmembrane 9 superfamily protein member 4 | 0.002282143 | 1 | 0.76149608 | 0.818517644 |
| 226490_at | NHSL1 | NHS-like 1 | 0.002282143 | 1 | 0.76149608 | 1 |
| 220942_x_at | C3orf28 | chromosome 3 open reading frame 28 | 0.002282143 | 1 | 0.76149608 | 1 |
| 204429_s_at | SLC2A5 | solute carrier family 2 (facilitated glucose/fructose transporter), member 5 | 0.00228284 | 0.835232341 | 0.76149608 | 0.991683272 |
| 223594_at | TMEM117 | transmembrane protein 117 | 0.002289364 | 1 | 0.76149608 | 1 |
| 1562290_at | PPP2R2C | protein phosphatase 2 (formerly 2A), regulatory subunit B, gamma isoform | 0.002294502 | 1 | 0.76149608 | 0.757924823 |
| 1555773_at | BPIL2 | bactericidal/permeability-increasing protein-like 2 | 0.002296675 | 1 | 0.76149608 | 1 |
| 222383_s_at | ALOXE3 | arachidonate lipoxygenase 3 | 0.002296726 | 1 | 0.76149608 | 1 |
| 225384_at | DOCK7 | dedicator of cytokinesis 7 | 0.002298645 | 1 | 0.76149608 | 1 |
| 205534_at | PCDH7 | protocadherin 7 | 0.002298645 | 1 | 0.140211401 | 1 |
| 204478_s_at | RABIF | RAB interacting factor | 0.002303992 | 1 | 0.76149608 | 1 |
| 208093_s_at | NDEL1 | nudE nuclear distribution gene E homolog (A. nidulans)-like 1 | 0.00230514 | 1 | 0.76149608 | 1 |
| 202993_at | ILVBL | ilvB (bacterial acetolactate synthase)-like | 0.002314009 | 1 | 0.706144049 | 1 |
| 217678_at | SLC7A11 | solute carrier family 7, (cationic amino acid transporter, y+ system) member 11 | 0.002314176 | 1 | 0.76149608 | 1 |
| 213007_at | FANCI | Fanconi anemia, complementation group I | 0.002314176 | 1 | 0.76149608 | 1 |
| 206643_at | HAL | histidine ammonia-lyase | 0.002314642 | 1 | 0.76149608 | 1 |
| 229310_at | KLHL29 | kelch-like 29 (Drosophila) | 0.002324835 | 0.835232341 | 0.799559105 | 0.317666613 |
| 203955_at | KIAA0649 | KIAA0649 | 0.002331062 | 1 | 0.76149608 | 0.757924823 |
| 1558508_a_at | C1orf53 | chromosome 1 open reading frame 53 | 0.002341501 | 1 | 0.76149608 | 0.868029882 |
| 227241_at | MUC15 | mucin 15, cell surface associated | 0.002343385 | 1 | 0.76149608 | 1 |
| 233388_at | CA12 | carbonic anhydrase XII | 0.002346651 | 1 | 0.76149608 | 1 |
| 222881_at | HPSE | heparanase | 0.002346651 | 1 | 0.76149608 | 1 |
| 230999_at | FLJ39051 | hypothetical gene supported by AK096370 | 0.002352461 | 0.919530297 | 0.76149608 | 0.757924823 |
| 226946_at | C5orf33 | chromosome 5 open reading frame 33 | 0.002354915 | 1 | 0.76149608 | 0.757924823 |
| 242998_at | RDH12 | retinol dehydrogenase 12 (all-trans/9-cis/11-cis) | 0.002383777 | 1 | 0.76149608 | 1 |
| 1555551_at | SERPINB5 | serpin peptidase inhibitor, clade B (ovalbumin), member 5 | 0.002383777 | 1 | 0.76149608 | 1 |
| 210004_at | OLR1 | oxidized low density lipoprotein (lectin-like) receptor 1 | 0.002386552 | 1 | 0.039088634 | 1 |
| 1557352_at | SQLE | squalene epoxidase | 0.002392733 | 1 | 0.778100278 | 0.757924823 |
| 219275_at | PDCD5 | programmed cell death 5 | 0.002392733 | 1 | 0.76149608 | 1 |
| 238669_at | PTGS1 | prostaglandin-endoperoxide synthase 1 (prostaglandin G/H synthase and cyclooxygenase) | 0.002393127 | 1 | 0.76149608 | 1 |
| 203333_at | KIFAP3 | kinesin-associated protein 3 | 0.002393127 | 1 | 0.76149608 | 0.926714483 |
| 239650_at | NAP5 | Nck-associated protein 5 | 0.002406935 | 1 | 0.813357379 | 1 |
| 230051_at | C10orf47 | chromosome 10 open reading frame 47 | 0.00243489 | 1 | 0.76149608 | 1 |
| 1553976_a_at | RP11-529I10.4 | deleted in a mouse model of primary ciliary dyskinesia | 0.002443383 | 1 | 0.51993576 | 1 |
| 206667_s_at | SCAMP1 | secretory carrier membrane protein 1 | 0.002447494 | 1 | 0.76149608 | 1 |
| 236449_at | CSTB | cystatin B (stefin B) | 0.002447494 | 1 | 0.76149608 | 0.96628047 |
| 205768_s_at | SLC27A2 | solute carrier family 27 (fatty acid transporter), member 2 | 0.002450432 | 0.835232341 | 0.76149608 | 1 |
| 211362_s_at | SERPINB13 | serpin peptidase inhibitor, clade B (ovalbumin), member 13 | 0.002450432 | 1 | 0.76149608 | 1 |
| 224847_at | CDK6 | cyclin-dependent kinase 6 | 0.002453339 | 1 | 0.76149608 | 1 |
| 218883_s_at | MLF1IP | MLF1 interacting protein | 0.002454823 | 1 | 0.011232943 | 1 |
| 1552685_a_at | GRHL1 | grainyhead-like 1 (Drosophila) | 0.002463634 | 1 | 0.76149608 | 1 |
| 226302_at | ATP8B1 | ATPase, Class I, type 8B, member 1 | 0.002509511 | 1 | 0.76149608 | 1 |
| 222250_s_at | INTS7 | integrator complex subunit 7 | 0.00251079 | 1 | 0.76149608 | 1 |
| 216935_at | C1orf46 | chromosome 1 open reading frame 46 | 0.00251079 | 1 | 0.76149608 | 1 |
| 209719_x_at | SERPINB3 | serpin peptidase inhibitor, clade B (ovalbumin), member 3 | 0.002520362 | 1 | 0.522093853 | 1 |
| 243582_at | SH3RF2 | SH3 domain containing ring finger 2 | 0.002522125 | 1 | 0.178485405 | 1 |
| 224189_x_at | EHF | ets homologous factor | 0.002534309 | 1 | 0.76149608 | 1 |
| 211081_s_at | MAP4K5 | mitogen-activated protein kinase kinase kinase kinase 5 | 0.00257184 | 1 | 0.853051784 | 0.807620746 |
| 215800_at | DUOX1 | dual oxidase 1 | 0.002581306 | 1 | 0.76149608 | 1 |
| 211657_at | CEACAM6 | carcinoembryonic antigen-related cell adhesion molecule 6 (non-specific cross reacting antigen) | 0.002583275 | 1 | 0.76149608 | 1 |
| 1554628_at | ZNF57 | zinc finger protein 57 | 0.002583275 | 1 | 0.76149608 | 0.996556305 |
| 1557094_at | LOC653110 | hypothetical LOC653110 | 0.002584503 | 1 | 0.113999991 | 1 |
| 209018_s_at | PINK1 | PTEN induced putative kinase 1 | 0.002584739 | 1 | 0.76149608 | 0.835052221 |
| 203417_at | MFAP2 | microfibrillar-associated protein 2 | 0.002584739 | 1 | 0.001559489 | 1 |
| 218638_s_at | SPON2 | spondin 2, extracellular matrix protein | 0.002584739 | 1 | 0.168858754 | 1 |
| 1564307_a_at | A2ML1 | alpha-2-macroglobulin-like 1 | 0.002584739 | 1 | 0.76149608 | 1 |
| 222866_s_at | FLVCR2 | feline leukemia virus subgroup C cellular receptor family, member 2 | 0.002597676 | 1 | 0.653072047 | 1 |
| 210041_s_at | PGM3 | phosphoglucomutase 3 | 0.002600345 | 1 | 0.76149608 | 0.963499472 |
| 244692_at | CYP4F22 | cytochrome P450, family 4, subfamily F, polypeptide 22 | 0.002605342 | 1 | 0.76149608 | 1 |
| 222692_s_at | FNDC3B | fibronectin type III domain containing 3B | 0.002619813 | 1 | 0.000366502 | 0.757924823 |
| 224772_at | NAV1 | neuron navigator 1 | 0.002633971 | 1 | 0.76149608 | 0.771858165 |
| 207345_at | FST | follistatin | 0.00263892 | 1 | 0.76149608 | 1 |
| 204034_at | ETHE1 | ethylmalonic encephalopathy 1 | 0.002646452 | 1 | 0.572756439 | 1 |
| 227158_at | C14orf126 | chromosome 14 open reading frame 126 | 0.002646756 | 1 | 0.76149608 | 0.856503614 |
| 225998_at | GAB1 | GRB2-associated binding protein 1 | 0.002646756 | 1 | 0.76149608 | 0.818538188 |
| 221276_s_at | SYNC1 | syncoilin, intermediate filament 1 | 0.002647864 | 1 | 0.076915947 | 1 |
| 204969_s_at | RDX | radixin | 0.002653174 | 1 | 0.934854964 | 1 |
| 228873_at | COL22A1 | collagen, type XXII, alpha 1 | 0.002661775 | 0.835232341 | 0.76149608 | 0.955430351 |
| 205014_at | FGFBP1 | fibroblast growth factor binding protein 1 | 0.002665494 | 1 | 0.718585732 | 1 |
| 1556773_at | PTHLH | parathyroid hormone-like hormone | 0.002666901 | 1 | 0.76149608 | 1 |
| 205422_s_at | ITGBL1 | integrin, beta-like 1 (with EGF-like repeat domains) | 0.002672902 | 1 | 0.168630245 | 1 |
| 209859_at | TRIM9 | tripartite motif-containing 9 | 0.002693802 | 0.835232341 | 0.76149608 | 1 |
| 209228_x_at | TUSC3 | tumor suppressor candidate 3 | 0.002740244 | 1 | 0.768878283 | 0.960596745 |
| 219148_at | PBK | PDZ binding kinase | 0.002753993 | 1 | 0.76149608 | 1 |
| 204654_s_at | TFAP2A | transcription factor AP-2 alpha (activating enhancer binding protein 2 alpha) | 0.002764593 | 1 | 0.436987309 | 1 |
| 215723_s_at | PLD1 | phospholipase D1, phosphatidylcholine-specific | 0.002767652 | 1 | 0.76149608 | 1 |
| 1554406_a_at | CLEC7A | C-type lectin domain family 7, member A | 0.002786387 | 1 | 0.76149608 | 1 |
| 1556551_s_at | SLC39A6 | solute carrier family 39 (zinc transporter), member 6 | 0.002786387 | 1 | 0.76149608 | 1 |
| 1552575_a_at | C6orf141 | chromosome 6 open reading frame 141 | 0.002819984 | 1 | 0.76149608 | 0.757924823 |
| 218559_s_at | MAFB | v-maf musculoaponeurotic fibrosarcoma oncogene homolog B (avian) | 0.002831798 | 1 | 0.76149608 | 1 |
| 217930_s_at | TOLLIP | toll interacting protein | 0.002832488 | 1 | 0.76149608 | 1 |
| 219529_at | CLIC3 | chloride intracellular channel 3 | 0.002836542 | 1 | 0.76149608 | 1 |
| 211016_x_at | HSPA4 | heat shock 70kDa protein 4 | 0.002864137 | 1 | 0.76149608 | 1 |
| 212800_at | STX6 | syntaxin 6 | 0.002864137 | 1 | 0.76149608 | 1 |
| 219092_s_at | IPPK | inositol 1,3,4,5,6-pentakisphosphate 2-kinase | 0.002873585 | 1 | 0.76149608 | 1 |
| 206439_at | EPYC | epiphycan | 0.00287546 | 0.835232341 | 0.002261505 | 0.757924823 |
| 202431_s_at | MYC | v-myc myelocytomatosis viral oncogene homolog (avian) | 0.002875531 | 1 | 0.76149608 | 1 |
| 206033_s_at | DSC3 | desmocollin 3 | 0.002898318 | 1 | 0.685644826 | 1 |
| 219403_s_at | HPSE | heparanase | 0.002901452 | 1 | 0.76149608 | 1 |
| 209696_at | FBP1 | fructose-1,6-bisphosphatase 1 | 0.002911044 | 0.835232341 | 4.16E-05 | 0.757924823 |
| 230769_at | DENND2C | DENN/MADD domain containing 2C | 0.0029163 | 1 | 0.837417211 | 1 |
| 222979_s_at | SURF4 | surfeit 4 | 0.002931849 | 1 | 0.534479063 | 1 |
| 231240_at | DIO2 | deiodinase, iodothyronine, type II | 0.002950868 | 1 | 0.216423917 | 1 |
| 229518_at | FAM46B | family with sequence similarity 46, member B | 0.002955804 | 1 | 0.669554127 | 1 |
| 239648_at | DCUN1D3 | DCN1, defective in cullin neddylation 1, domain containing 3 (S. cerevisiae) | 0.002955804 | 1 | 0.76149608 | 1 |
| 241824_at | FOSL2 | FOS-like antigen 2 | 0.002955804 | 1 | 0.321932759 | 1 |
| 1554314_at | C6orf141 | chromosome 6 open reading frame 141 | 0.002955804 | 1 | 0.76149608 | 0.860569388 |
| 204679_at | KCNK1 | potassium channel, subfamily K, member 1 | 0.002971242 | 1 | 0.76149608 | 1 |
| 204256_at | ELOVL6 | ELOVL family member 6, elongation of long chain fatty acids (FEN1/Elo2, SUR4/Elo3-like, yeast) | 0.00298377 | 1 | 0.469139815 | 1 |
| 204451_at | FZD1 | frizzled homolog 1 (Drosophila) | 0.002984328 | 1 | 0.76149608 | 1 |
| 226537_at | HINT3 | histidine triad nucleotide binding protein 3 | 0.003008773 | 1 | 0.76149608 | 0.806628152 |
| 217764_s_at | RAB31 | RAB31, member RAS oncogene family | 0.00301984 | 1 | 0.029652562 | 1 |
| 228155_at | C10orf58 | chromosome 10 open reading frame 58 | 0.003034149 | 1 | 0.76149608 | 1 |
| 233565_s_at | SDCBP2 | syndecan binding protein (syntenin) 2 | 0.003044674 | 1 | 0.76149608 | 1 |
| 205349_at | GNA15 | guanine nucleotide binding protein (G protein), alpha 15 (Gq class) | 0.003069349 | 1 | 0.76149608 | 1 |
| 204678_s_at | KCNK1 | potassium channel, subfamily K, member 1 | 0.00308508 | 1 | 0.76149608 | 1 |
| 232449_at | BCDO2 | beta-carotene dioxygenase 2 | 0.003108315 | 1 | 0.847886617 | 0.757924823 |
| 227029_at | C14orf24 | chromosome 14 open reading frame 24 | 0.003124673 | 1 | 0.76149608 | 1 |
| 222262_s_at | ETNK1 | ethanolamine kinase 1 | 0.003124884 | 1 | 0.76149608 | 0.757924823 |
| 202154_x_at | TUBB3 | tubulin, beta 3 | 0.003133039 | 1 | 0.534479063 | 1 |
| 211506_s_at | IL8 | interleukin 8 | 0.003146849 | 1 | 0.76149608 | 1 |
| 231098_at | NA | NA | 0.003146938 | 0.893377707 | 0.76149608 | 1 |
| 214290_s_at | HIST2H2AA4 | histone cluster 2, H2aa4 | 0.003146938 | 1 | 0.007461286 | 0.875370307 |
| 229385_s_at | PLAC2 | placenta-specific 2 | 0.003158101 | 1 | 0.362624333 | 1 |
| 219953_s_at | C11orf17 | chromosome 11 open reading frame 17 | 0.003171144 | 1 | 0.76149608 | 1 |
| 226275_at | MXD1 | MAX dimerization protein 1 | 0.003174952 | 1 | 0.76149608 | 1 |
| 205724_at | PKP1 | plakophilin 1 (ectodermal dysplasia/skin fragility syndrome) | 0.003192217 | 1 | 0.76149608 | 1 |
| 206653_at | POLR3G | polymerase (RNA) III (DNA directed) polypeptide G (32kD) | 0.003207289 | 1 | 0.76149608 | 1 |
| 215165_x_at | UMPS | uridine monophosphate synthetase (orotate phosphoribosyl transferase and orotidine-5'-decarboxylase) | 0.003212574 | 1 | 0.76149608 | 0.969503583 |
| 209238_at | STX3 | syntaxin 3 | 0.003222748 | 1 | 0.76149608 | 0.903409005 |
| 1555292_at | FAM40B | family with sequence similarity 40, member B | 0.003230554 | 1 | 0.76149608 | 0.760255174 |
| 206307_s_at | FOXD1 | forkhead box D1 | 0.00323929 | 1 | 0.76149608 | 1 |
| 217892_s_at | LIMA1 | LIM domain and actin binding 1 | 0.003250357 | 1 | 0.76149608 | 1 |
| 229152_at | C4orf7 | chromosome 4 open reading frame 7 | 0.003278102 | 0.835232341 | 0.918141091 | 0.757924823 |
| 57703_at | SENP5 | SUMO1/sentrin specific peptidase 5 | 0.003278102 | 1 | 0.417492144 | 0.757924823 |
| 204952_at | LYPD3 | LY6/PLAUR domain containing 3 | 0.003278102 | 1 | 0.76149608 | 1 |
| 203973_s_at | CEBPD | CCAAT/enhancer binding protein (C/EBP), delta | 0.00328256 | 1 | 0.76149608 | 1 |
| 204351_at | S100P | S100 calcium binding protein P | 0.003288906 | 1 | 0.207066522 | 1 |
| 203074_at | ANXA8L2 | annexin A8-like 2 | 0.003299899 | 1 | 0.237796749 | 1 |
| 207565_s_at | MR1 | major histocompatibility complex, class I-related | 0.003300674 | 0.835232341 | 0.76149608 | 1 |
| 218550_s_at | LRRC20 | leucine rich repeat containing 20 | 0.003324457 | 1 | 0.76149608 | 0.768533603 |
| 203639_s_at | FGFR2 | fibroblast growth factor receptor 2 (bacteria-expressed kinase, keratinocyte growth factor receptor, craniofacial dysostosis 1, Crouzon syndrome, Pfeiffer syndrome, Jackson-Weiss syndrome) | 0.003378581 | 1 | 0.76149608 | 1 |
| 217031_at | KRT84 | keratin 84 | 0.003379619 | 0.997172739 | 0.76149608 | 0.757924823 |
| 206043_s_at | ATP2C2 | ATPase, Ca++ transporting, type 2C, member 2 | 0.003383036 | 1 | 0.76149608 | 1 |
| 225667_s_at | FAM84A | family with sequence similarity 84, member A | 0.003383036 | 1 | 0.880588938 | 1 |
| 1552619_a_at | ANLN | anillin, actin binding protein | 0.003396928 | 1 | 0.215338337 | 1 |
| 226247_at | PLEKHA1 | pleckstrin homology domain containing, family A (phosphoinositide binding specific) member 1 | 0.003396928 | 1 | 0.76149608 | 0.982022523 |
| 202035_s_at | SFRP1 | secreted frizzled-related protein 1 | 0.003402685 | 1 | 0.886333508 | 1 |
| 218686_s_at | RHBDF1 | rhomboid 5 homolog 1 (Drosophila) | 0.003421519 | 1 | 0.76149608 | 1 |
| 208156_x_at | EPPK1 | epiplakin 1 | 0.003426935 | 1 | 0.76149608 | 0.977010558 |
| 227542_at | SOCS6 | suppressor of cytokine signaling 6 | 0.003426989 | 1 | 0.76149608 | 1 |
| 218399_s_at | CDCA4 | cell division cycle associated 4 | 0.003446692 | 1 | 0.76149608 | 1 |
| 212115_at | HN1L | hematological and neurological expressed 1-like | 0.003447983 | 1 | 0.76149608 | 1 |
| 243938_x_at | DNAH5 | dynein, axonemal, heavy chain 5 | 0.003455983 | 1 | 0.76149608 | 1 |
| 202013_s_at | EXT2 | exostoses (multiple) 2 | 0.003477379 | 1 | 0.76149608 | 1 |
| 227371_at | BAIAP2L1 | BAI1-associated protein 2-like 1 | 0.003489534 | 1 | 0.58841161 | 0.993907079 |
| 203592_s_at | FSTL3 | follistatin-like 3 (secreted glycoprotein) | 0.003492096 | 1 | 0.76149608 | 1 |
| 223457_at | COPG2 | coatomer protein complex, subunit gamma 2 | 0.003500321 | 1 | 0.76149608 | 1 |
| 208702_x_at | APLP2 | amyloid beta (A4) precursor-like protein 2 | 0.003530264 | 1 | 0.615196664 | 1 |
| 228038_at | SOX2 | SRY (sex determining region Y)-box 2 | 0.003535029 | 1 | 0.76149608 | 1 |
| 211056_s_at | SRD5A1 | steroid-5-alpha-reductase, alpha polypeptide 1 (3-oxo-5 alpha-steroid delta 4-dehydrogenase alpha 1) | 0.003535029 | 1 | 0.76149608 | 1 |
| 205863_at | S100A12 | S100 calcium binding protein A12 | 0.003535029 | 1 | 0.400684144 | 1 |
| 223095_at | MARVELD1 | MARVEL domain containing 1 | 0.003550069 | 1 | 0.76149608 | 0.977958181 |
| 220030_at | STYK1 | serine/threonine/tyrosine kinase 1 | 0.003563685 | 1 | 0.76149608 | 0.757924823 |
| 223658_at | KCNK6 | potassium channel, subfamily K, member 6 | 0.003575863 | 1 | 0.718514007 | 0.992233669 |
| 219330_at | VANGL1 | vang-like 1 (van gogh, Drosophila) | 0.00361189 | 1 | 0.76149608 | 1 |
| 201379_s_at | TPD52L2 | tumor protein D52-like 2 | 0.003618182 | 1 | 0.291592994 | 1 |
| 244758_at | ZNF452 | zinc finger protein 452 | 0.003618182 | 1 | 0.76149608 | 0.814368849 |
| 228559_at | CENPN | centromere protein N | 0.003618182 | 1 | 0.76149608 | 1 |
| 227080_at | ZNF697 | zinc finger protein 697 | 0.003626274 | 1 | 0.447383644 | 1 |
| 214927_at | ITGBL1 | integrin, beta-like 1 (with EGF-like repeat domains) | 0.00364843 | 0.835232341 | 0.001748613 | 0.967531894 |
| 223574_x_at | PPP2R2C | protein phosphatase 2 (formerly 2A), regulatory subunit B, gamma isoform | 0.003649698 | 1 | 0.688291505 | 0.93970461 |
| 211126_s_at | CSRP2 | cysteine and glycine-rich protein 2 | 0.00366685 | 1 | 0.846161125 | 0.757924823 |
| 208425_s_at | TANC2 | tetratricopeptide repeat, ankyrin repeat and coiled-coil containing 2 | 0.00367723 | 1 | 0.76149608 | 1 |
| 218516_s_at | IMPAD1 | inositol monophosphatase domain containing 1 | 0.003688216 | 1 | 0.76149608 | 1 |
| 203324_s_at | CAV2 | caveolin 2 | 0.003696189 | 1 | 0.277244248 | 1 |
| 222937_s_at | MMP28 | matrix metallopeptidase 28 | 0.003701613 | 1 | 0.76149608 | 0.757924823 |
| 233255_s_at | BIVM | basic, immunoglobulin-like variable motif containing | 0.00370968 | 1 | 0.76149608 | 0.905958698 |
| 222118_at | CENPN | centromere protein N | 0.00370968 | 1 | 0.76149608 | 1 |
| 222257_s_at | ACE2 | angiotensin I converting enzyme (peptidyl-dipeptidase A) 2 | 0.00370968 | 1 | 0.76149608 | 0.758640508 |
| 230508_at | DKK3 | dickkopf homolog 3 (Xenopus laevis) | 0.00370968 | 1 | 0.76149608 | 1 |
| 231439_at | FAM84A | family with sequence similarity 84, member A | 0.0038086 | 1 | 0.81780971 | 1 |
| 217002_s_at | HTR3A | 5-hydroxytryptamine (serotonin) receptor 3A | 0.003827569 | 1 | 0.76149608 | 0.757924823 |
| 226374_at | CXADR | coxsackie virus and adenovirus receptor | 0.003845755 | 1 | 0.76149608 | 1 |
| 219010_at | C1orf106 | chromosome 1 open reading frame 106 | 0.003848549 | 1 | 0.386841025 | 1 |
| 214841_at | CNIH3 | cornichon homolog 3 (Drosophila) | 0.003882267 | 0.862438926 | 0.76149608 | 0.814164315 |
| 229582_at | C18orf37 | chromosome 18 open reading frame 37 | 0.003923278 | 1 | 0.76149608 | 0.757924823 |
| 205219_s_at | GALK2 | galactokinase 2 | 0.003956039 | 1 | 0.76149608 | 0.895389412 |
| 213790_at | ADAM12 | ADAM metallopeptidase domain 12 (meltrin alpha) | 0.003980817 | 1 | 0.001131936 | 1 |
| 206032_at | DSC3 | desmocollin 3 | 0.004006112 | 1 | 0.718269333 | 1 |
| 226027_at | C9orf119 | chromosome 9 open reading frame 119 | 0.004031522 | 1 | 0.76149608 | 1 |
| 211607_x_at | EGFR | epidermal growth factor receptor (erythroblastic leukemia viral (v-erb-b) oncogene homolog, avian) | 0.004046182 | 1 | 0.76149608 | 1 |
| 205122_at | TMEFF1 | transmembrane protein with EGF-like and two follistatin-like domains 1 | 0.004063756 | 1 | 0.329584847 | 1 |
| 203180_at | ALDH1A3 | aldehyde dehydrogenase 1 family, member A3 | 0.004068888 | 1 | 0.76149608 | 1 |
| 203100_s_at | CDYL | chromodomain protein, Y-like | 0.004077445 | 1 | 0.76149608 | 0.833406891 |
| 218854_at | DSE | dermatan sulfate epimerase | 0.004077445 | 1 | 0.586408931 | 1 |
| 226960_at | UNQ473 | DMC | 0.00408476 | 1 | 0.76149608 | 0.771000642 |
| 226955_at | AFAP1L1 | actin filament associated protein 1-like 1 | 0.004103502 | 1 | 0.76149608 | 0.757924823 |
| 1560587_s_at | PRDX5 | peroxiredoxin 5 | 0.004104124 | 1 | 0.66971651 | 1 |
| 1569410_at | RP1-14N1.3 | filaggrin 2 | 0.004104145 | 1 | 0.76149608 | 1 |
| 242100_at | CHSY-2 | chondroitin synthase-2 | 0.004117139 | 1 | 0.76149608 | 0.99261454 |
| 201219_at | ZRANB1 | zinc finger, RAN-binding domain containing 1 | 0.004175753 | 1 | 0.76149608 | 1 |
| 210582_s_at | LIMK2 | LIM domain kinase 2 | 0.004193644 | 1 | 0.76149608 | 1 |
| 242123_at | PAQR7 | progestin and adipoQ receptor family member VII | 0.004195057 | 0.920369745 | 0.76149608 | 0.986016806 |
| 231849_at | KRT80 | keratin 80 | 0.004195057 | 1 | 0.76149608 | 1 |
| 217272_s_at | SERPINB13 | serpin peptidase inhibitor, clade B (ovalbumin), member 13 | 0.004198141 | 1 | 0.76149608 | 1 |
| 223193_x_at | C3orf28 | chromosome 3 open reading frame 28 | 0.004219183 | 1 | 0.76149608 | 1 |
| 224710_at | RAB34 | RAB34, member RAS oncogene family | 0.004232776 | 1 | 0.384230819 | 1 |
| 201611_s_at | ICMT | isoprenylcysteine carboxyl methyltransferase | 0.004232776 | 1 | 0.76149608 | 1 |
| 207630_s_at | CREM | cAMP responsive element modulator | 0.004232776 | 1 | 0.76149608 | 1 |
| 210255_at | RAD51L1 | RAD51-like 1 (S. cerevisiae) | 0.004240658 | 1 | 0.76149608 | 0.76414244 |
| 204751_x_at | DSC2 | desmocollin 2 | 0.004301881 | 1 | 0.76149608 | 1 |
| 218235_s_at | UTP11L | UTP11-like, U3 small nucleolar ribonucleoprotein, (yeast) | 0.004303718 | 1 | 0.76149608 | 1 |
| 204469_at | PTPRZ1 | protein tyrosine phosphatase, receptor-type, Z polypeptide 1 | 0.004313356 | 1 | 0.76149608 | 1 |
| 241981_at | FAM20A | family with sequence similarity 20, member A | 0.004325548 | 1 | 0.76149608 | 1 |
| 226282_at | NA | NA | 0.004325628 | 1 | 0.76149608 | 1 |
| 229004_at | NA | NA | 0.004334173 | 1 | 0.286268188 | 1 |
| 207342_at | CNGB1 | cyclic nucleotide gated channel beta 1 | 0.004339959 | 1 | 0.76149608 | 0.975696812 |
| 206561_s_at | AKR1B10 | aldo-keto reductase family 1, member B10 (aldose reductase) | 0.004351858 | 1 | 0.536933332 | 1 |
| 223381_at | NUF2 | NUF2, NDC80 kinetochore complex component, homolog (S. cerevisiae) | 0.004355575 | 1 | 0.379422915 | 1 |
| 238542_at | ULBP2 | UL16 binding protein 2 | 0.004373215 | 1 | 0.646844368 | 1 |
| 224262_at | IL1F10 | interleukin 1 family, member 10 (theta) | 0.004377441 | 1 | 0.76149608 | 0.874086803 |
| 235016_at | REEP3 | receptor accessory protein 3 | 0.004392208 | 1 | 0.76149608 | 1 |
| 228441_s_at | PET112L | PET112-like (yeast) | 0.004399143 | 1 | 0.76149608 | 1 |
| 219121_s_at | RBM35A | RNA binding motif protein 35A | 0.004424922 | 1 | 0.409700523 | 1 |
| 41660_at | CELSR1 | cadherin, EGF LAG seven-pass G-type receptor 1 (flamingo homolog, Drosophila) | 0.004428221 | 1 | 0.76149608 | 0.845290108 |
| 1554921_a_at | SCEL | sciellin | 0.004428607 | 1 | 0.76149608 | 1 |
| 241946_at | ZDHHC21 | zinc finger, DHHC-type containing 21 | 0.004429002 | 1 | 0.819320453 | 1 |
| 212514_x_at | DDX3X | DEAD (Asp-Glu-Ala-Asp) box polypeptide 3, X-linked | 0.004430503 | 1 | 0.76149608 | 0.992031629 |
| 91816_f_at | MEX3D | mex-3 homolog D (C. elegans) | 0.004437218 | 1 | 0.730967235 | 1 |
| 203797_at | VSNL1 | visinin-like 1 | 0.004437623 | 1 | 0.76149608 | 1 |
| 225766_s_at | TNPO1 | transportin 1 | 0.004453275 | 1 | 0.208917516 | 0.979021396 |
| 222651_s_at | TRPS1 | trichorhinophalangeal syndrome I | 0.00445574 | 1 | 0.76149608 | 1 |
| 200983_x_at | CD59 | CD59 molecule, complement regulatory protein | 0.00445574 | 1 | 0.76149608 | 1 |
| 201839_s_at | TACSTD1 | tumor-associated calcium signal transducer 1 | 0.004471711 | 1 | 0.269394097 | 1 |
| 223991_s_at | GALNT2 | UDP-N-acetyl-alpha-D-galactosamine:polypeptide N-acetylgalactosaminyltransferase 2 (GalNAc-T2) | 0.00447947 | 1 | 0.010728709 | 1 |
| 202893_at | UNC13B | unc-13 homolog B (C. elegans) | 0.004489051 | 1 | 0.76149608 | 0.953679392 |
| 1568765_at | SERPINE1 | serpin peptidase inhibitor, clade E (nexin, plasminogen activator inhibitor type 1), member 1 | 0.004502668 | 1 | 0.046290975 | 0.808626259 |
| 231311_at | NA | NA | 0.004508919 | 1 | 0.76149608 | 1 |
| 202450_s_at | CTSK | cathepsin K | 0.004519767 | 1 | 0.008404614 | 1 |
| 201136_at | PLP2 | proteolipid protein 2 (colonic epithelium-enriched) | 0.004524429 | 1 | 0.76149608 | 1 |
| 211372_s_at | IL1R2 | interleukin 1 receptor, type II | 0.004527748 | 1 | 0.76149608 | 1 |
| 205542_at | STEAP1 | six transmembrane epithelial antigen of the prostate 1 | 0.004541675 | 1 | 0.039795243 | 1 |
| 220988_s_at | C1QTNF3 | C1q and tumor necrosis factor related protein 3 | 0.004563871 | 0.835232341 | 0.76149608 | 0.893873761 |
| 228931_at | COQ4 | coenzyme Q4 homolog (S. cerevisiae) | 0.004563871 | 0.989198477 | 0.76149608 | 1 |
| 209853_s_at | PSME3 | proteasome (prosome, macropain) activator subunit 3 (PA28 gamma; Ki) | 0.004563871 | 1 | 0.76149608 | 1 |
| 220187_at | STEAP4 | STEAP family member 4 | 0.004581023 | 1 | 0.872252421 | 1 |
| 218118_s_at | TIMM23 | translocase of inner mitochondrial membrane 23 homolog (yeast) | 0.004597573 | 1 | 0.419661827 | 1 |
| 218582_at | MARCH5 | membrane-associated ring finger (C3HC4) 5 | 0.00461592 | 1 | 0.76149608 | 1 |
| 212396_s_at | KIAA0090 | KIAA0090 | 0.00461592 | 1 | 0.76149608 | 1 |
| 207891_s_at | UCHL5IP | UCHL5 interacting protein | 0.004654624 | 0.867739398 | 0.76149608 | 0.757924823 |
| 229441_at | PRSS23 | protease, serine, 23 | 0.004667426 | 1 | 0.232796818 | 1 |
| 242397_at | OLR1 | oxidized low density lipoprotein (lectin-like) receptor 1 | 0.004682998 | 1 | 0.007465617 | 0.961494813 |
| 208899_x_at | ATP6V1D | ATPase, H+ transporting, lysosomal 34kDa, V1 subunit D | 0.004706786 | 1 | 0.387759748 | 1 |
| 236738_at | LOC401097 | Similar to LOC166075 | 0.004706786 | 1 | 0.188476307 | 1 |
| 226483_at | TMEM68 | transmembrane protein 68 | 0.004706786 | 1 | 0.76149608 | 0.845456323 |
| 229933_at | C1orf74 | chromosome 1 open reading frame 74 | 0.004709566 | 1 | 0.76149608 | 1 |
| 231055_at | NA | NA | 0.004712054 | 1 | 0.76149608 | 0.93970461 |
| 220230_s_at | CYB5R2 | cytochrome b5 reductase 2 | 0.004733863 | 1 | 0.76149608 | 1 |
| 213425_at | WNT5A | wingless-type MMTV integration site family, member 5A | 0.004811304 | 1 | 0.76149608 | 1 |
| 204733_at | KLK6 | kallikrein-related peptidase 6 | 0.004821534 | 1 | 0.76149608 | 1 |
| 215294_s_at | SMARCA1 | SWI/SNF related, matrix associated, actin dependent regulator of chromatin, subfamily a, member 1 | 0.004827308 | 0.835232341 | 0.76149608 | 1 |
| 201105_at | LGALS1 | lectin, galactoside-binding, soluble, 1 (galectin 1) | 0.004848299 | 1 | 0.051747094 | 1 |
| 209344_at | TPM4 | tropomyosin 4 | 0.004897154 | 1 | 0.327652185 | 1 |
| 205503_at | PTPN14 | protein tyrosine phosphatase, non-receptor type 14 | 0.004939214 | 1 | 0.76149608 | 1 |
| 203221_at | TLE1 | transducin-like enhancer of split 1 (E(sp1) homolog, Drosophila) | 0.004990065 | 1 | 0.76149608 | 0.818517644 |
| 212445_s_at | NEDD4L | neural precursor cell expressed, developmentally down-regulated 4-like | 0.004990065 | 1 | 0.76149608 | 1 |
| 209101_at | CTGF | connective tissue growth factor | 0.005002069 | 1 | 0.003296848 | 1 |
| 201215_at | PLS3 | plastin 3 (T isoform) | 0.005014029 | 1 | 0.756683614 | 1 |
| 206091_at | MATN3 | matrilin 3 | 0.005015919 | 0.88071935 | 0.76149608 | 0.757924823 |
| 203187_at | DOCK1 | dedicator of cytokinesis 1 | 0.005048637 | 0.835232341 | 0.76149608 | 0.9909114 |
| 225615_at | LOC126917 | hypothetical protein LOC126917 | 0.005113665 | 1 | 0.76149608 | 1 |
| 236495_at | PBEF1 | pre-B-cell colony enhancing factor 1 | 0.005121209 | 1 | 0.850046297 | 1 |
| 200666_s_at | DNAJB1 | DnaJ (Hsp40) homolog, subfamily B, member 1 | 0.005131454 | 1 | 0.76149608 | 1 |
| 203215_s_at | MYO6 | myosin VI | 0.005138825 | 1 | 0.938900616 | 1 |
| 201120_s_at | PGRMC1 | progesterone receptor membrane component 1 | 0.005138825 | 1 | 0.76149608 | 1 |
| 227032_at | PLXNA2 | plexin A2 | 0.005141239 | 1 | 0.76149608 | 0.883219436 |
| 211382_s_at | TACC2 | transforming, acidic coiled-coil containing protein 2 | 0.005146173 | 1 | 0.76149608 | 1 |
| 36711_at | MAFF | v-maf musculoaponeurotic fibrosarcoma oncogene homolog F (avian) | 0.005186397 | 1 | 0.574986324 | 1 |
| 225829_at | PDZD8 | PDZ domain containing 8 | 0.005187794 | 1 | 0.76149608 | 1 |
| 201897_s_at | CKS1B | CDC28 protein kinase regulatory subunit 1B | 0.005187794 | 1 | 0.76149608 | 1 |
| 207813_s_at | FDXR | ferredoxin reductase | 0.005187794 | 1 | 0.76149608 | 1 |
| 227925_at | FLJ39051 | hypothetical gene supported by AK096370 | 0.005195068 | 0.889636143 | 0.76149608 | 0.757924823 |
| 209502_s_at | BAIAP2 | BAI1-associated protein 2 | 0.005195068 | 1 | 0.76149608 | 1 |
| 203989_x_at | F2R | coagulation factor II (thrombin) receptor | 0.005207385 | 1 | 0.039899679 | 1 |
| 222702_x_at | CRIPT | cysteine-rich PDZ-binding protein | 0.005211834 | 1 | 0.76149608 | 1 |
| 219995_s_at | ZNF750 | zinc finger protein 750 | 0.005225894 | 1 | 0.76149608 | 1 |
| 210861_s_at | WISP3 | WNT1 inducible signaling pathway protein 3 | 0.005251031 | 1 | 0.124437412 | 1 |
| 203735_x_at | PPFIBP1 | PTPRF interacting protein, binding protein 1 (liprin beta 1) | 0.005253057 | 1 | 0.76149608 | 1 |
| 204470_at | CXCL1 | chemokine (C-X-C motif) ligand 1 (melanoma growth stimulating activity, alpha) | 0.005257098 | 1 | 0.107341676 | 1 |
| 219369_s_at | OTUB2 | OTU domain, ubiquitin aldehyde binding 2 | 0.005257098 | 1 | 0.76149608 | 1 |
| 240512_x_at | KCTD4 | potassium channel tetramerisation domain containing 4 | 0.005260539 | 0.976717895 | 0.761976763 | 0.909567895 |
| 218553_s_at | KCTD15 | potassium channel tetramerisation domain containing 15 | 0.005297332 | 1 | 0.76149608 | 1 |
| 222173_s_at | TBC1D2 | TBC1 domain family, member 2 | 0.005300899 | 1 | 0.76149608 | 1 |
| 219944_at | CLIP4 | CAP-GLY domain containing linker protein family, member 4 | 0.005300899 | 1 | 0.76149608 | 1 |
| 221100_at | C6orf15 | chromosome 6 open reading frame 15 | 0.00533452 | 1 | 0.76149608 | 0.757924823 |
| 235683_at | SESN3 | sestrin 3 | 0.005340545 | 1 | 0.76149608 | 0.757924823 |
| 225186_at | RAPH1 | Ras association (RalGDS/AF-6) and pleckstrin homology domains 1 | 0.005342259 | 1 | 0.76149608 | 1 |
| 226697_at | FAM114A1 | family with sequence similarity 114, member A1 | 0.005342862 | 1 | 0.100602575 | 1 |
| 210480_s_at | MYO6 | myosin VI | 0.00537109 | 1 | 0.76149608 | 1 |
| 239089_at | NA | NA | 0.005384298 | 1 | 0.76149608 | 0.957587496 |
| 218739_at | ABHD5 | abhydrolase domain containing 5 | 0.005390004 | 1 | 0.76149608 | 1 |
| 205393_s_at | CHEK1 | CHK1 checkpoint homolog (S. pombe) | 0.005394444 | 1 | 0.76149608 | 1 |
| 202859_x_at | IL8 | interleukin 8 | 0.005485059 | 1 | 0.76149608 | 1 |
| 225258_at | FBLIM1 | filamin binding LIM protein 1 | 0.005498444 | 1 | 0.76149608 | 1 |
| 220254_at | LRP12 | low density lipoprotein-related protein 12 | 0.005520614 | 1 | 0.76149608 | 1 |
| 223660_at | ADORA3 | adenosine A3 receptor | 0.005528105 | 1 | 0.76149608 | 0.760255174 |
| 218627_at | DRAM | damage-regulated autophagy modulator | 0.005528105 | 1 | 0.5244582 | 1 |
| 227880_s_at | TMEM185A | transmembrane protein 185A | 0.005547072 | 1 | 0.76149608 | 0.757924823 |
| 212658_at | LHFPL2 | lipoma HMGIC fusion partner-like 2 | 0.005547802 | 1 | 0.007698621 | 1 |
| 235272_at | SBSN | suprabasin | 0.005555707 | 1 | 0.76149608 | 1 |
| 222994_at | PRDX5 | peroxiredoxin 5 | 0.005563606 | 1 | 0.748090709 | 1 |
| 207291_at | PRRG4 | proline rich Gla (G-carboxyglutamic acid) 4 (transmembrane) | 0.005593544 | 1 | 0.564616942 | 1 |
| 202037_s_at | SFRP1 | secreted frizzled-related protein 1 | 0.005593544 | 1 | 1 | 1 |
| 218027_at | MRPL15 | mitochondrial ribosomal protein L15 | 0.005599988 | 1 | 0.222489947 | 1 |
| 203699_s_at | DIO2 | deiodinase, iodothyronine, type II | 0.005605771 | 1 | 0.059812998 | 1 |
| 223204_at | C4orf18 | chromosome 4 open reading frame 18 | 0.005616083 | 1 | 0.557070274 | 1 |
| 224534_at | KREMEN1 | kringle containing transmembrane protein 1 | 0.005616083 | 1 | 0.76149608 | 1 |
| 91684_g_at | EXOSC4 | exosome component 4 | 0.005650862 | 1 | 0.76149608 | 0.958332253 |
| 205538_at | CORO2A | coronin, actin binding protein, 2A | 0.005707415 | 1 | 0.76149608 | 0.766118386 |
| 228481_at | POSTN | periostin, osteoblast specific factor | 0.005740693 | 1 | 0.000138182 | 1 |
| 204597_x_at | STC1 | stanniocalcin 1 | 0.005742876 | 1 | 0.552589356 | 0.978780674 |
| 222899_at | ITGA11 | integrin, alpha 11 | 0.005747227 | 1 | 0.198149798 | 1 |
| 223454_at | CXCL16 | chemokine (C-X-C motif) ligand 16 | 0.00576418 | 1 | 0.76149608 | 1 |
| 211864_s_at | FER1L3 | fer-1-like 3, myoferlin (C. elegans) | 0.00576418 | 1 | 0.76149608 | 1 |
| 210233_at | IL1RAP | interleukin 1 receptor accessory protein | 0.005767153 | 1 | 0.552557583 | 1 |
| 233518_at | LOC654342 | similar to lymphocyte-specific protein 1 | 0.005799087 | 0.839448123 | 0.000199317 | 0.998671302 |
| 224997_x_at | H19 | H19, imprinted maternally expressed untranslated mRNA | 0.005817025 | 0.948189352 | 0.517467477 | 0.817659794 |
| 221867_at | N4BP1 | Nedd4 binding protein 1 | 0.005820635 | 1 | 0.76149608 | 0.945993514 |
| 205443_at | SNAPC1 | small nuclear RNA activating complex, polypeptide 1, 43kDa | 0.005835747 | 1 | 0.76149608 | 0.90117115 |
| 210978_s_at | TAGLN2 | transgelin 2 | 0.005859207 | 1 | 0.175912647 | 1 |
| 217849_s_at | CDC42BPB | CDC42 binding protein kinase beta (DMPK-like) | 0.005891484 | 1 | 0.081468872 | 0.967226795 |
| 1559254_at | C21orf113 | chromosome 21 open reading frame 113 | 0.00594891 | 0.976619693 | 0.10563304 | 0.970695426 |
| 207710_at | LCE2B | late cornified envelope 2B | 0.005968899 | 1 | 0.76149608 | 1 |
| 219305_x_at | FBXO2 | F-box protein 2 | 0.005971652 | 0.855776939 | 0.477789132 | 0.902148875 |
| 211980_at | COL4A1 | collagen, type IV, alpha 1 | 0.005982854 | 1 | 0.000555615 | 1 |
| 209304_x_at | GADD45B | growth arrest and DNA-damage-inducible, beta | 0.005991878 | 0.867099309 | 0.76149608 | 0.757924823 |
| 203595_s_at | IFIT5 | interferon-induced protein with tetratricopeptide repeats 5 | 0.005991878 | 0.950878263 | 0.76149608 | 1 |
| 32088_at | BLZF1 | basic leucine zipper nuclear factor 1 (JEM-1) | 0.005991878 | 1 | 0.76149608 | 0.96653369 |
| 205099_s_at | CCR1 | chemokine (C-C motif) receptor 1 | 0.005999951 | 1 | 0.003481677 | 1 |
| 220060_s_at | C12orf48 | chromosome 12 open reading frame 48 | 0.006007259 | 1 | 0.76149608 | 1 |
| 222809_x_at | C14orf65 | chromosome 14 open reading frame 65 | 0.006024804 | 1 | 0.76149608 | 1 |
| 215150_at | YOD1 | YOD1 OTU deubiquinating enzyme 1 homolog (S. cerevisiae) | 0.006027083 | 1 | 0.76149608 | 1 |
| 221477_s_at | MGC5618 | hypothetical protein MGC5618 | 0.006039683 | 1 | 0.011331273 | 1 |
| 228485_s_at | SLC44A1 | solute carrier family 44, member 1 | 0.006101212 | 1 | 0.76149608 | 0.938034533 |
| 219523_s_at | ODZ3 | odz, odd Oz/ten-m homolog 3 (Drosophila) | 0.006169874 | 0.995926896 | 0.408488669 | 1 |
| 209873_s_at | PKP3 | plakophilin 3 | 0.006178542 | 1 | 0.76149608 | 1 |
| 203715_at | TBCE | tubulin folding cofactor E | 0.006207846 | 0.848157865 | 0.76149608 | 0.757924823 |
| 209168_at | GPM6B | glycoprotein M6B | 0.006207846 | 1 | 0.76149608 | 1 |
| 201262_s_at | BGN | biglycan | 0.006234132 | 1 | 0.168134066 | 0.93567734 |
| 218412_s_at | GTF2IRD1 | GTF2I repeat domain containing 1 | 0.006234295 | 1 | 0.76149608 | 1 |
| 203628_at | IGF1R | insulin-like growth factor 1 receptor | 0.006239404 | 0.835232341 | 0.773333604 | 0.902584394 |
| 229479_at | LOC646324 | hypothetical LOC646324 | 0.006282612 | 0.954505458 | 0.004764854 | 0.757924823 |
| 203230_at | DVL1 | dishevelled, dsh homolog 1 (Drosophila) | 0.006282612 | 1 | 0.676961378 | 0.905425721 |
| 226658_at | PDPN | podoplanin | 0.006342139 | 1 | 0.160075989 | 1 |
| 203430_at | HEBP2 | heme binding protein 2 | 0.006342574 | 1 | 0.76149608 | 1 |
| 209727_at | GM2A | GM2 ganglioside activator | 0.006379233 | 1 | 0.76149608 | 0.915793116 |
| 1560531_at | LCE1B | late cornified envelope 1B | 0.006432943 | 1 | 0.76149608 | 1 |
| 1557938_s_at | PTRF | polymerase I and transcript release factor | 0.006447202 | 1 | 0.76149608 | 1 |
| 219377_at | FAM59A | family with sequence similarity 59, member A | 0.006454349 | 1 | 0.76149608 | 1 |
| 222463_s_at | BACE1 | beta-site APP-cleaving enzyme 1 | 0.006460819 | 1 | 0.427910767 | 1 |
| 206364_at | KIF14 | kinesin family member 14 | 0.006460819 | 1 | 0.70166502 | 1 |
| 214549_x_at | SPRR1A | small proline-rich protein 1A | 0.006463157 | 1 | 0.76149608 | 1 |
| 200916_at | TAGLN2 | transgelin 2 | 0.006463157 | 1 | 0.032031878 | 1 |
| 222039_at | LOC146909 | hypothetical protein LOC146909 | 0.006473878 | 1 | 0.32537301 | 1 |
| 207117_at | ZNF117 | zinc finger protein 117 | 0.006473878 | 1 | 0.199133506 | 0.757924823 |
| 202008_s_at | NID1 | nidogen 1 | 0.006480779 | 1 | 0.76149608 | 1 |
| 232254_at | FBXO25 | F-box protein 25 | 0.006486955 | 1 | 0.76149608 | 0.798946092 |
| 206582_s_at | GPR56 | G protein-coupled receptor 56 | 0.006486955 | 1 | 0.76149608 | 0.882467023 |
| 207121_s_at | MAPK6 | mitogen-activated protein kinase 6 | 0.006493311 | 1 | 0.76149608 | 1 |
| 206972_s_at | GPR161 | G protein-coupled receptor 161 | 0.00650088 | 0.835232341 | 0.76149608 | 0.757924823 |
| 209454_s_at | TEAD3 | TEA domain family member 3 | 0.006513903 | 1 | 0.76149608 | 1 |
| 1553454_at | RPTN | repetin | 0.006535242 | 1 | 0.76149608 | 1 |
| 213001_at | ANGPTL2 | angiopoietin-like 2 | 0.006546792 | 1 | 0.213805381 | 1 |
| 231778_at | DLX3 | distal-less homeobox 3 | 0.006546792 | 1 | 0.76149608 | 1 |
| 1555742_at | NA | NA | 0.006546792 | 1 | 0.76149608 | 0.903182309 |
| 236044_at | PPAPDC1A | phosphatidic acid phosphatase type 2 domain containing 1A | 0.00656846 | 1 | 0.197571389 | 0.885671855 |
| 204601_at | N4BP1 | Nedd4 binding protein 1 | 0.006591093 | 1 | 0.76149608 | 1 |
| 204825_at | MELK | maternal embryonic leucine zipper kinase | 0.006600345 | 1 | 0.76149608 | 1 |
| 213848_at | NA | NA | 0.006621615 | 1 | 0.76149608 | 1 |
| 206605_at | P11 | 26 serine protease | 0.006622667 | 1 | 0.76149608 | 1 |
| 230972_at | ANKRD9 | ankyrin repeat domain 9 | 0.006671163 | 1 | 0.76149608 | 1 |
| 242317_at | NA | NA | 0.006671163 | 1 | 0.76149608 | 1 |
| 224002_s_at | FKBP7 | FK506 binding protein 7 | 0.006710107 | 1 | 0.5244582 | 1 |
| 227942_s_at | CRIPT | cysteine-rich PDZ-binding protein | 0.006736761 | 1 | 0.76149608 | 0.982176884 |
| 202053_s_at | ALDH3A2 | aldehyde dehydrogenase 3 family, member A2 | 0.006744017 | 1 | 0.76149608 | 1 |
| 210138_at | RGS20 | regulator of G-protein signaling 20 | 0.006744017 | 1 | 0.346820034 | 1 |
| 217763_s_at | RAB31 | RAB31, member RAS oncogene family | 0.006753998 | 1 | 0.004914902 | 1 |
| 224329_s_at | CNFN | cornifelin | 0.006770588 | 1 | 0.55081196 | 1 |
| 201436_at | EIF4E | eukaryotic translation initiation factor 4E | 0.006770588 | 1 | 0.766956101 | 0.999688462 |
| 238584_at | IQCA | IQ motif containing with AAA domain | 0.006777342 | 1 | 0.76149608 | 0.816999078 |
| 226196_s_at | C14orf179 | chromosome 14 open reading frame 179 | 0.006794883 | 1 | 0.270384589 | 0.971871772 |
| 235924_at | NA | NA | 0.006796421 | 1 | 0.76149608 | 0.761941401 |
| 232080_at | HECW2 | HECT, C2 and WW domain containing E3 ubiquitin protein ligase 2 | 0.006796861 | 1 | 0.76149608 | 0.985635246 |
| 204967_at | SHROOM2 | shroom family member 2 | 0.006798252 | 1 | 0.174556601 | 1 |
| 227829_at | GYLTL1B | glycosyltransferase-like 1B | 0.0068203 | 1 | 0.76149608 | 0.757924823 |
| 208153_s_at | FAT2 | FAT tumor suppressor homolog 2 (Drosophila) | 0.006820468 | 1 | 0.385543041 | 1 |
| 219825_at | CYP26B1 | cytochrome P450, family 26, subfamily B, polypeptide 1 | 0.006881852 | 1 | 0.766806234 | 1 |
| 203535_at | S100A9 | S100 calcium binding protein A9 | 0.006881852 | 1 | 0.4357538 | 1 |
| 222848_at | CENPK | centromere protein K | 0.006881852 | 1 | 0.76149608 | 1 |
| 201537_s_at | DUSP3 | dual specificity phosphatase 3 (vaccinia virus phosphatase VH1-related) | 0.006881852 | 1 | 0.271952833 | 1 |
| 204565_at | THEM2 | thioesterase superfamily member 2 | 0.006882572 | 1 | 0.73719442 | 0.892272605 |
| 221696_s_at | STYK1 | serine/threonine/tyrosine kinase 1 | 0.006955455 | 1 | 0.76149608 | 0.824245111 |
| 204380_s_at | FGFR3 | fibroblast growth factor receptor 3 (achondroplasia, thanatophoric dwarfism) | 0.006976689 | 1 | 0.76149608 | 1 |
| 202575_at | CRABP2 | cellular retinoic acid binding protein 2 | 0.007006876 | 1 | 0.342973574 | 1 |
| 227642_at | TFCP2L1 | transcription factor CP2-like 1 | 0.007053253 | 1 | 0.76149608 | 1 |
| 212570_at | ENDOD1 | endonuclease domain containing 1 | 0.007064591 | 1 | 0.76149608 | 1 |
| 226621_at | FGG | fibrinogen gamma chain | 0.007064591 | 1 | 0.76149608 | 1 |
| 232170_at | S100A7A | S100 calcium binding protein A7A | 0.007073345 | 1 | 0.239863539 | 1 |
| 208083_s_at | ITGB6 | integrin, beta 6 | 0.007100009 | 1 | 0.111269936 | 1 |
| 209652_s_at | PGF | placental growth factor, vascular endothelial growth factor-related protein | 0.007122659 | 1 | 0.76149608 | 1 |
| 216685_s_at | MTAP | methylthioadenosine phosphorylase | 0.007123509 | 0.835232341 | 0.76149608 | 1 |
| 239433_at | LRRC8E | leucine rich repeat containing 8 family, member E | 0.007132658 | 1 | 0.76149608 | 1 |
| 213549_at | NA | NA | 0.007161712 | 1 | 0.76149608 | 1 |
| 219366_at | AVEN | apoptosis, caspase activation inhibitor | 0.007161712 | 1 | 0.76149608 | 0.853347338 |
| 206912_at | FOXE1 | forkhead box E1 (thyroid transcription factor 2) | 0.00716685 | 1 | 0.76149608 | 1 |
| 214649_s_at | MTMR2 | myotubularin related protein 2 | 0.00717323 | 1 | 0.76149608 | 1 |
| 238028_at | FLJ90086 | similar to AI661453 protein | 0.007184179 | 1 | 0.76149608 | 1 |
| 219420_s_at | C1orf163 | chromosome 1 open reading frame 163 | 0.007194813 | 1 | 0.76149608 | 1 |
| 226942_at | PHF20L1 | PHD finger protein 20-like 1 | 0.007223841 | 1 | 0.76149608 | 0.836108901 |
| 200733_s_at | PTP4A1 | protein tyrosine phosphatase type IVA, member 1 | 0.007223841 | 1 | 0.671367293 | 1 |
| 213362_at | PTPRD | protein tyrosine phosphatase, receptor type, D | 0.007249105 | 1 | 0.76149608 | 1 |
| 203779_s_at | MPZL2 | myelin protein zero-like 2 | 0.007283894 | 1 | 0.76149608 | 1 |
| 239572_at | GJA3 | gap junction protein, alpha 3, 46kDa | 0.007309493 | 1 | 0.76149608 | 0.960517727 |
| 239370_at | NA | NA | 0.007313028 | 1 | 0.76149608 | 1 |
| 205113_at | NEFM | neurofilament, medium polypeptide 150kDa | 0.007318583 | 0.906618065 | 0.76149608 | 1 |
| 1555383_a_at | POF1B | premature ovarian failure, 1B | 0.007318987 | 1 | 0.76149608 | 1 |
| 213780_at | TCHH | trichohyalin | 0.007348793 | 1 | 0.76149608 | 1 |
| 200784_s_at | LRP1 | low density lipoprotein-related protein 1 (alpha-2-macroglobulin receptor) | 0.007359404 | 1 | 0.100271735 | 1 |
| 203064_s_at | FOXK2 | forkhead box K2 | 0.007372318 | 1 | 0.760085174 | 0.907966981 |
| 230195_at | NA | NA | 0.007406193 | 0.858452395 | 0.76149608 | 0.757924823 |
| 206595_at | CST6 | cystatin E/M | 0.007406193 | 1 | 0.331255637 | 1 |
| 225846_at | RBM35A | RNA binding motif protein 35A | 0.007450408 | 1 | 0.310297427 | 1 |
| 201231_s_at | ENO1 | enolase 1, (alpha) | 0.007529681 | 1 | 0.252094219 | 1 |
| 218013_x_at | DCTN4 | dynactin 4 (p62) | 0.007535059 | 1 | 0.76149608 | 1 |
| 203746_s_at | HCCS | holocytochrome c synthase (cytochrome c heme-lyase) | 0.007539625 | 1 | 0.76149608 | 0.99321009 |
| 218884_s_at | GUF1 | GUF1 GTPase homolog (S. cerevisiae) | 0.007539625 | 1 | 0.76149608 | 0.757924823 |
| 202421_at | IGSF3 | immunoglobulin superfamily, member 3 | 0.007539625 | 1 | 0.76149608 | 1 |
| 223452_s_at | DKFZP564J0863 | DKFZP564J0863 protein | 0.007539625 | 1 | 0.206197669 | 1 |
| 218922_s_at | LASS4 | LAG1 homolog, ceramide synthase 4 | 0.007539625 | 1 | 0.76149608 | 0.798302594 |
| 1552978_a_at | SCAMP1 | secretory carrier membrane protein 1 | 0.007550809 | 1 | 0.832961086 | 1 |
| 220724_at | FLJ21511 | hypothetical protein FLJ21511 | 0.007552027 | 1 | 0.76149608 | 1 |
| 221667_s_at | HSPB8 | heat shock 22kDa protein 8 | 0.007563766 | 1 | 0.76149608 | 1 |
| 214701_s_at | FN1 | fibronectin 1 | 0.007564812 | 0.982770731 | 0.348892225 | 1 |
| 205172_x_at | CLTB | clathrin, light chain (Lcb) | 0.00756853 | 1 | 0.76149608 | 1 |
| 210791_s_at | RICS | Rho GTPase-activating protein | 0.007575652 | 1 | 0.76149608 | 0.940258372 |
| 204614_at | SERPINB2 | serpin peptidase inhibitor, clade B (ovalbumin), member 2 | 0.007583457 | 1 | 0.76149608 | 1 |
| 202855_s_at | SLC16A3 | solute carrier family 16, member 3 (monocarboxylic acid transporter 4) | 0.00759252 | 1 | 0.353844404 | 1 |
| 212970_at | NA | NA | 0.007604909 | 1 | 0.76149608 | 0.757924823 |
| 229432_at | NAGS | N-acetylglutamate synthase | 0.007615764 | 0.852700273 | 0.76149608 | 0.969637474 |
| 209156_s_at | COL6A2 | collagen, type VI, alpha 2 | 0.00761948 | 1 | 0.000174036 | 1 |
| 202489_s_at | FXYD3 | FXYD domain containing ion transport regulator 3 | 0.007656519 | 1 | 0.30166686 | 1 |
| 225293_at | COL27A1 | collagen, type XXVII, alpha 1 | 0.00765844 | 1 | 0.76149608 | 1 |
| 219211_at | USP18 | ubiquitin specific peptidase 18 | 0.007661377 | 1 | 0.76149608 | 1 |
| 1555427_s_at | SYNCRIP | synaptotagmin binding, cytoplasmic RNA interacting protein | 0.007661377 | 1 | 0.76149608 | 1 |
| 219850_s_at | EHF | ets homologous factor | 0.007696322 | 1 | 0.76149608 | 1 |
| 216396_s_at | EI24 | etoposide induced 2.4 mRNA | 0.007705995 | 1 | 0.213454447 | 1 |
| 210873_x_at | APOBEC3A | apolipoprotein B mRNA editing enzyme, catalytic polypeptide-like 3A | 0.00771159 | 1 | 0.76149608 | 1 |
| 219362_at | MAK10 | MAK10 homolog, amino-acid N-acetyltransferase subunit, (S. cerevisiae) | 0.007753081 | 1 | 0.76149608 | 0.846710202 |
| 206177_s_at | ARG1 | arginase, liver | 0.007768839 | 1 | 0.76149608 | 1 |
| 226246_at | KCTD1 | potassium channel tetramerisation domain containing 1 | 0.007811505 | 1 | 0.76149608 | 1 |
| 205513_at | TCN1 | transcobalamin I (vitamin B12 binding protein, R binder family) | 0.007831101 | 1 | 0.76149608 | 1 |
| 239273_s_at | MMP28 | matrix metallopeptidase 28 | 0.007850881 | 1 | 0.76149608 | 0.926274767 |
| 1553589_a_at | PDZK1IP1 | PDZK1 interacting protein 1 | 0.007936544 | 1 | 0.76149608 | 1 |
| 219553_at | NME7 | non-metastatic cells 7, protein expressed in (nucleoside-diphosphate kinase) | 0.007986171 | 0.835232341 | 0.76149608 | 1 |
| 221853_s_at | NOMO3 | NODAL modulator 3 | 0.007995156 | 0.895958406 | 0.76149608 | 0.757924823 |
| 223506_at | ZC3H8 | zinc finger CCCH-type containing 8 | 0.008003527 | 0.835232341 | 0.764734882 | 0.757924823 |
| 203889_at | SCG5 | secretogranin V (7B2 protein) | 0.008010335 | 0.984911827 | 0.288175949 | 1 |
| 202085_at | TJP2 | tight junction protein 2 (zona occludens 2) | 0.008035944 | 1 | 0.76149608 | 1 |
| 220239_at | KLHL7 | kelch-like 7 (Drosophila) | 0.008035944 | 1 | 0.045569402 | 1 |
| 203222_s_at | TLE1 | transducin-like enhancer of split 1 (E(sp1) homolog, Drosophila) | 0.008046312 | 1 | 0.888496126 | 0.757924823 |
| 213992_at | COL4A6 | collagen, type IV, alpha 6 | 0.00805221 | 1 | 0.044560186 | 0.757924823 |
| 244533_at | NA | NA | 0.008061661 | 1 | 0.279788272 | 1 |
| 1553602_at | MUCL1 | mucin-like 1 | 0.008069151 | 1 | 0.76149608 | 1 |
| 219234_x_at | SCRN3 | secernin 3 | 0.008091235 | 0.835232341 | 0.76149608 | 0.847925717 |
| 225113_at | AGPS | alkylglycerone phosphate synthase | 0.008091235 | 1 | 0.76149608 | 1 |
| 222155_s_at | GPR172A | G protein-coupled receptor 172A | 0.008146202 | 1 | 0.055479569 | 1 |
| 229865_at | FNDC3B | fibronectin type III domain containing 3B | 0.008150098 | 1 | 0.055445166 | 0.757924823 |
| 207191_s_at | ISLR | immunoglobulin superfamily containing leucine-rich repeat | 0.008172325 | 0.835232341 | 0.150061598 | 0.9958919 |
| 225655_at | UHRF1 | ubiquitin-like, containing PHD and RING finger domains, 1 | 0.008189678 | 1 | 0.180809005 | 1 |
| 218433_at | PANK3 | pantothenate kinase 3 | 0.008233861 | 1 | 0.76149608 | 1 |
| 1552301_a_at | CORO6 | coronin 6 | 0.008253738 | 1 | 0.76149608 | 0.990027275 |
| 205896_at | SLC22A4 | solute carrier family 22 (organic cation transporter), member 4 | 0.0082917 | 1 | 0.76149608 | 1 |
| 206157_at | PTX3 | pentraxin-related gene, rapidly induced by IL-1 beta | 0.008296419 | 0.835232341 | 0.028309119 | 1 |
| 219263_at | RNF128 | ring finger protein 128 | 0.008296419 | 1 | 0.341573215 | 1 |
| 221870_at | EHD2 | EH-domain containing 2 | 0.008339325 | 1 | 0.76149608 | 0.947874481 |
| 214560_at | FPRL2 | formyl peptide receptor-like 2 | 0.008339325 | 1 | 0.76149608 | 1 |
| 201850_at | CAPG | capping protein (actin filament), gelsolin-like | 0.008384762 | 1 | 0.091408771 | 1 |
| 202686_s_at | AXL | AXL receptor tyrosine kinase | 0.008393728 | 0.900673885 | 0.141681673 | 1 |
| 37152_at | PPARD | peroxisome proliferator-activated receptor delta | 0.008448248 | 1 | 0.76149608 | 1 |
| 218963_s_at | KRT23 | keratin 23 (histone deacetylase inducible) | 0.008448248 | 1 | 0.76149608 | 1 |
| 220475_at | SLC28A3 | solute carrier family 28 (sodium-coupled nucleoside transporter), member 3 | 0.008487585 | 1 | 0.76149608 | 0.978780674 |
| 241994_at | XDH | xanthine dehydrogenase | 0.008504605 | 1 | 0.337751916 | 1 |
| 230323_s_at | TMEM45B | transmembrane protein 45B | 0.008584393 | 1 | 0.76149608 | 1 |
| 203878_s_at | MMP11 | matrix metallopeptidase 11 (stromelysin 3) | 0.008604217 | 1 | 0.098997683 | 0.882674605 |
| 218728_s_at | CNIH4 | cornichon homolog 4 (Drosophila) | 0.008616141 | 1 | 0.388471307 | 1 |
| 203931_s_at | MRPL12 | mitochondrial ribosomal protein L12 | 0.008663203 | 1 | 0.443439493 | 1 |
| 224913_s_at | TIMM50 | translocase of inner mitochondrial membrane 50 homolog (S. cerevisiae) | 0.008704954 | 1 | 0.76149608 | 1 |
| 222393_s_at | NAT13 | N-acetyltransferase 13 | 0.008704954 | 1 | 0.76149608 | 1 |
| 200785_s_at | LRP1 | low density lipoprotein-related protein 1 (alpha-2-macroglobulin receptor) | 0.008733403 | 1 | 0.003003956 | 1 |
| 201130_s_at | CDH1 | cadherin 1, type 1, E-cadherin (epithelial) | 0.008753616 | 1 | 0.76149608 | 1 |
| 207324_s_at | DSC1 | desmocollin 1 | 0.008760403 | 1 | 0.76149608 | 1 |
| 209129_at | TRIP6 | thyroid hormone receptor interactor 6 | 0.008790284 | 1 | 0.76149608 | 1 |
| 212850_s_at | LRP4 | low density lipoprotein receptor-related protein 4 | 0.008791605 | 1 | 0.76149608 | 1 |
| 227718_at | PURB | purine-rich element binding protein B | 0.008810619 | 1 | 0.396850385 | 1 |
| 1557321_a_at | CAPN14 | calpain 14 | 0.008830226 | 1 | 0.853051784 | 0.757924823 |
| 239286_at | NA | NA | 0.0088703 | 0.835232341 | 0.76149608 | 0.801906544 |
| 206407_s_at | CCL13 | chemokine (C-C motif) ligand 13 | 0.008895439 | 1 | 0.011108721 | 1 |
| 1552477_a_at | IRF6 | interferon regulatory factor 6 | 0.00890899 | 1 | 0.222240255 | 1 |
| 225147_at | PSCD3 | pleckstrin homology, Sec7 and coiled-coil domains 3 | 0.008941926 | 1 | 0.393818075 | 0.997123464 |
| 221666_s_at | PYCARD | PYD and CARD domain containing | 0.008975867 | 1 | 0.76149608 | 1 |
| 223738_s_at | PGM2 | phosphoglucomutase 2 | 0.008982984 | 1 | 0.76149608 | 1 |
| 206026_s_at | TNFAIP6 | tumor necrosis factor, alpha-induced protein 6 | 0.008982984 | 1 | 0.020181909 | 1 |
| 202932_at | YES1 | v-yes-1 Yamaguchi sarcoma viral oncogene homolog 1 | 0.008998222 | 1 | 0.60589169 | 1 |
| 231033_at | NA | NA | 0.009014859 | 1 | 0.76149608 | 1 |
| 213518_at | PRKCI | protein kinase C, iota | 0.009043002 | 1 | 0.76149608 | 1 |
| 236534_at | BNIPL | BCL2/adenovirus E1B 19kD interacting protein like | 0.009061035 | 1 | 0.76149608 | 1 |
| 1569144_a_at | MGC59937 | Similar to RIKEN cDNA 2310002J15 gene | 0.009061035 | 1 | 0.76149608 | 1 |
| 242665_at | FMNL2 | formin-like 2 | 0.009064256 | 1 | 0.76149608 | 1 |
| 1555905_a_at | C3orf23 | chromosome 3 open reading frame 23 | 0.009068102 | 1 | 0.79573068 | 1 |
| 238462_at | STS-1 | Cbl-interacting protein Sts-1 | 0.009137762 | 0.835232341 | 0.76149608 | 0.937577677 |
| 200764_s_at | CTNNA1 | catenin (cadherin-associated protein), alpha 1, 102kDa | 0.009150277 | 1 | 0.76149608 | 1 |
| 213599_at | OIP5 | Opa interacting protein 5 | 0.009154073 | 1 | 0.76149608 | 1 |
| 228323_at | CASC5 | cancer susceptibility candidate 5 | 0.009163687 | 1 | 0.76149608 | 1 |
| 206007_at | PRG4 | proteoglycan 4 | 0.009180707 | 0.835232341 | 0.76149608 | 0.757924823 |
| 219863_at | HERC5 | hect domain and RLD 5 | 0.009180707 | 1 | 0.76149608 | 0.977858424 |
| 211715_s_at | BDH1 | 3-hydroxybutyrate dehydrogenase, type 1 | 0.009199537 | 1 | 0.76149608 | 0.960388551 |
| 232860_x_at | RBM41 | RNA binding motif protein 41 | 0.00921932 | 1 | 0.76149608 | 0.788301287 |
| 226803_at | CHMP4C | chromatin modifying protein 4C | 0.009229093 | 1 | 0.76149608 | 1 |
| 231733_at | ICEBERG | ICEBERG caspase-1 inhibitor | 0.009231311 | 1 | 0.76149608 | 1 |
| 208992_s_at | STAT3 | signal transducer and activator of transcription 3 (acute-phase response factor) | 0.009309188 | 1 | 0.76149608 | 1 |
| 228975_at | SP6 | Sp6 transcription factor | 0.009361349 | 1 | 0.76149608 | 0.757924823 |
| 204542_at | ST6GALNAC2 | ST6 (alpha-N-acetyl-neuraminyl-2,3-beta-galactosyl-1,3)-N-acetylgalactosaminide alpha-2,6-sialyltransferase 2 | 0.009382027 | 1 | 0.468682922 | 1 |
| 206662_at | GLRX | glutaredoxin (thioltransferase) | 0.009394531 | 0.882844912 | 0.76149608 | 0.757924823 |
| 213787_s_at | TBC1D25 | TBC1 domain family, member 25 | 0.009413878 | 1 | 0.76149608 | 1 |
| 237262_at | NA | NA | 0.009415961 | 1 | 0.828440304 | 1 |
| 202525_at | PRSS8 | protease, serine, 8 | 0.009542742 | 1 | 0.76149608 | 1 |
| 1554795_a_at | FBLIM1 | filamin binding LIM protein 1 | 0.009546232 | 1 | 0.76149608 | 0.9240141 |
| 1552660_a_at | C5orf22 | chromosome 5 open reading frame 22 | 0.009549759 | 1 | 0.76149608 | 1 |
| 219557_s_at | NRIP3 | nuclear receptor interacting protein 3 | 0.009563525 | 0.835232341 | 0.76149608 | 1 |
| 204656_at | SHB | Src homology 2 domain containing adaptor protein B | 0.009636661 | 1 | 0.76149608 | 1 |
| 238741_at | FAM83A | family with sequence similarity 83, member A | 0.009695699 | 1 | 0.76149608 | 1 |
| 213085_s_at | WWC1 | WW and C2 domain containing 1 | 0.009698013 | 1 | 0.76149608 | 1 |
| 217789_at | SNX6 | sorting nexin 6 | 0.009701026 | 1 | 0.76149608 | 0.878387022 |
| 234985_at | NA | NA | 0.009719694 | 1 | 0.608894252 | 1 |
| 235075_at | DSG3 | desmoglein 3 (pemphigus vulgaris antigen) | 0.009791537 | 1 | 0.39233294 | 1 |
| 219181_at | LIPG | lipase, endothelial | 0.009793535 | 1 | 0.76149608 | 1 |
| 205996_s_at | AK2 | adenylate kinase 2 | 0.009872416 | 0.835232341 | 0.76149608 | 1 |
| 220782_x_at | KLK12 | kallikrein-related peptidase 12 | 0.009872416 | 1 | 0.76149608 | 0.88002135 |
| 213895_at | EMP1 | epithelial membrane protein 1 | 0.009918456 | 1 | 0.76149608 | 1 |
| 233488_at | RNASE7 | ribonuclease, RNase A family, 7 | 0.009926204 | 1 | 0.76149608 | 1 |
| 206239_s_at | SPINK1 | serine peptidase inhibitor, Kazal type 1 | 0.009943197 | 0.835232341 | 0.76149608 | 0.757924823 |
| 205783_at | KLK13 | kallikrein-related peptidase 13 | 0.009983644 | 1 | 0.76149608 | 1 |
| 224595_at | SLC44A1 | solute carrier family 44, member 1 | 0.009991087 | 1 | 0.76149608 | 0.985878358 |
| 224345_x_at | C3orf28 | chromosome 3 open reading frame 28 | 0.009991087 | 1 | 0.76149608 | 0.994442479 |
| 204638_at | ACP5 | acid phosphatase 5, tartrate resistant | 0.011223264 | 1 | 0.000174036 | 1 |
| 201792_at | AEBP1 | AE binding protein 1 | 0.012664894 | 1 | 1.73E-06 | 1 |
| 208002_s_at | ACOT7 | acyl-CoA thioesterase 7 | 0.015703954 | 1 | 0.004288003 | 1 |
| 216971_s_at | PLEC1 | plectin 1, intermediate filament binding protein 500kDa | 0.016591108 | 1 | 6.34E-05 | 1 |
| 214702_at | FN1 | fibronectin 1 | 0.016786191 | 1 | 0.006377006 | 1 |
| 214511_x_at | FCGR1B | Fc fragment of IgG, high affinity Ib, receptor (CD64) | 0.020171089 | 1 | 0.00134785 | 1 |
| 222450_at | TMEPAI | transmembrane, prostate androgen induced RNA | 0.021007262 | 1 | 0.007698621 | 1 |
| 209835_x_at | CD44 | CD44 molecule (Indian blood group) | 0.026954764 | 1 | 0.002593984 | 1 |
| 210387_at | HIST1H2BG | histone cluster 1, H2bg | 0.034470019 | 1 | 8.81E-07 | 0.757924823 |
| 242836_at | ATP1B3 | ATPase, Na+/K+ transporting, beta 3 polypeptide | 0.037224712 | 1 | 2.72E-05 | 0.985872792 |
| 211429_s_at | SERPINA1 | serpin peptidase inhibitor, clade A (alpha-1 antiproteinase, antitrypsin), member 1 | 0.045957654 | 1 | 3.23E-05 | 1 |
| 202237_at | NNMT | nicotinamide N-methyltransferase | 0.052847737 | 1 | 0.001807581 | 1 |
| 235629_at | FN1 | fibronectin 1 | 0.066474086 | 1 | 0.001407728 | 1 |
| 219257_s_at | SPHK1 | sphingosine kinase 1 | 0.072356306 | 1 | 0.001466787 | 1 |
| 203980_at | FABP4 | fatty acid binding protein 4, adipocyte | 0.100008695 | 0.018538719 | 0.946698908 | 0.000379155 |
| 1566901_at | TGIF1 | TGFB-induced factor homeobox 1 | 0.119329744 | 1 | 0.000864298 | 0.988351766 |
| 205666_at | FMO1 | flavin containing monooxygenase 1 | 0.121702149 | 1 | 0.00023332 | 1 |
| 231644_at | NA | NA | 0.12423398 | 1 | 0.001840796 | 1 |
| 205680_at | MMP10 | matrix metallopeptidase 10 (stromelysin 2) | 0.14671656 | 1 | 0.007698621 | 1 |
| 208131_s_at | PTGIS | prostaglandin I2 (prostacyclin) synthase | 0.152343319 | 0.835232341 | 4.96E-05 | 1 |
| 213290_at | COL6A2 | collagen, type VI, alpha 2 | 0.169024113 | 0.985216542 | 0.00886156 | 1 |
| 241473_at | SULF1 | sulfatase 1 | 0.180650089 | 1 | 0.005542578 | 1 |
| 209906_at | C3AR1 | complement component 3a receptor 1 | 0.200836417 | 0.861772026 | 0.008802344 | 1 |
| 201596_x_at | KRT18 | keratin 18 | 0.224204639 | 1 | 0.005149918 | 1 |
| 210220_at | FZD2 | frizzled homolog 2 (Drosophila) | 0.282373138 | 1 | 0.000417282 | 1 |
| 239227_at | EXT1 | exostoses (multiple) 1 | 0.316527356 | 1 | 0.00250143 | 1 |
| 214079_at | DHRS2 | dehydrogenase/reductase (SDR family) member 2 | 0.369494134 | 0.835232341 | 0.008086507 | 0.757924823 |
| 203915_at | CXCL9 | chemokine (C-X-C motif) ligand 9 | 0.377139295 | 0.710851024 | 0.005149918 | 1 |
| 205119_s_at | FPR1 | formyl peptide receptor 1 | 0.399674554 | 1 | 0.008915449 | 1 |
| 236114_at | RUNX1 | runt-related transcription factor 1 (acute myeloid leukemia 1; aml1 oncogene) | 0.460162204 | 0.835232341 | 2.86E-08 | 0.990598024 |
| 227061_at | NA | NA | 0.622943935 | 0.006515148 | 0.025299588 | 1 |
| 242691_at | NA | NA | 0.627822273 | 1 | 1.89E-08 | 0.767574168 |
| 232406_at | JAG1 | jagged 1 (Alagille syndrome) | 0.691719996 | 1 | 0.0045612 | 1 |
| 223499_at | C1QTNF5 | C1q and tumor necrosis factor related protein 5 | 0.704506876 | 1 | 0.006872315 | 0.996225734 |
| 225847_at | AADACL1 | arylacetamide deacetylase-like 1 | 0.796125153 | 4.71E-05 | 0.992198784 | 2.87E-07 |
| 203936_s_at | MMP9 | matrix metallopeptidase 9 (gelatinase B, 92kDa gelatinase, 92kDa type IV collagenase) | 0.796125153 | 0.00024006 | 0.76149608 | 0.306919256 |
| 227724_at | TTC8 | tetratricopeptide repeat domain 8 | 0.796125153 | 0.001437951 | 0.76149608 | 0.757924823 |
| 215221_at | FOXP1 | forkhead box P1 | 0.796125153 | 0.005462695 | 1 | 0.707515813 |
| 217022_s_at | IGHA1 | immunoglobulin heavy constant alpha 1 | 0.796125153 | 0.066390332 | 0.76149608 | 0.000148085 |
| 211645_x_at | NA | NA | 0.796125153 | 0.105195153 | 0.279423787 | 1.06E-07 |
| 209695_at | PTP4A3 | protein tyrosine phosphatase type IVA, member 3 | 0.796125153 | 0.835232341 | 0.646960757 | 2.27E-06 |
| 214777_at | NA | NA | 0.796125153 | 0.835232341 | 0.477483834 | 1.45E-05 |
| 204602_at | DKK1 | dickkopf homolog 1 (Xenopus laevis) | 0.796125153 | 1 | 0.006330728 | 1 |
| 1559020_a_at | NA | NA | 0.796125153 | 1 | 2.13E-06 | 0.757924823 |
| 1565976_at | FCHO2 | FCH domain only 2 | 0.796125153 | 1 | 0.005310514 | 0.808429417 |
| 1557512_at | CLIP1 | CAP-GLY domain containing linker protein 1 | 0.796125153 | 1 | 0.00020597 | 0.757924823 |
| 215198_s_at | CALD1 | caldesmon 1 | 0.796125153 | 1 | 0.003748975 | 0.978140601 |
| 232472_at | FNDC3B | fibronectin type III domain containing 3B | 0.796125153 | 1 | 0.005159389 | 0.757924823 |
| 232174_at | EXT1 | exostoses (multiple) 1 | 0.796125153 | 1 | 0.008534532 | 1 |
| 236203_at | HLA-DQA1 | major histocompatibility complex, class II, DQ alpha 1 | 0.79780399 | 0.000430501 | 0.766923684 | 0.757924823 |
| 206574_s_at | PTP4A3 | protein tyrosine phosphatase type IVA, member 3 | 0.798765105 | 0.956144543 | 0.515274822 | 7.23E-05 |
| 238481_at | MGP | matrix Gla protein | 0.800721644 | 0.029878459 | 1 | 0.002466998 |
| 202062_s_at | SEL1L | sel-1 suppressor of lin-12-like (C. elegans) | 0.811773314 | 0.835232341 | 0.772501579 | 0.006387276 |
| 223299_at | SEC11C | SEC11 homolog C (S. cerevisiae) | 0.811899985 | 0.683673154 | 0.76149608 | 0.000197125 |
| 227259_at | CD47 | CD47 molecule | 0.815662129 | 0.000704459 | 0.998932888 | 0.757924823 |
| 216491_x_at | IGHM | immunoglobulin heavy constant mu | 0.824898207 | 0.407311891 | 0.76149608 | 0.009798604 |
| 240118_at | TXNDC11 | thioredoxin domain containing 11 | 0.854300786 | 0.899687645 | 0.000697106 | 1.26E-08 |
| 210152_at | LILRB4 | leukocyte immunoglobulin-like receptor, subfamily B (with TM and ITIM domains), member 4 | 0.863510573 | 0.59517691 | 0.003015899 | 0.992714816 |
| 204588_s_at | SLC7A7 | solute carrier family 7 (cationic amino acid transporter, y+ system), member 7 | 0.865813174 | 0.076520025 | 0.003807964 | 0.757924823 |
| 233607_at | BICC1 | bicaudal C homolog 1 (Drosophila) | 0.871602778 | 1 | 0.009358569 | 1 |
| 216576_x_at | NTN2L | netrin 2-like (chicken) | 0.897050596 | 0.506459847 | 0.76149608 | 2.43E-05 |
| 231828_at | LOC253039 | hypothetical protein LOC253039 | 0.910582251 | 0.002200204 | 1 | 0.104271563 |
| 243521_at | NA | NA | 0.913938968 | 0.007830898 | 1 | 0.757924823 |
| 211643_x_at | IGKC | immunoglobulin kappa constant | 0.93047624 | 0.835232341 | 0.76149608 | 5.61E-05 |
| 216560_x_at | IGL@ | immunoglobulin lambda locus | 0.936908574 | 0.348297585 | 0.76149608 | 0.000890979 |
| 211644_x_at | IGKC | immunoglobulin kappa constant | 0.942672493 | 0.835232341 | 0.76149608 | 0.001074587 |
| 227808_at | DNAJC15 | DnaJ (Hsp40) homolog, subfamily C, member 15 | 0.961898994 | 0.001335841 | 0.966097147 | 0.757924823 |
| 224342_x_at | LOC96610 | hypothetical gene LOC96610 | 0.979663542 | 0.353643726 | 0.76149608 | 0.000114867 |
| 1556180_at | LOC729678 | hypothetical protein LOC729678 | 0.988909615 | 0.007612235 | 0.91630251 | 0.051274405 |
| 234306_s_at | SLAMF7 | SLAM family member 7 | 0.989773284 | 0.835232341 | 0.76149608 | 0.007079 |
| 217856_at | RBM8A | RNA binding motif protein 8A | 0.991933118 | 0.006977229 | 1 | 0.303293767 |
| 222846_at | RAB8B | RAB8B, member RAS oncogene family | 0.99575629 | 0.000116098 | 1 | 0.757924823 |
| 227396_at | PTPRJ | protein tyrosine phosphatase, receptor type, J | 0.99753966 | 0.008499627 | 0.76149608 | 0.757924823 |
| 228304_at | NA | NA | 0.998610368 | 0.006061064 | 1 | 0.757924823 |
| 219014_at | PLAC8 | placenta-specific 8 | 1 | 8.41E-17 | 1 | 1.51E-08 |
| 225207_at | PDK4 | pyruvate dehydrogenase kinase, isozyme 4 | 1 | 3.01E-13 | 1 | 2.82E-05 |
| 226641_at | ANKRD44 | ankyrin repeat domain 44 | 1 | 4.68E-13 | 1 | 6.50E-05 |
| 236280_at | NA | NA | 1 | 7.52E-13 | 1 | 8.17E-11 |
| 210072_at | CCL19 | chemokine (C-C motif) ligand 19 | 1 | 7.52E-13 | 1 | 3.41E-11 |
| 44673_at | SIGLEC1 | sialic acid binding Ig-like lectin 1, sialoadhesin | 1 | 6.35E-12 | 1 | 1.61E-09 |
| 215933_s_at | HHEX | hematopoietically expressed homeobox | 1 | 8.75E-12 | 1 | 9.60E-10 |
| 221558_s_at | LEF1 | lymphoid enhancer-binding factor 1 | 1 | 8.75E-12 | 1 | 2.91E-06 |
| 232615_at | PDE4DIP | phosphodiesterase 4D interacting protein (myomegalin) | 1 | 9.06E-12 | 1 | 4.49E-05 |
| 227384_s_at | FAM91A2 | family with sequence similarity 91, member A2 | 1 | 9.06E-12 | 1 | 2.55E-05 |
| 228071_at | GIMAP7 | GTPase, IMAP family member 7 | 1 | 9.06E-12 | 1 | 0.058110892 |
| 239946_at | KIAA0922 | KIAA0922 | 1 | 9.06E-12 | 1 | 8.17E-11 |
| 205890_s_at | UBD | ubiquitin D | 1 | 2.88E-11 | 1 | 0.515699356 |
| 215785_s_at | CYFIP2 | cytoplasmic FMR1 interacting protein 2 | 1 | 4.15E-11 | 1 | 3.29E-07 |
| 211796_s_at | TRBC1 | T cell receptor beta constant 1 | 1 | 8.88E-11 | 1 | 0.018421958 |
| 227346_at | IKZF1 | IKAROS family zinc finger 1 (Ikaros) | 1 | 1.08E-10 | 1 | 1.32E-06 |
| 209685_s_at | PRKCB1 | protein kinase C, beta 1 | 1 | 1.38E-10 | 1 | 8.17E-11 |
| 229513_at | STRBP | spermatid perinuclear RNA binding protein | 1 | 3.29E-10 | 1 | 1.73E-08 |
| 202746_at | ITM2A | integral membrane protein 2A | 1 | 5.49E-10 | 1 | 0.167523874 |
| 241681_at | MBNL1 | muscleblind-like (Drosophila) | 1 | 5.90E-10 | 1 | 0.001348494 |
| 1557240_a_at | BBX | bobby sox homolog (Drosophila) | 1 | 7.73E-10 | 1 | 0.004883259 |
| 209687_at | CXCL12 | chemokine (C-X-C motif) ligand 12 (stromal cell-derived factor 1) | 1 | 7.73E-10 | 1 | 0.757924823 |
| 229686_at | P2RY8 | purinergic receptor P2Y, G-protein coupled, 8 | 1 | 1.48E-09 | 1 | 1.41E-08 |
| 219519_s_at | SIGLEC1 | sialic acid binding Ig-like lectin 1, sialoadhesin | 1 | 1.95E-09 | 0.934116887 | 1.41E-08 |
| 226365_at | LOC728555 | hypothetical protein LOC728555 | 1 | 3.23E-09 | 1 | 0.333738195 |
| 210915_x_at | TRBC1 | T cell receptor beta constant 1 | 1 | 3.30E-09 | 1 | 0.000753879 |
| 206693_at | IL7 | interleukin 7 | 1 | 3.30E-09 | 1 | 0.015509727 |
| 202920_at | ANK2 | ankyrin 2, neuronal | 1 | 3.30E-09 | 1 | 1 |
| 228167_at | KLHL6 | kelch-like 6 (Drosophila) | 1 | 5.03E-09 | 1 | 7.90E-09 |
| 207238_s_at | PTPRC | protein tyrosine phosphatase, receptor type, C | 1 | 5.47E-09 | 1 | 0.064502009 |
| 210172_at | SF1 | splicing factor 1 | 1 | 5.73E-09 | 1 | 7.61E-07 |
| 34210_at | CD52 | CD52 molecule | 1 | 7.53E-09 | 1 | 6.09E-08 |
| 204220_at | GMFG | glia maturation factor, gamma | 1 | 8.37E-09 | 1 | 0.098917617 |
| 1556657_at | MBNL1 | muscleblind-like (Drosophila) | 1 | 8.51E-09 | 0.266283731 | 6.74E-06 |
| 228455_at | RBM15 | RNA binding motif protein 15 | 1 | 8.51E-09 | 1 | 0.000225779 |
| 226435_at | PAPLN | papilin, proteoglycan-like sulfated glycoprotein | 1 | 9.18E-09 | 1 | 8.17E-11 |
| 243366_s_at | CERKL | ceramide kinase-like | 1 | 1.24E-08 | 1 | 1.26E-08 |
| 206337_at | CCR7 | chemokine (C-C motif) receptor 7 | 1 | 1.52E-08 | 1 | 0.000191784 |
| 209200_at | MEF2C | myocyte enhancer factor 2C | 1 | 1.90E-08 | 1 | 3.92E-06 |
| 208792_s_at | CLU | clusterin | 1 | 2.00E-08 | 1 | 0.333738195 |
| 228055_at | NAPSB | napsin B aspartic peptidase pseudogene | 1 | 2.53E-08 | 1 | 1.32E-07 |
| 212942_s_at | KIAA1199 | KIAA1199 | 1 | 2.53E-08 | 1 | 3.28E-05 |
| 204563_at | SELL | selectin L (lymphocyte adhesion molecule 1) | 1 | 2.55E-08 | 1 | 0.001310221 |
| 235385_at | NA | NA | 1 | 2.55E-08 | 0.991680402 | 0.122326997 |
| 213326_at | VAMP1 | vesicle-associated membrane protein 1 (synaptobrevin 1) | 1 | 3.54E-08 | 1 | 3.53E-07 |
| 204891_s_at | LCK | lymphocyte-specific protein tyrosine kinase | 1 | 3.78E-08 | 1 | 0.004943457 |
| 204719_at | ABCA8 | ATP-binding cassette, sub-family A (ABC1), member 8 | 1 | 4.17E-08 | 1 | 0.757924823 |
| 38149_at | ARHGAP25 | Rho GTPase activating protein 25 | 1 | 5.06E-08 | 1 | 0.000110155 |
| 222731_at | ZDHHC2 | zinc finger, DHHC-type containing 2 | 1 | 5.46E-08 | 1 | 0.148340562 |
| 207277_at | CD209 | CD209 molecule | 1 | 6.60E-08 | 1 | 5.87E-06 |
| 242268_at | CUGBP2 | CUG triplet repeat, RNA binding protein 2 | 1 | 1.05E-07 | 1 | 7.11E-05 |
| 239597_at | PAN3 | PAN3 polyA specific ribonuclease subunit homolog (S. cerevisiae) | 1 | 1.05E-07 | 1 | 0.000465726 |
| 204352_at | TRAF5 | TNF receptor-associated factor 5 | 1 | 1.19E-07 | 1 | 0.002337113 |
| 212414_s_at | SEPT6 | septin 6 | 1 | 1.19E-07 | 1 | 0.012216206 |
| 1555247_a_at | RAPGEF6 | Rap guanine nucleotide exchange factor (GEF) 6 | 1 | 1.23E-07 | 1 | 0.082291032 |
| 203685_at | BCL2 | B-cell CLL/lymphoma 2 | 1 | 1.39E-07 | 1 | 4.01E-07 |
| 203471_s_at | PLEK | pleckstrin | 1 | 1.39E-07 | 0.979162603 | 0.001950322 |
| 235051_at | CCDC50 | coiled-coil domain containing 50 | 1 | 1.76E-07 | 1 | 0.000225779 |
| 228592_at | MS4A1 | membrane-spanning 4-domains, subfamily A, member 1 | 1 | 1.94E-07 | 1 | 9.60E-05 |
| 240665_at | CUGBP2 | CUG triplet repeat, RNA binding protein 2 | 1 | 2.06E-07 | 1 | 0.000197125 |
| 1559078_at | BCL11A | B-cell CLL/lymphoma 11A (zinc finger protein) | 1 | 2.30E-07 | 1 | 8.52E-05 |
| 1559025_at | SEPT9 | septin 9 | 1 | 2.43E-07 | 0.886843414 | 0.002063752 |
| 211919_s_at | CXCR4 | chemokine (C-X-C motif) receptor 4 | 1 | 2.55E-07 | 1 | 0.641076252 |
| 229800_at | DCLK1 | doublecortin-like kinase 1 | 1 | 2.92E-07 | 1 | 0.963313169 |
| 233302_at | BCL11B | B-cell CLL/lymphoma 11B (zinc finger protein) | 1 | 3.07E-07 | 1 | 0.020724052 |
| 213998_s_at | DDX17 | DEAD (Asp-Glu-Ala-Asp) box polypeptide 17 | 1 | 3.59E-07 | 1 | 0.002975215 |
| 240572_s_at | LOC374443 | CLR pseudogene | 1 | 3.59E-07 | 1 | 0.010281206 |
| 202747_s_at | ITM2A | integral membrane protein 2A | 1 | 3.59E-07 | 1 | 0.141684356 |
| 238536_at | LOC642236 | similar to FRG1 protein (FSHD region gene 1 protein) | 1 | 3.73E-07 | 1 | 0.006387276 |
| 208791_at | CLU | clusterin | 1 | 3.77E-07 | 1 | 5.31E-05 |
| 219574_at | MARCH1 | membrane-associated ring finger (C3HC4) 1 | 1 | 3.81E-07 | 1 | 0.757924823 |
| 219279_at | DOCK10 | dedicator of cytokinesis 10 | 1 | 3.93E-07 | 1 | 0.00440915 |
| 206545_at | CD28 | CD28 molecule | 1 | 4.55E-07 | 1 | 7.89E-06 |
| 219471_at | C13orf18 | chromosome 13 open reading frame 18 | 1 | 5.65E-07 | 1 | 0.233026471 |
| 1556543_at | ZCCHC7 | zinc finger, CCHC domain containing 7 | 1 | 5.84E-07 | 1 | 5.20E-05 |
| 227266_s_at | FYB | FYN binding protein (FYB-120/130) | 1 | 6.03E-07 | 1 | 0.005628695 |
| 218918_at | MAN1C1 | mannosidase, alpha, class 1C, member 1 | 1 | 7.35E-07 | 1 | 0.757924823 |
| 235879_at | MBNL1 | muscleblind-like (Drosophila) | 1 | 7.35E-07 | 1 | 0.106525089 |
| 241891_at | DOCK8 | dedicator of cytokinesis 8 | 1 | 7.50E-07 | 1 | 1.93E-05 |
| 223058_at | FAM107B | family with sequence similarity 107, member B | 1 | 8.35E-07 | 1 | 0.106392173 |
| 203666_at | CXCL12 | chemokine (C-X-C motif) ligand 12 (stromal cell-derived factor 1) | 1 | 8.35E-07 | 1 | 0.024786859 |
| 212672_at | ATM | ataxia telangiectasia mutated | 1 | 9.15E-07 | 1 | 0.001647831 |
| 210538_s_at | BIRC3 | baculoviral IAP repeat-containing 3 | 1 | 9.25E-07 | 1 | 0.001078571 |
| 226525_at | STK17B | serine/threonine kinase 17b | 1 | 1.03E-06 | 1 | 0.114152632 |
| 232527_at | NA | NA | 1 | 1.10E-06 | 1 | 0.166959686 |
| 203868_s_at | VCAM1 | vascular cell adhesion molecule 1 | 1 | 1.27E-06 | 1 | 0.330962078 |
| 205242_at | CXCL13 | chemokine (C-X-C motif) ligand 13 (B-cell chemoattractant) | 1 | 1.28E-06 | 1 | 1 |
| 236000_s_at | HNRPD | heterogeneous nuclear ribonucleoprotein D (AU-rich element RNA binding protein 1, 37kDa) | 1 | 1.36E-06 | 1 | 1.99E-05 |
| 232843_s_at | DOCK8 | dedicator of cytokinesis 8 | 1 | 2.14E-06 | 1 | 0.003740808 |
| 1554240_a_at | ITGAL | integrin, alpha L (antigen CD11A (p180), lymphocyte function-associated antigen 1; alpha polypeptide) | 1 | 2.85E-06 | 0.987968935 | 0.000655105 |
| 235046_at | NA | NA | 1 | 2.91E-06 | 1 | 0.757924823 |
| 214093_s_at | FUBP1 | far upstream element (FUSE) binding protein 1 | 1 | 3.89E-06 | 1 | 0.071841291 |
| 203332_s_at | INPP5D | inositol polyphosphate-5-phosphatase, 145kDa | 1 | 4.05E-06 | 1 | 0.001768082 |
| 209795_at | CD69 | CD69 molecule | 1 | 4.05E-06 | 1 | 0.371177424 |
| 210664_s_at | TFPI | tissue factor pathway inhibitor (lipoprotein-associated coagulation inhibitor) | 1 | 4.05E-06 | 1 | 0.175607597 |
| 230180_at | DDX17 | DEAD (Asp-Glu-Ala-Asp) box polypeptide 17 | 1 | 4.05E-06 | 1 | 0.010507861 |
| 237009_at | CD69 | CD69 molecule | 1 | 4.05E-06 | 1 | 0.000604601 |
| 207651_at | GPR171 | G protein-coupled receptor 171 | 1 | 4.05E-06 | 1 | 0.445117393 |
| 227261_at | KLF12 | Kruppel-like factor 12 | 1 | 4.27E-06 | 1 | 5.93E-05 |
| 244313_at | CR1 | complement component (3b/4b) receptor 1 (Knops blood group) | 1 | 4.32E-06 | 1 | 0.073171752 |
| 209201_x_at | CXCR4 | chemokine (C-X-C motif) receptor 4 | 1 | 4.37E-06 | 1 | 0.247453715 |
| 212873_at | HMHA1 | histocompatibility (minor) HA-1 | 1 | 4.59E-06 | 1 | 0.104856938 |
| 232315_at | LOC400713 | zinc finger-like | 1 | 4.59E-06 | 0.76149608 | 0.757924823 |
| 235213_at | ITPKB | inositol 1,4,5-trisphosphate 3-kinase B | 1 | 4.59E-06 | 1 | 0.001156954 |
| 225191_at | CIRBP | cold inducible RNA binding protein | 1 | 4.77E-06 | 1 | 1.04E-05 |
| 213416_at | CERKL | ceramide kinase-like | 1 | 5.38E-06 | 1 | 0.034389955 |
| 238875_at | PLGLB1 | plasminogen-like B1 | 1 | 6.06E-06 | 1 | 0.000388962 |
| 1558111_at | MBNL1 | muscleblind-like (Drosophila) | 1 | 6.55E-06 | 1 | 0.049317831 |
| 210116_at | SH2D1A | SH2 domain protein 1A, Duncan's disease (lymphoproliferative syndrome) | 1 | 7.68E-06 | 1 | 0.000114808 |
| 243546_at | SESN3 | sestrin 3 | 1 | 7.91E-06 | 1 | 0.000388962 |
| 204793_at | GPRASP1 | G protein-coupled receptor associated sorting protein 1 | 1 | 1.00E-05 | 1 | 0.000208815 |
| 242405_at | MAML2 | mastermind-like 2 (Drosophila) | 1 | 1.02E-05 | 1 | 0.14945904 |
| 205739_x_at | ZNF107 | zinc finger protein 107 | 1 | 1.08E-05 | 0.932118087 | 0.448662405 |
| 204613_at | PLCG2 | phospholipase C, gamma 2 (phosphatidylinositol-specific) | 1 | 1.15E-05 | 1 | 4.10E-05 |
| 230653_at | LOC728555 | hypothetical protein LOC728555 | 1 | 1.19E-05 | 1 | 0.757924823 |
| 227383_at | FAM91A2 | family with sequence similarity 91, member A2 | 1 | 1.19E-05 | 1 | 0.015398775 |
| 241956_at | PCGF5 | polycomb group ring finger 5 | 1 | 1.21E-05 | 1 | 0.014408294 |
| 1559156_at | PIAS1 | protein inhibitor of activated STAT, 1 | 1 | 1.24E-05 | 1 | 1.03E-06 |
| 215269_at | TMEM1 | transmembrane protein 1 | 1 | 1.24E-05 | 1 | 0.003215747 |
| 226444_at | SLC39A10 | solute carrier family 39 (zinc transporter), member 10 | 1 | 1.40E-05 | 1 | 0.002479757 |
| 208914_at | GGA2 | golgi associated, gamma adaptin ear containing, ARF binding protein 2 | 1 | 1.47E-05 | 1 | 0.000692718 |
| 1557239_at | BBX | bobby sox homolog (Drosophila) | 1 | 1.55E-05 | 1 | 0.113609164 |
| 213193_x_at | TRBC1 | T cell receptor beta constant 1 | 1 | 1.81E-05 | 0.993565012 | 0.061891097 |
| 207339_s_at | LTB | lymphotoxin beta (TNF superfamily, member 3) | 1 | 1.82E-05 | 1 | 7.58E-06 |
| 219243_at | GIMAP4 | GTPase, IMAP family member 4 | 1 | 1.92E-05 | 1 | 0.751708629 |
| 226981_at | MLL | myeloid/lymphoid or mixed-lineage leukemia (trithorax homolog, Drosophila) | 1 | 1.97E-05 | 1 | 0.05481923 |
| 209068_at | HNRPDL | heterogeneous nuclear ribonucleoprotein D-like | 1 | 1.99E-05 | 1 | 0.163750747 |
| 204057_at | IRF8 | interferon regulatory factor 8 | 1 | 2.18E-05 | 1 | 0.051074249 |
| 208998_at | UCP2 | uncoupling protein 2 (mitochondrial, proton carrier) | 1 | 2.43E-05 | 0.789612795 | 0.006159903 |
| 227719_at | NA | NA | 1 | 2.44E-05 | 1 | 0.460220584 |
| 213375_s_at | CG018 | hypothetical gene CG018 | 1 | 2.56E-05 | 1 | 0.20705485 |
| 219777_at | GIMAP6 | GTPase, IMAP family member 6 | 1 | 2.56E-05 | 1 | 0.557598322 |
| 204950_at | CARD8 | caspase recruitment domain family, member 8 | 1 | 2.71E-05 | 1 | 0.068701902 |
| 204863_s_at | IL6ST | interleukin 6 signal transducer (gp130, oncostatin M receptor) | 1 | 2.72E-05 | 1 | 5.61E-05 |
| 241435_at | ETS1 | v-ets erythroblastosis virus E26 oncogene homolog 1 (avian) | 1 | 2.72E-05 | 1 | 0.130830989 |
| 242827_x_at | TNFAIP8 | tumor necrosis factor, alpha-induced protein 8 | 1 | 2.73E-05 | 1 | 0.000144138 |
| 244592_at | FLI1 | Friend leukemia virus integration 1 | 1 | 2.78E-05 | 1 | 0.001770908 |
| 1559584_a_at | C16orf54 | chromosome 16 open reading frame 54 | 1 | 2.81E-05 | 1 | 0.00340697 |
| 204192_at | CD37 | CD37 molecule | 1 | 2.81E-05 | 1 | 0.002821996 |
| 201998_at | ST6GAL1 | ST6 beta-galactosamide alpha-2,6-sialyltranferase 1 | 1 | 2.94E-05 | 0.973932516 | 0.000119785 |
| 203381_s_at | APOE | apolipoprotein E | 1 | 2.94E-05 | 0.76149608 | 0.757924823 |
| 212587_s_at | PTPRC | protein tyrosine phosphatase, receptor type, C | 1 | 3.44E-05 | 0.965279246 | 0.520365351 |
| 39582_at | CYLD | cylindromatosis (turban tumor syndrome) | 1 | 3.44E-05 | 1 | 0.138683209 |
| 240865_at | NA | NA | 1 | 3.56E-05 | 1 | 0.174064006 |
| 236295_s_at | NLRC3 | NLR family, CARD domain containing 3 | 1 | 3.86E-05 | 1 | 0.029414287 |
| 223553_s_at | DOK3 | docking protein 3 | 1 | 3.91E-05 | 0.963476796 | 1.78E-05 |
| 230389_at | FNBP1 | formin binding protein 1 | 1 | 3.91E-05 | 1 | 0.000497704 |
| 1552316_a_at | GIMAP1 | GTPase, IMAP family member 1 | 1 | 3.98E-05 | 1 | 0.241545103 |
| 235023_at | VPS13C | vacuolar protein sorting 13 homolog C (S. cerevisiae) | 1 | 4.28E-05 | 1 | 0.017321254 |
| 205110_s_at | FGF13 | fibroblast growth factor 13 | 1 | 4.30E-05 | 1 | 0.078366169 |
| 206687_s_at | PTPN6 | protein tyrosine phosphatase, non-receptor type 6 | 1 | 4.34E-05 | 0.990507052 | 0.173338474 |
| 225786_at | LOC284702 | hypothetical protein LOC284702 | 1 | 4.38E-05 | 1 | 0.180164496 |
| 211726_s_at | FMO2 | flavin containing monooxygenase 2 (non-functional) | 1 | 4.52E-05 | 1 | 0.757924823 |
| 210972_x_at | TRA@ | T cell receptor alpha locus | 1 | 4.60E-05 | 1 | 0.022473131 |
| 229193_at | CROP | cisplatin resistance-associated overexpressed protein | 1 | 4.71E-05 | 1 | 0.03712392 |
| 203185_at | RASSF2 | Ras association (RalGDS/AF-6) domain family 2 | 1 | 4.71E-05 | 1 | 0.41111181 |
| 228771_at | ADRBK2 | adrenergic, beta, receptor kinase 2 | 1 | 4.88E-05 | 1 | 0.00083941 |
| 232024_at | GIMAP2 | GTPase, IMAP family member 2 | 1 | 5.01E-05 | 0.982840656 | 0.58435426 |
| 228869_at | SLIC1 | selectin ligand interactor cytoplasmic-1 | 1 | 5.47E-05 | 0.811023267 | 0.000449065 |
| 36545_s_at | SFI1 | Sfi1 homolog, spindle assembly associated (yeast) | 1 | 5.80E-05 | 1 | 0.082767538 |
| 236685_at | NA | NA | 1 | 5.96E-05 | 1 | 0.601504092 |
| 202663_at | WIPF1 | WAS/WASL interacting protein family, member 1 | 1 | 6.08E-05 | 1 | 0.757924823 |
| 238447_at | RBMS3 | RNA binding motif, single stranded interacting protein | 1 | 6.54E-05 | 1 | 0.531796269 |
| AFFX-HUMRGE/M10098_5_at | NA | NA | 1 | 6.55E-05 | 1 | 0.757924823 |
| 239231_at | NA | NA | 1 | 7.14E-05 | 0.94135776 | 0.072040737 |
| 232125_at | NA | NA | 1 | 7.52E-05 | 1 | 0.757924823 |
| 204236_at | FLI1 | Friend leukemia virus integration 1 | 1 | 7.65E-05 | 1 | 0.691959903 |
| 1555892_s_at | LOC253039 | hypothetical protein LOC253039 | 1 | 7.66E-05 | 1 | 0.009454847 |
| 228390_at | NA | NA | 1 | 7.83E-05 | 1 | 3.53E-05 |
| 223059_s_at | FAM107B | family with sequence similarity 107, member B | 1 | 7.83E-05 | 0.992845533 | 0.757924823 |
| 218625_at | NRN1 | neuritin 1 | 1 | 7.88E-05 | 1 | 0.001310221 |
| 212415_at | SEPT6 | septin 6 | 1 | 7.90E-05 | 1 | 0.000172266 |
| 216033_s_at | FYN | FYN oncogene related to SRC, FGR, YES | 1 | 7.90E-05 | 1 | 0.757924823 |
| 204118_at | CD48 | CD48 molecule | 1 | 8.27E-05 | 1 | 0.019077121 |
| 212660_at | PHF15 | PHD finger protein 15 | 1 | 8.62E-05 | 1 | 0.084757099 |
| 213388_at | LOC727942 | similar to phosphodiesterase 4D interacting protein isoform 2 | 1 | 9.83E-05 | 1 | 0.24676356 |
| 1552315_at | GIMAP1 | GTPase, IMAP family member 1 | 1 | 9.83E-05 | 1 | 0.457328944 |
| 1552634_a_at | ZNF101 | zinc finger protein 101 | 1 | 9.98E-05 | 0.80026495 | 0.182244751 |
| 204923_at | CXorf9 | chromosome X open reading frame 9 | 1 | 0.000104918 | 1 | 0.00208074 |
| 236293_at | RHOH | ras homolog gene family, member H | 1 | 0.000110963 | 1 | 7.11E-05 |
| 209083_at | CORO1A | coronin, actin binding protein, 1A | 1 | 0.0001116 | 0.947324676 | 0.162796113 |
| 220577_at | GVIN1 | GTPase, very large interferon inducible 1 | 1 | 0.00011225 | 1 | 0.000273327 |
| 228471_at | ANKRD44 | ankyrin repeat domain 44 | 1 | 0.00011418 | 1 | 0.002709222 |
| 244061_at | ARHGAP15 | Rho GTPase activating protein 15 | 1 | 0.000121582 | 1 | 0.002567996 |
| 228268_at | FMO2 | flavin containing monooxygenase 2 (non-functional) | 1 | 0.000123187 | 1 | 0.933848863 |
| 239050_s_at | NA | NA | 1 | 0.000123187 | 1 | 0.006982834 |
| 207957_s_at | PRKCB1 | protein kinase C, beta 1 | 1 | 0.000125351 | 1 | 9.60E-05 |
| 212774_at | ZNF238 | zinc finger protein 238 | 1 | 0.000131753 | 0.993243818 | 0.757924823 |
| 204116_at | IL2RG | interleukin 2 receptor, gamma (severe combined immunodeficiency) | 1 | 0.000131753 | 0.90906223 | 0.005628695 |
| 203923_s_at | CYBB | cytochrome b-245, beta polypeptide (chronic granulomatous disease) | 1 | 0.00013477 | 0.76149608 | 0.757924823 |
| 221648_s_at | NA | NA | 1 | 0.000144957 | 0.991535652 | 0.023181495 |
| 231235_at | NKTR | natural killer-tumor recognition sequence | 1 | 0.000148074 | 1 | 0.157100116 |
| 203760_s_at | SLA | Src-like-adaptor | 1 | 0.000148074 | 0.963462877 | 0.149254951 |
| 215073_s_at | NR2F2 | nuclear receptor subfamily 2, group F, member 2 | 1 | 0.000156255 | 1 | 0.757924823 |
| 212332_at | RBL2 | retinoblastoma-like 2 (p130) | 1 | 0.00015776 | 1 | 0.000197125 |
| 231093_at | FCRL3 | Fc receptor-like 3 | 1 | 0.00015776 | 0.939245239 | 0.008161908 |
| 228693_at | CCDC50 | coiled-coil domain containing 50 | 1 | 0.000165536 | 1 | 0.00164207 |
| 220059_at | STAP1 | signal transducing adaptor family member 1 | 1 | 0.000176198 | 1 | 0.000110155 |
| 238604_at | NA | NA | 1 | 0.000177416 | 0.863852643 | 0.021208239 |
| 209199_s_at | MEF2C | myocyte enhancer factor 2C | 1 | 0.000177416 | 1 | 0.182904799 |
| 227525_at | GLCCI1 | glucocorticoid induced transcript 1 | 1 | 0.000178937 | 0.76149608 | 0.000112432 |
| 221004_s_at | ITM2C | integral membrane protein 2C | 1 | 0.000181253 | 0.781440857 | 0.000298113 |
| 203382_s_at | APOE | apolipoprotein E | 1 | 0.000181314 | 0.193106401 | 0.450605568 |
| 204774_at | EVI2A | ecotropic viral integration site 2A | 1 | 0.000182869 | 0.990977527 | 0.668597225 |
| 1558486_at | ZNF493 | zinc finger protein 493 | 1 | 0.000191324 | 1 | 0.000527488 |
| 240008_at | ARID1B | AT rich interactive domain 1B (SWI1-like) | 1 | 0.000201784 | 1 | 0.017770532 |
| 237839_at | ANK3 | ankyrin 3, node of Ranvier (ankyrin G) | 1 | 0.00020305 | 1 | 0.055731724 |
| 221249_s_at | FAM117A | family with sequence similarity 117, member A | 1 | 0.000205883 | 1 | 0.008327759 |
| 227368_at | C6orf166 | chromosome 6 open reading frame 166 | 1 | 0.000214481 | 1 | 0.002551362 |
| 231940_at | ZNF529 | zinc finger protein 529 | 1 | 0.000214596 | 1 | 0.390467101 |
| 210658_s_at | GGA2 | golgi associated, gamma adaptin ear containing, ARF binding protein 2 | 1 | 0.000216566 | 1 | 0.137352064 |
| 239049_at | NA | NA | 1 | 0.000219862 | 1 | 0.051274405 |
| 236696_at | SR140 | U2-associated SR140 protein | 1 | 0.000233758 | 1 | 0.005151495 |
| 208450_at | LGALS2 | lectin, galactoside-binding, soluble, 2 | 1 | 0.000233758 | 0.89464749 | 0.014009266 |
| 203474_at | IQGAP2 | IQ motif containing GTPase activating protein 2 | 1 | 0.000234069 | 0.863982197 | 0.757924823 |
| 228999_at | CHD2 | chromodomain helicase DNA binding protein 2 | 1 | 0.000235528 | 1 | 0.004457768 |
| 211742_s_at | EVI2B | ecotropic viral integration site 2B | 1 | 0.000236024 | 0.985012677 | 0.076051998 |
| 236079_at | DKFZp667E0512 | hypothetical protein DKFZp667E0512 | 1 | 0.00024006 | 1 | 0.102982706 |
| 205798_at | IL7R | interleukin 7 receptor | 1 | 0.000241432 | 1 | 0.757924823 |
| 229072_at | NA | NA | 1 | 0.0002429 | 1 | 0.001535364 |
| 1556658_a_at | MBNL1 | muscleblind-like (Drosophila) | 1 | 0.000244643 | 0.226525773 | 0.004883259 |
| 226906_s_at | ARHGAP9 | Rho GTPase activating protein 9 | 1 | 0.000244643 | 0.95661777 | 0.054098891 |
| 230332_at | ZCCHC7 | zinc finger, CCHC domain containing 7 | 1 | 0.000246432 | 1 | 0.000163896 |
| 226773_at | NA | NA | 1 | 0.000279836 | 1 | 0.006982834 |
| 238558_at | MBNL1 | muscleblind-like (Drosophila) | 1 | 0.000280534 | 1 | 0.026807708 |
| 210299_s_at | FHL1 | four and a half LIM domains 1 | 1 | 0.000293956 | 1 | 0.54610685 |
| 205671_s_at | HLA-DOB | major histocompatibility complex, class II, DO beta | 1 | 0.000296474 | 1 | 0.001517417 |
| 1570507_at | SFRS2IP | splicing factor, arginine/serine-rich 2, interacting protein | 1 | 0.000297893 | 1 | 0.0631577 |
| 241036_at | HPS3 | Hermansky-Pudlak syndrome 3 | 1 | 0.000309506 | 1 | 0.000604601 |
| 228599_at | MS4A1 | membrane-spanning 4-domains, subfamily A, member 1 | 1 | 0.000316342 | 1 | 4.57E-05 |
| 236265_at | SP4 | Sp4 transcription factor | 1 | 0.000316979 | 1 | 0.069721631 |
| 224451_x_at | ARHGAP9 | Rho GTPase activating protein 9 | 1 | 0.000329235 | 0.941695197 | 0.185158811 |
| 213539_at | CD3D | CD3d molecule, delta (CD3-TCR complex) | 1 | 0.000332418 | 1 | 0.085385584 |
| 201150_s_at | TIMP3 | TIMP metallopeptidase inhibitor 3 (Sorsby fundus dystrophy, pseudoinflammatory) | 1 | 0.00033678 | 0.98606178 | 0.01116613 |
| 214974_x_at | CXCL5 | chemokine (C-X-C motif) ligand 5 | 1 | 0.000342811 | 1 | 0.77032101 |
| 244791_at | UBAC2 | UBA domain containing 2 | 1 | 0.00034364 | 1 | 0.253095839 |
| 220467_at | FLJ21272 | hypothetical protein FLJ21272 | 1 | 0.000344502 | 0.76149608 | 0.086414594 |
| 221530_s_at | BHLHB3 | basic helix-loop-helix domain containing, class B, 3 | 1 | 0.000354302 | 0.76149608 | 0.000799242 |
| 228202_at | PLN | phospholamban | 1 | 0.000375514 | 1 | 0.754230436 |
| 231647_s_at | FCRL5 | Fc receptor-like 5 | 1 | 0.000385439 | 0.80225087 | 0.008235889 |
| 221756_at | PIK3IP1 | phosphoinositide-3-kinase interacting protein 1 | 1 | 0.000420901 | 1 | 0.002691185 |
| 226713_at | CCDC50 | coiled-coil domain containing 50 | 1 | 0.000429043 | 1 | 0.024529252 |
| 205659_at | HDAC9 | histone deacetylase 9 | 1 | 0.000429043 | 0.76149608 | 0.029807178 |
| 213888_s_at | TRAF3IP3 | TRAF3 interacting protein 3 | 1 | 0.00047882 | 1 | 0.032713476 |
| 203485_at | RTN1 | reticulon 1 | 1 | 0.000502518 | 0.76149608 | 0.350613505 |
| 243981_at | STK4 | serine/threonine kinase 4 | 1 | 0.000505599 | 1 | 0.00340697 |
| 227227_at | LOC728871 | hypothetical protein LOC728871 | 1 | 0.000507606 | 1 | 0.078910277 |
| 51228_at | RBM12B | RNA binding motif protein 12B | 1 | 0.000510614 | 1 | 0.757924823 |
| 231024_at | LOC572558 | hypothetical locus LOC572558 | 1 | 0.000556492 | 1 | 0.078405876 |
| 1552977_a_at | TNRC5 | trinucleotide repeat containing 5 | 1 | 0.000563438 | 0.775941902 | 0.757924823 |
| 238429_at | TMEM71 | transmembrane protein 71 | 1 | 0.000577064 | 1 | 0.757924823 |
| 212179_at | SFRS18 | splicing factor, arginine/serine-rich 18 | 1 | 0.000577064 | 1 | 0.159518058 |
| 205668_at | LY75 | lymphocyte antigen 75 | 1 | 0.000580837 | 1 | 0.408018401 |
| 203528_at | SEMA4D | sema domain, immunoglobulin domain (Ig), transmembrane domain (TM) and short cytoplasmic domain, (semaphorin) 4D | 1 | 0.00060467 | 1 | 0.105307872 |
| 210279_at | GPR18 | G protein-coupled receptor 18 | 1 | 0.000608256 | 1 | 0.000770278 |
| 221897_at | TRIM52 | tripartite motif-containing 52 | 1 | 0.000627907 | 1 | 0.032875902 |
| 229872_s_at | LOC642441 | hypothetical LOC642441 | 1 | 0.000646931 | 0.99058837 | 0.033957838 |
| 239294_at | NA | NA | 1 | 0.000669569 | 0.955505745 | 0.065125105 |
| 212829_at | PIP4K2A | phosphatidylinositol-5-phosphate 4-kinase, type II, alpha | 1 | 0.000675524 | 1 | 0.408018401 |
| 230085_at | PCYT1B | phosphate cytidylyltransferase 1, choline, beta | 1 | 0.000688174 | 1 | 0.070684288 |
| 217627_at | ZNF573 | zinc finger protein 573 | 1 | 0.000705052 | 1 | 0.004911655 |
| 221645_s_at | ZNF83 | zinc finger protein 83 | 1 | 0.000744363 | 1 | 0.050923419 |
| 204731_at | TGFBR3 | transforming growth factor, beta receptor III | 1 | 0.000746429 | 1 | 0.757924823 |
| 228528_at | NA | NA | 1 | 0.000781499 | 1 | 0.004883259 |
| 204852_s_at | PTPN7 | protein tyrosine phosphatase, non-receptor type 7 | 1 | 0.000782122 | 0.962132649 | 0.002166846 |
| 203608_at | ALDH5A1 | aldehyde dehydrogenase 5 family, member A1 (succinate-semialdehyde dehydrogenase) | 1 | 0.00079191 | 1 | 0.00252217 |
| 223562_at | PARVG | parvin, gamma | 1 | 0.000799499 | 0.90019955 | 0.024786859 |
| 223746_at | STK4 | serine/threonine kinase 4 | 1 | 0.000805537 | 1 | 0.009184994 |
| 226811_at | FAM46C | family with sequence similarity 46, member C | 1 | 0.000826742 | 0.754007941 | 6.28E-13 |
| 230141_at | ARID4A | AT rich interactive domain 4A (RBP1-like) | 1 | 0.000826742 | 1 | 0.091543015 |
| 210556_at | NFATC3 | nuclear factor of activated T-cells, cytoplasmic, calcineurin-dependent 3 | 1 | 0.000832202 | 0.984859527 | 0.047814672 |
| 222073_at | COL4A3 | collagen, type IV, alpha 3 (Goodpasture antigen) | 1 | 0.000838355 | 1 | 0.032713476 |
| 232333_at | MAML2 | mastermind-like 2 (Drosophila) | 1 | 0.000838355 | 1 | 0.446281334 |
| 205306_x_at | KMO | kynurenine 3-monooxygenase (kynurenine 3-hydroxylase) | 1 | 0.000849198 | 0.76149608 | 0.148340562 |
| 213605_s_at | LOC728411 | similar to Beta-glucuronidase precursor | 1 | 0.000879273 | 1 | 0.757924823 |
| 201008_s_at | TXNIP | thioredoxin interacting protein | 1 | 0.00090448 | 1 | 0.182288519 |
| 228345_at | CHIC1 | cysteine-rich hydrophobic domain 1 | 1 | 0.000907603 | 1 | 0.497502403 |
| 241893_at | MGAT5 | mannosyl (alpha-1,6-)-glycoprotein beta-1,6-N-acetyl-glucosaminyltransferase | 1 | 0.000923783 | 1 | 0.074063523 |
| 232543_x_at | ARHGAP9 | Rho GTPase activating protein 9 | 1 | 0.000946119 | 0.97070021 | 0.044844731 |
| 201009_s_at | TXNIP | thioredoxin interacting protein | 1 | 0.000959216 | 0.978078614 | 0.183133291 |
| 206928_at | ZNF124 | zinc finger protein 124 | 1 | 0.000966632 | 0.928514696 | 0.757924823 |
| 219295_s_at | PCOLCE2 | procollagen C-endopeptidase enhancer 2 | 1 | 0.000966632 | 1 | 0.739074633 |
| 215528_at | MGAT5 | mannosyl (alpha-1,6-)-glycoprotein beta-1,6-N-acetyl-glucosaminyltransferase | 1 | 0.000986683 | 1 | 0.032573177 |
| 208451_s_at | C4A | complement component 4A (Rodgers blood group) | 1 | 0.000995369 | 1 | 8.57E-08 |
| 1564139_at | LOC144571 | hypothetical protein LOC144571 | 1 | 0.001004097 | 1 | 0.315594638 |
| 240307_at | FUBP1 | far upstream element (FUSE) binding protein 1 | 1 | 0.001043013 | 1 | 0.221953019 |
| 244383_at | NA | NA | 1 | 0.001056169 | 1 | 0.004280721 |
| 242946_at | CD53 | CD53 molecule | 1 | 0.001057729 | 1 | 0.001310221 |
| 213160_at | DOCK2 | dedicator of cytokinesis 2 | 1 | 0.001081138 | 0.991282558 | 0.052052601 |
| 212980_at | USP34 | ubiquitin specific peptidase 34 | 1 | 0.001093293 | 1 | 0.003215747 |
| 203241_at | UVRAG | UV radiation resistance associated gene | 1 | 0.001105914 | 1 | 0.031191451 |
| 65472_at | USP39 | ubiquitin specific peptidase 39 | 1 | 0.001132126 | 1 | 0.029807178 |
| 225228_at | TMEM77 | transmembrane protein 77 | 1 | 0.001204186 | 1 | 0.654153932 |
| 226878_at | HLA-DOA | major histocompatibility complex, class II, DO alpha | 1 | 0.001250755 | 0.93165876 | 0.235498716 |
| 212486_s_at | FYN | FYN oncogene related to SRC, FGR, YES | 1 | 0.001294873 | 1 | 0.27462343 |
| 205541_s_at | GSPT2 | G1 to S phase transition 2 | 1 | 0.001294873 | 1 | 0.003399502 |
| 218870_at | ARHGAP15 | Rho GTPase activating protein 15 | 1 | 0.001304912 | 1 | 0.174197727 |
| 227646_at | NA | NA | 1 | 0.001328447 | 1 | 0.425068065 |
| 215599_at | SMA4 | glucuronidase, beta pseudogene | 1 | 0.001351486 | 0.893938559 | 0.670183694 |
| 235652_at | SCML1 | sex comb on midleg-like 1 (Drosophila) | 1 | 0.001353123 | 1 | 0.312730013 |
| 203620_s_at | FCHSD2 | FCH and double SH3 domains 2 | 1 | 0.001404694 | 0.957109528 | 0.064502009 |
| 201719_s_at | EPB41L2 | erythrocyte membrane protein band 4.1-like 2 | 1 | 0.001416451 | 0.992547707 | 0.302221398 |
| 213620_s_at | ICAM2 | intercellular adhesion molecule 2 | 1 | 0.001437951 | 1 | 0.006608736 |
| 225957_at | LOC153222 | adult retina protein | 1 | 0.001465302 | 1 | 0.287169783 |
| 203879_at | PIK3CD | phosphoinositide-3-kinase, catalytic, delta polypeptide | 1 | 0.001481846 | 0.987843153 | 0.106774383 |
| 208885_at | LCP1 | lymphocyte cytosolic protein 1 (L-plastin) | 1 | 0.001489669 | 0.975168126 | 0.413988487 |
| 204890_s_at | LCK | lymphocyte-specific protein tyrosine kinase | 1 | 0.0015017 | 0.97558506 | 0.635864731 |
| 1555613_a_at | ZAP70 | zeta-chain (TCR) associated protein kinase 70kDa | 1 | 0.001513516 | 1 | 0.084014943 |
| 207777_s_at | SP140 | SP140 nuclear body protein | 1 | 0.001519607 | 1 | 0.012782138 |
| 203549_s_at | LPL | lipoprotein lipase | 1 | 0.001535305 | 0.913956629 | 1.88E-08 |
| 1557813_at | SSBP2 | single-stranded DNA binding protein 2 | 1 | 0.001590206 | 1 | 0.025121488 |
| 205255_x_at | TCF7 | transcription factor 7 (T-cell specific, HMG-box) | 1 | 0.001590206 | 0.989480085 | 0.084848806 |
| 218805_at | GIMAP5 | GTPase, IMAP family member 5 | 1 | 0.001604639 | 1 | 0.531796269 |
| 226412_at | SFRS18 | splicing factor, arginine/serine-rich 18 | 1 | 0.001605419 | 1 | 0.046543404 |
| 238635_at | C5orf28 | chromosome 5 open reading frame 28 | 1 | 0.00160688 | 1 | 0.292748292 |
| 235556_at | LOC153222 | adult retina protein | 1 | 0.00165042 | 1 | 0.205139167 |
| 240890_at | CASP4 | caspase 4, apoptosis-related cysteine peptidase | 1 | 0.001683123 | 1 | 0.174064006 |
| 232182_at | LOC642236 | similar to FRG1 protein (FSHD region gene 1 protein) | 1 | 0.001683123 | 1 | 0.078366169 |
| 228333_at | NA | NA | 1 | 0.001683123 | 1 | 0.757924823 |
| 209894_at | LEPR | leptin receptor | 1 | 0.001683123 | 1 | 0.024264241 |
| 202156_s_at | CUGBP2 | CUG triplet repeat, RNA binding protein 2 | 1 | 0.001722053 | 1 | 0.267887839 |
| 244008_at | NA | NA | 1 | 0.001722053 | 1 | 0.757924823 |
| 221978_at | HLA-F | major histocompatibility complex, class I, F | 1 | 0.001722053 | 0.904446427 | 0.099705839 |
| 235170_at | ZNF92 | zinc finger protein 92 | 1 | 0.001722053 | 0.875325799 | 0.757924823 |
| 1554250_s_at | TRIM73 | tripartite motif-containing 73 | 1 | 0.001722053 | 1 | 0.267138646 |
| 229317_at | NA | NA | 1 | 0.001736323 | 1 | 0.178633998 |
| 1554007_at | ZNF483 | zinc finger protein 483 | 1 | 0.00175854 | 1 | 0.000282384 |
| 222281_s_at | NA | NA | 1 | 0.001780198 | 1 | 0.019829217 |
| 221264_s_at | TARDBP | TAR DNA binding protein | 1 | 0.00186301 | 1 | 0.003865461 |
| 239954_at | ZNF160 | zinc finger protein 160 | 1 | 0.00186301 | 1 | 0.085215626 |
| 229111_at | MASP2 | mannan-binding lectin serine peptidase 2 | 1 | 0.001938647 | 1 | 0.039340138 |
| 227224_at | RALGPS2 | Ral GEF with PH domain and SH3 binding motif 2 | 1 | 0.001954864 | 1 | 0.012325479 |
| 209683_at | FAM49A | family with sequence similarity 49, member A | 1 | 0.001954864 | 1 | 0.604124229 |
| 227152_at | C12orf35 | chromosome 12 open reading frame 35 | 1 | 0.001954864 | 1 | 0.225625248 |
| 240141_at | PAPD4 | PAP associated domain containing 4 | 1 | 0.00196569 | 1 | 0.06802327 |
| 223395_at | ABI3BP | ABI gene family, member 3 (NESH) binding protein | 1 | 0.00196569 | 0.997727633 | 0.757924823 |
| 239545_at | CASD1 | CAS1 domain containing 1 | 1 | 0.001978568 | 1 | 0.016473206 |
| 226625_at | TGFBR3 | transforming growth factor, beta receptor III | 1 | 0.00198884 | 1 | 0.757924823 |
| 32541_at | PPP3CC | protein phosphatase 3 (formerly 2B), catalytic subunit, gamma isoform | 1 | 0.002001928 | 1 | 0.661473099 |
| 227082_at | NA | NA | 1 | 0.002004796 | 1 | 0.757924823 |
| 229629_at | NA | NA | 1 | 0.002013546 | 1 | 0.024264241 |
| 237104_at | CTSS | cathepsin S | 1 | 0.002041834 | 0.938024327 | 0.18848277 |
| 203548_s_at | LPL | lipoprotein lipase | 1 | 0.002041834 | 1 | 2.27E-06 |
| 244414_at | MAML2 | mastermind-like 2 (Drosophila) | 1 | 0.002041834 | 1 | 0.362586091 |
| 64064_at | GIMAP5 | GTPase, IMAP family member 5 | 1 | 0.002053385 | 1 | 0.757924823 |
| 232889_at | GUSBP1 | glucuronidase, beta pseudogene 1 | 1 | 0.002053401 | 1 | 0.502844431 |
| 214787_at | DENND4A | DENN/MADD domain containing 4A | 1 | 0.002069891 | 0.886244125 | 0.757924823 |
| 232204_at | EBF1 | early B-cell factor 1 | 1 | 0.002069891 | 1 | 0.068545819 |
| 1556818_at | ARID1B | AT rich interactive domain 1B (SWI1-like) | 1 | 0.002076324 | 1 | 0.354083598 |
| 244023_at | SYK | spleen tyrosine kinase | 1 | 0.002084037 | 1 | 0.004751391 |
| 227978_s_at | ZADH2 | zinc binding alcohol dehydrogenase, domain containing 2 | 1 | 0.0021952 | 1 | 0.142309078 |
| 205285_s_at | FYB | FYN binding protein (FYB-120/130) | 1 | 0.002228435 | 1 | 0.024337272 |
| 228630_at | ZNF84 | zinc finger protein 84 | 1 | 0.002228435 | 1 | 0.245456618 |
| 234260_at | CDC42SE2 | CDC42 small effector 2 | 1 | 0.002254942 | 0.849962682 | 0.081106599 |
| 244267_at | NA | NA | 1 | 0.002258909 | 1 | 0.231666346 |
| 1559067_a_at | NA | NA | 1 | 0.002258909 | 0.827591899 | 0.757924823 |
| 229389_at | ATG16L2 | ATG16 autophagy related 16-like 2 (S. cerevisiae) | 1 | 0.002297287 | 1 | 0.004586337 |
| 229391_s_at | FAM26F | family with sequence similarity 26, member F | 1 | 0.002321889 | 1 | 1 |
| 239292_at | NA | NA | 1 | 0.002321889 | 1 | 0.019669133 |
| 1558410_s_at | IMMP2L | IMP2 inner mitochondrial membrane peptidase-like (S. cerevisiae) | 1 | 0.002335097 | 1 | 0.048962612 |
| 201539_s_at | FHL1 | four and a half LIM domains 1 | 1 | 0.002335516 | 1 | 0.575398115 |
| 211085_s_at | STK4 | serine/threonine kinase 4 | 1 | 0.002470894 | 1 | 0.472561761 |
| 226633_at | RAB8B | RAB8B, member RAS oncogene family | 1 | 0.002491204 | 0.914873838 | 0.757924823 |
| 209606_at | PSCDBP | pleckstrin homology, Sec7 and coiled-coil domains, binding protein | 1 | 0.002514152 | 0.9435547 | 0.416106928 |
| 205861_at | SPIB | Spi-B transcription factor (Spi-1/PU.1 related) | 1 | 0.002544071 | 0.996558985 | 0.016201476 |
| 232722_at | RNASET2 | ribonuclease T2 | 1 | 0.002544071 | 1 | 0.333362771 |
| 239393_at | PAN3 | PAN3 polyA specific ribonuclease subunit homolog (S. cerevisiae) | 1 | 0.002546507 | 1 | 0.012581045 |
| 205407_at | RECK | reversion-inducing-cysteine-rich protein with kazal motifs | 1 | 0.002550197 | 1 | 0.761941401 |
| 238649_at | PITPNC1 | phosphatidylinositol transfer protein, cytoplasmic 1 | 1 | 0.002604895 | 1 | 0.757924823 |
| 240168_at | XPO7 | exportin 7 | 1 | 0.002638284 | 1 | 0.577582048 |
| 227663_at | LOC728555 | hypothetical protein LOC728555 | 1 | 0.002666334 | 1 | 0.757924823 |
| 213940_s_at | FNBP1 | formin binding protein 1 | 1 | 0.002676666 | 1 | 0.449697373 |
| 213603_s_at | RAC2 | ras-related C3 botulinum toxin substrate 2 (rho family, small GTP binding protein Rac2) | 1 | 0.002724268 | 0.930883745 | 0.216443883 |
| 225282_at | SMAP1L | stromal membrane-associated protein 1-like | 1 | 0.002724268 | 0.961232036 | 0.272965225 |
| 242343_x_at | ZNF518 | zinc finger protein 518 | 1 | 0.00272566 | 1 | 0.059508668 |
| 226751_at | C2orf32 | chromosome 2 open reading frame 32 | 1 | 0.002743559 | 1 | 0.202029894 |
| 239301_at | LOC644285 | hypothetical LOC644285 | 1 | 0.002768997 | 1 | 0.433726696 |
| 202124_s_at | TRAK2 | trafficking protein, kinesin binding 2 | 1 | 0.002776082 | 1 | 0.757924823 |
| 35974_at | LRMP | lymphoid-restricted membrane protein | 1 | 0.002813284 | 0.995849058 | 0.040281744 |
| 1569652_at | MLLT3 | myeloid/lymphoid or mixed-lineage leukemia (trithorax homolog, Drosophila); translocated to, 3 | 1 | 0.002814084 | 1 | 0.757924823 |
| 230970_at | SSH2 | slingshot homolog 2 (Drosophila) | 1 | 0.00284668 | 1 | 0.035541722 |
| 230735_at | IFNAR2 | interferon (alpha, beta and omega) receptor 2 | 1 | 0.002883043 | 1 | 0.055815349 |
| 220005_at | P2RY13 | purinergic receptor P2Y, G-protein coupled, 13 | 1 | 0.002971363 | 0.845102023 | 0.757924823 |
| 1552343_s_at | PDE7A | phosphodiesterase 7A | 1 | 0.003062407 | 0.864135621 | 0.346859351 |
| 205267_at | POU2AF1 | POU class 2 associating factor 1 | 1 | 0.003079095 | 0.900325291 | 0.000110155 |
| 1560396_at | KLHL6 | kelch-like 6 (Drosophila) | 1 | 0.003087325 | 1 | 0.158772536 |
| 213258_at | TFPI | tissue factor pathway inhibitor (lipoprotein-associated coagulation inhibitor) | 1 | 0.003087325 | 1 | 0.757924823 |
| 64418_at | NA | NA | 1 | 0.003113173 | 0.996983929 | 0.242838483 |
| 203680_at | PRKAR2B | protein kinase, cAMP-dependent, regulatory, type II, beta | 1 | 0.003155472 | 1 | 0.074683368 |
| 213122_at | TSPYL5 | TSPY-like 5 | 1 | 0.003196419 | 1 | 0.757924823 |
| 235535_x_at | C20orf80 | chromosome 20 open reading frame 80 | 1 | 0.003300643 | 0.971545273 | 0.073991742 |
| 206983_at | CCR6 | chemokine (C-C motif) receptor 6 | 1 | 0.003309609 | 1 | 0.019979693 |
| 219505_at | CECR1 | cat eye syndrome chromosome region, candidate 1 | 1 | 0.00343054 | 0.911918279 | 0.031191451 |
| 229588_at | DNAJC10 | DnaJ (Hsp40) homolog, subfamily C, member 10 | 1 | 0.00343054 | 1 | 0.074063523 |
| 206042_x_at | SNURF | SNRPN upstream reading frame | 1 | 0.003433884 | 0.963806601 | 0.534636003 |
| 238706_at | PAPD4 | PAP associated domain containing 4 | 1 | 0.003440813 | 1 | 0.069820573 |
| 209337_at | PSIP1 | PC4 and SFRS1 interacting protein 1 | 1 | 0.003497073 | 1 | 0.552670969 |
| 205987_at | CD1C | CD1c molecule | 1 | 0.003503712 | 1 | 0.10908443 |
| 206848_at | HOXA7 | homeobox A7 | 1 | 0.003503712 | 1 | 0.124004619 |
| 235008_at | ANKRD10 | ankyrin repeat domain 10 | 1 | 0.003539401 | 1 | 0.143067973 |
| 207522_s_at | ATP2A3 | ATPase, Ca++ transporting, ubiquitous | 1 | 0.003551764 | 0.947953398 | 0.057561111 |
| 1569225_a_at | SCML4 | sex comb on midleg-like 4 (Drosophila) | 1 | 0.003604963 | 1 | 0.062427307 |
| 233510_s_at | PARVG | parvin, gamma | 1 | 0.003604963 | 1 | 0.037745813 |
| 204949_at | ICAM3 | intercellular adhesion molecule 3 | 1 | 0.003650457 | 0.962196824 | 0.077735183 |
| 209459_s_at | ABAT | 4-aminobutyrate aminotransferase | 1 | 0.003657964 | 0.76149608 | 0.17932277 |
| 223588_at | THAP2 | THAP domain containing, apoptosis associated protein 2 | 1 | 0.003662954 | 1 | 0.523305939 |
| 1558972_s_at | C6orf190 | chromosome 6 open reading frame 190 | 1 | 0.003717035 | 1 | 0.486394982 |
| 1553165_at | AP1GBP1 | AP1 gamma subunit binding protein 1 | 1 | 0.003744365 | 0.811306939 | 0.330962078 |
| 236545_at | PPP3CA | protein phosphatase 3 (formerly 2B), catalytic subunit, alpha isoform | 1 | 0.003779787 | 1 | 0.757924823 |
| 227877_at | C5orf39 | chromosome 5 open reading frame 39 | 1 | 0.003779787 | 1 | 0.143067973 |
| 232210_at | BCL2 | B-cell CLL/lymphoma 2 | 1 | 0.003825894 | 1 | 0.005000396 |
| 227198_at | AFF3 | AF4/FMR2 family, member 3 | 1 | 0.00384073 | 1 | 0.003925734 |
| 206636_at | RASA2 | RAS p21 protein activator 2 | 1 | 0.003866309 | 1 | 0.301941515 |
| 212188_at | KCTD12 | potassium channel tetramerisation domain containing 12 | 1 | 0.003883938 | 1 | 0.26028675 |
| 215392_at | USP3 | ubiquitin specific peptidase 3 | 1 | 0.003915652 | 1 | 0.537808853 |
| 206219_s_at | VAV1 | vav 1 guanine nucleotide exchange factor | 1 | 0.003915652 | 1 | 0.178652685 |
| 221796_at | NTRK2 | neurotrophic tyrosine kinase, receptor, type 2 | 1 | 0.003921039 | 1 | 0.757924823 |
| 229985_at | BTNL9 | butyrophilin-like 9 | 1 | 0.00397382 | 1 | 0.003161416 |
| 238303_at | STT3B | STT3, subunit of the oligosaccharyltransferase complex, homolog B (S. cerevisiae) | 1 | 0.004144304 | 1 | 0.076495197 |
| 243527_at | RAP1A | RAP1A, member of RAS oncogene family | 1 | 0.004182307 | 1 | 0.004244358 |
| 228105_at | SAPS3 | SAPS domain family, member 3 | 1 | 0.004182307 | 1 | 0.071841291 |
| 227771_at | LIFR | leukemia inhibitory factor receptor alpha | 1 | 0.004182307 | 1 | 0.005437859 |
| 225420_at | GPAM | glycerol-3-phosphate acyltransferase, mitochondrial | 1 | 0.004182307 | 1 | 6.64E-06 |
| 205961_s_at | PSIP1 | PC4 and SFRS1 interacting protein 1 | 1 | 0.004182307 | 1 | 0.509160213 |
| 232489_at | CCDC76 | coiled-coil domain containing 76 | 1 | 0.004182307 | 1 | 0.256691227 |
| 209936_at | RBM5 | RNA binding motif protein 5 | 1 | 0.004194631 | 1 | 0.051274405 |
| 217989_at | HSD17B11 | hydroxysteroid (17-beta) dehydrogenase 11 | 1 | 0.004233473 | 1 | 0.544930888 |
| 241885_at | TAF15 | TAF15 RNA polymerase II, TATA box binding protein (TBP)-associated factor, 68kDa | 1 | 0.004257045 | 1 | 0.037745813 |
| 219778_at | ZFPM2 | zinc finger protein, multitype 2 | 1 | 0.004263033 | 1 | 4.64E-05 |
| 236379_at | EPB41 | erythrocyte membrane protein band 4.1 (elliptocytosis 1, RH-linked) | 1 | 0.004282267 | 1 | 0.047892552 |
| 234081_at | SETBP1 | SET binding protein 1 | 1 | 0.004282267 | 1 | 0.08645175 |
| 205419_at | EBI2 | Epstein-Barr virus induced gene 2 (lymphocyte-specific G protein-coupled receptor) | 1 | 0.004282267 | 1 | 0.759079083 |
| 207996_s_at | C18orf1 | chromosome 18 open reading frame 1 | 1 | 0.004365117 | 0.925036735 | 0.757924823 |
| 208442_s_at | ATM | ataxia telangiectasia mutated | 1 | 0.004436621 | 0.76149608 | 0.051274405 |
| 235735_at | TNFSF8 | tumor necrosis factor (ligand) superfamily, member 8 | 1 | 0.004439435 | 1 | 0.007488649 |
| 213566_at | RNASE6 | ribonuclease, RNase A family, k6 | 1 | 0.004439435 | 0.76149608 | 0.591865189 |
| 205885_s_at | ITGA4 | integrin, alpha 4 (antigen CD49D, alpha 4 subunit of VLA-4 receptor) | 1 | 0.00444476 | 0.937209908 | 0.259199997 |
| 212316_at | NUP210 | nucleoporin 210kDa | 1 | 0.004477371 | 1 | 0.060171747 |
| 204606_at | CCL21 | chemokine (C-C motif) ligand 21 | 1 | 0.004514936 | 1 | 0.000353241 |
| 210162_s_at | NFATC1 | nuclear factor of activated T-cells, cytoplasmic, calcineurin-dependent 1 | 1 | 0.004585612 | 1 | 0.693895972 |
| 240016_at | SENP6 | SUMO1/sentrin specific peptidase 6 | 1 | 0.004585612 | 1 | 0.236762724 |
| 1555355_a_at | ETS1 | v-ets erythroblastosis virus E26 oncogene homolog 1 (avian) | 1 | 0.004650256 | 0.968371766 | 0.757924823 |
| 213906_at | MYBL1 | v-myb myeloblastosis viral oncogene homolog (avian)-like 1 | 1 | 0.004726242 | 0.991278738 | 1.22E-06 |
| 1563674_at | FCRL2 | Fc receptor-like 2 | 1 | 0.004767884 | 0.998883167 | 0.007317848 |
| 205298_s_at | BTN2A2 | butyrophilin, subfamily 2, member A2 | 1 | 0.004767884 | 1 | 0.152652354 |
| 236649_at | DTWD1 | DTW domain containing 1 | 1 | 0.004781603 | 1 | 0.074063523 |
| 209829_at | C6orf32 | chromosome 6 open reading frame 32 | 1 | 0.004781997 | 1 | 0.104271563 |
| 202242_at | TSPAN7 | tetraspanin 7 | 1 | 0.004820316 | 1 | 0.121967993 |
| 233219_at | MKLN1 | muskelin 1, intracellular mediator containing kelch motifs | 1 | 0.004829035 | 1 | 0.551145544 |
| 205945_at | IL6R | interleukin 6 receptor | 1 | 0.00486921 | 0.961283846 | 0.003923928 |
| 230836_at | ST8SIA4 | ST8 alpha-N-acetyl-neuraminide alpha-2,8-sialyltransferase 4 | 1 | 0.004872314 | 0.96693345 | 0.757924823 |
| 235374_at | MDH1 | malate dehydrogenase 1, NAD (soluble) | 1 | 0.004872314 | 1 | 0.091930041 |
| 220739_s_at | CNNM3 | cyclin M3 | 1 | 0.004901673 | 1 | 0.006813852 |
| 222313_at | CNOT2 | CCR4-NOT transcription complex, subunit 2 | 1 | 0.004913058 | 1 | 0.628292522 |
| 204912_at | IL10RA | interleukin 10 receptor, alpha | 1 | 0.004946839 | 0.892126542 | 0.757924823 |
| 225816_at | PHF17 | PHD finger protein 17 | 1 | 0.004948379 | 1 | 0.003923928 |
| 209750_at | NR1D2 | nuclear receptor subfamily 1, group D, member 2 | 1 | 0.004973498 | 1 | 0.148452446 |
| 209613_s_at | ADH1B | alcohol dehydrogenase IB (class I), beta polypeptide | 1 | 0.005068311 | 1 | 0.552670969 |
| 226934_at | NA | NA | 1 | 0.005068311 | 1 | 0.183647552 |
| 227931_at | NA | NA | 1 | 0.005075319 | 1 | 0.465114148 |
| 229308_at | NA | NA | 1 | 0.005075406 | 1 | 0.006813852 |
| 227677_at | JAK3 | Janus kinase 3 (a protein tyrosine kinase, leukocyte) | 1 | 0.005145711 | 0.823391391 | 0.397021788 |
| 226218_at | IL7R | interleukin 7 receptor | 1 | 0.005158777 | 0.951171607 | 0.757924823 |
| 226219_at | ARHGAP30 | Rho GTPase activating protein 30 | 1 | 0.005162883 | 0.910289761 | 0.3372705 |
| 228465_at | NA | NA | 1 | 0.005170013 | 1 | 0.021208239 |
| 206201_s_at | MEOX2 | mesenchyme homeobox 2 | 1 | 0.005197234 | 1 | 0.134189061 |
| 211795_s_at | FYB | FYN binding protein (FYB-120/130) | 1 | 0.00526585 | 0.883121005 | 0.368132434 |
| 210202_s_at | BIN1 | bridging integrator 1 | 1 | 0.00526585 | 1 | 0.027985369 |
| 214551_s_at | CD7 | CD7 molecule | 1 | 0.00526585 | 0.984449745 | 0.757924823 |
| 217838_s_at | EVL | Enah/Vasp-like | 1 | 0.005397437 | 0.76149608 | 0.448662405 |
| 231283_at | MGAT4A | mannosyl (alpha-1,3-)-glycoprotein beta-1,4-N-acetylglucosaminyltransferase, isozyme A | 1 | 0.005402201 | 0.992503967 | 0.59282793 |
| 244153_at | NA | NA | 1 | 0.005420765 | 1 | 0.143494623 |
| 217540_at | NA | NA | 1 | 0.005461055 | 0.942839651 | 0.079279038 |
| 1559490_at | LRCH3 | leucine-rich repeats and calponin homology (CH) domain containing 3 | 1 | 0.005461055 | 1 | 0.006387276 |
| 207977_s_at | DPT | dermatopontin | 1 | 0.005494134 | 1 | 1 |
| 225809_at | DKFZP564O0823 | DKFZP564O0823 protein | 1 | 0.005520011 | 1 | 0.047106841 |
| 1552790_a_at | TLOC1 | translocation protein 1 | 1 | 0.005524078 | 1 | 0.345123624 |
| 217028_at | CXCR4 | chemokine (C-X-C motif) receptor 4 | 1 | 0.005592104 | 0.955507234 | 0.757924823 |
| 1562761_at | C9orf95 | chromosome 9 open reading frame 95 | 1 | 0.005636278 | 1 | 0.350593705 |
| 221011_s_at | LBH | limb bud and heart development homolog (mouse) | 1 | 0.005638168 | 0.926104639 | 0.757924823 |
| 225571_at | LIFR | leukemia inhibitory factor receptor alpha | 1 | 0.005639943 | 1 | 0.000965958 |
| 228097_at | MYLIP | myosin regulatory light chain interacting protein | 1 | 0.005771924 | 1 | 0.467323692 |
| 228532_at | C1orf162 | chromosome 1 open reading frame 162 | 1 | 0.00593744 | 1 | 0.757924823 |
| 204328_at | TMC6 | transmembrane channel-like 6 | 1 | 0.00593744 | 0.985406412 | 0.189039359 |
| 236301_at | NA | NA | 1 | 0.00593744 | 1 | 0.023579937 |
| 234366_x_at | IGL@ | immunoglobulin lambda locus | 1 | 0.005952829 | 0.76149608 | 0.019979693 |
| 204749_at | NAP1L3 | nucleosome assembly protein 1-like 3 | 1 | 0.006016954 | 1 | 0.757924823 |
| 213587_s_at | ATP6V0E2 | ATPase, H+ transporting V0 subunit e2 | 1 | 0.006016954 | 1 | 0.706037354 |
| 213982_s_at | RABGAP1L | RAB GTPase activating protein 1-like | 1 | 0.006061064 | 0.985406412 | 0.118152074 |
| 230142_s_at | CIRBP | cold inducible RNA binding protein | 1 | 0.006061064 | 1 | 0.008141454 |
| 222001_x_at | LOC728855 | hypothetical protein LOC728855 | 1 | 0.006092704 | 0.795065212 | 0.402108084 |
| 204688_at | SGCE | sarcoglycan, epsilon | 1 | 0.006110237 | 0.89225284 | 0.395856374 |
| 239723_at | SLC40A1 | solute carrier family 40 (iron-regulated transporter), member 1 | 1 | 0.006126951 | 1 | 0.001378468 |
| 1558105_a_at | NA | NA | 1 | 0.006126951 | 0.960521657 | 0.029880289 |
| 227593_at | FLJ37453 | hypothetical protein LOC645580 | 1 | 0.006140597 | 1 | 0.757924823 |
| 237741_at | SLC25A36 | solute carrier family 25, member 36 | 1 | 0.00626071 | 1 | 0.526815319 |
| 243109_at | MCTP2 | multiple C2 domains, transmembrane 2 | 1 | 0.006336784 | 1 | 0.082291032 |
| 201580_s_at | TXNDC13 | thioredoxin domain containing 13 | 1 | 0.006352344 | 1 | 0.757924823 |
| 224873_s_at | MRPS25 | mitochondrial ribosomal protein S25 | 1 | 0.006396934 | 1 | 0.287402738 |
| 216069_at | PRMT2 | protein arginine methyltransferase 2 | 1 | 0.006411608 | 1 | 0.757924823 |
| 1555846_a_at | NA | NA | 1 | 0.006411608 | 1 | 0.121321092 |
| 217655_at | FXYD5 | FXYD domain containing ion transport regulator 5 | 1 | 0.006411608 | 1 | 0.002380164 |
| 232617_at | CTSS | cathepsin S | 1 | 0.006462317 | 0.76149608 | 0.757924823 |
| 205382_s_at | CFD | complement factor D (adipsin) | 1 | 0.006515148 | 1 | 1 |
| 238785_at | C3orf63 | chromosome 3 open reading frame 63 | 1 | 0.006549755 | 0.989480085 | 0.145554127 |
| 219563_at | C14orf139 | chromosome 14 open reading frame 139 | 1 | 0.00655907 | 1 | 0.048962612 |
| 209723_at | SERPINB9 | serpin peptidase inhibitor, clade B (ovalbumin), member 9 | 1 | 0.00655907 | 1 | 0.757924823 |
| 230925_at | APBB1IP | amyloid beta (A4) precursor protein-binding, family B, member 1 interacting protein | 1 | 0.006592301 | 0.860325282 | 0.104856938 |
| 209539_at | ARHGEF6 | Rac/Cdc42 guanine nucleotide exchange factor (GEF) 6 | 1 | 0.006668394 | 0.996294934 | 0.545112662 |
| 241751_at | OFD1 | oral-facial-digital syndrome 1 | 1 | 0.006668394 | 1 | 0.451688697 |
| 221601_s_at | FAIM3 | Fas apoptotic inhibitory molecule 3 | 1 | 0.006669172 | 0.995932236 | 0.022337065 |
| 223640_at | HCST | hematopoietic cell signal transducer | 1 | 0.006738768 | 0.921547755 | 0.757924823 |
| 209607_x_at | SULT1A3 | sulfotransferase family, cytosolic, 1A, phenol-preferring, member 3 | 1 | 0.006738768 | 1 | 0.006867417 |
| 210858_x_at | ATM | ataxia telangiectasia mutated | 1 | 0.00680745 | 0.76149608 | 0.043033299 |
| 1565703_at | SMAD4 | SMAD family member 4 | 1 | 0.006859872 | 0.76149608 | 0.078366169 |
| 226362_at | SERF1A | small EDRK-rich factor 1A (telomeric) | 1 | 0.006859872 | 1 | 0.757924823 |
| 1557293_at | LOC440993 | hypothetical gene supported by AK128346 | 1 | 0.006859872 | 0.78457813 | 0.757924823 |
| 213029_at | NFIB | nuclear factor I/B | 1 | 0.006859872 | 1 | 0.757924823 |
| 233461_x_at | ZNF226 | zinc finger protein 226 | 1 | 0.006859872 | 1 | 0.100367053 |
| 228963_at | NA | NA | 1 | 0.006894589 | 1 | 0.165732046 |
| 236314_at | NA | NA | 1 | 0.006959361 | 1 | 0.020796237 |
| 229694_at | BRWD2 | bromodomain and WD repeat domain containing 2 | 1 | 0.006966528 | 1 | 0.099705839 |
| 228287_at | LOC727773 | similar to inhibitor of growth family, member 5 | 1 | 0.006990251 | 1 | 0.465114148 |
| 237895_at | TNRC6B | trinucleotide repeat containing 6B | 1 | 0.00703022 | 1 | 0.019669133 |
| 204265_s_at | GPSM3 | G-protein signaling modulator 3 (AGS3-like, C. elegans) | 1 | 0.007038759 | 0.966483976 | 0.051789916 |
| 226808_at | LOC643641 | hypothetical protein LOC643641 | 1 | 0.007132319 | 1 | 0.001310221 |
| 209469_at | GPM6A | glycoprotein M6A | 1 | 0.007132319 | 1 | 0.757924823 |
| 231111_at | C1orf112 | chromosome 1 open reading frame 112 | 1 | 0.007132319 | 1 | 0.757924823 |
| 238860_at | C6orf130 | chromosome 6 open reading frame 130 | 1 | 0.007132319 | 1 | 0.073225865 |
| 202760_s_at | PALM2-AKAP2 | PALM2-AKAP2 protein | 1 | 0.007294749 | 0.996545693 | 0.757924823 |
| 212187_x_at | PTGDS | prostaglandin D2 synthase 21kDa (brain) | 1 | 0.007313937 | 1 | 0.757924823 |
| 205756_s_at | F8 | coagulation factor VIII, procoagulant component (hemophilia A) | 1 | 0.00735241 | 1 | 0.490240046 |
| 1555852_at | PSMB9 | proteasome (prosome, macropain) subunit, beta type, 9 (large multifunctional peptidase 2) | 1 | 0.007498311 | 0.971149621 | 0.333738195 |
| 226458_at | NA | NA | 1 | 0.007590071 | 1 | 0.368427914 |
| 205392_s_at | CCL14 | chemokine (C-C motif) ligand 14 | 1 | 0.007654242 | 1 | 0.574084972 |
| 241917_at | FCHSD2 | FCH and double SH3 domains 2 | 1 | 0.00766386 | 1 | 0.058006214 |
| 244375_at | EVL | Enah/Vasp-like | 1 | 0.007696013 | 1 | 0.095619488 |
| 1561884_at | CEPT1 | choline/ethanolamine phosphotransferase 1 | 1 | 0.007698131 | 1 | 0.241545103 |
| 1564077_at | NA | NA | 1 | 0.007830898 | 1 | 0.06880435 |
| 229437_at | BIC | BIC transcript | 1 | 0.007830898 | 0.765180009 | 0.205091153 |
| 232001_at | LOC439949 | hypothetical gene supported by AY007155 | 1 | 0.007910809 | 1 | 0.187916272 |
| 206965_at | KLF12 | Kruppel-like factor 12 | 1 | 0.007943773 | 1 | 0.054098891 |
| 235810_at | ZNF182 | zinc finger protein 182 | 1 | 0.008004921 | 1 | 0.487619977 |
| 217147_s_at | TRAT1 | T cell receptor associated transmembrane adaptor 1 | 1 | 0.008095098 | 1 | 0.191805265 |
| 204160_s_at | ENPP4 | ectonucleotide pyrophosphatase/phosphodiesterase 4 (putative function) | 1 | 0.008105623 | 1 | 0.469483299 |
| 205831_at | CD2 | CD2 molecule | 1 | 0.008132775 | 0.904128164 | 0.757924823 |
| 228562_at | NA | NA | 1 | 0.008132775 | 1 | 0.617312549 |
| 1553856_s_at | P2RY10 | purinergic receptor P2Y, G-protein coupled, 10 | 1 | 0.008153431 | 1 | 0.098874003 |
| 201540_at | FHL1 | four and a half LIM domains 1 | 1 | 0.008155935 | 1 | 0.757924823 |
| 227485_at | DDX26B | DEAD/H (Asp-Glu-Ala-Asp/His) box polypeptide 26B | 1 | 0.008347826 | 0.954524274 | 0.169855766 |
| 233089_at | QRSL1 | glutaminyl-tRNA synthase (glutamine-hydrolyzing)-like 1 | 1 | 0.008477217 | 1 | 0.192091788 |
| 221834_at | LONP2 | lon peptidase 2, peroxisomal | 1 | 0.008477217 | 1 | 0.110222693 |
| 239646_at | RAPGEF6 | Rap guanine nucleotide exchange factor (GEF) 6 | 1 | 0.008493672 | 1 | 0.221245682 |
| 239895_at | AQR | aquarius homolog (mouse) | 1 | 0.008547785 | 1 | 0.757924823 |
| 224583_at | COTL1 | coactosin-like 1 (Dictyostelium) | 1 | 0.008547785 | 0.76149608 | 0.757924823 |
| 203217_s_at | ST3GAL5 | ST3 beta-galactoside alpha-2,3-sialyltransferase 5 | 1 | 0.008547785 | 0.944935296 | 0.757924823 |
| 219667_s_at | BANK1 | B-cell scaffold protein with ankyrin repeats 1 | 1 | 0.008551018 | 1 | 0.001601145 |
| 209754_s_at | TMPO | thymopoietin | 1 | 0.008551018 | 0.927342404 | 0.757924823 |
| 235372_at | FCRLA | Fc receptor-like A | 1 | 0.008636033 | 0.997397051 | 0.004280721 |
| 226333_at | NA | NA | 1 | 0.008636033 | 0.991913871 | 0.303358274 |
| 225564_at | SPATA13 | spermatogenesis associated 13 | 1 | 0.008646631 | 0.926488203 | 0.757924823 |
| 226659_at | DEF6 | differentially expressed in FDCP 6 homolog (mouse) | 1 | 0.008679688 | 0.911450463 | 0.314962808 |
| 230669_at | RASA2 | RAS p21 protein activator 2 | 1 | 0.008679688 | 1 | 0.164150171 |
| 218353_at | RGS5 | regulator of G-protein signaling 5 | 1 | 0.008709455 | 1 | 0.016125925 |
| 216250_s_at | LPXN | leupaxin | 1 | 0.008709455 | 0.869138152 | 0.696714243 |
| 206181_at | SLAMF1 | signaling lymphocytic activation molecule family member 1 | 1 | 0.008824858 | 1 | 0.166959686 |
| 208617_s_at | PTP4A2 | protein tyrosine phosphatase type IVA, member 2 | 1 | 0.008901632 | 0.914386565 | 0.757924823 |
| 236198_at | NA | NA | 1 | 0.008997123 | 1 | 0.044044487 |
| 225136_at | PLEKHA2 | pleckstrin homology domain containing, family A (phosphoinositide binding specific) member 2 | 1 | 0.008997123 | 0.987210228 | 0.39612527 |
| 229968_at | TLR1 | toll-like receptor 1 | 1 | 0.009097133 | 0.871992123 | 0.551145544 |
| 230304_at | NA | NA | 1 | 0.009179884 | 0.966594941 | 0.054231213 |
| 236316_at | FAM3C | family with sequence similarity 3, member C | 1 | 0.009179884 | 1 | 0.757924823 |
| 225763_at | RCSD1 | RCSD domain containing 1 | 1 | 0.009195893 | 0.952878777 | 0.106774383 |
| 221905_at | CYLD | cylindromatosis (turban tumor syndrome) | 1 | 0.009195893 | 1 | 0.290680336 |
| 202158_s_at | CUGBP2 | CUG triplet repeat, RNA binding protein 2 | 1 | 0.009366401 | 1 | 0.757924823 |
| 233690_at | RUNX1 | runt-related transcription factor 1 (acute myeloid leukemia 1; aml1 oncogene) | 1 | 0.009366401 | 0.76149608 | 0.516221784 |
| 220132_s_at | CLEC2D | C-type lectin domain family 2, member D | 1 | 0.009392257 | 0.995027607 | 0.560040362 |
| 232583_at | NCK2 | NCK adaptor protein 2 | 1 | 0.009722304 | 1 | 0.070273199 |
| 214768_x_at | IGKC | immunoglobulin kappa constant | 1 | 0.009767307 | 0.000178236 | 5.99E-13 |
| 211105_s_at | NFATC1 | nuclear factor of activated T-cells, cytoplasmic, calcineurin-dependent 1 | 1 | 0.009795496 | 1 | 0.020174523 |
| 221748_s_at | TNS1 | tensin 1 | 1 | 0.009876948 | 1 | 0.757924823 |
| 240070_at | VSTM3 | V-set and transmembrane domain containing 3 | 1 | 0.00996675 | 0.780731464 | 0.284462114 |
| 203906_at | IQSEC1 | IQ motif and Sec7 domain 1 | 1 | 0.00996675 | 1 | 0.17813606 |
| 243589_at | KIAA1267 | KIAA1267 | 1 | 0.010743529 | 1 | 0.004288704 |
| 234276_at | USP6NL | USP6 N-terminal like | 1 | 0.011377324 | 1 | 0.004575486 |
| 1562364_at | GVIN1 | GTPase, very large interferon inducible 1 | 1 | 0.012941511 | 1 | 0.000377975 |
| 1559065_a_at | CLEC4G | C-type lectin superfamily 4, member G | 1 | 0.013139148 | 1 | 0.000527488 |
| 1556277_a_at | PAPD4 | PAP associated domain containing 4 | 1 | 0.013326769 | 1 | 0.002466998 |
| 1555779_a_at | CD79A | CD79a molecule, immunoglobulin-associated alpha | 1 | 0.013829995 | 0.99213204 | 0.001295948 |
| 210356_x_at | MS4A1 | membrane-spanning 4-domains, subfamily A, member 1 | 1 | 0.016632537 | 1 | 0.000144138 |
| 1568594_s_at | TRIM52 | tripartite motif-containing 52 | 1 | 0.021499606 | 0.76149608 | 0.007462375 |
| 206478_at | KIAA0125 | KIAA0125 | 1 | 0.021499606 | 0.925775221 | 7.23E-05 |
| 217164_at | NA | NA | 1 | 0.022277973 | 0.76149608 | 0.008629963 |
| 203920_at | NR1H3 | nuclear receptor subfamily 1, group H, member 3 | 1 | 0.02257033 | 0.76149608 | 0.000110155 |
| 209760_at | KIAA0922 | KIAA0922 | 1 | 0.028779548 | 1 | 0.004883259 |
| 207175_at | ADIPOQ | adiponectin, C1Q and collagen domain containing | 1 | 0.031445622 | 1 | 0.003215747 |
| 235476_at | TRIM59 | tripartite motif-containing 59 | 1 | 0.032395728 | 0.986154045 | 0.006813852 |
| 207536_s_at | TNFRSF9 | tumor necrosis factor receptor superfamily, member 9 | 1 | 0.032813085 | 0.793756255 | 0.000483963 |
| 235183_at | NA | NA | 1 | 0.0338224 | 0.97441754 | 0.000799242 |
| 228643_at | COMMD10 | COMM domain containing 10 | 1 | 0.034214718 | 1 | 0.000158777 |
| 241505_at | NA | NA | 1 | 0.034430039 | 1 | 0.004785479 |
| 243780_at | NA | NA | 1 | 0.037706078 | 1 | 0.003975729 |
| 204184_s_at | ADRBK2 | adrenergic, beta, receptor kinase 2 | 1 | 0.03774413 | 1 | 0.004178719 |
| 206641_at | TNFRSF17 | tumor necrosis factor receptor superfamily, member 17 | 1 | 0.040736941 | 0.76149608 | 0.000468508 |
| 242859_at | TFCP2 | transcription factor CP2 | 1 | 0.042375178 | 1 | 0.001542576 |
| 215555_at | C1orf63 | chromosome 1 open reading frame 63 | 1 | 0.043650664 | 0.950586918 | 0.001517417 |
| 212314_at | KIAA0746 | KIAA0746 protein | 1 | 0.045131155 | 0.966453781 | 0.004280721 |
| 233228_at | ZNF407 | zinc finger protein 407 | 1 | 0.063268724 | 1 | 0.005723843 |
| 205692_s_at | CD38 | CD38 molecule | 1 | 0.069422521 | 0.434962492 | 3.41E-11 |
| 205357_s_at | AGTR1 | angiotensin II receptor, type 1 | 1 | 0.075899679 | 1 | 3.28E-05 |
| 210002_at | GATA6 | GATA binding protein 6 | 1 | 0.076209115 | 1 | 0.004785479 |
| 239451_at | HSP90B1 | heat shock protein 90kDa beta (Grp94), member 1 | 1 | 0.079728959 | 0.927516178 | 0.009254822 |
| 220702_at | NA | NA | 1 | 0.081729331 | 0.939191887 | 0.009695093 |
| 230064_at | NA | NA | 1 | 0.084636078 | 1 | 0.003448083 |
| 241860_at | STK17B | serine/threonine kinase 17b | 1 | 0.097316127 | 1 | 0.006916303 |
| 1561615_s_at | SLC8A1 | solute carrier family 8 (sodium/calcium exchanger), member 1 | 1 | 0.134757783 | 0.887183699 | 0.005628695 |
| 223569_at | PPAPDC1B | phosphatidic acid phosphatase type 2 domain containing 1B | 1 | 0.163954419 | 0.76149608 | 0.000144138 |
| 205913_at | PLIN | perilipin | 1 | 0.172666786 | 1 | 0.008413255 |
| 212311_at | KIAA0746 | KIAA0746 protein | 1 | 0.206533265 | 0.846041539 | 0.000906527 |
| 239798_at | PDK1 | pyruvate dehydrogenase kinase, isozyme 1 | 1 | 0.221374482 | 0.989145969 | 0.005202472 |
| 222282_at | PAPD4 | PAP associated domain containing 4 | 1 | 0.249695835 | 1 | 0.002838597 |
| 1556183_at | FLJ40330 | similar to protein immuno-reactive with anti-PTH polyclonal antibodies | 1 | 0.352324581 | 0.76149608 | 0.002241705 |
| 237625_s_at | IGKC | immunoglobulin kappa constant | 1 | 0.396068663 | 0.762841245 | 0.003215747 |
| 223565_at | MGC29506 | hypothetical protein MGC29506 | 1 | 0.406720285 | 0.76149608 | 0.000597324 |
| 228766_at | CD36 | CD36 molecule (thrombospondin receptor) | 1 | 0.435482403 | 1 | 0.002821996 |
| 39318_at | TCL1A | T-cell leukemia/lymphoma 1A | 1 | 0.451501299 | 0.84381753 | 0.005628695 |
| 1569607_s_at | LOC643187 | similar to ankyrin repeat domain 20A | 1 | 0.518559081 | 1 | 6.47E-05 |
| 211559_s_at | CCNG2 | cyclin G2 | 1 | 0.669696654 | 1 | 0.002691185 |
| 235028_at | NA | NA | 1 | 0.835232341 | 8.95E-09 | 0.221232794 |
| 1560814_a_at | CCDC32 | coiled-coil domain containing 32 | 1 | 0.835232341 | 0.93529306 | 0.005628695 |
| 217148_x_at | IGL@ | immunoglobulin lambda locus | 1 | 0.835232341 | 0.76149608 | 0.00083288 |
| 1565743_at | PFAAP5 | phosphonoformate immuno-associated protein 5 | 1 | 0.835232341 | 2.73E-05 | 0.650936832 |
| 1557270_at | ZNF69 | zinc finger protein 69 | 1 | 0.835232341 | 0.000148425 | 0.323593643 |
| 205238_at | CXorf34 | chromosome X open reading frame 34 | 1 | 0.835232341 | 0.003747848 | 0.757924823 |
| 1563364_at | RFXDC2 | regulatory factor X domain containing 2 | 1 | 0.835232341 | 0.000893834 | 0.470286767 |
| 238883_at | MED13L | mediator complex subunit 13-like | 1 | 0.835232341 | 1 | 0.004943457 |
| 215029_at | NA | NA | 1 | 0.835232341 | 8.36E-05 | 0.757924823 |
| 1560332_at | CHST11 | carbohydrate (chondroitin 4) sulfotransferase 11 | 1 | 0.835232341 | 8.35E-06 | 0.757924823 |
| 215123_at | LOC23117 | KIAA0220-like protein | 1 | 0.835232341 | 1.20E-06 | 0.757924823 |
| 216813_at | NA | NA | 1 | 0.835232341 | 0.000103082 | 0.757924823 |
| 219159_s_at | SLAMF7 | SLAM family member 7 | 1 | 0.835232341 | 0.76149608 | 1.89E-07 |
| 239412_at | IRF5 | interferon regulatory factor 5 | 1 | 0.835232341 | 0.994962689 | 0.004147533 |
| 1558515_at | NA | NA | 1 | 0.896940861 | 0.009818308 | 0.450605568 |
| 1552986_at | LOC142937 | hypothetical protein BC008131 | 1 | 0.928344923 | 0.006833851 | 0.757924823 |
| 207735_at | RNF125 | ring finger protein 125 | 1 | 0.957216183 | 0.003896604 | 0.757924823 |
| 1569091_at | ZMYM2 | zinc finger, MYM-type 2 | 1 | 1 | 0.003096782 | 0.757924823 |
| 1558710_at | ARIH1 | ariadne homolog, ubiquitin-conjugating enzyme E2 binding protein, 1 (Drosophila) | 1 | 1 | 0.001535106 | 0.757924823 |
| 1554543_at | SPAG9 | sperm associated antigen 9 | 1 | 1 | 0.003591736 | 0.757924823 |
| 241716_at | HSPD1 | heat shock 60kDa protein 1 (chaperonin) | 1 | 1 | 0.002148196 | 0.757924823 |
| 223036_at | FARSB | phenylalanyl-tRNA synthetase, beta subunit | 1 | 1 | 0.000146112 | 0.757924823 |

Abbreviations: *P* - p value; NA - not applicable.
