# Supplementary material for: Estimation of groin recurrence risk in patients with squamous cell vulvar carcinoma by the assessment of marker gene expression in the lymph nodes
Source: BMC Cancer. 2012 Jun 6;12:223. doi: 10.1186/1471-2407-12-223 (PMC3414830; doi:10.1186/1471-2407-12-223)
Supplement: Additional file 3 — Table S1. Mean levels of expression of PERP, S100A8, FABP5, SFN, CA12, JUP and CSTA in LN(+) and LN(−) samples, measured by qRT-PCR. Abbreviations: ME – mean expression level in all involved [LN(+)] and uninvolved [LN(−)] lymph node samples included in the qRT-PCR analysis. [file 1471-2407-12-223-S1.doc]

Supplementary Table 1. Mean levels of expression of *PERP*, *S100A8*, *FABP5*, *SFN*, *CA12*, *JUP* and *CSTA* in LN(+) and LN(-) samples, measured by qRT-PCR.

| **Transcript** | **ME in LN(-) samples**  **[range]** | **ME in LN(+) samples**  **[range]** |
| --- | --- | --- |
| PERP | 0.91  [0.01 – 27.98] | 8.18  [0.14 – 40.16] |
| S100A8 | 2.01  [0.06 – 73.49] | 26.74  [0.14 – 112.53] |
| FABP5 | 3.36  [0.28 – 73.80] | 17.72  [0.70 – 94.29] |
| SFN | 0.21  [0.001 – 4.818] | 2.41  [0.002 – 10.53] |
| CA12 | 0.04  [0.003 – 0.49] | 0.21  [0.01 – 0.61] |
| JUP | 0.18  [0.03 – 2.16] | 0.45  [0.03 – 1.32] |
| CSTA | 0.02  [0.001 – 0.48] | 0.05  [0.001 – 0.33] |

Abbreviations: ME – mean expression level in all involved [LN(+)] and uninvolved [LN(-)] lymph node samples included in the qRT-PCR analysis.
